# Role of reactive oxygen species in lesion mimic formation and conferred basal resistance to *Fusarium graminearum* in barley lesion mimic mutant *5386*

**DOI:** 10.3389/fpls.2022.1020551

**Published:** 2022-10-31

**Authors:** Wenqiang Wang, Jifa Zhang, Fenxia Guo, Yindi Di, Yuhui Wang, Wankun Li, Yali Sun, Yuhai Wang, Fei Ni, Daolin Fu, Wei Wang, Qunqun Hao

**Affiliations:** ^1^ College of Life Sciences, Zaozhuang University, Zaozhuang, China; ^2^ State Key Laboratory of Crop Biology, College of Agronomy, Shandong Agricultural University, Tai’an, China; ^3^ Shandong Shofine Seed Technology Co., Ltd., Jining, China; ^4^ Spring Valley Agriscience Co., Ltd., Jinan, China; ^5^ Qihe Bureau of Agriculture and Rural, Qihe, China; ^6^ State Key Laboratory of Crop Biology, College of Life Sciences, Shandong Agricultural University, Tai’an, China

**Keywords:** lesion mimic, RNA-Seq, ROS accumulation, antioxidant competence, glutathione, barley

## Abstract

This study investigated the barley lesion mimic mutant (LMM) *5386*, evidenced by a leaf brown spot phenotype localized on the chromosome 3H, and its conferred basal resistance to *Fusarium graminearum*. RNA-seq analysis identified 1453 genes that were differentially expressed in LMM *5386* compared to those in the wild type. GO and KEGG functional annotations suggested that lesion mimic formation was mediated by pathways involving oxidation reduction and glutathione metabolism. Additionally, reactive oxygen species (ROS) accumulation in brown spots was substantially higher in LMM *5386* than in the wild-type plant; therefore, antioxidant competence, which is indicated by ROS accumulation, was significantly lower in LMM *5386*. Furthermore, the reduction of glycine in LMM *5386* inhibited glutathione biosynthesis. These results suggest that the decrease in antioxidant competence and glutathione biosynthesis caused considerable ROS accumulation, leading to programmed cell death, which eventually reduced the yield components in LMM *5386*.

## Introduction

Lesion mimics (LMs), also known as hypersensitive reaction-like traits, arise spontaneously in leaf tissues without being attacked by plant pathogens (McGrann et al., 2015). LM mutants (LMMs) spontaneously form necrotic plaques under normal growth conditions; thus, LMMs are valuable genetic resources for studying programmed cell death (PCD) signaling pathways and disease resistance in plants (Moeder and Yoshioka, 2008).

Recently, many LMMs have been identified and studied in a variety of plants including *Arabidopsis* (Serrano et al., 2010), barley (Hao et al., 2019), maize (Hurni et al., 2015), and rice (Shang et al., 2009). These previous LMM formation studies have drawn the several conclusions, including that (1) mutations and abnormal expressions of disease-resistance genes lead to hypersensitivity and subsequent PCD in plants, causing necrotic plaques similar to those caused by pathogen infection (Shirano et al., 2002); (2) the abnormal expression of PCD-controlling genes leads to the loss of control of PCD, which can lead to necrotic plaque formation (Dietrich et al., 1994); (3) plant metabolism disorders can also induce necrotic plaques in plants (Hu et al., 1998); and (4) external environmental changes can also induce plaque appearance (Wang et al., 2015a; Wang et al., 2016a).

LMMs are crucial for studying hypersensitive responses (HRs) in plants (Bruggeman et al., 2015), and the level of reactive oxygen species (ROS) has been identified as capable of producing LMMs (Sindhu et al., 2018). Hao et al. (2019) reported that autonomic lesions associated with *LMM194* were often accompanied by excessive ROS, occasionally leading to cell death. Rice containing *LMM6* were more resistant to blast fungus and had higher ROS accumulations (Xiao et al., 2015). Oxidative stress genes are also expressed in LMM lines (Devadas et al., 2002; Torres et al., 2002), as are enzymes involved in antioxidant systems such as glutathione S-transferase (GST), peroxidase (POD), and superoxide dismutase (SOD) (Klibenstein et al., 1999; Hao et al., 2019), suggesting that oxidative stress signals are activated by LM genes.

Some LMMs have been isolated in plants, most of which displaying enhanced pathogen resistance; for example, *lls1* mutations exhibit enhanced resistance to fungal pathogens in maize (Simmons et al., 1998), *spl* mutations exhibit resistance to the blast fungus in rice (Yin et al., 2000), *M66* mutations show increased resistance to yellow rust and powdery mildew (Kinane and Jones, 2001), and *lm1* and *lm2* mutations enhance leaf rust resistance in wheat (Yao et al., 2009). Furthermore, a novel light-dependent LM gene (*lm3*) shows resistance to powdery mildew in wheat (Wang et al., 2016a).

Currently, very few *Triticeae*-tribe LM genes have been cloned, with two LM genes cloned from barley, including *TIGRINA-D.12* (Khandal et al., 2009) and *NEC1* (Rostoks et al., 2006). *TIGRINA-D.12* and *NEC1* in barley are homologous to *FLU* (At3g14110) and *HLM1* (At5g54250) in *Arabidopsis thaliana*, respectively. The FLU protein regulates chlorophyll synthesis in *Arabidopsis*, with the LMM FLU protein forming LMs in mature leaves (Meskauskiene et al., 2011). Additionally, three LM genes (*lm1*, *lm2, and lm3*) have been mapped on 3BS, 4BL, and 3BL, respectively, in wheat (Yao et al., 2009; Wang et al., 2016a).

Barley is an ideal model of the *Triticeae* tribe and an economically important cereal. In this study, we identified a novel LMM *5386* in barley and investigated LM formation and the resistance to *F. graminearum*. The results are expected to improve our understanding of the role of LMMs in barley.

## Materials and methods

### Plant materials

A barley LMM 5386 line was generated through the application of 29 mmol L^−1^ ethyl methanesulfonate (EMS) to the “Tamalpais” wild type (WT) cultivar in 2009. After more than six generations of selfing, LMM 5386 line was genetically stable and showed no separation phenomenon.

The experiments were performed at Zaozhuang University, Zaozhuang, Shandong, China. Grains were sown at a density of 300 seeds m^−2^. The plot size was 2 × 2 m with six rows (0.25 m between rows). Each plot was used for sample collection. The experiments were conducted at least in triplicates.

### Bulked segregant RNA−Seq and data analysis

Leaves of 50 lesion mimic F2 lines were pooled to construct a bulk RNA sample. Morex was additionally processed as a parental check. Total RNA was extracted from each sample using TRIzol reagent following the manufacturer’s specifications (Invitrogen). The quality and quantity of each RNA sample were measured using an Agilent 2100 Bioanalyzer (Agilent Technologies, CA, USA).

The mRNAs were isolated from the total RNA using Dynabeads mRNA DIRECT Kit (Invitrogen) and were separated into short fragments using a fragmentation buffer. Using these short fragments as templates, random primers, and a SuperScript double-stranded cDNA synthesis kit (Invitrogen), double-stranded cDNA was synthesized. Ligated fragments were then generated through a series of reactions that included the purification of the PCR products, end repair, dA-tailing, and ligation of the Illumina adapters. After agarose gel electrophoresis, suitable fragments were selected for PCR amplification. The final library was evaluated using quantitative RT-PCR with a StepOne Plus Real-Time PCR system (Applied Biosystems, Foster City, CA, USA). Sequencing reactions were performed on an Illumina HiSeq 2000 (Biomarker Technologies Corporation, Beijing, China). Single nucleotide polymorphisms (SNPs) and insertion deletion polymorphisms (InDels) were called using the HaplotypeCaller module in GATK v3.2.

### RNA-seq and data analysis

Flag leaves of WT and LMM *5386* lines were collected, and three biological replicates were used for RNA-seq. GO and KEGG analyses were performed to identify DEGs enriched in GO terms and metabolic pathways, respectively. A corrected p-value (≤ 0.05) was set as the threshold for significantly enriched GO terms and KEGG pathways.

### Determination of H_2_O_2_ content and ${\text{O}}_{2}^{•-}$ production rate

H_2_O_2_ content and ${\text{O}}_{2}^{•-}$ production rates were measured according to previously reported methods (Hao et al., 2018). Accumulation of ${\text{O}}_{2}^{•-}$ and H_2_O_2_ in flag leaves was visually evaluated by staining with NBT (0.5 mg mL^−1^, pH 7.6) for ${\text{O}}_{2}^{•-}$ and DAB (1 mg mL^−1^, pH 3.8) for H_2_O_2_.

### Determination of glutathione and glycine content

The GSH and Gly content was measured according to previously reported methods (Noctor et al., 1997).

### Measurement of antioxidant enzyme activity

The activities of superoxide dismutase (SOD), catalase (CAT), ascorbate peroxidase (APX), peroxidase (POD), glutathione reductase (GR), and glutathione-S-transferases (GST) were detected based on previously described methods: SOD (Wang et al., 2020), CAT (Yang et al., 2020), APX (Wang et al., 2017), POD (Wang et al., 2016b), GR (Yin et al., 2017), and GST (Li et al., 2018). All samples were analyzed using a Shimadzu UV-1900i spectrophotometer.

### Quantitative reverse transcription PCR analysis

Total RNA was isolated from leaves using TRIzol Reagent (Invitrogen, Carlsbad, CA, USA). The RNA was used to produce cDNA using a reverse transcription kit (Vazyme, Nanjing, China). Quantitative reverse transcription PCR was performed using the ChamQ Universal SYBR qPCR Master Mix Kit (Vazyme, China). The expression of a specific gene versus a control was determined using the formula 2^−△△CT^. Actin was evaluated as the control gene (Hao et al., 2018). Information on the genes analyzed is presented in **Supplementary Table 3**.

### *Fusarium graminearum* test

*F. graminearum* is an engineered strain expressing the AmCyan fluorescent protein. The resistance to *F. graminearum* was tested according to previously reported methods (Hao et al., 2018). The lesion length was measured at 3DAI and 7 DAI using the ImageJ program.

### Powdery mildew bioassay

Leaf tissues were infiltrated with a solution of spores of *B. graminis* f. sp. *hordei* (Lumbroso et al., 1982). Leaf tissues were then boiled for 10 min in a solution containing glycerol, lactic acid, phenol, and distilled water (1: 1: 1: 1, v/v/v/v) with 0.5 mg/mL trypan blue, and maintained at room temperature for 6–8 h. Leaf tissues were then clarified overnight in 2.5 mg/mL chloral hydrate. Leaf samples were examined using an Olympus BX50 light microscope (Olympus, Tokyo, Japan).

### Statistical analysis

All analyses were performed at least in triplicate. The IBM SPSS Statistics program was used to perform the statistical analyses. All comparisons were analyzed using factorial ANOVA. Differences between the means among the lines were compared using Duncan’s multiple range tests at 0.05 probability levels.

## Results

### Phenotype analysis of WT and LMM *5386* lines

**Figure 1:**
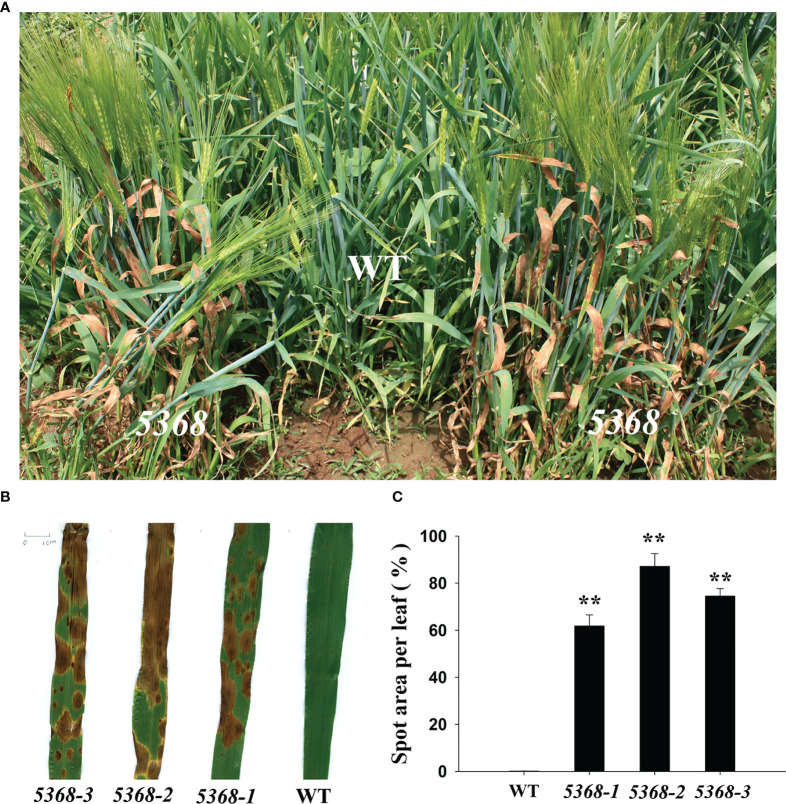
Phenotype differences between WT and LMM *5386* lines in the field. **(A)** Phenotype in the field; **(B)** leaf brown spots; **(C)** spot area per leaf. Values are means ± SD based on 30 replicates. Error bars indicate standard deviations. **P < 0.01.

We found that several brown spots were spontaneously produced in the leaves of LMM *5386* lines under field conditions (**Figure 1A**). Three independent lines (*5386-1*, *5386-2*, and *5386-3*) of LMM *5386* mutants were selected for phenotype identification. The brown spot area per leaf was observed and quantified in WT and LMM *5386* lines, with that of the LMM *5386* line plants being significantly higher than of the WT plants (**Figures 1B, C**).

### The barley *LM 5386* gene is localized on the chromosome 3H

For genetic analysis of LM genes in the LMM *5386* lines, we formed the F2 population with Morex (whole genome sequencing, WGS), including 343 individual lines, and we randomly analyzed the phenotype data of this F2 population. There were 89 LM lines and 254 non-LM lines, meeting the 1:3 separation ratio by Chi-square test (χ^2^ = 0.25, P = 0.62 > 0.05) (**Table 1**). Overall, *LM 5386* behaved as a single recessive gene.

**Table 1:** The genetic analysis of lesion mimic 5386/Morex F2 population.

Generation	Sum	Phenotypic trait	Actual value	Theoretical value	Separation ratio	χ^2^ value	P value
F_2_	343	lesion mimic	89	85	1:2.9	0.25	0.62
		No lesion mimic	254	258			

Using the BSR-Seq data, 865 high-confidence SNPs and 72 InDels were revealed between the F2 recessive homozygous RNA pool and Morex RNA. Among the 937 co-segregating polymorphisms, 589 (62.8%) were localized on the chromosome 3H (**Supplementary Figure 1**).

### RNA-seq analysis of WT and LMM *5386* lines

To better understand the mechanism behind LM formation in the LMM lines, we performed RNA-seq analyses using flagged leaves of the WT and LMM *5386* lines. These analyses provided 65.80 Gb of clean bases, and the percentage of Q30 in each sample was not less than 93.89%, with 91.62–92.09% of the reads being accurately mapped to the reference genome and 2.38–3.69% of the reads being mapped to multiple genome sequences (**Supplementary Table 1**). The Pearson correlation coefficients among biological replicates were higher than 0.95 (**Supplementary Figure 2**).

**Figure 2:**
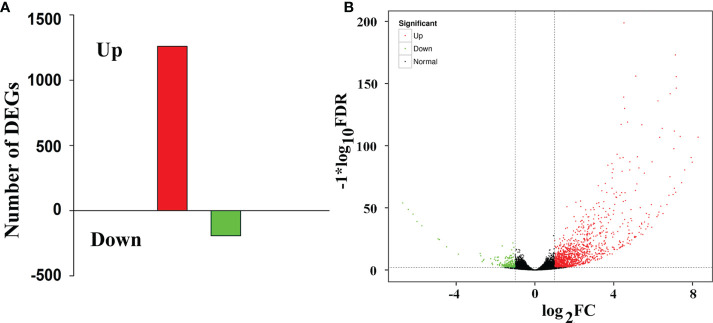
RNA-Seq analyses of WT and LMM *5386*. **(A)** Changes in the gene expression profiles in WT- and LMM *5386*-line flag leaves; **(B)** differentially expressed genes (DEGs). Values are means ± SD based on three replicates.

Compared with that in the WT lines, 1453 differentially expressed genes (DEGs) were found in the LMM *5386* lines, of which 1260 were upregulated and 193 were downregulated (**Figure 2**, **Supplementary Table 2**). DEGs with the same or similar expression patterns were placed into groups based on hierarchical clustering analysis. The nine largest DEGs (Group 1) were enriched in the GSH metabolic process, GSH transferase activity, anchored component of the plasma membrane, aleurone grain membrane, and cytokinin biosynthetic process (**Supplementary Figure 3**).

**Figure 3:**
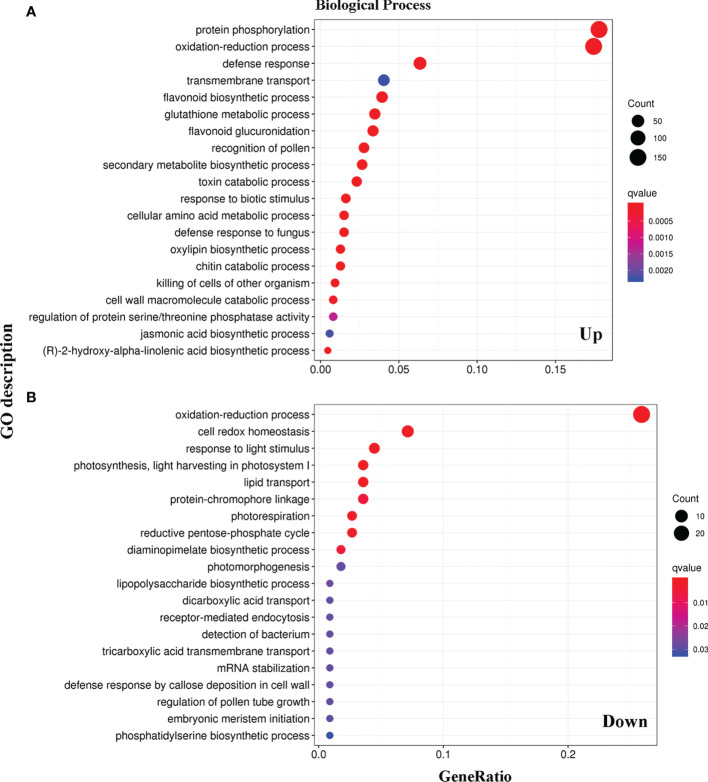
Categories and distribution of Gene Ontology (GO) terms in WT and LMM *5386* lines. **(A)** upregulated differentially expressed genes (DEGs); **(B)** downregulated differentially expressed genes (DEGs).

The functions of the 1453 DEGs were verified using the gene ontology (GO) database (http://www.geneontology.org), providing annotations of the biological processes, molecular functions, and cellular components. Specifically, we compared the biological process between the WT and LMM *5386* lines. Among the upregulated DEGs, those encoding the protein phosphorylation, oxidation-reduction, defense response, transmembrane transport, flavonoid biosynthetic, GSH metabolic, and flavonoid glucuronidation were notably over-represented (**Figure 3A**, **Supplementary Table 2**). Among the downregulated DEGs, many were significantly enriched in oxidation-reduction, cell redox homeostasis, light stimulus, light harvesting in photosystem I, lipid transport, and protein−chromophore linkage (**Figure 3B**, **Supplementary Table 2**). In both upregulated and downregulated DEGs, a substantial number of genes were enriched in oxidation-reduction (**Figure 3**).

**Figure 4:**
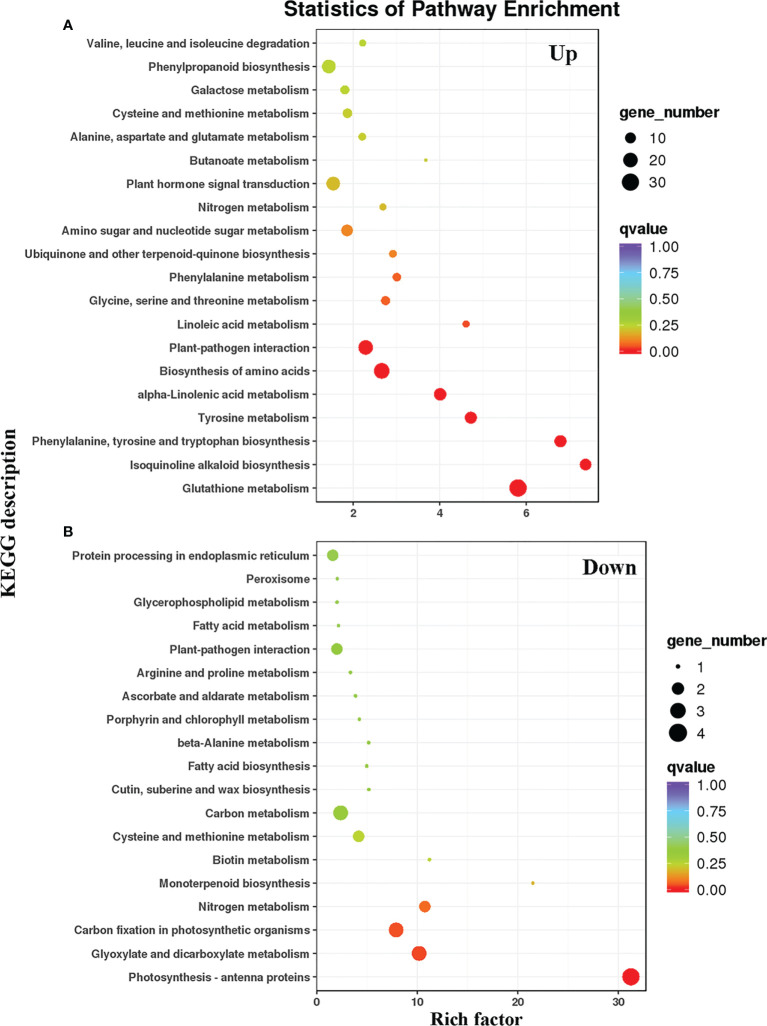
KEGG pathways of prominently enriched differentially expressed genes (DEGs) in WT and LMM *5386* lines. **(A)** upregulated differentially expressed genes (DEGs); **(B)** downregulated differentially expressed genes (DEGs).

To compare the DEG metabolic pathways in the WT and LMM *5386* lines, we used the Kyoto Encyclopedia of Genes and Genomes (KEGG) database (https://www.genome.jp/kegg/pathway.html). Among the upregulated DEGs, many genes were significantly enriched in GSH metabolism, isoquinoline alkaloid biosynthesis, phenylalanine, tyrosine biosynthesis, tryptophan biosynthesis, tyrosine metabolism, alpha−linolenic acid metabolism, amino acid biosynthesis, and plant−pathogen interactions (**Figure 4A**, **Supplementary Table 2**). Among the downregulated DEGs, many genes were significantly enriched in photosynthesis antenna proteins, glyoxylate metabolism, dicarboxylate metabolism, carbon fixation in photosynthetic organisms, and nitrogen metabolism (**Figure 4B**, **Supplementary Table 2**). Lastly, in both upregulated and downregulated DEGs, a considerable number of genes were enriched in GSH and nitrogen metabolism (**Figure 4**).

### ROS analysis of WT and LMM *5386* lines

**Figure 5:**
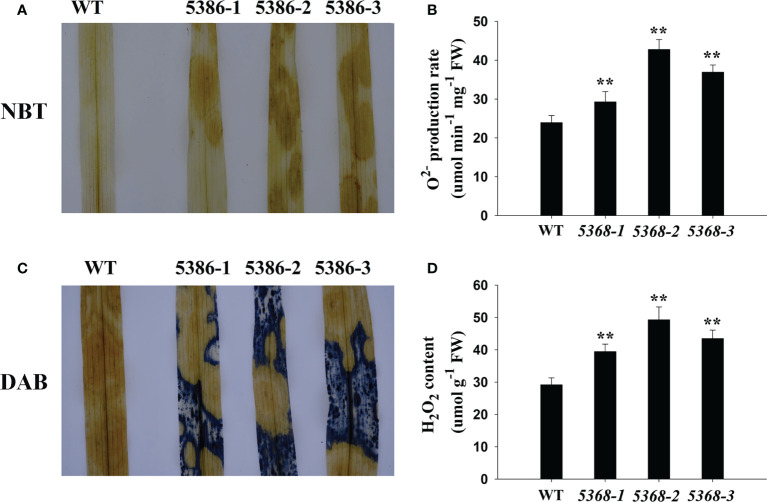
Changes in reactive oxygen species (ROS) accumulation in flag leaves in WT and LMM *5386* lines. **(A)** NBT staining results for ${\text{O}}_{2}^{•-}$ ; **(B)** ${\text{O}}_{2}^{•-}$ production rate; **(C)** DAB staining for H_2_O_2_; **(D)** H_2_O_2_ content. Values are means ± SD of three replicates. Error bars indicate standard deviations. **P < 0.01.

GO analysis showed that many DEGs were enriched in the oxidation-reduction process (**Figure 3**). Therefore, we compared the H_2_O_2_ contents and ${\text{O}}_{2}^{•-}$ production rates between the WT and LMM *5386* lines, which were determined by diaminobenzidine (DAB) and nitroblue tetrazolium (NBT) staining, respectively. The LMM *5386* line plants was heavier in color when stained with NBT (**Figure 5A**) and DAB (**Figure 5C**) than the WT plants. This indicated that there were higher concentrations of ${\text{O}}_{2}^{•-}$ and H_2_O_2_ in LMM *5386* lines. We further confirmed this pattern by quantifying the ${\text{O}}_{2}^{•-}$ production rates and H_2_O_2_ contents. Similarly, LMM *5386* line plants showed a relatively higher ${\text{O}}_{2}^{•-}$ production rate and H_2_O_2_ content than the WT plants (**Figures 5B, D**).

### GSH and Gly analysis of WT and LMM *5386* lines

**Figure 6:**
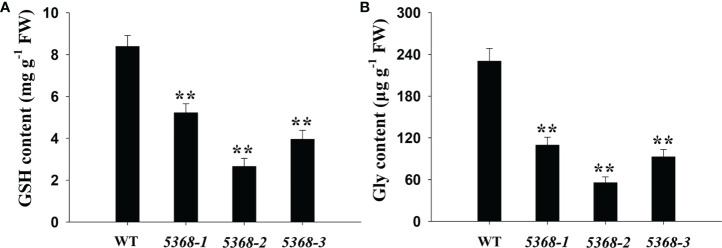
Changes in **(A)** GSH content and **(B)** Gly content in flag leaves between WT and LMM *5386* lines. Values are mean ± SD based on three replicates. Error bars indicate standard deviations. **P < 0.01.

KEGG analyses showed that many DEGs were significantly enriched in GSH and nitrogen metabolism (**Figure 4**); therefore, we compared the GSH and Gly contents between the WT and LMM *5386* line plants. Both GSH and Gly contents were significantly lower in LMM *5386* line plants than in the WT plants (**Figure 6**).

### Antioxidant competence analysis of WT and LMM *5386* lines

**Figure 7:**
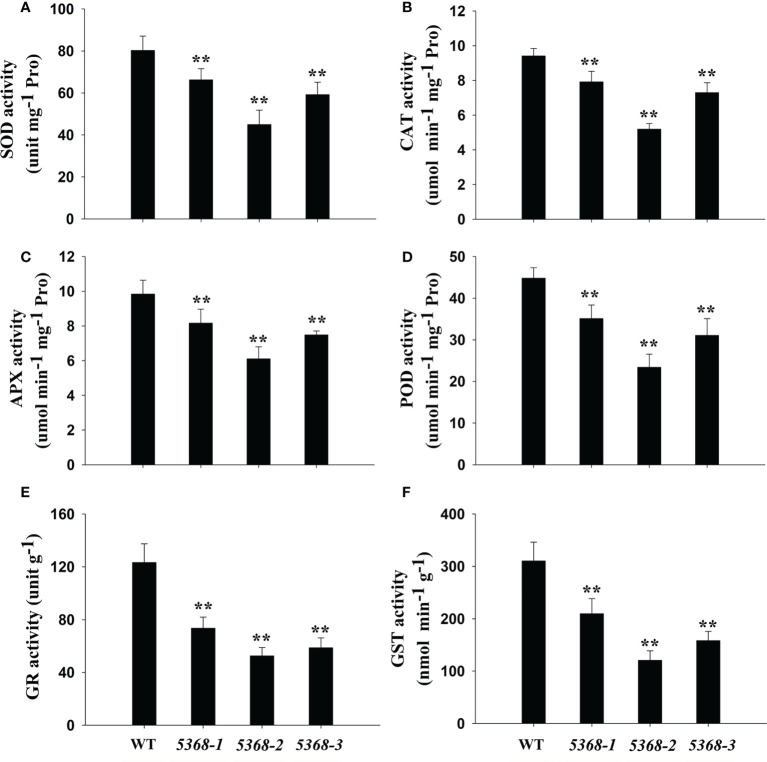
Changes in antioxidant enzyme activity in flag leaves between WT and LMM *5386* lines. **(A)** Superoxide dismutase (SOD), **(B)** catalase (CAT), **(C)** ascorbate peroxidase (APX), **(D)** peroxidase (POD), **(E)** glutathione reductase (GR), and **(F)** glutathione-S-transferases (GST) activity. Values are means ± SD of three replicates. Error bars indicate standard deviations. **P < 0.01.

Antioxidant enzyme activities were also compared between WT and LMM *5386* lines. We measured SOD (**Figure 7A**), CAT (**Figure 7B**), APX (**Figure 7C**), POD) (**Figure 7D**, GR (**Figure 7E**), and GST (**Figure 7F**) activity levels, which were significantly lower in the LMM *5386* line plants than in the WT plants. Nevertheless, the downregulation of GR and GST was significantly greater than that of SOD, CAT, APX, and POD in LMM *5386* lines.

**Figure 8:**
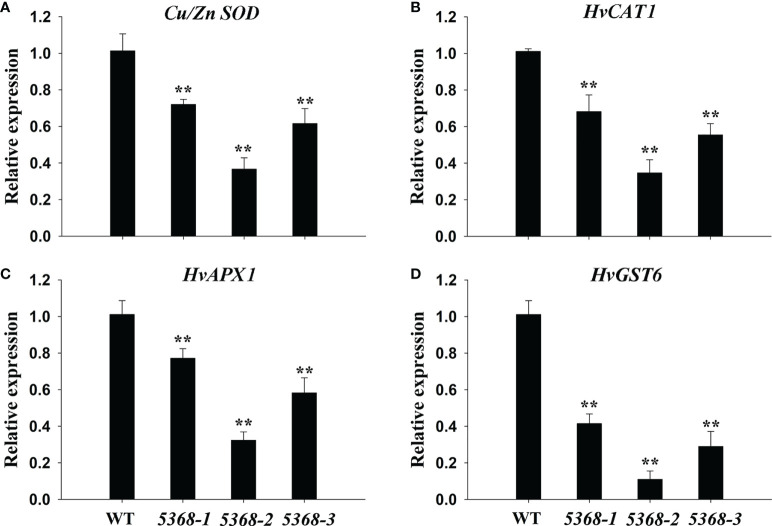
Relative expression of antioxidant enzyme genes in flag leaves of WT and LMM *5386* lines. **(A)** *C_U_/Z_N_ SOD*, **(B)** *H_V_CAT1*, **(C)** *H_V_APX1*, and **(D)** *H_V_GST6*. Values are means ± SD based on six replicates. Error bars indicate standard deviations. **P < 0.01.

ROS-scavenging related genes were detected between the WT and LMM *5386* lines. *Cu/Zn-SOD* encodes a chloroplastic copper/zinc superoxide dismutase and *CAT* encodes a catalase (Wang et al., 2016b). Those genes included *Cu/Zn-SOD* (**Figure 8A**), *HvCAT1* (**Figure 8B**), *HvAPX1* (**Figure 8C**), and *HvGST6* (**Figure 8D**). As shown in **Figure 8**, transcription levels of the ROS-scavenging genes were significantly lower in the LMM *5386* lines than those in the WT lines.

### Resistance to *Fusarium graminearum* analysis of WT and LMM *5386* lines

**Figure 9:**
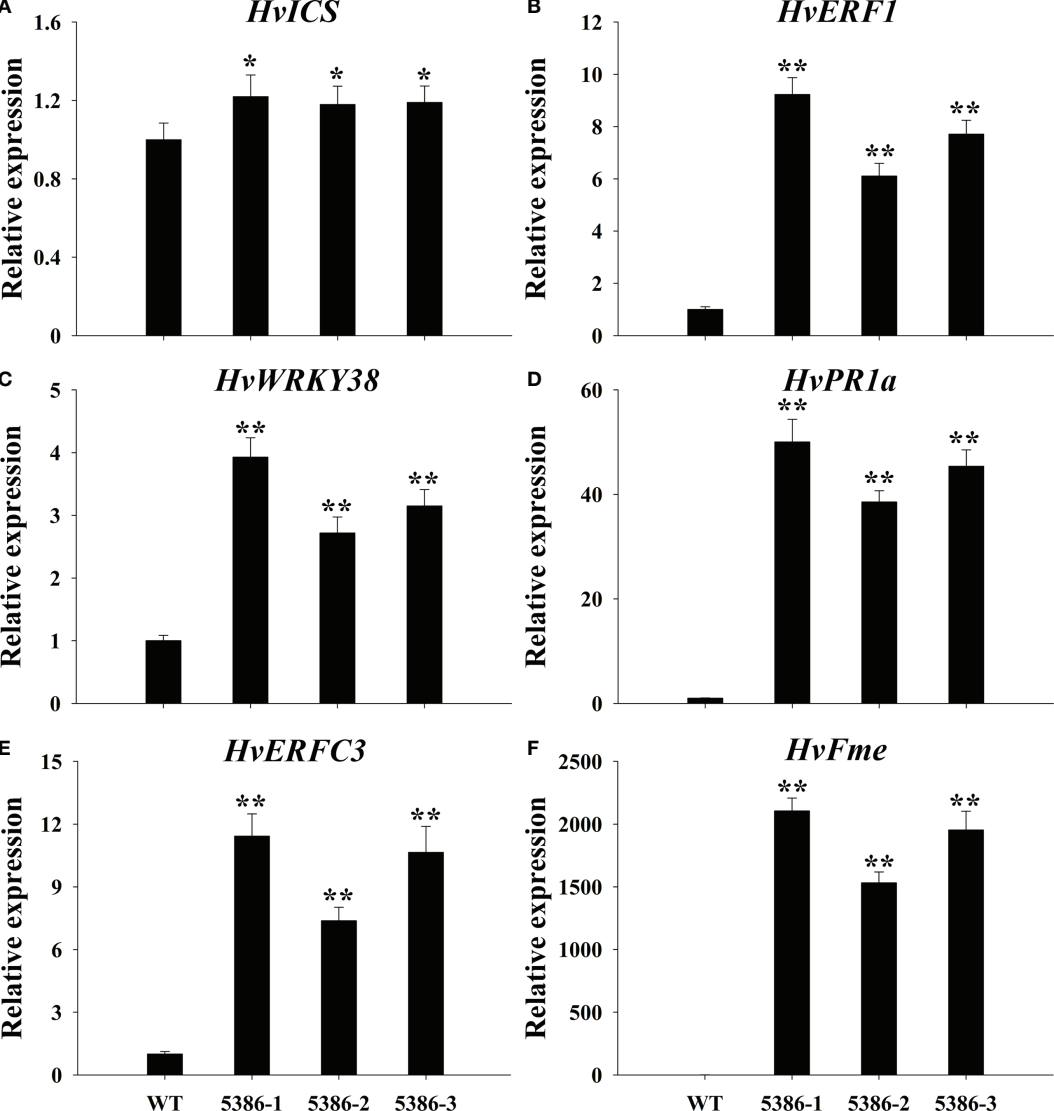
Relative expression of disease resistance-related genes in flag leaves of WT and LMM *5386* lines. **(A)** *HvICS*, **(B)** *HvERF1*, **(C)** *HvWRKY38*, **(D)** *HvPR1a*, **(E)** *HvERFC3*, **(F)** *HvFme*. Values are means ± SD based on six replicates. Error bars indicate standard deviations. *P < 0.05; **P < 0.01.

**Figure 10:**
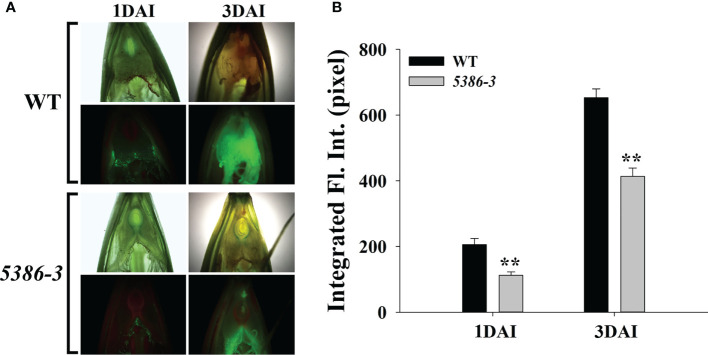
Phenotype differences between inoculated *(F) graminearum* in spikes between WT and LMM *5386* lines. **(A)** Green fluorescence phenotype 1 day after inoculation (DAI) and 3 DAI. **(B)** Integrated fluorescence intensity (IFI) at 1 DAI and 3 DAI. Values are means ± SD based on thirty replicates. Error bars indicate standard deviations. **P < 0.01.

RNA-seq analyses indicated that a considerable number of disease-resistance-associated genes were altered between the WT and LMM *5386* lines. Therefore, we determined the expression of six disease-resistance-related genes, that is, isochorismate synthase (*HvICS*) (**Figure 9A**), ethylene response factor 1 (*HvERF1*) (**Figure 9B**), *HvWRKY38* (**Figure 9C**), pathogenesis related protein-1a (*HvPR1a*) (**Figure 9D**), ethylene-responsive transcription factor 3 (*HvERFC3*) (**Figure 9E**), and flavonoid O-methyltransferase protein (*HvFme*) (**Figure 9F**). The expression levels of these disease-resistance-related genes in the LMM *5386* lines were significantly higher than those in the WT plants (**Figure 9**). We estimated *F. graminearum* growth by observation and quantification of the integrated fluorescence intensity (IFI) of the infected florets; LMM *5386* lines were associated with the lower IFI reading at 1 and 3 days after inoculation (DAI) (**Figure 10**). We further estimated the *F. graminearum* resistance by statistical IFI analyses of the infected leaves. Again, the IFI readings of the LMM *5386* line plants were significantly lower than those of the WT plants (**Supplementary Figure 4**). We also tested the LMM *5386* lines for their disease resistance responses to *Blumeria graminis* f. sp. *hordei*, which is a biotrophic pathogen. At 5 DAI, the LMM *5386* lines showed shorter mycelia relative to those of the WT (**Supplementary Figure 5**).

## Discussion

### Brown spot phenotype of LMM *5386* lines

Barley (*Hordeum vulgare*, 2n = 14), the fourth largest cereal crop in the world, offers high yields and good stress tolerance (Hao et al., 2019) and presents diverse morphological and genetic features, making it a model species for the *Triticeae* tribe. LMs are common in plant mutation populations, and LMMs with abundant phenotype have been reported in rice and *Arabidopsis thaliana* (Meskauskiene et al., 2011; Harkenrider et al., 2016), such as small red, reddish-brown, tiny black, and yellow leaf spots (Hao et al., 2019). We identified the novel LMM *5386* in barley, which spontaneously produced many large and severe brown spots at the four-leaf stage and then spread throughout the plant in the whole growth stage (**Figure 1**). LMMs have been classified as “initiation” and “propagation” types (Landoni et al., 2013), and we categorized LMM *5386* as a “propagation” type.

For the highly repetitive sequences in wheat and barley, BSR-Seq provides an effective approach for monogenic mapping (Liu et al., 2012; Chayut et al., 2015). In this study, we demonstrated that the BSR-Seq identified the LM *5386* gene at the chromosome 3H in barley (**Supplementary Figure 1**).

### LM caused by ROS accumulation in LMM *5386* lines

RNA-seq is a useful approach for analyzing DEGs at the transcriptome level and clarifying their regulatory network, thus providing insight into the mechanism of LM formation. Transcriptomic analysis showed that DEG responses were mediated through various pathways (Li et al., 2017). RNA-seq was used to analyze the 1453 DEGs involved in the formation of LM in LMM *5386* lines (**Figure 2**, **Supplementary Table 2**). GO analysis revealed that many DEGs were enriched in the oxidation-reduction process (**Figure 3**), suggesting that this process is crucial to LM formation.

PCD can be classified as either autolytic or non-autolytic (van Doorn, 2011). Autolytic PCD primarily occurs during plant growth and includes LM formation, whereas non-autolytic PCD primarily occurs when plants are subjected to external stress (van Doorn, 2011). ROSs, such as H_2_O_2_ and ${\text{O}}_{2}^{•-}$ , are the primary participants in the formation and regulation of the HR, which is the most definitive characteristic of PCD (Coll et al., 2011). Wang et al. (2016b) demonstrated that antioxidant enzymes can remove excess ROS to maintain better plant growth. In this study, LMM *5386* line plants were associated with lower antioxidant enzyme-encoding genes expression levels, lower antioxidant enzyme activity, and higher levels of ROS than the WT plants (**Figures 5**, **7**, and **8**). These results suggested that decreased antioxidant competence leads to ROS accumulation and subsequent PCD in LMM *5386* lines.

In rice, many signaling pathways and biological processes are involved in LM formation, including protein phosphorylation (Harkenrider et al., 2016), abscisic acid signaling (Wang et al., 2015b), and protein ubiquitination (Liu et al., 2015). KEGG analyses showed that many upregulated DEGs were significantly enriched in GSH metabolism (**Figure 4A**). H_2_O_2_ removal is predominantly achieved by ascorbate/GSH cycles in higher plants, and GSH is an intermediate recirculation product (Noctor and Foyer, 1998). GSH is a special class of amino acid derivative consisting of glutamate, cysteine, and Gly (Kaya, 2020). Gly produced by photorespiration is crucial to GSH synthesis (Noctor et al., 1997). LMM *5386* lines were associated with lower Gly content, resulting in the inhibition of GSH biosynthesis (**Figure 6**). These results suggested that decreased GSH biosynthesis is also important to this burst in ROS and subsequent LM formation.

### LMM *5386* lines confer basal resistance to *Fusarium graminearum*

Currently, more than 30 LMMs have been identified exhibiting blast disease resistance (Zhu et al., 2016). Salicylic acid (SA), jasmonic (JA), and ethylene (ET) are important to the disease resistance of LMMs (Lorenzo et al., 2003; Vlot et al., 2009; Hao et al., 2019). SA protects plants from biotrophic pathogens, whereas JA and ET protect plants from necrotrophic pathogens (Glazebrook, 2005). *F. graminearum* is hemibiotrophic, that is, it is initially biotrophic but becomes necrotrophic during pathogenesis when cell death is induced (Kazan et al., 2012). The expression of disease-resistance-related genes in SA, JA, and ET pathways were significantly increased in the LMM *5386* line plants compared to that in the WT plants (**Figure 9**). These results suggested that the LMM *5386* lines had some disease resistance. Using floret- and leave-based inoculated *F. graminearum* tests, the IFI was lower in the LMM *5386* line plants than that in the WT plants (**Figure 10**, **Supplementary Figure 4**). This further confirmed that the LMM *5386* lines inhibited the growth of *Blumeria graminis* f. sp. *hordei* (**Supplementary Figure 5**). Therefore, LMM *5386* lines conferred basal resistance to some biotrophic pathogens or to some pathogens at the biotrophic stages of their life cycle.

## Conclusion

The barley LMM *5386* plants conferred basal resistance to *F. graminearum*; however, its decreased antioxidant competence and GSH contents caused ROS accumulation and subsequent PCD, eventually reducing its yield components (**Table 2**).

**Table 2:** Yield components of WT and LMM *5386* lines.

Cultivar	1000-kernel weight (g)	Spike number	Grain length (mm)	Grain width (mm)
WT	39.5±1.06a	65.0±3.98a	7.03±0.51a	3.07±0.27a
LMM *5386*	37.9±1.13b	64.8±2.45a	6.85±0.47a	2.97±0.22a

## Data availability statement

The datasets presented in this study can be found in online repositories. The names of the repository/repositories and accession number(s) can be found below: SRA database, accession numbers SRR21511341-SRR21511346.

## Author contributions

The work presented here was carried out in collaboration among all authors. WeiW and WenW defined the research theme. QH and JZ designed most of the methods and experiments. QH and JZ carried out the laboratory experiments. QH and WenW wrote the paper. All authors contributed to the article and approved the submitted version.

## Funding

This work was supported by the Natural Science Foundation of Shandong (ZR2020QC113), China Postdoctoral Science Foundation (2022M711980), the National Natural Science Foundation of China (32001536), the Science and Technology Plan Projects of Zaozhuang (2019NS01), the Doctoral Research Initiation Funds of Zaozhuang University (2018BS043, 2020BS001), the Opening Foundation of State Key Laboratory of Crop Biology (2020KF06).

## Conflict of interest

Authors JZ and DF were employed by Spring Valley Agriscience Co., Ltd. Author WenW was employed by Shandong Shofine Seed Technology Co., Ltd.

The remaining authors declare that the research was conducted in the absence of any commercial or financial relationships that could be construed as a potential conflict of interest.

## Publisher’s note

All claims expressed in this article are solely those of the authors and do not necessarily represent those of their affiliated organizations, or those of the publisher, the editors and the reviewers. Any product that may be evaluated in this article, or claim that may be made by its manufacturer, is not guaranteed or endorsed by the publisher.

## Supplementary material

Supplementary Figure 1 BSR-Seq analysis SNPs and InDels of lesion mimic 5386/Morex F2 population.

Supplementary Figure 2 Pearson correlation coefficients among three biological replicates of WT and LMM *5386* lines.

Supplementary Figure 3 Hierarchical clustering analysis of nine differentially expressed genes (DEGs) based on the log (FC) of gene expression in Group 1. The color gradient from red to green represents relative levels of gene expression (from low to high, respectively). The numbers in the scale bar indicate the gene expression scores.

Supplementary Figure 4 Phenotype differences on inoculated *F. graminearum* in leaves between WT and LMM *5386* lines. **(A)** Green fluorescence phenotype at 3 days after inoculation (DAI) and 7 DAI. **(B)** Integrated fluorescence intensity (IFI) at 3 DAI and 7 DAI. Values are means ± SD based on thirty replicates. Error bars indicate standard deviations. *P < 0.05; **P < 0.01

Supplementary Figure 5 Phenotype differences on inoculated powdery mildew in leaves between WT and LMM *5386* lines. **(A)** Trypan blue staining of developing hyphae of *Blumeria graminis* f. sp. *Hordei*. **(B)** Length of fungal hyphae in infected plants. Values are means ± SD based on thirty replicates. Error bars indicate standard deviations. *P < 0.05; **P < 0.01
