# Supplementary figures and images for: Role of reactive oxygen species in lesion mimic formation and conferred basal resistance to Fusarium graminearum in barley lesion mimic mutant 5386
Source: Front Plant Sci. 2022 Oct 31;13:1020551. doi: 10.3389/fpls.2022.1020551 (PMC9869871; doi:10.3389/fpls.2022.1020551)

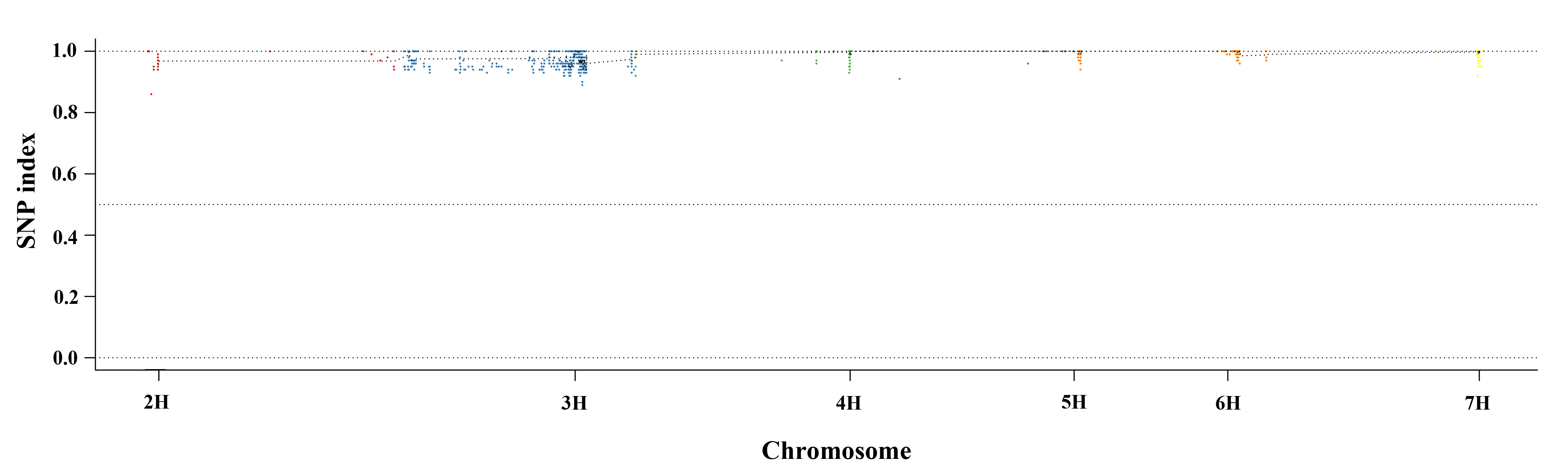

Supplement: Supplementary Figure 1 — BSR-Seq analysis SNPs and InDels of lesion mimic 5386/Morex F2 population. [file Image_1.jpeg]

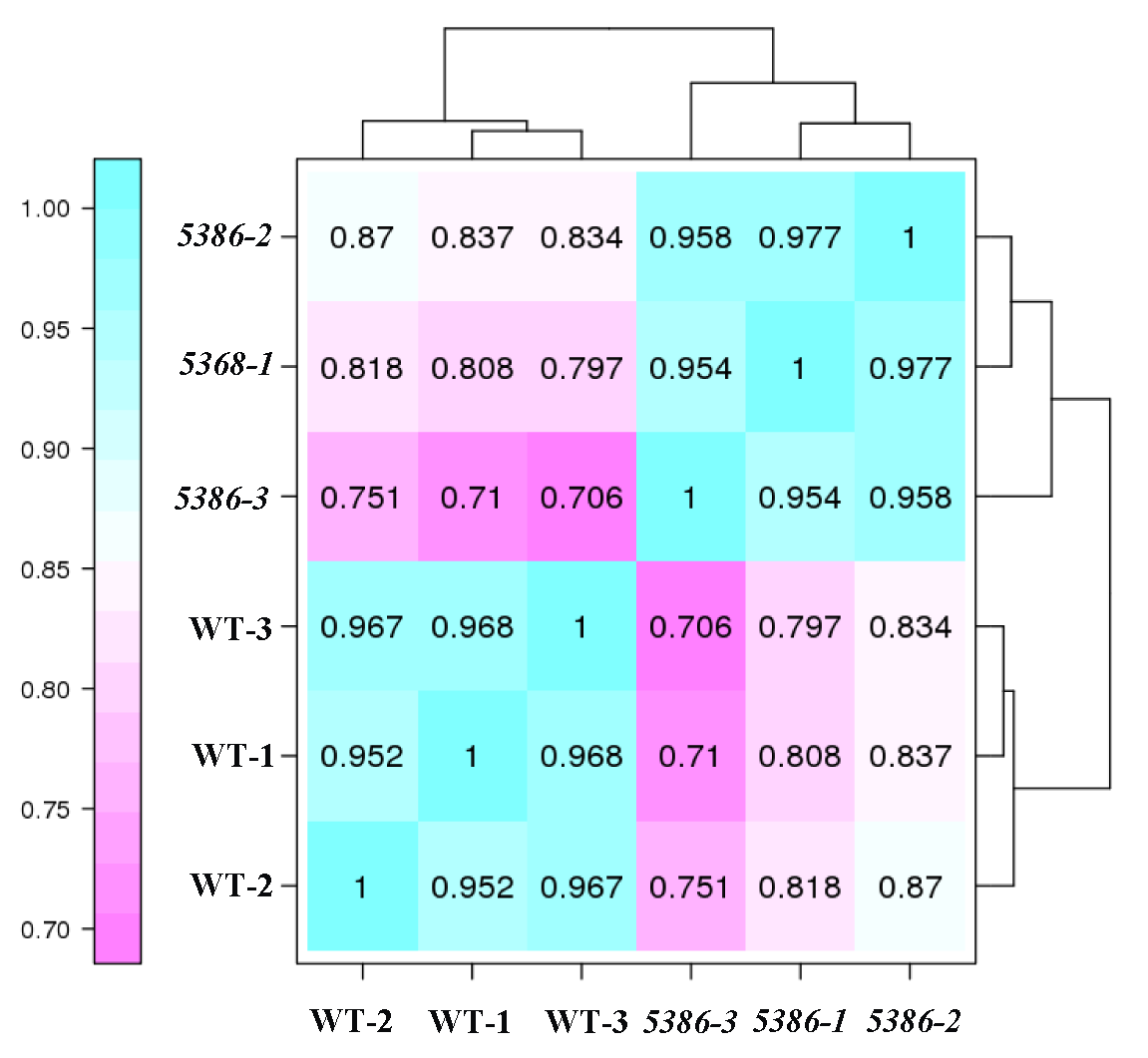

Supplement: Supplementary Figure 2 — Pearson correlation coefficients among three biological replicates of WT and LMM 5386 lines. [file Image_2.tif]

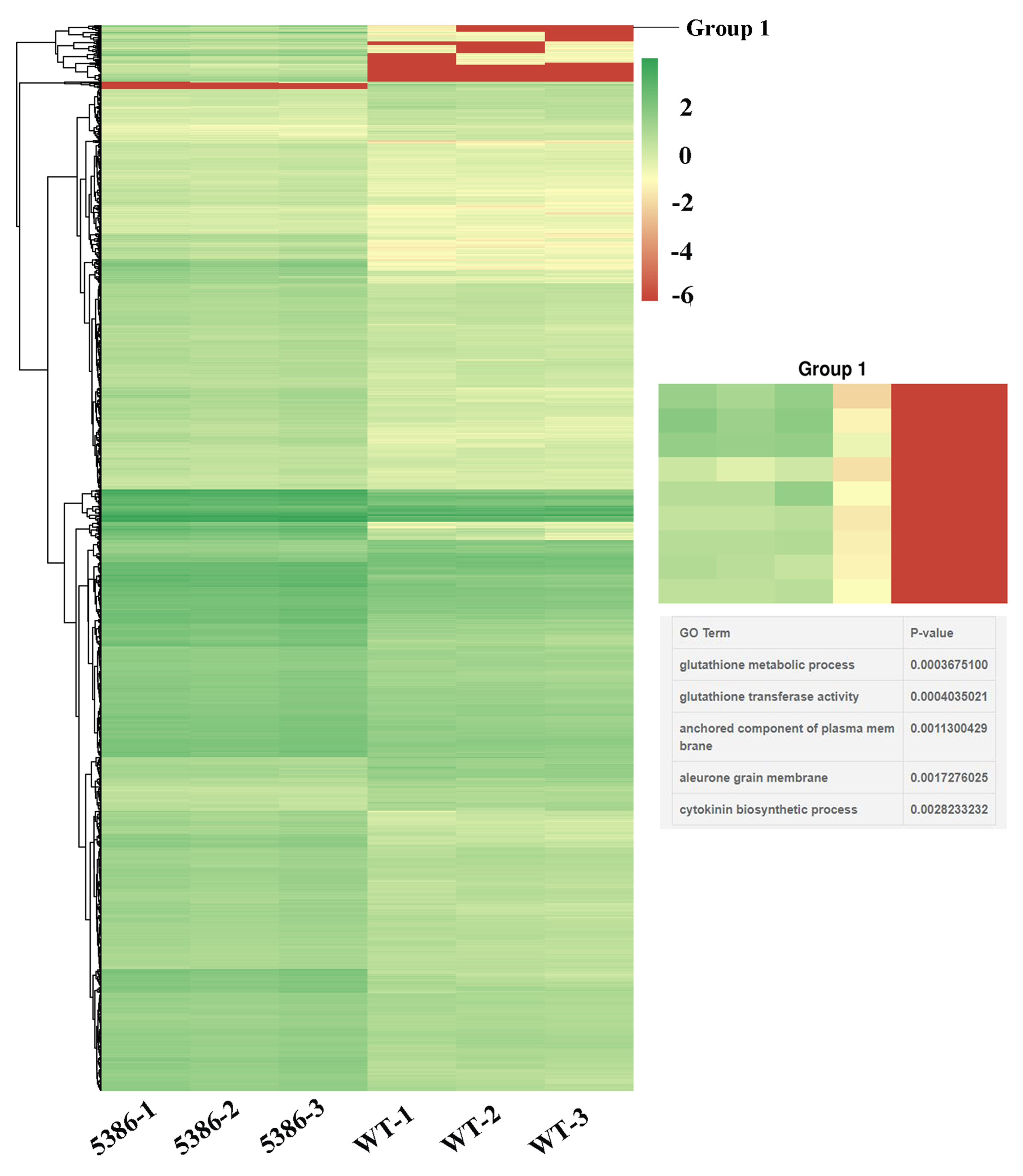

Supplement: Supplementary Figure 3 — Hierarchical clustering analysis of nine differentially expressed genes (DEGs) based on the log (FC) of gene expression in Group 1. The color gradient from red to green represents relative levels of gene expression (from low to high, respectively). The numbers in the scale bar indicate the gene expression scores. [file Image_3.tif]

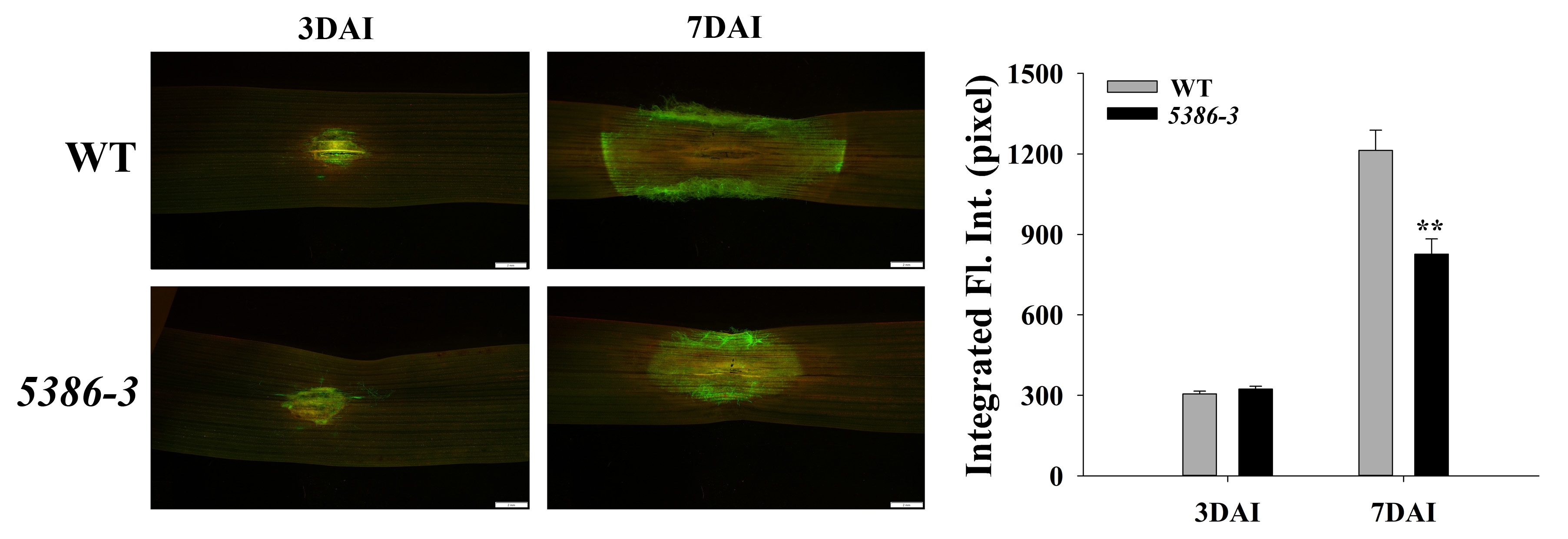

Supplement: Supplementary Figure 4 — Phenotype differences on inoculated F. graminearum in leaves between WT and LMM 5386 lines. (A) Green fluorescence phenotype at 3 days after inoculation (DAI) and 7 DAI. (B) Integrated fluorescence intensity (IFI) at 3 DAI and 7 DAI. Values are means ± SD based on thirty replicates. Error bars indicate standard deviations. *P < 0.05; **P < 0.01 [file Image_4.jpeg]

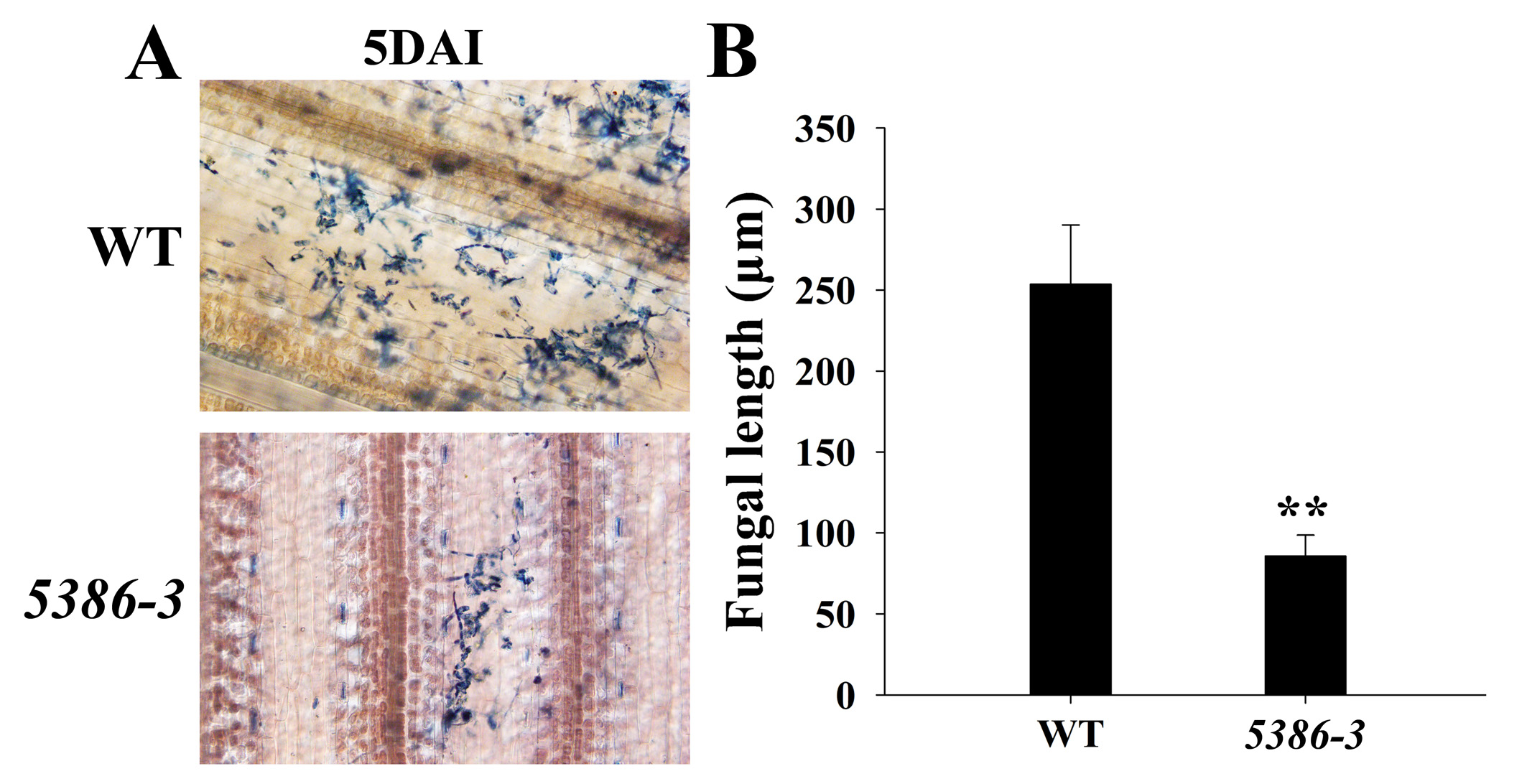

Supplement: Supplementary Figure 5 — Phenotype differences on inoculated powdery mildew in leaves between WT and LMM 5386 lines. (A) Trypan blue staining of developing hyphae of Blumeria graminis f. sp. Hordei. (B) Length of fungal hyphae in infected plants. Values are means ± SD based on thirty replicates. Error bars indicate standard deviations. *P < 0.05; **P < 0.01 [file Image_5.jpeg]
