# Supplementary material for: Role of reactive oxygen species in lesion mimic formation and conferred basal resistance to Fusarium graminearum in barley lesion mimic mutant 5386
Source: Front Plant Sci. 2022 Oct 31;13:1020551. doi: 10.3389/fpls.2022.1020551 (PMC9869871; doi:10.3389/fpls.2022.1020551)
Supplement: Supplementary file 6 [file DataSheet_1.docx]

**Supplementary Table 1. Summary of the sequence data from RNA sequencing.**

| Samples | Clean reads | Clean bases | GC Content | %≥Q30 | Mapped Reads | Multiple Map Reads |
| --- | --- | --- | --- | --- | --- | --- |
| *5368-1* | 37,047,360 | 10,979,042,458 | 57.65% | 94.05% | 68,044,432 (91.83%) | 1,920,373 (2.59%) |
| *5386-2* | 37,877,134 | 11,262,123,100 | 57.30% | 93.89% | 69,430,643 (91.65%) | 2,093,998 (2.76%) |
| *5386-3* | 35,801,059 | 10,557,653,852 | 57.24% | 93.93% | 65,598,544 (91.62%) | 2,160,694 (3.02%) |
| WT-1 | 37,799,868 | 11,181,592,898 | 56.97% | 94.08% | 69,620,925 (92.09%) | 2,833,217 (3.75%) |
| WT-2 | 37,517,418 | 11,105,141,892 | 57.06% | 94.04% | 69,011,360 (91.97%) | 2,040,514 (2.72%) |
| WT-3 | 36,100,179 | 10,713,673,214 | 57.14% | 93.98% | 66,411,477 (91.98%) | 2,642,342 (3.66%) |

| **Supplementary** **Table 2. Differentially Expressed Genes in RNA-Seq** | | | | | | | | | | | | | | | |
| --- | --- | --- | --- | --- | --- | --- | --- | --- | --- | --- | --- | --- | --- | --- | --- |
| ID | WT-1_Count | WT-2_Count | WT-3_Count | 5386-1_Count | 5386-2_Count | 5386-3_Count | WT-1_FPKM | WT-2_FPKM | WT-3_FPKM | 5386-1_FPKM | 5386-2_FPKM | 5386-3_FPKM | FDR | log2FC | regulated |
| HORVU.MOREX.r2.6HG0502750 | 11.00 | 57.00 | 17.00 | 512.00 | 251.00 | 418.00 | 0.10 | 0.56 | 0.17 | 5.05 | 2.41 | 4.34 | 0.00 | 3.01 | up |
| HORVU.MOREX.r2.3HG0232890 | 119.00 | 98.00 | 76.00 | 240.00 | 306.00 | 342.00 | 1.51 | 1.23 | 1.00 | 3.02 | 3.76 | 4.53 | 0.00 | 1.46 | up |
| HORVU.MOREX.r2.3HG0265690 | 7.00 | 3.00 | 29.00 | 89.00 | 57.00 | 91.00 | 0.09 | 0.04 | 0.38 | 1.11 | 0.69 | 1.19 | 0.00 | 1.66 | up |
| HORVU.MOREX.r2.5HG0436740 | 76.00 | 47.00 | 53.00 | 179.00 | 136.00 | 134.00 | 1.04 | 0.64 | 0.76 | 2.43 | 1.80 | 1.92 | 0.00 | 1.19 | up |
| HORVU.MOREX.r2.7HG0584640 | 4.00 | 16.00 | 14.00 | 601.00 | 355.00 | 704.00 | 0.04 | 0.15 | 0.14 | 5.83 | 3.35 | 7.19 | 0.00 | 4.73 | up |
| HORVU.MOREX.r2.3HG0257820 | 4034.00 | 3910.00 | 3665.00 | 12009.00 | 12387.00 | 13068.00 | 46.83 | 45.07 | 44.29 | 137.66 | 138.43 | 157.84 | 0.00 | 1.63 | up |
| HORVU.MOREX.r2.5HG0415170 | 0.00 | 0.00 | 0.00 | 2255.00 | 988.00 | 1942.00 | 0.00 | 0.00 | 0.00 | 23.76 | 10.15 | 21.56 | 0.00 | 7.99 | up |
| HORVU.MOREX.r2.1HG0065170 | 2128.00 | 2111.00 | 1834.00 | 4428.00 | 4163.00 | 4158.00 | 14.92 | 14.70 | 13.40 | 30.67 | 28.11 | 30.35 | 0.00 | 1.01 | up |
| HORVU.MOREX.r2.5HG0444510 | 2.00 | 0.00 | 0.00 | 99.00 | 99.00 | 591.00 | 0.09 | 0.00 | 0.00 | 4.46 | 4.35 | 28.15 | 0.00 | 1.73 | up |
| HORVU.MOREX.r2.3HG0205460 | 170.00 | 195.00 | 164.00 | 427.00 | 401.00 | 474.00 | 1.30 | 1.48 | 1.30 | 3.22 | 2.95 | 3.77 | 0.00 | 1.22 | up |
| HORVU.MOREX.r2.2HG0169770 | 4284.00 | 5222.00 | 3551.00 | 36451.00 | 26972.00 | 39292.00 | 40.31 | 48.79 | 34.79 | 338.76 | 244.37 | 384.74 | 0.00 | 2.84 | up |
| HORVU.MOREX.r2.5HG0402030 | 192.00 | 62.00 | 125.00 | 503.00 | 825.00 | 921.00 | 2.80 | 0.89 | 1.90 | 7.27 | 11.62 | 14.03 | 0.00 | 2.19 | up |
| HORVU.MOREX.r2.3HG0203700 | 978.00 | 1211.00 | 606.00 | 3499.00 | 2836.00 | 3641.00 | 15.25 | 18.74 | 9.84 | 53.88 | 42.56 | 59.06 | 0.00 | 1.71 | up |
| HORVU.MOREX.r2.2HG0079040 | 1264.00 | 1131.00 | 899.00 | 3286.00 | 2213.00 | 3363.00 | 22.47 | 19.96 | 16.65 | 57.71 | 37.88 | 62.22 | 0.00 | 1.33 | up |
| HORVU.MOREX.r2.4HG0279610 | 121.00 | 457.00 | 58.00 | 17565.00 | 12033.00 | 23880.00 | 3.11 | 11.71 | 1.54 | 448.35 | 299.43 | 642.25 | 0.00 | 3.18 | up |
| HORVU.MOREX.r2.5HG0392250 | 1358.00 | 1236.00 | 1057.00 | 91337.00 | 69535.00 | 75818.00 | 47.84 | 43.23 | 38.77 | 3179.30 | 2359.67 | 2780.65 | 0.00 | 5.84 | up |
| HORVU.MOREX.r2.3HG0247670 | 272.00 | 244.00 | 189.00 | 582.00 | 481.00 | 651.00 | 2.87 | 2.55 | 2.08 | 6.06 | 4.89 | 7.15 | 0.00 | 1.19 | up |
| HORVU.MOREX.r2.1HG0014200 | 1960.00 | 2254.00 | 1304.00 | 5968.00 | 3799.00 | 4979.00 | 70.35 | 80.31 | 48.73 | 211.55 | 131.30 | 185.98 | 0.00 | 1.32 | up |
| HORVU.MOREX.r2.4HG0314890 | 63.00 | 20.00 | 24.00 | 84.00 | 119.00 | 176.00 | 0.70 | 0.21 | 0.27 | 0.93 | 1.28 | 2.05 | 0.00 | 1.41 | up |
| HORVU.MOREX.r2.3HG0224000 | 273.00 | 100.00 | 81.00 | 941.00 | 680.00 | 707.00 | 3.26 | 1.18 | 1.00 | 11.11 | 7.83 | 8.80 | 0.00 | 1.99 | up |
| HORVU.MOREX.r2.3HG0260290 | 379.00 | 358.00 | 189.00 | 6596.00 | 3736.00 | 5291.00 | 9.37 | 8.79 | 4.87 | 161.20 | 89.01 | 136.24 | 0.00 | 3.76 | up |
| Hordeum_vulgare_newGene_699 | 23.00 | 31.00 | 21.00 | 57.00 | 71.00 | 66.00 | 0.48 | 0.70 | 0.49 | 1.24 | 1.50 | 1.47 | 0.00 | 1.16 | up |
| HORVU.MOREX.r2.7HG0592630 | 338.00 | 598.00 | 387.00 | 1176.00 | 747.00 | 1064.00 | 6.77 | 11.89 | 8.08 | 23.28 | 14.41 | 22.19 | 0.00 | 1.07 | up |
| HORVU.MOREX.r2.4HG0343490 | 375.00 | 440.00 | 273.00 | 998.00 | 1020.00 | 1406.00 | 4.76 | 5.55 | 3.61 | 12.54 | 12.49 | 18.61 | 0.00 | 1.54 | up |
| HORVU.MOREX.r2.5HG0392720 | 301.00 | 948.00 | 437.00 | 1484.00 | 1132.00 | 1207.00 | 5.25 | 16.42 | 7.94 | 25.59 | 19.02 | 21.92 | 0.00 | 1.04 | up |
| HORVU.MOREX.r2.3HG0253010 | 226.00 | 275.00 | 173.00 | 1351.00 | 689.00 | 986.00 | 3.68 | 4.45 | 2.93 | 21.79 | 10.83 | 16.76 | 0.00 | 1.98 | up |
| HORVU.MOREX.r2.5HG0407370 | 12.00 | 0.00 | 12.00 | 562.00 | 574.00 | 908.00 | 0.32 | 0.00 | 0.34 | 15.29 | 15.21 | 26.02 | 0.00 | 2.31 | up |
| HORVU.MOREX.r2.3HG0259280 | 0.00 | 0.00 | 0.00 | 80.00 | 74.00 | 66.00 | 0.00 | 0.00 | 0.00 | 1.26 | 1.13 | 1.09 | 0.00 | 4.35 | up |
| HORVU.MOREX.r2.4HG0321210 | 92.00 | 78.00 | 13.00 | 2835.00 | 1631.00 | 2331.00 | 2.17 | 1.83 | 0.31 | 66.71 | 37.41 | 57.81 | 0.00 | 3.20 | up |
| HORVU.MOREX.r2.2HG0179240 | 3306.00 | 4027.00 | 3180.00 | 8628.00 | 7881.00 | 10798.00 | 28.87 | 34.91 | 28.90 | 74.40 | 66.25 | 98.11 | 0.00 | 1.30 | up |
| HORVU.MOREX.r2.3HG0275320 | 12749.00 | 19731.00 | 14452.00 | 36540.00 | 32591.00 | 35474.00 | 319.59 | 491.11 | 377.17 | 904.59 | 786.56 | 925.29 | 0.00 | 1.09 | up |
| HORVU.MOREX.r2.3HG0234530 | 36.00 | 4.00 | 32.00 | 853.00 | 570.00 | 559.00 | 0.39 | 0.04 | 0.36 | 9.08 | 5.92 | 6.27 | 0.00 | 2.74 | up |
| HORVU.MOREX.r2.4HG0328290 | 543.00 | 379.00 | 361.00 | 1815.00 | 1432.00 | 1531.00 | 7.17 | 4.97 | 4.97 | 23.68 | 18.21 | 21.05 | 0.00 | 1.78 | up |
| HORVU.MOREX.r2.2HG0153890 | 0.00 | 0.00 | 0.00 | 146.00 | 102.00 | 149.00 | 0.00 | 0.00 | 0.00 | 2.86 | 1.94 | 3.08 | 0.00 | 5.06 | up |
| Hordeum_vulgare_newGene_1556 | 969.00 | 524.00 | 488.00 | 5316.00 | 4343.00 | 7377.00 | 23.05 | 12.38 | 12.07 | 124.94 | 99.52 | 182.69 | 0.00 | 2.82 | up |
| Hordeum_vulgare_newGene_1555 | 7269.00 | 7219.00 | 7331.00 | 22133.00 | 25670.00 | 34456.00 | 332.27 | 327.62 | 348.92 | 999.13 | 1130.04 | 1639.18 | 0.00 | 1.81 | up |
| HORVU.MOREX.r2.1HG0014010 | 366.00 | 243.00 | 324.00 | 661.00 | 960.00 | 1387.00 | 4.86 | 3.20 | 4.47 | 8.66 | 12.27 | 19.15 | 0.00 | 1.51 | up |
| HORVU.MOREX.r2.2HG0149830 | 57.00 | 5.00 | 24.00 | 178.00 | 356.00 | 566.00 | 1.09 | 0.10 | 0.48 | 3.37 | 6.60 | 11.33 | 0.00 | 1.80 | up |
| HORVU.MOREX.r2.7HG0557110 | 729.00 | 437.00 | 248.00 | 1154.00 | 1015.00 | 1175.00 | 5.48 | 3.26 | 1.94 | 8.57 | 7.35 | 9.19 | 0.00 | 1.09 | up |
| HORVU.MOREX.r2.3HG0252860 | 18.00 | 11.00 | 0.00 | 102.00 | 68.00 | 70.00 | 0.42 | 0.25 | 0.00 | 2.36 | 1.54 | 1.69 | 0.00 | 1.89 | up |
| HORVU.MOREX.r2.5HG0392680 | 1215.00 | 1392.00 | 1362.00 | 5114.00 | 3447.00 | 4993.00 | 4.87 | 5.54 | 5.68 | 20.24 | 13.30 | 20.82 | 0.00 | 1.67 | up |
| HORVU.MOREX.r2.2HG0091450 | 9.00 | 11.00 | 13.00 | 5918.00 | 2232.00 | 4422.00 | 0.09 | 0.11 | 0.15 | 65.61 | 24.12 | 51.65 | 0.00 | 7.38 | up |
| HORVU.MOREX.r2.2HG0109210 | 46.00 | 33.00 | 29.00 | 254.00 | 63.00 | 127.00 | 2.06 | 1.45 | 1.35 | 11.36 | 2.73 | 5.96 | 0.00 | 1.58 | up |
| HORVU.MOREX.r2.3HG0272680 | 132.00 | 226.00 | 187.00 | 373.00 | 486.00 | 481.00 | 0.64 | 1.09 | 0.95 | 1.80 | 2.29 | 2.45 | 0.00 | 1.19 | up |
| HORVU.MOREX.r2.3HG0183930 | 639.00 | 1153.00 | 787.00 | 7515.00 | 5404.00 | 7268.00 | 5.79 | 10.36 | 7.41 | 67.23 | 47.12 | 68.50 | 0.00 | 2.80 | up |
| HORVU.MOREX.r2.5HG0407120 | 0.00 | 0.00 | 3.00 | 117.00 | 166.00 | 124.00 | 0.00 | 0.00 | 0.04 | 1.95 | 2.69 | 2.17 | 0.00 | 4.75 | up |
| HORVU.MOREX.r2.4HG0286890 | 481.00 | 342.00 | 233.00 | 1285.00 | 1125.00 | 1598.00 | 6.71 | 4.73 | 3.37 | 17.70 | 15.10 | 23.18 | 0.00 | 1.76 | up |
| HORVU.MOREX.r2.5HG0425590 | 312.00 | 349.00 | 224.00 | 1107.00 | 984.00 | 1154.00 | 3.38 | 3.75 | 2.52 | 11.86 | 10.28 | 13.03 | 0.00 | 1.77 | up |
| HORVU.MOREX.r2.5HG0427340 | 21.00 | 42.00 | 19.00 | 978.00 | 711.00 | 927.00 | 0.35 | 0.71 | 0.32 | 16.61 | 11.76 | 16.59 | 0.00 | 4.58 | up |
| HORVU.MOREX.r2.6HG0452050 | 0.00 | 15.00 | 8.00 | 266.00 | 140.00 | 237.00 | 0.00 | 0.17 | 0.10 | 3.00 | 1.54 | 2.81 | 0.00 | 3.48 | up |
| HORVU.MOREX.r2.1HG0036040 | 18389.00 | 17113.00 | 12601.00 | 78909.00 | 81773.00 | 107330.00 | 464.99 | 429.65 | 331.72 | 1970.45 | 1990.71 | 2823.91 | 0.00 | 2.36 | up |
| HORVU.MOREX.r2.5HG0421480 | 68.00 | 28.00 | 36.00 | 162.00 | 132.00 | 118.00 | 1.16 | 0.46 | 0.63 | 2.71 | 2.16 | 2.08 | 0.00 | 1.37 | up |
| Hordeum_vulgare_newGene_1558 | 483.00 | 715.00 | 593.00 | 4295.00 | 4696.00 | 7111.00 | 17.10 | 25.16 | 21.88 | 150.40 | 160.31 | 262.38 | 0.00 | 2.98 | up |
| HORVU.MOREX.r2.6HG0504490 | 524.00 | 653.00 | 490.00 | 1292.00 | 1336.00 | 2099.00 | 7.21 | 8.93 | 7.03 | 17.57 | 17.71 | 30.09 | 0.00 | 1.40 | up |
| HORVU.MOREX.r2.2HG0079820 | 47.00 | 113.00 | 51.00 | 1301.00 | 873.00 | 1249.00 | 1.38 | 3.30 | 1.54 | 37.90 | 24.79 | 38.36 | 0.00 | 3.63 | up |
| HORVU.MOREX.r2.3HG0184850 | 114.00 | 50.00 | 40.00 | 646.00 | 255.00 | 602.00 | 3.70 | 1.60 | 1.33 | 20.72 | 7.96 | 20.32 | 0.00 | 2.36 | up |
| HORVU.MOREX.r2.4HG0329560 | 5734.00 | 9128.00 | 6048.00 | 28485.00 | 22075.00 | 25509.00 | 64.62 | 102.15 | 70.96 | 317.05 | 239.54 | 299.16 | 0.00 | 1.77 | up |
| HORVU.MOREX.r2.5HG0430540 | 808.00 | 421.00 | 233.00 | 3105.00 | 2177.00 | 2635.00 | 9.57 | 4.94 | 2.87 | 36.31 | 24.82 | 32.47 | 0.00 | 1.87 | up |
| HORVU.MOREX.r2.2HG0152550 | 149.00 | 221.00 | 165.00 | 434.00 | 432.00 | 588.00 | 1.32 | 1.94 | 1.52 | 3.79 | 3.68 | 5.41 | 0.00 | 1.34 | up |
| HORVU.MOREX.r2.4HG0299540 | 260.00 | 183.00 | 133.00 | 1217.00 | 1019.00 | 1594.00 | 5.14 | 3.58 | 2.73 | 23.76 | 19.39 | 32.80 | 0.00 | 2.51 | up |
| HORVU.MOREX.r2.2HG0143550 | 1144.00 | 1158.00 | 887.00 | 17054.00 | 10669.00 | 15451.00 | 19.73 | 19.83 | 15.93 | 290.64 | 177.26 | 277.44 | 0.00 | 3.58 | up |
| HORVU.MOREX.r2.3HG0246000 | 158.00 | 129.00 | 134.00 | 1124.00 | 873.00 | 1115.00 | 1.96 | 1.59 | 1.73 | 13.81 | 10.46 | 14.45 | 0.00 | 2.74 | up |
| HORVU.MOREX.r2.5HG0398310 | 339.00 | 270.00 | 211.00 | 6550.00 | 3466.00 | 4026.00 | 6.64 | 5.26 | 4.30 | 127.09 | 65.55 | 82.30 | 0.00 | 3.79 | up |
| HORVU.MOREX.r2.5HG0429830 | 139.00 | 38.00 | 52.00 | 1803.00 | 1055.00 | 1332.00 | 2.39 | 0.65 | 0.93 | 30.63 | 17.47 | 23.84 | 0.00 | 3.01 | up |
| HORVU.MOREX.r2.2HG0102570 | 351.00 | 376.00 | 190.00 | 1419.00 | 1155.00 | 1618.00 | 6.30 | 6.70 | 3.53 | 25.15 | 19.95 | 30.21 | 0.00 | 2.03 | up |
| HORVU.MOREX.r2.1HG0070560 | 122.00 | 140.00 | 70.00 | 1789.00 | 913.00 | 1397.00 | 1.50 | 1.71 | 0.90 | 21.81 | 10.85 | 17.94 | 0.00 | 3.29 | up |
| HORVU.MOREX.r2.4HG0343410 | 1021.00 | 706.00 | 571.00 | 3517.00 | 1518.00 | 2294.00 | 8.39 | 5.76 | 4.87 | 28.53 | 12.00 | 19.60 | 0.00 | 1.49 | up |
| HORVU.MOREX.r2.1HG0073030 | 41.00 | 37.00 | 42.00 | 181.00 | 145.00 | 173.00 | 0.39 | 0.35 | 0.41 | 1.72 | 1.33 | 1.72 | 0.00 | 1.86 | up |
| HORVU.MOREX.r2.1HG0041150 | 680.00 | 885.00 | 747.00 | 1867.00 | 1676.00 | 1870.00 | 7.38 | 9.53 | 8.43 | 20.00 | 17.50 | 21.11 | 0.00 | 1.16 | up |
| HORVU.MOREX.r2.7HG0553030 | 732.00 | 788.00 | 599.00 | 2113.00 | 1735.00 | 1841.00 | 6.67 | 7.12 | 5.68 | 19.02 | 15.22 | 17.45 | 0.00 | 1.35 | up |
| HORVU.MOREX.r2.7HG0527370 | 858.00 | 1921.00 | 1453.00 | 3494.00 | 4492.00 | 5220.00 | 7.26 | 16.14 | 12.80 | 29.21 | 36.60 | 45.97 | 0.00 | 1.50 | up |
| HORVU.MOREX.r2.1HG0066110 | 574.00 | 1055.00 | 688.00 | 2120.00 | 1841.00 | 2393.00 | 10.14 | 18.52 | 12.65 | 37.00 | 31.33 | 44.00 | 0.00 | 1.36 | up |
| HORVU.MOREX.r2.3HG0268840 | 51.00 | 21.00 | 16.00 | 609.00 | 256.00 | 727.00 | 1.99 | 0.81 | 0.64 | 23.45 | 9.59 | 29.52 | 0.00 | 3.36 | up |
| HORVU.MOREX.r2.2HG0152020 | 309.00 | 608.00 | 498.00 | 2674.00 | 1739.00 | 2231.00 | 3.35 | 6.54 | 5.61 | 28.60 | 18.13 | 25.14 | 0.00 | 2.06 | up |
| HORVU.MOREX.r2.6HG0464420 | 421.00 | 507.00 | 358.00 | 3485.00 | 2515.00 | 2551.00 | 1.84 | 2.19 | 1.62 | 15.01 | 10.56 | 11.58 | 0.00 | 2.61 | up |
| HORVU.MOREX.r2.1HG0060470 | 452.00 | 720.00 | 672.00 | 2090.00 | 1808.00 | 2150.00 | 6.34 | 10.03 | 9.82 | 28.99 | 24.45 | 31.42 | 0.00 | 1.61 | up |
| HORVU.MOREX.r2.4HG0338480 | 89.00 | 95.00 | 46.00 | 1231.00 | 684.00 | 1017.00 | 4.43 | 4.72 | 2.38 | 60.91 | 33.00 | 53.05 | 0.00 | 3.33 | up |
| HORVU.MOREX.r2.6HG0508040 | 877.00 | 586.00 | 305.00 | 4246.00 | 2977.00 | 4440.00 | 6.36 | 4.22 | 2.30 | 30.44 | 20.81 | 33.54 | 0.00 | 2.44 | up |
| HORVU.MOREX.r2.4HG0319060 | 104.00 | 73.00 | 28.00 | 1335.00 | 804.00 | 1253.00 | 1.88 | 1.32 | 0.52 | 23.95 | 14.07 | 23.69 | 0.00 | 3.51 | up |
| HORVU.MOREX.r2.UnG0625080 | 0.00 | 0.00 | 0.00 | 61.00 | 98.00 | 72.00 | 0.00 | 0.00 | 0.00 | 1.30 | 2.05 | 1.62 | 0.00 | 4.33 | up |
| Hordeum_vulgare_newGene_5991 | 9322.00 | 6739.00 | 6699.00 | 14468.00 | 15287.00 | 23102.00 | 89.31 | 64.10 | 66.82 | 136.90 | 141.01 | 230.31 | 0.00 | 1.12 | up |
| HORVU.MOREX.r2.2HG0105160 | 84.00 | 79.00 | 56.00 | 2471.00 | 1888.00 | 2374.00 | 1.42 | 1.32 | 0.99 | 41.37 | 30.81 | 41.87 | 0.00 | 4.70 | up |
| HORVU.MOREX.r2.4HG0348020 | 0.00 | 0.00 | 0.00 | 109.00 | 88.00 | 138.00 | 0.00 | 0.00 | 0.00 | 2.56 | 2.01 | 3.42 | 0.00 | 4.81 | up |
| HORVU.MOREX.r2.1HG0043200 | 34203.00 | 29323.00 | 18630.00 | 113921.00 | 114546.00 | 149940.00 | 786.27 | 669.28 | 445.86 | 2586.16 | 2535.07 | 3586.40 | 0.00 | 2.06 | up |
| HORVU.MOREX.r2.1HG0071530 | 106.00 | 145.00 | 115.00 | 352.00 | 366.00 | 383.00 | 2.69 | 3.69 | 3.06 | 8.88 | 9.02 | 10.20 | 0.00 | 1.49 | up |
| HORVU.MOREX.r2.7HG0616260 | 284.00 | 699.00 | 804.00 | 1587.00 | 1519.00 | 1856.00 | 4.19 | 10.26 | 12.39 | 23.19 | 21.63 | 28.58 | 0.00 | 1.30 | up |
| HORVU.MOREX.r2.2HG0139510 | 44.00 | 40.00 | 50.00 | 116.00 | 78.00 | 127.00 | 0.48 | 0.42 | 0.56 | 1.23 | 0.81 | 1.42 | 0.00 | 1.09 | up |
| HORVU.MOREX.r2.6HG0520360 | 13.00 | 16.00 | 16.00 | 100.00 | 78.00 | 128.00 | 0.42 | 0.51 | 0.54 | 3.26 | 2.47 | 4.41 | 0.00 | 2.33 | up |
| HORVU.MOREX.r2.4HG0289900 | 161.00 | 367.00 | 246.00 | 1084.00 | 788.00 | 1042.00 | 2.26 | 5.12 | 3.60 | 15.06 | 10.68 | 15.27 | 0.00 | 1.74 | up |
| HORVU.MOREX.r2.6HG0459660 | 28.00 | 4.00 | 3.00 | 4961.00 | 4711.00 | 7384.00 | 0.30 | 0.04 | 0.03 | 52.47 | 48.57 | 82.29 | 0.00 | 3.36 | up |
| HORVU.MOREX.r2.3HG0266360 | 29.00 | 31.00 | 48.00 | 101.00 | 82.00 | 101.00 | 0.23 | 0.24 | 0.40 | 0.80 | 0.63 | 0.85 | 0.00 | 1.19 | up |
| HORVU.MOREX.r2.5HG0367820 | 179.00 | 353.00 | 234.00 | 1690.00 | 1627.00 | 2039.00 | 6.22 | 12.19 | 8.48 | 58.09 | 54.55 | 73.88 | 0.00 | 2.63 | up |
| HORVU.MOREX.r2.1HG0043040 | 228.00 | 256.00 | 154.00 | 652.00 | 595.00 | 747.00 | 3.57 | 3.99 | 2.51 | 10.12 | 8.99 | 12.21 | 0.00 | 1.54 | up |
| HORVU.MOREX.r2.5HG0439680 | 2592.00 | 3211.00 | 1568.00 | 15135.00 | 10545.00 | 12517.00 | 99.81 | 122.79 | 62.88 | 575.66 | 391.00 | 501.60 | 0.00 | 2.22 | up |
| HORVU.MOREX.r2.7HG0605420 | 497.00 | 527.00 | 401.00 | 1046.00 | 1126.00 | 1217.00 | 5.01 | 5.28 | 4.21 | 10.42 | 10.94 | 12.78 | 0.00 | 1.18 | up |
| HORVU.MOREX.r2.6HG0471860 | 162.00 | 57.00 | 100.00 | 768.00 | 431.00 | 587.00 | 3.04 | 1.06 | 1.94 | 14.27 | 7.81 | 11.48 | 0.00 | 2.13 | up |
| HORVU.MOREX.r2.1HG0050020 | 11072.00 | 14435.00 | 14469.00 | 54632.00 | 38050.00 | 47126.00 | 68.43 | 88.58 | 93.10 | 333.45 | 226.41 | 303.06 | 0.00 | 1.71 | up |
| HORVU.MOREX.r2.3HG0250270 | 66.00 | 95.00 | 43.00 | 227.00 | 404.00 | 416.00 | 1.19 | 1.72 | 0.81 | 4.08 | 7.08 | 7.88 | 0.00 | 2.08 | up |
| HORVU.MOREX.r2.3HG0258800 | 28.00 | 69.00 | 57.00 | 89.00 | 305.00 | 312.00 | 0.15 | 0.37 | 0.32 | 0.47 | 1.58 | 1.75 | 0.00 | 1.73 | up |
| HORVU.MOREX.r2.2HG0171910 | 11693.00 | 5824.00 | 5391.00 | 43367.00 | 73510.00 | 133082.00 | 73.44 | 36.31 | 35.25 | 268.98 | 444.49 | 869.70 | 0.00 | 2.45 | up |
| HORVU.MOREX.r2.2HG0097090 | 153.00 | 146.00 | 144.00 | 694.00 | 530.00 | 892.00 | 1.04 | 0.98 | 1.01 | 4.66 | 3.46 | 6.31 | 0.00 | 2.09 | up |
| HORVU.MOREX.r2.5HG0440790 | 82.00 | 72.00 | 81.00 | 214.00 | 237.00 | 182.00 | 1.66 | 1.46 | 1.72 | 4.30 | 4.65 | 3.85 | 0.00 | 1.31 | up |
| HORVU.MOREX.r2.2HG0107840 | 202.00 | 117.00 | 148.00 | 483.00 | 416.00 | 413.00 | 3.55 | 2.04 | 2.70 | 8.37 | 7.04 | 7.53 | 0.00 | 1.35 | up |
| HORVU.MOREX.r2.5HG0383480 | 117.00 | 85.00 | 56.00 | 513.00 | 350.00 | 439.00 | 1.33 | 0.95 | 0.66 | 5.80 | 3.85 | 5.22 | 0.00 | 2.11 | up |
| HORVU.MOREX.r2.7HG0534360 | 7.00 | 18.00 | 31.00 | 5277.00 | 2085.00 | 4019.00 | 0.07 | 0.20 | 0.36 | 59.31 | 22.84 | 47.59 | 0.00 | 5.11 | up |
| HORVU.MOREX.r2.5HG0435700 | 0.00 | 3.00 | 5.00 | 193.00 | 168.00 | 218.00 | 0.00 | 0.02 | 0.04 | 1.61 | 1.36 | 1.91 | 0.00 | 4.78 | up |
| HORVU.MOREX.r2.5HG0424970 | 372.00 | 721.00 | 286.00 | 2276.00 | 1885.00 | 2185.00 | 4.74 | 9.12 | 3.79 | 28.66 | 23.14 | 28.98 | 0.00 | 2.02 | up |
| HORVU.MOREX.r2.1HG0053690 | 233.00 | 179.00 | 78.00 | 1364.00 | 968.00 | 1716.00 | 10.01 | 7.65 | 3.47 | 58.00 | 40.13 | 76.92 | 0.00 | 2.68 | up |
| HORVU.MOREX.r2.3HG0235440 | 60.00 | 86.00 | 94.00 | 125.00 | 217.00 | 217.00 | 0.72 | 1.03 | 1.19 | 1.50 | 2.54 | 2.74 | 0.00 | 1.06 | up |
| HORVU.MOREX.r2.4HG0275980 | 15.00 | 50.00 | 30.00 | 244.00 | 299.00 | 343.00 | 0.26 | 0.88 | 0.54 | 4.28 | 5.11 | 6.33 | 0.00 | 2.79 | up |
| HORVU.MOREX.r2.2HG0142130 | 495.00 | 366.00 | 260.00 | 1632.00 | 920.00 | 1484.00 | 4.93 | 3.62 | 2.70 | 16.07 | 8.83 | 15.40 | 0.00 | 1.67 | up |
| HORVU.MOREX.r2.3HG0260240 | 128.00 | 93.00 | 34.00 | 2777.00 | 1943.00 | 3914.00 | 3.26 | 2.35 | 0.89 | 69.93 | 47.72 | 103.88 | 0.00 | 3.72 | up |
| HORVU.MOREX.r2.3HG0205850 | 376.00 | 252.00 | 221.00 | 698.00 | 714.00 | 734.00 | 6.73 | 4.48 | 4.10 | 12.32 | 12.30 | 13.65 | 0.00 | 1.23 | up |
| HORVU.MOREX.r2.7HG0591390 | 116.00 | 84.00 | 48.00 | 564.00 | 522.00 | 514.00 | 2.17 | 1.56 | 0.92 | 10.48 | 9.45 | 10.06 | 0.00 | 2.45 | up |
| HORVU.MOREX.r2.3HG0204450 | 406.00 | 220.00 | 142.00 | 770.00 | 574.00 | 442.00 | 6.89 | 3.70 | 2.51 | 12.92 | 9.38 | 7.82 | 0.01 | 1.03 | up |
| HORVU.MOREX.r2.3HG0244530 | 204.00 | 265.00 | 188.00 | 675.00 | 701.00 | 837.00 | 1.45 | 1.88 | 1.39 | 4.76 | 4.82 | 6.22 | 0.00 | 1.65 | up |
| HORVU.MOREX.r2.5HG0399300 | 701.00 | 1074.00 | 893.00 | 2198.00 | 1983.00 | 2244.00 | 4.47 | 6.80 | 5.92 | 13.84 | 12.17 | 14.89 | 0.00 | 1.19 | up |
| HORVU.MOREX.r2.3HG0206740 | 87.00 | 90.00 | 55.00 | 740.00 | 575.00 | 816.00 | 0.36 | 0.38 | 0.24 | 3.08 | 2.34 | 3.58 | 0.00 | 2.98 | up |
| HORVU.MOREX.r2.4HG0333690 | 719.00 | 783.00 | 523.00 | 1970.00 | 1558.00 | 2776.00 | 10.47 | 11.32 | 7.93 | 28.35 | 21.85 | 42.10 | 0.00 | 1.52 | up |
| HORVU.MOREX.r2.1HG0036070 | 22.00 | 9.00 | 8.00 | 57.00 | 58.00 | 88.00 | 0.54 | 0.22 | 0.20 | 1.38 | 1.35 | 2.23 | 0.00 | 1.81 | up |
| HORVU.MOREX.r2.7HG0568910 | 196.00 | 442.00 | 220.00 | 5300.00 | 3788.00 | 4079.00 | 4.03 | 9.04 | 4.72 | 107.94 | 75.20 | 87.53 | 0.00 | 3.64 | up |
| HORVU.MOREX.r2.1HG0053700 | 212.00 | 186.00 | 62.00 | 843.00 | 609.00 | 1619.00 | 13.50 | 11.75 | 4.08 | 53.16 | 37.46 | 107.64 | 0.00 | 1.92 | up |
| HORVU.MOREX.r2.6HG0508510 | 0.00 | 0.00 | 0.00 | 1282.00 | 758.00 | 2103.00 | 0.00 | 0.00 | 0.00 | 18.22 | 10.50 | 31.50 | 0.00 | 5.93 | up |
| HORVU.MOREX.r2.4HG0284710 | 1153.00 | 753.00 | 473.00 | 5646.00 | 4752.00 | 7096.00 | 20.01 | 12.98 | 8.55 | 96.79 | 79.42 | 128.18 | 0.00 | 2.62 | up |
| HORVU.MOREX.r2.6HG0521010 | 152.00 | 281.00 | 132.00 | 375.00 | 455.00 | 455.00 | 2.70 | 4.98 | 2.44 | 6.62 | 7.82 | 8.47 | 0.00 | 1.07 | up |
| HORVU.MOREX.r2.3HG0226850 | 49.00 | 33.00 | 42.00 | 999.00 | 469.00 | 598.00 | 0.53 | 0.36 | 0.48 | 10.86 | 4.96 | 6.84 | 0.00 | 3.61 | up |
| HORVU.MOREX.r2.6HG0501140 | 155.00 | 262.00 | 141.00 | 506.00 | 404.00 | 515.00 | 3.87 | 6.52 | 3.67 | 12.51 | 9.74 | 13.42 | 0.00 | 1.24 | up |
| HORVU.MOREX.r2.3HG0265850 | 1144.00 | 944.00 | 977.00 | 8877.00 | 9276.00 | 15179.00 | 19.50 | 15.98 | 17.33 | 149.50 | 152.30 | 269.35 | 0.00 | 3.23 | up |
| HORVU.MOREX.r2.2HG0106420 | 163.00 | 147.00 | 98.00 | 1583.00 | 919.00 | 950.00 | 1.53 | 1.37 | 0.95 | 14.71 | 8.32 | 9.30 | 0.00 | 2.83 | up |
| HORVU.MOREX.r2.UnG0626230 | 41.00 | 14.00 | 32.00 | 196.00 | 347.00 | 453.00 | 1.00 | 0.32 | 0.79 | 4.73 | 8.16 | 11.50 | 0.00 | 2.90 | up |
| HORVU.MOREX.r2.4HG0289360 | 44.00 | 43.00 | 36.00 | 301.00 | 114.00 | 182.00 | 0.74 | 0.73 | 0.64 | 5.11 | 1.87 | 3.25 | 0.00 | 1.93 | up |
| HORVU.MOREX.r2.7HG0534470 | 101.00 | 260.00 | 398.00 | 1030.00 | 751.00 | 961.00 | 2.75 | 7.06 | 11.37 | 27.89 | 19.81 | 27.41 | 0.00 | 1.56 | up |
| HORVU.MOREX.r2.2HG0081850 | 7.00 | 36.00 | 21.00 | 1128.00 | 810.00 | 1117.00 | 0.04 | 0.24 | 0.15 | 7.74 | 5.42 | 8.08 | 0.00 | 4.88 | up |
| HORVU.MOREX.r2.5HG0352010 | 8438.00 | 7700.00 | 6159.00 | 23228.00 | 21085.00 | 34413.00 | 211.51 | 191.63 | 160.74 | 575.03 | 508.88 | 897.62 | 0.00 | 1.70 | up |
| HORVU.MOREX.r2.3HG0246160 | 40.00 | 70.00 | 103.00 | 153.00 | 211.00 | 230.00 | 0.20 | 0.36 | 0.55 | 0.78 | 1.06 | 1.24 | 0.00 | 1.25 | up |
| HORVU.MOREX.r2.7HG0609160 | 502.00 | 527.00 | 401.00 | 3606.00 | 2477.00 | 2602.00 | 3.59 | 3.75 | 2.99 | 25.53 | 17.10 | 19.41 | 0.00 | 2.47 | up |
| HORVU.MOREX.r2.2HG0114500 | 19.00 | 16.00 | 2.00 | 182.00 | 117.00 | 127.00 | 0.16 | 0.14 | 0.01 | 1.56 | 0.98 | 1.15 | 0.00 | 2.68 | up |
| HORVU.MOREX.r2.6HG0501260 | 10.00 | 2.00 | 3.00 | 2418.00 | 1186.00 | 2348.00 | 0.27 | 0.05 | 0.08 | 64.59 | 30.87 | 66.06 | 0.00 | 5.63 | up |
| HORVU.MOREX.r2.3HG0269260 | 158.00 | 154.00 | 106.00 | 425.00 | 296.00 | 469.00 | 1.77 | 1.71 | 1.23 | 4.70 | 3.18 | 5.46 | 0.00 | 1.38 | up |
| HORVU.MOREX.r2.UnG0633100 | 37.00 | 32.00 | 43.00 | 3045.00 | 1554.00 | 200.00 | 0.40 | 0.34 | 0.48 | 32.50 | 16.17 | 2.25 | 0.00 | 2.50 | up |
| HORVU.MOREX.r2.7HG0542590 | 7.00 | 22.00 | 24.00 | 232.00 | 189.00 | 196.00 | 0.25 | 0.83 | 0.97 | 9.06 | 7.17 | 8.03 | 0.00 | 3.03 | up |
| HORVU.MOREX.r2.3HG0212160 | 437.00 | 239.00 | 209.00 | 1914.00 | 1186.00 | 1594.00 | 8.50 | 4.61 | 4.22 | 36.76 | 22.20 | 32.24 | 0.00 | 2.17 | up |
| HORVU.MOREX.r2.6HG0494790 | 2798.00 | 3404.00 | 2900.00 | 6140.00 | 6635.00 | 6775.00 | 22.85 | 27.61 | 24.66 | 49.53 | 52.17 | 57.58 | 0.00 | 1.05 | up |
| HORVU.MOREX.r2.1HG0060530 | 59.00 | 132.00 | 108.00 | 367.00 | 280.00 | 426.00 | 1.27 | 2.82 | 2.41 | 7.82 | 5.82 | 9.57 | 0.00 | 1.64 | up |
| HORVU.MOREX.r2.3HG0187510 | 79.00 | 122.00 | 80.00 | 231.00 | 198.00 | 244.00 | 0.72 | 1.11 | 0.76 | 2.09 | 1.74 | 2.32 | 0.00 | 1.15 | up |
| HORVU.MOREX.r2.5HG0402390 | 245.00 | 67.00 | 18.00 | 4828.00 | 3013.00 | 4688.00 | 7.90 | 2.15 | 0.58 | 154.03 | 93.72 | 157.60 | 0.00 | 2.48 | up |
| HORVU.MOREX.r2.2HG0100730 | 1993.00 | 2784.00 | 3018.00 | 8897.00 | 7780.00 | 8440.00 | 57.08 | 79.18 | 90.01 | 251.70 | 214.59 | 251.57 | 0.00 | 1.59 | up |
| HORVU.MOREX.r2.3HG0194610 | 541.00 | 309.00 | 230.00 | 1146.00 | 822.00 | 1114.00 | 4.83 | 2.74 | 2.14 | 10.11 | 7.06 | 10.35 | 0.00 | 1.35 | up |
| HORVU.MOREX.r2.1HG0050640 | 96.00 | 212.00 | 57.00 | 1600.00 | 1214.00 | 1405.00 | 1.64 | 3.59 | 1.01 | 26.95 | 19.92 | 24.92 | 0.00 | 3.11 | up |
| HORVU.MOREX.r2.7HG0584900 | 0.00 | 0.00 | 2.00 | 113.00 | 134.00 | 101.00 | 0.00 | 0.00 | 0.03 | 2.01 | 2.31 | 1.88 | 0.00 | 4.70 | up |
| HORVU.MOREX.r2.3HG0193480 | 127.00 | 165.00 | 173.00 | 766.00 | 599.00 | 559.00 | 3.28 | 4.22 | 4.63 | 19.53 | 14.90 | 15.03 | 0.00 | 1.92 | up |
| Hordeum_vulgare_newGene_4744 | 94.00 | 85.00 | 103.00 | 1005.00 | 501.00 | 577.00 | 0.88 | 0.79 | 1.00 | 9.27 | 4.50 | 5.61 | 0.00 | 2.61 | up |
| HORVU.MOREX.r2.3HG0266870 | 759.00 | 799.00 | 631.00 | 1453.00 | 1613.00 | 1678.00 | 15.86 | 16.60 | 13.72 | 30.00 | 32.47 | 36.51 | 0.00 | 1.05 | up |
| Hordeum_vulgare_newGene_6675 | 76.00 | 49.00 | 16.00 | 305.00 | 121.00 | 120.00 | 0.85 | 0.72 | 0.17 | 3.11 | 1.33 | 1.48 | 0.00 | 1.46 | up |
| HORVU.MOREX.r2.6HG0504970 | 125.00 | 124.00 | 127.00 | 374.00 | 271.00 | 347.00 | 0.93 | 0.92 | 0.99 | 2.76 | 1.95 | 2.70 | 0.00 | 1.29 | up |
| HORVU.MOREX.r2.2HG0170800 | 11.00 | 41.00 | 18.00 | 18318.00 | 9978.00 | 14613.00 | 0.12 | 0.45 | 0.20 | 205.88 | 109.33 | 173.05 | 0.00 | 6.71 | up |
| HORVU.MOREX.r2.2HG0083800 | 38.00 | 29.00 | 22.00 | 108.00 | 104.00 | 87.00 | 0.28 | 0.22 | 0.17 | 0.81 | 0.77 | 0.69 | 0.00 | 1.52 | up |
| HORVU.MOREX.r2.3HG0210080 | 156.00 | 135.00 | 157.00 | 331.00 | 391.00 | 286.00 | 1.27 | 1.09 | 1.33 | 2.66 | 3.07 | 2.43 | 0.00 | 1.07 | up |
| HORVU.MOREX.r2.2HG0129010 | 264.00 | 354.00 | 230.00 | 1427.00 | 894.00 | 1228.00 | 1.99 | 2.65 | 1.81 | 10.65 | 6.51 | 9.66 | 0.00 | 1.93 | up |
| HORVU.MOREX.r2.3HG0258920 | 206.00 | 262.00 | 220.00 | 727.00 | 621.00 | 619.00 | 5.22 | 6.60 | 5.80 | 18.21 | 15.16 | 16.33 | 0.00 | 1.43 | up |
| HORVU.MOREX.r2.4HG0286320 | 399.00 | 940.00 | 814.00 | 1195.00 | 2094.00 | 2588.00 | 6.62 | 15.49 | 14.07 | 19.60 | 33.49 | 44.73 | 0.00 | 1.27 | up |
| HORVU.MOREX.r2.5HG0361390 | 611.00 | 469.00 | 351.00 | 1857.00 | 1756.00 | 1857.00 | 18.68 | 14.25 | 11.16 | 56.13 | 51.74 | 59.13 | 0.00 | 1.82 | up |
| HORVU.MOREX.r2.4HG0324220 | 1183.00 | 1334.00 | 1044.00 | 38428.00 | 24441.00 | 33270.00 | 11.27 | 12.62 | 10.35 | 361.82 | 224.34 | 330.05 | 0.00 | 4.56 | up |
| HORVU.MOREX.r2.4HG0297490 | 654.00 | 639.00 | 470.00 | 2559.00 | 1887.00 | 2333.00 | 7.55 | 7.33 | 5.65 | 29.22 | 21.00 | 28.06 | 0.00 | 1.84 | up |
| HORVU.MOREX.r2.1HG0028050 | 0.00 | 0.00 | 0.00 | 80.00 | 72.00 | 88.00 | 0.00 | 0.00 | 0.00 | 2.43 | 2.15 | 2.82 | 0.00 | 4.45 | up |
| HORVU.MOREX.r2.7HG0534240 | 1.00 | 0.00 | 0.00 | 1914.00 | 694.00 | 2107.00 | 0.01 | 0.00 | 0.00 | 21.51 | 7.59 | 24.94 | 0.00 | 7.46 | up |
| HORVU.MOREX.r2.2HG0108440 | 47.00 | 41.00 | 12.00 | 89.00 | 100.00 | 106.00 | 0.69 | 0.59 | 0.18 | 1.28 | 1.39 | 1.60 | 0.01 | 1.25 | up |
| HORVU.MOREX.r2.3HG0249560 | 136.00 | 142.00 | 133.00 | 372.00 | 351.00 | 382.00 | 1.94 | 2.01 | 1.97 | 5.24 | 4.82 | 5.68 | 0.00 | 1.34 | up |
| HORVU.MOREX.r2.7HG0528920 | 238.00 | 219.00 | 239.00 | 702.00 | 475.00 | 773.00 | 2.83 | 2.58 | 2.95 | 8.24 | 5.43 | 9.56 | 0.00 | 1.37 | up |
| HORVU.MOREX.r2.3HG0207730 | 11.00 | 0.00 | 8.00 | 143.00 | 390.00 | 422.00 | 0.32 | 0.00 | 0.26 | 4.41 | 11.77 | 13.77 | 0.00 | 2.04 | up |
| HORVU.MOREX.r2.1HG0051720 | 226.00 | 155.00 | 89.00 | 567.00 | 331.00 | 388.00 | 3.62 | 2.46 | 1.47 | 8.99 | 5.11 | 6.48 | 0.00 | 1.26 | up |
| HORVU.MOREX.r2.2HG0129580 | 12.00 | 46.00 | 18.00 | 90.00 | 85.00 | 137.00 | 0.32 | 1.27 | 0.51 | 2.52 | 2.31 | 4.02 | 0.00 | 1.60 | up |
| HORVU.MOREX.r2.3HG0192120 | 1094.00 | 822.00 | 659.00 | 4735.00 | 3046.00 | 4082.00 | 20.32 | 15.16 | 12.73 | 86.87 | 54.48 | 78.91 | 0.00 | 2.05 | up |
| HORVU.MOREX.r2.7HG0537910 | 62.00 | 22.00 | 49.00 | 304.00 | 220.00 | 150.00 | 1.49 | 0.52 | 1.24 | 7.30 | 5.14 | 3.80 | 0.00 | 1.93 | up |
| HORVU.MOREX.r2.2HG0172670 | 81.00 | 119.00 | 69.00 | 265.00 | 201.00 | 239.00 | 0.78 | 1.14 | 0.69 | 2.54 | 1.87 | 2.41 | 0.00 | 1.26 | up |
| Hordeum_vulgare_newGene_1522 | 2312.00 | 2508.00 | 2244.00 | 5062.00 | 4815.00 | 4881.00 | 59.14 | 63.69 | 59.76 | 127.88 | 118.59 | 129.90 | 0.00 | 1.01 | up |
| HORVU.MOREX.r2.1HG0010770 | 10.00 | 22.00 | 25.00 | 283.00 | 201.00 | 275.00 | 0.10 | 0.24 | 0.29 | 3.09 | 2.14 | 3.18 | 0.00 | 3.26 | up |
| HORVU.MOREX.r2.5HG0419410 | 50.00 | 66.00 | 39.00 | 84.00 | 151.00 | 150.00 | 0.70 | 0.92 | 0.56 | 1.17 | 2.04 | 2.19 | 0.00 | 1.13 | up |
| HORVU.MOREX.r2.1HG0015850 | 67.00 | 42.00 | 46.00 | 210.00 | 213.00 | 189.00 | 0.91 | 0.57 | 0.65 | 2.84 | 2.81 | 2.69 | 0.00 | 1.79 | up |
| HORVU.MOREX.r2.1HG0045440 | 201.00 | 145.00 | 116.00 | 550.00 | 395.00 | 557.00 | 3.41 | 2.44 | 2.04 | 9.24 | 6.47 | 9.85 | 0.00 | 1.55 | up |
| HORVU.MOREX.r2.3HG0187780 | 874.00 | 927.00 | 651.00 | 2640.00 | 1743.00 | 2781.00 | 7.46 | 7.86 | 5.78 | 22.26 | 14.33 | 24.71 | 0.00 | 1.44 | up |
| HORVU.MOREX.r2.1HG0077090 | 12.00 | 21.00 | 28.00 | 58.00 | 102.00 | 52.00 | 0.08 | 0.15 | 0.20 | 0.41 | 0.71 | 0.39 | 0.00 | 1.40 | up |
| HORVU.MOREX.r2.1HG0071870 | 15.00 | 8.00 | 2.00 | 90.00 | 88.00 | 89.00 | 0.46 | 0.25 | 0.07 | 2.81 | 2.69 | 2.94 | 0.00 | 2.60 | up |
| HORVU.MOREX.r2.7HG0611950 | 100.00 | 96.00 | 90.00 | 1522.00 | 1442.00 | 2119.00 | 0.56 | 0.54 | 0.53 | 8.52 | 7.87 | 12.50 | 0.00 | 3.93 | up |
| HORVU.MOREX.r2.6HG0456060 | 74.00 | 73.00 | 44.00 | 931.00 | 611.00 | 1205.00 | 4.74 | 4.65 | 2.95 | 59.40 | 37.96 | 81.01 | 0.00 | 3.48 | up |
| HORVU.MOREX.r2.6HG0508410 | 118.00 | 75.00 | 82.00 | 679.00 | 764.00 | 818.00 | 4.22 | 2.67 | 3.05 | 24.04 | 26.37 | 30.54 | 0.00 | 2.85 | up |
| HORVU.MOREX.r2.5HG0441570 | 666.00 | 1411.00 | 1034.00 | 2498.00 | 1968.00 | 2806.00 | 8.20 | 17.26 | 13.26 | 30.39 | 23.34 | 35.96 | 0.00 | 1.12 | up |
| HORVU.MOREX.r2.7HG0582550 | 6115.00 | 6769.00 | 5576.00 | 73233.00 | 47955.00 | 68535.00 | 75.51 | 82.98 | 71.68 | 893.00 | 570.09 | 880.54 | 0.00 | 3.22 | up |
| HORVU.MOREX.r2.4HG0342490 | 2987.00 | 2706.00 | 1807.00 | 32108.00 | 21491.00 | 29216.00 | 43.75 | 39.35 | 27.55 | 464.50 | 303.10 | 445.34 | 0.00 | 3.27 | up |
| HORVU.MOREX.r2.5HG0355940 | 41.00 | 99.00 | 66.00 | 478.00 | 395.00 | 477.00 | 0.53 | 1.27 | 0.90 | 6.15 | 4.95 | 6.47 | 0.00 | 2.46 | up |
| HORVU.MOREX.r2.7HG0549060 | 1487.00 | 897.00 | 650.00 | 4557.00 | 4333.00 | 6286.00 | 12.28 | 7.36 | 5.59 | 37.17 | 34.46 | 54.03 | 0.00 | 2.11 | up |
| HORVU.MOREX.r2.2HG0080380 | 940.00 | 764.00 | 601.00 | 1435.00 | 1623.00 | 2136.00 | 24.27 | 19.60 | 16.17 | 36.62 | 40.38 | 57.45 | 0.00 | 1.08 | up |
| HORVU.MOREX.r2.UnG0624360 | 84.00 | 59.00 | 69.00 | 543.00 | 413.00 | 572.00 | 0.92 | 0.65 | 0.79 | 5.92 | 4.39 | 6.57 | 0.00 | 2.64 | up |
| HORVU.MOREX.r2.7HG0554270 | 1075.00 | 2015.00 | 1572.00 | 4025.00 | 2893.00 | 3337.00 | 68.71 | 127.85 | 104.58 | 254.02 | 177.97 | 221.87 | 0.00 | 1.05 | up |
| HORVU.MOREX.r2.5HG0430670 | 91.00 | 172.00 | 113.00 | 423.00 | 310.00 | 357.00 | 0.79 | 1.48 | 1.02 | 3.61 | 2.58 | 3.21 | 0.00 | 1.40 | up |
| HORVU.MOREX.r2.3HG0271330 | 53.00 | 54.00 | 63.00 | 1310.00 | 1028.00 | 1247.00 | 0.84 | 0.87 | 1.05 | 20.90 | 15.98 | 20.95 | 0.00 | 4.17 | up |
| HORVU.MOREX.r2.2HG0092500 | 3715.00 | 2274.00 | 1460.00 | 7595.00 | 7776.00 | 10676.00 | 68.16 | 41.42 | 27.88 | 137.59 | 137.34 | 203.80 | 0.00 | 1.62 | up |
| HORVU.MOREX.r2.2HG0102100 | 8376.00 | 7832.00 | 7685.00 | 28011.00 | 21440.00 | 22433.00 | 90.54 | 84.06 | 86.49 | 299.02 | 223.14 | 252.33 | 0.00 | 1.51 | up |
| HORVU.MOREX.r2.7HG0546970 | 422.00 | 471.00 | 316.00 | 1238.00 | 863.00 | 1141.00 | 4.43 | 4.91 | 3.46 | 12.85 | 8.74 | 12.48 | 0.00 | 1.33 | up |
| HORVU.MOREX.r2.6HG0473430 | 2607.00 | 3497.00 | 2701.00 | 7700.00 | 6342.00 | 7656.00 | 67.38 | 89.73 | 72.66 | 196.55 | 157.80 | 205.91 | 0.00 | 1.23 | up |
| HORVU.MOREX.r2.7HG0587950 | 23.00 | 27.00 | 7.00 | 576.00 | 353.00 | 434.00 | 1.00 | 1.14 | 0.30 | 25.04 | 14.95 | 19.87 | 0.00 | 3.97 | up |
| HORVU.MOREX.r2.1HG0002210 | 0.00 | 0.00 | 0.00 | 599.00 | 541.00 | 898.00 | 0.00 | 0.00 | 0.00 | 9.46 | 8.33 | 14.97 | 0.00 | 7.08 | up |
| HORVU.MOREX.r2.7HG0556560 | 7.00 | 6.00 | 17.00 | 217.00 | 171.00 | 183.00 | 0.28 | 0.23 | 0.72 | 8.97 | 6.88 | 7.95 | 0.00 | 3.57 | up |
| HORVU.MOREX.r2.5HG0396880 | 2138.00 | 1280.00 | 768.00 | 6205.00 | 5930.00 | 9804.00 | 34.82 | 20.70 | 13.02 | 99.82 | 93.00 | 166.18 | 0.00 | 1.89 | up |
| HORVU.MOREX.r2.1HG0067870 | 706.00 | 1004.00 | 612.00 | 2033.00 | 1614.00 | 2007.00 | 16.15 | 22.81 | 14.58 | 45.96 | 35.56 | 47.80 | 0.00 | 1.20 | up |
| HORVU.MOREX.r2.7HG0542400 | 429.00 | 578.00 | 391.00 | 1328.00 | 965.00 | 1338.00 | 3.83 | 5.12 | 3.63 | 11.72 | 8.29 | 12.44 | 0.00 | 1.29 | up |
| HORVU.MOREX.r2.3HG0255980 | 1333.00 | 1325.00 | 1398.00 | 2920.00 | 2575.00 | 3342.00 | 9.61 | 9.49 | 10.50 | 20.80 | 17.89 | 25.09 | 0.00 | 1.05 | up |
| HORVU.MOREX.r2.4HG0335580 | 31.00 | 31.00 | 15.00 | 922.00 | 593.00 | 857.00 | 0.38 | 0.39 | 0.19 | 11.45 | 7.18 | 11.22 | 0.00 | 4.49 | up |
| HORVU.MOREX.r2.1HG0029940 | 1924.00 | 2143.00 | 1882.00 | 6280.00 | 5040.00 | 5663.00 | 32.06 | 35.45 | 32.64 | 103.33 | 80.85 | 98.19 | 0.00 | 1.45 | up |
| HORVU.MOREX.r2.6HG0475980 | 206.00 | 143.00 | 74.00 | 478.00 | 280.00 | 353.00 | 2.80 | 1.93 | 1.04 | 6.44 | 3.68 | 5.00 | 0.00 | 1.20 | up |
| HORVU.MOREX.r2.3HG0234560 | 33.00 | 28.00 | 35.00 | 222.00 | 203.00 | 194.00 | 0.41 | 0.34 | 0.45 | 2.74 | 2.45 | 2.52 | 0.00 | 2.46 | up |
| HORVU.MOREX.r2.5HG0399220 | 3848.00 | 6068.00 | 4649.00 | 9704.00 | 12459.00 | 13231.00 | 43.28 | 67.77 | 54.45 | 107.80 | 134.93 | 154.86 | 0.00 | 1.20 | up |
| HORVU.MOREX.r2.4HG0325280 | 4696.00 | 4783.00 | 4849.00 | 12372.00 | 13526.00 | 10215.00 | 40.52 | 40.98 | 43.56 | 105.42 | 112.36 | 91.71 | 0.00 | 1.26 | up |
| HORVU.MOREX.r2.3HG0262520 | 212.00 | 211.00 | 265.00 | 684.00 | 624.00 | 648.00 | 2.73 | 2.70 | 3.56 | 8.72 | 7.75 | 8.70 | 0.00 | 1.41 | up |
| HORVU.MOREX.r2.5HG0416270 | 100.00 | 150.00 | 114.00 | 351.00 | 363.00 | 810.00 | 0.82 | 1.23 | 0.98 | 2.88 | 2.90 | 6.99 | 0.00 | 1.81 | up |
| HORVU.MOREX.r2.2HG0173210 | 448.00 | 726.00 | 500.00 | 1189.00 | 1267.00 | 1243.00 | 3.12 | 5.02 | 3.63 | 8.19 | 8.50 | 9.02 | 0.00 | 1.07 | up |
| HORVU.MOREX.r2.1HG0006510 | 2.00 | 52.00 | 19.00 | 933.00 | 671.00 | 486.00 | 0.02 | 0.48 | 0.19 | 8.78 | 6.15 | 4.82 | 0.00 | 2.10 | up |
| HORVU.MOREX.r2.2HG0169860 | 55.00 | 78.00 | 125.00 | 886.00 | 603.00 | 1071.00 | 0.95 | 1.34 | 2.25 | 15.17 | 10.08 | 19.34 | 0.00 | 2.93 | up |
| HORVU.MOREX.r2.5HG0353020 | 38.00 | 40.00 | 47.00 | 138.00 | 99.00 | 157.00 | 1.45 | 1.53 | 1.88 | 5.29 | 3.70 | 6.34 | 0.00 | 1.45 | up |
| HORVU.MOREX.r2.2HG0080890 | 3.00 | 7.00 | 6.00 | 178.00 | 172.00 | 168.00 | 0.03 | 0.08 | 0.07 | 1.99 | 1.86 | 1.97 | 0.00 | 4.21 | up |
| HORVU.MOREX.r2.5HG0412130 | 0.00 | 7.00 | 4.00 | 4732.00 | 2082.00 | 4448.00 | 0.00 | 0.08 | 0.05 | 59.08 | 25.34 | 58.50 | 0.00 | 4.84 | up |
| HORVU.MOREX.r2.2HG0155830 | 1338.00 | 2973.00 | 1209.00 | 3629.00 | 5795.00 | 4626.00 | 10.56 | 23.29 | 9.93 | 28.28 | 44.03 | 37.98 | 0.00 | 1.21 | up |
| HORVU.MOREX.r2.2HG0085110 | 8688.00 | 7587.00 | 6718.00 | 33051.00 | 23381.00 | 28064.00 | 33.22 | 28.80 | 26.74 | 124.80 | 86.07 | 111.66 | 0.00 | 1.78 | up |
| HORVU.MOREX.r2.6HG0517070 | 6.00 | 13.00 | 5.00 | 300.00 | 142.00 | 114.00 | 0.05 | 0.10 | 0.04 | 2.47 | 1.13 | 0.99 | 0.00 | 3.51 | up |
| HORVU.MOREX.r2.1HG0062750 | 39.00 | 24.00 | 13.00 | 701.00 | 238.00 | 376.00 | 1.29 | 0.77 | 0.43 | 23.24 | 7.69 | 13.12 | 0.00 | 3.34 | up |
| HORVU.MOREX.r2.7HG0538250 | 62.00 | 126.00 | 91.00 | 420.00 | 204.00 | 231.00 | 0.53 | 1.08 | 0.81 | 3.57 | 1.69 | 2.07 | 0.00 | 1.40 | up |
| HORVU.MOREX.r2.5HG0394670 | 302.00 | 475.00 | 371.00 | 1876.00 | 1542.00 | 2319.00 | 4.11 | 6.41 | 5.26 | 25.23 | 20.22 | 32.86 | 0.00 | 2.18 | up |
| HORVU.MOREX.r2.5HG0394580 | 697.00 | 891.00 | 631.00 | 1636.00 | 1747.00 | 2110.00 | 8.14 | 10.32 | 7.67 | 18.87 | 19.63 | 25.63 | 0.00 | 1.23 | up |
| HORVU.MOREX.r2.1HG0005050 | 34.00 | 50.00 | 62.00 | 163.00 | 199.00 | 261.00 | 1.68 | 2.48 | 3.22 | 8.03 | 9.56 | 13.58 | 0.00 | 1.84 | up |
| HORVU.MOREX.r2.6HG0493350 | 233.00 | 153.00 | 134.00 | 649.00 | 666.00 | 660.00 | 1.90 | 1.23 | 1.13 | 5.22 | 5.23 | 5.60 | 0.00 | 1.78 | up |
| HORVU.MOREX.r2.5HG0365720 | 16.00 | 13.00 | 8.00 | 106.00 | 80.00 | 105.00 | 0.24 | 0.19 | 0.13 | 1.67 | 1.22 | 1.74 | 0.00 | 2.49 | up |
| HORVU.MOREX.r2.3HG0183210 | 301.00 | 210.00 | 305.00 | 669.00 | 530.00 | 818.00 | 15.18 | 10.54 | 16.03 | 33.39 | 25.80 | 43.02 | 0.00 | 1.18 | up |
| HORVU.MOREX.r2.1HG0042360 | 707.00 | 554.00 | 497.00 | 1550.00 | 1064.00 | 1910.00 | 6.71 | 5.22 | 4.92 | 14.54 | 9.73 | 18.88 | 0.00 | 1.25 | up |
| HORVU.MOREX.r2.6HG0464870 | 23.00 | 64.00 | 26.00 | 441.00 | 283.00 | 322.00 | 0.65 | 1.86 | 0.78 | 12.77 | 7.99 | 9.82 | 0.00 | 2.78 | up |
| HORVU.MOREX.r2.6HG0449500 | 125.00 | 63.00 | 34.00 | 239.00 | 239.00 | 251.00 | 0.79 | 0.39 | 0.22 | 1.49 | 1.46 | 1.65 | 0.00 | 1.43 | up |
| HORVU.MOREX.r2.3HG0233680 | 205.00 | 144.00 | 89.00 | 797.00 | 754.00 | 940.00 | 2.44 | 1.70 | 1.09 | 9.37 | 8.64 | 11.65 | 0.00 | 2.29 | up |
| HORVU.MOREX.r2.1HG0014140 | 734.00 | 707.00 | 491.00 | 1361.00 | 1314.00 | 1544.00 | 24.52 | 23.46 | 17.07 | 44.91 | 42.28 | 53.70 | 0.00 | 1.05 | up |
| HORVU.MOREX.r2.3HG0212350 | 685.00 | 630.00 | 448.00 | 1183.00 | 1577.00 | 2117.00 | 9.47 | 8.66 | 6.45 | 16.17 | 21.02 | 30.49 | 0.00 | 1.35 | up |
| HORVU.MOREX.r2.1HG0068840 | 54.00 | 57.00 | 44.00 | 425.00 | 274.00 | 307.00 | 0.46 | 0.48 | 0.39 | 3.58 | 2.25 | 2.73 | 0.00 | 2.47 | up |
| HORVU.MOREX.r2.1HG0043930 | 1228.00 | 1215.00 | 1000.00 | 3280.00 | 2829.00 | 3911.00 | 17.58 | 17.28 | 14.90 | 46.40 | 39.01 | 58.29 | 0.00 | 1.46 | up |
| HORVU.MOREX.r2.2HG0140850 | 101.00 | 90.00 | 82.00 | 244.00 | 234.00 | 234.00 | 2.88 | 2.55 | 2.43 | 6.89 | 6.43 | 6.96 | 0.00 | 1.28 | up |
| HORVU.MOREX.r2.1HG0049960 | 48.00 | 105.00 | 70.00 | 156.00 | 165.00 | 185.00 | 0.55 | 1.20 | 0.84 | 1.78 | 1.83 | 2.23 | 0.00 | 1.04 | up |
| HORVU.MOREX.r2.2HG0174600 | 408.00 | 304.00 | 111.00 | 9201.00 | 5477.00 | 7050.00 | 9.37 | 6.93 | 2.64 | 208.87 | 121.20 | 168.62 | 0.00 | 3.59 | up |
| HORVU.MOREX.r2.3HG0249910 | 99.00 | 209.00 | 167.00 | 470.00 | 370.00 | 547.00 | 4.03 | 8.50 | 7.08 | 18.99 | 14.58 | 23.28 | 0.00 | 1.39 | up |
| HORVU.MOREX.r2.3HG0186950 | 368.00 | 330.00 | 369.00 | 1689.00 | 1622.00 | 2223.00 | 3.12 | 2.78 | 3.27 | 14.18 | 13.27 | 19.66 | 0.00 | 2.25 | up |
| HORVU.MOREX.r2.6HG0498110 | 115.00 | 75.00 | 63.00 | 346.00 | 369.00 | 369.00 | 1.93 | 1.24 | 1.10 | 5.75 | 5.97 | 6.47 | 0.00 | 1.92 | up |
| HORVU.MOREX.r2.5HG0432480 | 425.00 | 571.00 | 517.00 | 8694.00 | 3758.00 | 6677.00 | 1.73 | 2.31 | 2.19 | 34.94 | 14.72 | 28.27 | 0.00 | 3.36 | up |
| HORVU.MOREX.r2.7HG0605360 | 32.00 | 44.00 | 19.00 | 89.00 | 93.00 | 90.00 | 0.33 | 0.44 | 0.20 | 0.90 | 0.91 | 0.96 | 0.00 | 1.30 | up |
| HORVU.MOREX.r2.3HG0185370 | 227.00 | 123.00 | 110.00 | 315.00 | 334.00 | 387.00 | 6.61 | 3.56 | 3.33 | 9.08 | 9.37 | 11.76 | 0.00 | 1.03 | up |
| HORVU.MOREX.r2.7HG0607430 | 11252.00 | 12278.00 | 9583.00 | 31056.00 | 25276.00 | 27405.00 | 123.70 | 134.02 | 109.68 | 337.17 | 267.53 | 313.49 | 0.00 | 1.27 | up |
| HORVU.MOREX.r2.2HG0100250 | 620.00 | 806.00 | 766.00 | 1952.00 | 1463.00 | 1755.00 | 10.82 | 13.97 | 13.92 | 33.66 | 24.58 | 31.89 | 0.00 | 1.16 | up |
| HORVU.MOREX.r2.2HG0141810 | 3317.00 | 4252.00 | 3311.00 | 11500.00 | 8188.00 | 8750.00 | 21.15 | 26.92 | 21.98 | 72.42 | 50.27 | 58.06 | 0.00 | 1.31 | up |
| HORVU.MOREX.r2.7HG0545680 | 709.00 | 1263.00 | 767.00 | 2956.00 | 3292.00 | 3612.00 | 5.98 | 10.58 | 6.74 | 24.64 | 26.75 | 31.72 | 0.00 | 1.74 | up |
| HORVU.MOREX.r2.4HG0318370 | 1041.00 | 831.00 | 628.00 | 1924.00 | 1943.00 | 2269.00 | 9.70 | 7.68 | 6.09 | 17.70 | 17.43 | 22.00 | 0.00 | 1.21 | up |
| HORVU.MOREX.r2.1HG0073880 | 4.00 | 24.00 | 25.00 | 15276.00 | 13505.00 | 16023.00 | 0.05 | 0.36 | 0.39 | 235.21 | 202.72 | 259.95 | 0.00 | 6.50 | up |
| HORVU.MOREX.r2.5HG0359840 | 755.00 | 1540.00 | 955.00 | 3239.00 | 2272.00 | 2465.00 | 26.44 | 53.57 | 34.81 | 112.07 | 76.62 | 89.85 | 0.00 | 1.19 | up |
| HORVU.MOREX.r2.3HG0239130 | 106.00 | 41.00 | 39.00 | 494.00 | 426.00 | 420.00 | 0.98 | 0.37 | 0.37 | 4.55 | 3.82 | 4.07 | 0.00 | 2.46 | up |
| HORVU.MOREX.r2.1HG0006680 | 104.00 | 137.00 | 94.00 | 500.00 | 506.00 | 605.00 | 2.12 | 2.78 | 1.99 | 10.10 | 9.97 | 12.87 | 0.00 | 2.14 | up |
| HORVU.MOREX.r2.3HG0187140 | 284.00 | 193.00 | 149.00 | 1018.00 | 886.00 | 1041.00 | 5.92 | 3.99 | 3.23 | 20.96 | 17.78 | 22.56 | 0.00 | 2.07 | up |
| HORVU.MOREX.r2.1HG0000230 | 50.00 | 53.00 | 54.00 | 125.00 | 129.00 | 118.00 | 0.25 | 0.26 | 0.28 | 0.61 | 0.62 | 0.61 | 0.00 | 1.12 | up |
| HORVU.MOREX.r2.3HG0187960 | 33.00 | 24.00 | 11.00 | 270.00 | 155.00 | 242.00 | 0.28 | 0.20 | 0.09 | 2.30 | 1.28 | 2.17 | 0.00 | 2.78 | up |
| HORVU.MOREX.r2.3HG0202820 | 57.00 | 17.00 | 34.00 | 213.00 | 169.00 | 197.00 | 1.37 | 0.41 | 0.85 | 5.12 | 3.96 | 4.98 | 0.00 | 2.01 | up |
| HORVU.MOREX.r2.2HG0114340 | 1127.00 | 1366.00 | 1002.00 | 2973.00 | 2520.00 | 2957.00 | 9.49 | 11.41 | 8.77 | 24.71 | 20.41 | 25.89 | 0.00 | 1.21 | up |
| HORVU.MOREX.r2.4HG0280110 | 59.00 | 97.00 | 53.00 | 345.00 | 242.00 | 248.00 | 1.25 | 2.04 | 1.17 | 7.24 | 4.96 | 5.48 | 0.00 | 1.81 | up |
| HORVU.MOREX.r2.2HG0103420 | 497.00 | 583.00 | 355.00 | 980.00 | 888.00 | 1352.00 | 5.42 | 6.32 | 4.04 | 10.58 | 9.34 | 15.38 | 0.00 | 1.08 | up |
| HORVU.MOREX.r2.6HG0476760 | 642.00 | 338.00 | 389.00 | 4791.00 | 3421.00 | 3985.00 | 3.01 | 1.57 | 1.90 | 22.21 | 15.46 | 19.46 | 0.00 | 2.92 | up |
| HORVU.MOREX.r2.5HG0385340 | 4568.00 | 2561.00 | 2851.00 | 10059.00 | 6937.00 | 6783.00 | 104.18 | 57.98 | 67.68 | 226.55 | 152.31 | 160.96 | 0.00 | 1.13 | up |
| HORVU.MOREX.r2.6HG0517560 | 77.00 | 133.00 | 89.00 | 147.00 | 234.00 | 324.00 | 0.93 | 1.61 | 1.13 | 1.77 | 2.75 | 4.12 | 0.00 | 1.07 | up |
| HORVU.MOREX.r2.2HG0173190 | 9.00 | 24.00 | 3.00 | 88.00 | 60.00 | 70.00 | 0.08 | 0.21 | 0.02 | 0.76 | 0.51 | 0.64 | 0.00 | 1.86 | up |
| HORVU.MOREX.r2.7HG0582010 | 1761.00 | 1288.00 | 899.00 | 9222.00 | 6444.00 | 7668.00 | 17.75 | 12.88 | 9.43 | 91.79 | 62.53 | 80.41 | 0.00 | 2.38 | up |
| HORVU.MOREX.r2.5HG0401310 | 1237.00 | 2076.00 | 2507.00 | 5292.00 | 3707.00 | 4743.00 | 39.31 | 65.48 | 82.93 | 166.09 | 113.40 | 156.83 | 0.00 | 1.12 | up |
| HORVU.MOREX.r2.7HG0590950 | 137.00 | 167.00 | 85.00 | 664.00 | 370.00 | 327.00 | 3.50 | 4.24 | 2.26 | 16.79 | 9.12 | 8.71 | 0.00 | 1.59 | up |
| HORVU.MOREX.r2.2HG0097430 | 200.00 | 260.00 | 182.00 | 719.00 | 479.00 | 574.00 | 1.23 | 1.59 | 1.16 | 4.38 | 2.84 | 3.68 | 0.00 | 1.36 | up |
| HORVU.MOREX.r2.5HG0402660 | 3807.00 | 4352.00 | 3682.00 | 13118.00 | 10986.00 | 12446.00 | 63.43 | 72.00 | 63.87 | 215.87 | 176.24 | 215.80 | 0.00 | 1.56 | up |
| HORVU.MOREX.r2.4HG0284370 | 570.00 | 502.00 | 484.00 | 1074.00 | 991.00 | 1242.00 | 8.86 | 7.74 | 7.82 | 16.49 | 14.83 | 20.08 | 0.00 | 1.02 | up |
| HORVU.MOREX.r2.7HG0597730 | 245.00 | 234.00 | 185.00 | 730.00 | 545.00 | 684.00 | 3.10 | 2.94 | 2.44 | 9.15 | 6.66 | 9.04 | 0.00 | 1.46 | up |
| HORVU.MOREX.r2.1HG0017380 | 7485.00 | 16107.00 | 13568.00 | 24171.00 | 29000.00 | 28933.00 | 140.88 | 301.00 | 265.86 | 449.27 | 525.50 | 566.61 | 0.00 | 1.04 | up |
| HORVU.MOREX.r2.1HG0033820 | 689.00 | 484.00 | 378.00 | 2271.00 | 2035.00 | 3081.00 | 11.12 | 7.76 | 6.36 | 36.23 | 31.64 | 51.78 | 0.00 | 2.08 | up |
| HORVU.MOREX.r2.1HG0023550 | 348.00 | 303.00 | 224.00 | 1446.00 | 1283.00 | 1490.00 | 4.35 | 3.76 | 2.92 | 17.89 | 15.47 | 19.42 | 0.00 | 2.15 | up |
| HORVU.MOREX.r2.3HG0266050 | 206.00 | 150.00 | 338.00 | 3577.00 | 2541.00 | 6874.00 | 3.39 | 2.45 | 5.78 | 58.19 | 40.30 | 117.84 | 0.00 | 3.06 | up |
| HORVU.MOREX.r2.4HG0320280 | 1352.00 | 568.00 | 427.00 | 3464.00 | 2291.00 | 4098.00 | 21.18 | 8.83 | 6.97 | 53.63 | 34.57 | 66.83 | 0.00 | 1.54 | up |
| HORVU.MOREX.r2.4HG0339680 | 49.00 | 26.00 | 15.00 | 211.00 | 309.00 | 299.00 | 1.37 | 0.71 | 0.43 | 5.83 | 8.35 | 8.73 | 0.00 | 2.71 | up |
| HORVU.MOREX.r2.5HG0367890 | 291.00 | 626.00 | 366.00 | 1202.00 | 1056.00 | 1260.00 | 10.13 | 21.62 | 13.27 | 41.31 | 35.40 | 45.65 | 0.00 | 1.34 | up |
| HORVU.MOREX.r2.5HG0441650 | 182.00 | 269.00 | 285.00 | 1112.00 | 1230.00 | 1478.00 | 2.49 | 3.67 | 4.07 | 15.10 | 16.27 | 21.14 | 0.00 | 2.23 | up |
| HORVU.MOREX.r2.6HG0476090 | 142.00 | 137.00 | 87.00 | 982.00 | 770.00 | 1175.00 | 4.29 | 4.09 | 2.72 | 29.36 | 22.44 | 37.02 | 0.00 | 2.79 | up |
| HORVU.MOREX.r2.5HG0384710 | 661.00 | 519.00 | 385.00 | 2303.00 | 1314.00 | 1277.00 | 8.07 | 6.29 | 4.89 | 27.78 | 15.46 | 16.23 | 0.00 | 1.49 | up |
| HORVU.MOREX.r2.1HG0073960 | 0.00 | 0.00 | 0.00 | 404.00 | 289.00 | 360.00 | 0.00 | 0.00 | 0.00 | 6.42 | 4.47 | 6.02 | 0.00 | 6.39 | up |
| HORVU.MOREX.r2.7HG0592710 | 1506.00 | 797.00 | 877.00 | 2513.00 | 2035.00 | 2760.00 | 20.18 | 10.60 | 12.23 | 33.25 | 26.25 | 38.48 | 0.00 | 1.08 | up |
| HORVU.MOREX.r2.4HG0315940 | 450.00 | 457.00 | 250.00 | 1739.00 | 1086.00 | 1498.00 | 2.17 | 2.19 | 1.25 | 8.30 | 5.05 | 7.53 | 0.00 | 1.75 | up |
| HORVU.MOREX.r2.2HG0110940 | 15.00 | 0.00 | 5.00 | 2125.00 | 1029.00 | 2776.00 | 0.15 | 0.00 | 0.04 | 21.79 | 10.28 | 29.99 | 0.00 | 2.03 | up |
| HORVU.MOREX.r2.4HG0283700 | 14.00 | 55.00 | 43.00 | 258.00 | 132.00 | 261.00 | 0.97 | 3.83 | 3.16 | 18.05 | 8.99 | 19.24 | 0.00 | 2.04 | up |
| HORVU.MOREX.r2.3HG0184800 | 13.00 | 15.00 | 3.00 | 608.00 | 504.00 | 525.00 | 0.21 | 0.24 | 0.05 | 9.91 | 8.01 | 9.01 | 0.00 | 5.11 | up |
| HORVU.MOREX.r2.2HG0171870 | 8.00 | 3.00 | 75.00 | 497.00 | 945.00 | 1254.00 | 0.05 | 0.02 | 0.51 | 3.23 | 5.99 | 8.59 | 0.01 | 1.61 | up |
| HORVU.MOREX.r2.4HG0283690 | 99.00 | 62.00 | 31.00 | 230.00 | 139.00 | 172.00 | 7.17 | 4.42 | 2.28 | 16.50 | 9.70 | 12.95 | 0.00 | 1.24 | up |
| HORVU.MOREX.r2.7HG0534330 | 1.00 | 2.00 | 4.00 | 748.00 | 189.00 | 542.00 | 0.02 | 0.03 | 0.10 | 17.82 | 4.37 | 13.60 | 0.00 | 5.39 | up |
| HORVU.MOREX.r2.6HG0457730 | 21004.00 | 18645.00 | 11728.00 | 74366.00 | 67815.00 | 92417.00 | 578.96 | 510.25 | 336.54 | 2024.23 | 1799.58 | 2650.51 | 0.00 | 2.06 | up |
| HORVU.MOREX.r2.3HG0199680 | 561.00 | 445.00 | 349.00 | 6154.00 | 4548.00 | 5474.00 | 6.56 | 5.17 | 4.25 | 71.11 | 51.24 | 66.64 | 0.00 | 3.40 | up |
| HORVU.MOREX.r2.3HG0196880 | 73.00 | 132.00 | 86.00 | 1701.00 | 1668.00 | 1931.00 | 1.83 | 3.31 | 2.26 | 42.47 | 40.60 | 50.78 | 0.00 | 3.97 | up |
| HORVU.MOREX.r2.1HG0055410 | 610.00 | 831.00 | 522.00 | 2111.00 | 1599.00 | 1777.00 | 3.39 | 4.59 | 3.02 | 11.60 | 8.57 | 10.29 | 0.00 | 1.40 | up |
| HORVU.MOREX.r2.2HG0084610 | 1584.00 | 2296.00 | 1650.00 | 4336.00 | 4050.00 | 4617.00 | 11.24 | 16.18 | 12.20 | 30.40 | 27.69 | 34.11 | 0.00 | 1.17 | up |
| HORVU.MOREX.r2.6HG0513160 | 776.00 | 560.00 | 321.00 | 1975.00 | 2149.00 | 1870.00 | 5.06 | 3.62 | 2.17 | 12.71 | 13.48 | 12.68 | 0.00 | 1.69 | up |
| HORVU.MOREX.r2.6HG0460850 | 355.00 | 559.00 | 201.00 | 62875.00 | 31052.00 | 47057.00 | 3.95 | 6.18 | 2.33 | 691.80 | 333.07 | 545.52 | 0.00 | 5.66 | up |
| HORVU.MOREX.r2.2HG0166910 | 4161.00 | 3898.00 | 2343.00 | 17313.00 | 11467.00 | 17532.00 | 47.35 | 44.04 | 27.76 | 194.58 | 125.64 | 207.62 | 0.00 | 2.00 | up |
| HORVU.MOREX.r2.7HG0560320 | 585.00 | 243.00 | 219.00 | 1315.00 | 634.00 | 853.00 | 27.84 | 11.50 | 10.85 | 61.88 | 29.06 | 42.29 | 0.00 | 1.19 | up |
| HORVU.MOREX.r2.1HG0057270 | 42.00 | 23.00 | 21.00 | 286.00 | 196.00 | 217.00 | 3.52 | 1.88 | 1.78 | 23.80 | 15.88 | 18.97 | 0.00 | 2.65 | up |
| HORVU.MOREX.r2.5HG0409540 | 29.00 | 34.00 | 52.00 | 105.00 | 105.00 | 78.00 | 0.33 | 0.39 | 0.62 | 1.20 | 1.17 | 0.94 | 0.00 | 1.12 | up |
| HORVU.MOREX.r2.4HG0338450 | 125.00 | 136.00 | 102.00 | 1290.00 | 625.00 | 798.00 | 6.29 | 6.78 | 5.32 | 64.40 | 30.39 | 41.94 | 0.00 | 2.65 | up |
| HORVU.MOREX.r2.5HG0445920 | 268.00 | 458.00 | 360.00 | 1745.00 | 1403.00 | 2005.00 | 2.26 | 3.84 | 3.16 | 14.56 | 11.41 | 17.63 | 0.00 | 2.10 | up |
| Hordeum_vulgare_newGene_740 | 201.00 | 142.00 | 112.00 | 400.00 | 347.00 | 655.00 | 3.03 | 2.11 | 1.74 | 5.93 | 5.02 | 10.25 | 0.00 | 1.44 | up |
| HORVU.MOREX.r2.2HG0106450 | 202.00 | 153.00 | 152.00 | 1596.00 | 951.00 | 1114.00 | 3.97 | 2.98 | 3.11 | 31.06 | 18.05 | 22.83 | 0.00 | 2.65 | up |
| HORVU.MOREX.r2.3HG0267870 | 1207.00 | 1277.00 | 1033.00 | 2730.00 | 2186.00 | 2486.00 | 6.88 | 7.23 | 6.13 | 15.37 | 12.00 | 14.75 | 0.00 | 1.01 | up |
| HORVU.MOREX.r2.1HG0078620 | 3.00 | 6.00 | 4.00 | 93.00 | 62.00 | 110.00 | 0.04 | 0.09 | 0.06 | 1.35 | 0.87 | 1.68 | 0.00 | 3.28 | up |
| HORVU.MOREX.r2.1HG0059600 | 34.00 | 8.00 | 18.00 | 101.00 | 67.00 | 108.00 | 0.54 | 0.12 | 0.29 | 1.59 | 1.02 | 1.79 | 0.00 | 1.67 | up |
| HORVU.MOREX.r2.3HG0187060 | 16.00 | 33.00 | 25.00 | 79.00 | 77.00 | 76.00 | 0.36 | 0.74 | 0.57 | 1.76 | 1.67 | 1.80 | 0.00 | 1.40 | up |
| HORVU.MOREX.r2.2HG0147250 | 11187.00 | 8062.00 | 7046.00 | 50653.00 | 42035.00 | 51128.00 | 235.73 | 168.67 | 154.56 | 1054.06 | 852.77 | 1121.01 | 0.00 | 2.32 | up |
| HORVU.MOREX.r2.5HG0403530 | 45.00 | 47.00 | 47.00 | 166.00 | 118.00 | 94.00 | 0.64 | 0.67 | 0.69 | 2.34 | 1.63 | 1.39 | 0.00 | 1.26 | up |
| HORVU.MOREX.r2.7HG0538280 | 0.00 | 28.00 | 21.00 | 1434.00 | 831.00 | 854.00 | 0.00 | 0.31 | 0.24 | 15.93 | 9.00 | 9.99 | 0.01 | 1.60 | up |
| HORVU.MOREX.r2.2HG0113060 | 13.00 | 24.00 | 15.00 | 113.00 | 99.00 | 117.00 | 0.11 | 0.19 | 0.12 | 0.91 | 0.78 | 1.00 | 0.00 | 2.30 | up |
| HORVU.MOREX.r2.5HG0425570 | 4780.00 | 3351.00 | 3012.00 | 40877.00 | 21785.00 | 33941.00 | 53.56 | 37.28 | 35.13 | 452.35 | 235.02 | 395.74 | 0.00 | 2.89 | up |
| HORVU.MOREX.r2.3HG0238330 | 90.00 | 46.00 | 21.00 | 199.00 | 207.00 | 243.00 | 0.82 | 0.41 | 0.19 | 1.78 | 1.81 | 2.30 | 0.00 | 1.67 | up |
| HORVU.MOREX.r2.7HG0611150 | 135.00 | 71.00 | 35.00 | 337.00 | 149.00 | 249.00 | 3.12 | 1.63 | 0.84 | 7.74 | 3.33 | 6.02 | 0.01 | 1.28 | up |
| HORVU.MOREX.r2.2HG0090200 | 16.00 | 6.00 | 10.00 | 75.00 | 62.00 | 54.00 | 0.57 | 0.19 | 0.37 | 2.70 | 2.15 | 2.05 | 0.00 | 2.01 | up |
| HORVU.MOREX.r2.2HG0154130 | 179.00 | 57.00 | 83.00 | 4080.00 | 3100.00 | 4647.00 | 2.97 | 0.94 | 1.44 | 66.94 | 49.58 | 80.34 | 0.00 | 3.96 | up |
| Hordeum_vulgare_newGene_2797 | 1779.00 | 1530.00 | 1454.00 | 5767.00 | 6936.00 | 9063.00 | 78.98 | 67.44 | 67.19 | 252.81 | 296.43 | 418.65 | 0.00 | 2.07 | up |
| HORVU.MOREX.r2.3HG0246670 | 2805.00 | 2656.00 | 2297.00 | 16452.00 | 11475.00 | 15631.00 | 47.15 | 44.32 | 40.19 | 273.08 | 185.68 | 273.38 | 0.00 | 2.38 | up |
| HORVU.MOREX.r2.2HG0144420 | 20.00 | 0.00 | 11.00 | 115.00 | 130.00 | 144.00 | 0.16 | 0.00 | 0.09 | 0.89 | 0.98 | 1.18 | 0.00 | 2.36 | up |
| HORVU.MOREX.r2.5HG0416510 | 235.00 | 131.00 | 82.00 | 671.00 | 391.00 | 646.00 | 2.12 | 1.17 | 0.76 | 5.98 | 3.40 | 6.07 | 0.00 | 1.66 | up |
| HORVU.MOREX.r2.1HG0018770 | 0.00 | 2.00 | 13.00 | 592.00 | 316.00 | 473.00 | 0.00 | 0.02 | 0.15 | 6.41 | 3.33 | 5.40 | 0.00 | 1.87 | up |
| HORVU.MOREX.r2.3HG0252970 | 26.00 | 6.00 | 5.00 | 78.00 | 101.00 | 127.00 | 1.49 | 0.29 | 0.28 | 4.39 | 5.59 | 7.58 | 0.00 | 2.18 | up |
| HORVU.MOREX.r2.2HG0177710 | 22.00 | 26.00 | 13.00 | 94.00 | 68.00 | 174.00 | 0.27 | 0.32 | 0.17 | 1.18 | 0.83 | 2.31 | 0.00 | 1.96 | up |
| HORVU.MOREX.r2.3HG0257110 | 652.00 | 612.00 | 634.00 | 1582.00 | 1380.00 | 1578.00 | 15.22 | 14.19 | 15.41 | 36.47 | 31.03 | 38.33 | 0.00 | 1.19 | up |
| HORVU.MOREX.r2.1HG0047370 | 644.00 | 267.00 | 337.00 | 6674.00 | 3983.00 | 5967.00 | 8.47 | 3.48 | 4.61 | 86.72 | 50.45 | 81.69 | 0.00 | 3.34 | up |
| HORVU.MOREX.r2.3HG0203720 | 1634.00 | 2398.00 | 1446.00 | 7142.00 | 5558.00 | 6818.00 | 25.48 | 37.12 | 23.47 | 109.96 | 83.43 | 110.60 | 0.00 | 1.73 | up |
| HORVU.MOREX.r2.4HG0335810 | 1416.00 | 2742.00 | 2369.00 | 8623.00 | 5692.00 | 7917.00 | 18.88 | 36.32 | 32.90 | 113.59 | 73.09 | 109.88 | 0.00 | 1.63 | up |
| HORVU.MOREX.r2.4HG0332840 | 70.00 | 111.00 | 84.00 | 202.00 | 178.00 | 208.00 | 2.15 | 3.41 | 2.67 | 6.16 | 5.29 | 6.67 | 0.00 | 1.04 | up |
| HORVU.MOREX.r2.7HG0544910 | 57.00 | 55.00 | 53.00 | 211.00 | 123.00 | 146.00 | 1.32 | 1.27 | 1.29 | 4.90 | 2.78 | 3.56 | 0.00 | 1.36 | up |
| HORVU.MOREX.r2.5HG0400740 | 17.00 | 20.00 | 0.00 | 1123.00 | 853.00 | 1080.00 | 0.29 | 0.34 | 0.00 | 19.42 | 14.37 | 19.68 | 0.00 | 2.01 | up |
| HORVU.MOREX.r2.2HG0152270 | 6.00 | 14.00 | 5.00 | 134.00 | 86.00 | 153.00 | 0.11 | 0.26 | 0.09 | 2.49 | 1.55 | 3.02 | 0.00 | 3.11 | up |
| HORVU.MOREX.r2.5HG0392230 | 1619.00 | 1671.00 | 1847.00 | 157037.00 | 112434.00 | 126162.00 | 57.06 | 58.45 | 67.78 | 5466.26 | 3815.44 | 4627.05 | 0.00 | 6.06 | up |
| HORVU.MOREX.r2.3HG0202130 | 278.00 | 543.00 | 343.00 | 2889.00 | 1650.00 | 2452.00 | 2.02 | 3.92 | 2.59 | 20.74 | 11.55 | 18.54 | 0.00 | 2.38 | up |
| HORVU.MOREX.r2.3HG0232780 | 58.00 | 45.00 | 50.00 | 228.00 | 236.00 | 211.00 | 1.13 | 0.87 | 1.01 | 4.41 | 4.44 | 4.29 | 0.00 | 1.98 | up |
| HORVU.MOREX.r2.5HG0380330 | 48.00 | 59.00 | 65.00 | 242.00 | 220.00 | 260.00 | 0.51 | 0.62 | 0.72 | 2.54 | 2.25 | 2.87 | 0.00 | 1.91 | up |
| HORVU.MOREX.r2.3HG0200400 | 4961.00 | 4217.00 | 3190.00 | 20303.00 | 14861.00 | 19464.00 | 19.21 | 16.21 | 12.86 | 77.63 | 55.40 | 78.42 | 0.00 | 2.03 | up |
| HORVU.MOREX.r2.5HG0439630 | 1247.00 | 928.00 | 525.00 | 6842.00 | 4827.00 | 7503.00 | 21.27 | 15.70 | 9.31 | 115.24 | 79.26 | 133.13 | 0.00 | 2.58 | up |
| HORVU.MOREX.r2.4HG0328590 | 1786.00 | 1491.00 | 1132.00 | 3513.00 | 5332.00 | 10870.00 | 21.59 | 17.89 | 14.24 | 41.94 | 62.06 | 136.75 | 0.00 | 1.65 | up |
| HORVU.MOREX.r2.6HG0496060 | 139.00 | 273.00 | 196.00 | 651.00 | 640.00 | 688.00 | 2.84 | 5.54 | 4.16 | 13.15 | 12.60 | 14.64 | 0.00 | 1.58 | up |
| HORVU.MOREX.r2.2HG0104330 | 1001.00 | 757.00 | 498.00 | 2438.00 | 1679.00 | 2064.00 | 16.21 | 12.17 | 8.40 | 38.99 | 26.17 | 34.78 | 0.00 | 1.33 | up |
| HORVU.MOREX.r2.7HG0595390 | 32.00 | 71.00 | 40.00 | 251.00 | 239.00 | 212.00 | 0.60 | 1.34 | 0.78 | 4.73 | 4.40 | 4.21 | 0.00 | 2.06 | up |
| HORVU.MOREX.r2.5HG0412080 | 76.00 | 81.00 | 107.00 | 378.00 | 284.00 | 311.00 | 0.46 | 0.49 | 0.68 | 2.29 | 1.68 | 1.98 | 0.00 | 1.72 | up |
| HORVU.MOREX.r2.2HG0173530 | 1004.00 | 1420.00 | 1004.00 | 3320.00 | 2646.00 | 3026.00 | 35.80 | 50.30 | 37.30 | 116.97 | 90.87 | 112.33 | 0.00 | 1.32 | up |
| HORVU.MOREX.r2.1HG0070500 | 21.00 | 27.00 | 4.00 | 2343.00 | 1161.00 | 1668.00 | 0.25 | 0.32 | 0.05 | 28.69 | 13.86 | 21.52 | 0.00 | 4.29 | up |
| HORVU.MOREX.r2.6HG0524260 | 41.00 | 44.00 | 75.00 | 342.00 | 549.00 | 627.00 | 0.32 | 0.34 | 0.61 | 2.67 | 4.18 | 5.16 | 0.00 | 2.87 | up |
| HORVU.MOREX.r2.2HG0112550 | 150.00 | 113.00 | 92.00 | 169.00 | 299.00 | 603.00 | 1.00 | 0.75 | 0.64 | 1.11 | 1.92 | 4.19 | 0.00 | 1.31 | up |
| HORVU.MOREX.r2.2HG0106100 | 570.00 | 550.00 | 438.00 | 1511.00 | 1001.00 | 1205.00 | 2.95 | 2.83 | 2.36 | 7.73 | 4.99 | 6.50 | 0.00 | 1.17 | up |
| HORVU.MOREX.r2.3HG0203880 | 38.00 | 55.00 | 24.00 | 148.00 | 164.00 | 124.00 | 1.01 | 1.46 | 0.67 | 3.95 | 4.25 | 3.48 | 0.00 | 1.67 | up |
| HORVU.MOREX.r2.4HG0298980 | 15.00 | 28.00 | 6.00 | 266.00 | 84.00 | 128.00 | 0.13 | 0.24 | 0.05 | 2.30 | 0.70 | 1.16 | 0.00 | 2.44 | up |
| HORVU.MOREX.r2.3HG0183450 | 127.00 | 118.00 | 87.00 | 843.00 | 392.00 | 554.00 | 1.12 | 1.03 | 0.80 | 7.40 | 3.35 | 5.12 | 0.00 | 2.18 | up |
| HORVU.MOREX.r2.3HG0234550 | 39.00 | 4.00 | 33.00 | 533.00 | 282.00 | 340.00 | 0.49 | 0.05 | 0.43 | 6.68 | 3.45 | 4.49 | 0.00 | 2.93 | up |
| HORVU.MOREX.r2.1HG0011940 | 405.00 | 339.00 | 199.00 | 909.00 | 794.00 | 850.00 | 5.44 | 4.52 | 2.79 | 12.08 | 10.28 | 11.90 | 0.00 | 1.32 | up |
| HORVU.MOREX.r2.4HG0329890 | 663.00 | 1081.00 | 461.00 | 2717.00 | 2263.00 | 2661.00 | 10.28 | 16.64 | 7.44 | 41.61 | 33.79 | 42.93 | 0.00 | 1.66 | up |
| HORVU.MOREX.r2.6HG0503700 | 76.00 | 139.00 | 176.00 | 243.00 | 246.00 | 406.00 | 1.25 | 2.25 | 3.01 | 3.94 | 3.88 | 6.93 | 0.01 | 1.02 | up |
| HORVU.MOREX.r2.5HG0379280 | 165.00 | 165.00 | 128.00 | 382.00 | 314.00 | 355.00 | 4.69 | 4.66 | 3.78 | 10.73 | 8.62 | 10.50 | 0.00 | 1.11 | up |
| HORVU.MOREX.r2.5HG0427370 | 19.00 | 38.00 | 23.00 | 3853.00 | 1871.00 | 2201.00 | 0.32 | 0.63 | 0.39 | 63.59 | 30.10 | 38.26 | 0.00 | 5.95 | up |
| HORVU.MOREX.r2.7HG0585160 | 33.00 | 99.00 | 75.00 | 275.00 | 205.00 | 233.00 | 0.40 | 1.20 | 0.95 | 3.34 | 2.43 | 2.98 | 0.00 | 1.54 | up |
| HORVU.MOREX.r2.2HG0176420 | 393.00 | 431.00 | 521.00 | 2433.00 | 1403.00 | 1544.00 | 4.81 | 5.24 | 6.65 | 29.48 | 16.56 | 19.70 | 0.00 | 1.84 | up |
| HORVU.MOREX.r2.1HG0000640 | 289.00 | 280.00 | 337.00 | 659.00 | 716.00 | 1098.00 | 1.60 | 1.54 | 1.95 | 3.62 | 3.83 | 6.35 | 0.00 | 1.33 | up |
| HORVU.MOREX.r2.2HG0146170 | 1916.00 | 1749.00 | 1343.00 | 4703.00 | 4030.00 | 4361.00 | 21.85 | 19.80 | 15.94 | 52.96 | 44.24 | 51.74 | 0.00 | 1.31 | up |
| HORVU.MOREX.r2.4HG0344600 | 552.00 | 533.00 | 296.00 | 1413.00 | 1330.00 | 1670.00 | 9.21 | 8.83 | 5.15 | 23.32 | 21.39 | 29.03 | 0.00 | 1.55 | up |
| HORVU.MOREX.r2.UnG0632130 | 29.00 | 45.00 | 38.00 | 372.00 | 160.00 | 418.00 | 0.36 | 0.57 | 0.50 | 4.67 | 1.96 | 5.53 | 0.00 | 2.63 | up |
| HORVU.MOREX.r2.6HG0451730 | 1169.00 | 1640.00 | 1256.00 | 4113.00 | 2779.00 | 3761.00 | 29.67 | 41.33 | 33.19 | 103.14 | 67.95 | 99.38 | 0.00 | 1.30 | up |
| HORVU.MOREX.r2.2HG0095190 | 36.00 | 38.00 | 13.00 | 184.00 | 126.00 | 158.00 | 1.30 | 1.36 | 0.50 | 6.71 | 4.46 | 6.05 | 0.00 | 2.05 | up |
| HORVU.MOREX.r2.7HG0529590 | 360.00 | 337.00 | 287.00 | 1015.00 | 861.00 | 2449.00 | 3.39 | 3.15 | 2.81 | 9.43 | 7.80 | 23.98 | 0.00 | 1.87 | up |
| HORVU.MOREX.r2.2HG0168670 | 314.00 | 615.00 | 470.00 | 944.00 | 1218.00 | 1175.00 | 3.92 | 7.64 | 6.12 | 11.66 | 14.66 | 15.28 | 0.00 | 1.15 | up |
| HORVU.MOREX.r2.5HG0392950 | 112.00 | 178.00 | 173.00 | 1336.00 | 1309.00 | 1528.00 | 0.72 | 1.13 | 1.16 | 8.50 | 8.12 | 10.24 | 0.00 | 3.00 | up |
| HORVU.MOREX.r2.2HG0166600 | 4043.00 | 2866.00 | 2167.00 | 6648.00 | 6770.00 | 6933.00 | 80.24 | 56.47 | 44.76 | 130.30 | 129.37 | 143.18 | 0.00 | 1.07 | up |
| HORVU.MOREX.r2.7HG0618780 | 1463.00 | 1141.00 | 883.00 | 7188.00 | 6275.00 | 7495.00 | 16.17 | 12.53 | 10.16 | 78.48 | 66.79 | 86.22 | 0.00 | 2.45 | up |
| HORVU.MOREX.r2.3HG0243670 | 2.00 | 0.00 | 0.00 | 180.00 | 55.00 | 131.00 | 0.01 | 0.00 | 0.00 | 1.51 | 0.45 | 1.16 | 0.00 | 4.17 | up |
| HORVU.MOREX.r2.4HG0288710 | 17.00 | 13.00 | 0.00 | 89.00 | 150.00 | 124.00 | 0.29 | 0.23 | 0.00 | 1.54 | 2.53 | 2.25 | 0.00 | 2.40 | up |
| Hordeum_vulgare_newGene_1437 | 82.00 | 117.00 | 125.00 | 311.00 | 217.00 | 213.00 | 0.93 | 1.32 | 1.48 | 3.50 | 2.38 | 2.53 | 0.00 | 1.07 | up |
| HORVU.MOREX.r2.3HG0268890 | 48.00 | 76.00 | 64.00 | 465.00 | 142.00 | 288.00 | 1.82 | 2.86 | 2.53 | 17.43 | 5.19 | 11.36 | 0.00 | 1.88 | up |
| HORVU.MOREX.r2.6HG0504120 | 78.00 | 377.00 | 334.00 | 3503.00 | 3058.00 | 5302.00 | 1.34 | 6.45 | 5.99 | 59.69 | 50.80 | 95.19 | 0.00 | 2.65 | up |
| HORVU.MOREX.r2.1HG0001410 | 59.00 | 78.00 | 53.00 | 630.00 | 412.00 | 796.00 | 0.49 | 0.64 | 0.46 | 5.21 | 3.33 | 6.95 | 0.00 | 2.97 | up |
| HORVU.MOREX.r2.1HG0067180 | 140.00 | 116.00 | 68.00 | 472.00 | 323.00 | 468.00 | 5.85 | 4.81 | 2.93 | 19.50 | 13.00 | 20.36 | 0.00 | 1.77 | up |
| HORVU.MOREX.r2.3HG0269080 | 181.00 | 118.00 | 32.00 | 974.00 | 504.00 | 635.00 | 1.46 | 0.94 | 0.26 | 7.77 | 3.92 | 5.33 | 0.00 | 1.78 | up |
| HORVU.MOREX.r2.1HG0013750 | 18127.00 | 11859.00 | 10349.00 | 35118.00 | 28510.00 | 32919.00 | 479.21 | 311.26 | 284.83 | 916.81 | 725.61 | 905.49 | 0.00 | 1.16 | up |
| HORVU.MOREX.r2.5HG0397800 | 33.00 | 6.00 | 14.00 | 228.00 | 167.00 | 158.00 | 0.31 | 0.05 | 0.14 | 2.12 | 1.51 | 1.55 | 0.00 | 2.64 | up |
| HORVU.MOREX.r2.7HG0602430 | 0.00 | 0.00 | 0.00 | 176.00 | 82.00 | 190.00 | 0.00 | 0.00 | 0.00 | 2.14 | 0.97 | 2.44 | 0.00 | 4.92 | up |
| HORVU.MOREX.r2.3HG0184560 | 90.00 | 114.00 | 72.00 | 478.00 | 216.00 | 270.00 | 2.23 | 2.79 | 1.86 | 11.72 | 5.16 | 6.97 | 0.00 | 1.58 | up |
| HORVU.MOREX.r2.2HG0141860 | 2614.00 | 1330.00 | 992.00 | 3506.00 | 3615.00 | 4751.00 | 46.48 | 23.47 | 18.37 | 61.58 | 61.89 | 87.92 | 0.00 | 1.11 | up |
| HORVU.MOREX.r2.1HG0001590 | 16.00 | 56.00 | 37.00 | 280.00 | 227.00 | 264.00 | 0.19 | 0.64 | 0.44 | 3.21 | 2.55 | 3.20 | 0.00 | 2.43 | up |
| HORVU.MOREX.r2.1HG0073870 | 2.00 | 0.00 | 0.00 | 208.00 | 202.00 | 280.00 | 0.02 | 0.00 | 0.00 | 2.39 | 2.26 | 3.39 | 0.00 | 5.57 | up |
| HORVU.MOREX.r2.3HG0186310 | 923.00 | 807.00 | 550.00 | 6509.00 | 4456.00 | 6146.00 | 13.82 | 12.00 | 8.58 | 96.34 | 64.29 | 95.86 | 0.00 | 2.74 | up |
| HORVU.MOREX.r2.5HG0393730 | 553.00 | 526.00 | 643.00 | 1351.00 | 1325.00 | 1375.00 | 3.95 | 3.72 | 4.77 | 9.52 | 9.10 | 10.20 | 0.00 | 1.16 | up |
| HORVU.MOREX.r2.4HG0280140 | 217.00 | 98.00 | 119.00 | 822.00 | 523.00 | 586.00 | 4.59 | 2.04 | 2.61 | 17.15 | 10.65 | 12.89 | 0.00 | 1.90 | up |
| Hordeum_vulgare_newGene_6923 | 28.00 | 45.00 | 54.00 | 140.00 | 158.00 | 190.00 | 0.15 | 0.25 | 0.31 | 0.76 | 0.84 | 1.09 | 0.00 | 1.70 | up |
| HORVU.MOREX.r2.2HG0132080 | 329.00 | 221.00 | 243.00 | 2167.00 | 1242.00 | 1459.00 | 3.08 | 2.05 | 2.37 | 20.07 | 11.21 | 14.23 | 0.00 | 2.41 | up |
| HORVU.MOREX.r2.3HG0266280 | 15330.00 | 6870.00 | 3992.00 | 107709.00 | 92212.00 | 138354.00 | 263.00 | 117.02 | 71.29 | 1824.83 | 1523.06 | 2469.74 | 0.00 | 2.66 | up |
| HORVU.MOREX.r2.4HG0297260 | 87.00 | 108.00 | 97.00 | 558.00 | 402.00 | 582.00 | 0.60 | 0.73 | 0.69 | 3.78 | 2.66 | 4.15 | 0.00 | 2.24 | up |
| HORVU.MOREX.r2.6HG0487440 | 74.00 | 135.00 | 118.00 | 661.00 | 386.00 | 670.00 | 1.44 | 2.60 | 2.38 | 12.72 | 7.24 | 13.60 | 0.00 | 2.16 | up |
| HORVU.MOREX.r2.5HG0427630 | 43.00 | 15.00 | 20.00 | 124.00 | 64.00 | 74.00 | 0.40 | 0.13 | 0.19 | 1.13 | 0.57 | 0.71 | 0.01 | 1.34 | up |
| HORVU.MOREX.r2.2HG0098340 | 42.00 | 61.00 | 24.00 | 214.00 | 249.00 | 369.00 | 1.02 | 1.50 | 0.60 | 5.24 | 5.95 | 9.54 | 0.00 | 2.36 | up |
| HORVU.MOREX.r2.3HG0226390 | 142.00 | 229.00 | 167.00 | 543.00 | 497.00 | 530.00 | 1.23 | 1.98 | 1.52 | 4.69 | 4.18 | 4.82 | 0.00 | 1.45 | up |
| HORVU.MOREX.r2.6HG0448860 | 18.00 | 84.00 | 27.00 | 376.00 | 381.00 | 670.00 | 0.19 | 0.90 | 0.30 | 4.06 | 4.01 | 7.63 | 0.00 | 2.80 | up |
| HORVU.MOREX.r2.5HG0414110 | 17921.00 | 24093.00 | 19608.00 | 49361.00 | 53599.00 | 55038.00 | 112.19 | 149.76 | 127.80 | 305.17 | 323.05 | 358.51 | 0.00 | 1.30 | up |
| HORVU.MOREX.r2.3HG0186570 | 1242.00 | 564.00 | 183.00 | 7197.00 | 3880.00 | 5362.00 | 20.75 | 9.36 | 3.17 | 118.78 | 62.42 | 93.24 | 0.00 | 1.95 | up |
| HORVU.MOREX.r2.3HG0253580 | 19.00 | 14.00 | 2.00 | 916.00 | 504.00 | 643.00 | 0.21 | 0.16 | 0.02 | 10.62 | 5.69 | 7.85 | 0.00 | 4.91 | up |
| HORVU.MOREX.r2.5HG0431600 | 1447.00 | 1691.00 | 1766.00 | 39491.00 | 38377.00 | 46847.00 | 50.36 | 58.46 | 64.02 | 1358.16 | 1286.72 | 1697.57 | 0.00 | 4.52 | up |
| HORVU.MOREX.r2.7HG0529400 | 2.00 | 0.00 | 2.00 | 2730.00 | 1842.00 | 4150.00 | 0.02 | 0.00 | 0.02 | 26.22 | 17.25 | 41.99 | 0.00 | 8.29 | up |
| Hordeum_vulgare_newGene_680 | 25.00 | 25.00 | 25.00 | 89.00 | 76.00 | 123.00 | 0.49 | 0.46 | 0.48 | 1.72 | 1.41 | 2.51 | 0.00 | 1.66 | up |
| HORVU.MOREX.r2.1HG0036730 | 5.00 | 31.00 | 44.00 | 155.00 | 148.00 | 155.00 | 0.06 | 0.39 | 0.58 | 1.96 | 1.82 | 2.05 | 0.00 | 1.87 | up |
| HORVU.MOREX.r2.1HG0074170 | 2770.00 | 3277.00 | 2687.00 | 6768.00 | 6002.00 | 6446.00 | 51.47 | 60.45 | 51.97 | 124.18 | 107.36 | 124.62 | 0.00 | 1.08 | up |
| HORVU.MOREX.r2.7HG0547720 | 79.00 | 85.00 | 62.00 | 338.00 | 247.00 | 214.00 | 0.71 | 0.75 | 0.58 | 2.99 | 2.13 | 1.99 | 0.00 | 1.66 | up |
| HORVU.MOREX.r2.3HG0235580 | 229.00 | 278.00 | 179.00 | 647.00 | 652.00 | 795.00 | 5.90 | 7.13 | 4.79 | 16.50 | 16.21 | 21.36 | 0.00 | 1.51 | up |
| HORVU.MOREX.r2.5HG0425560 | 1976.00 | 2235.00 | 1880.00 | 12598.00 | 8165.00 | 9930.00 | 22.10 | 24.81 | 21.88 | 139.14 | 87.91 | 115.56 | 0.00 | 2.22 | up |
| HORVU.MOREX.r2.6HG0476040 | 41.00 | 78.00 | 61.00 | 284.00 | 287.00 | 364.00 | 1.34 | 2.54 | 2.07 | 9.26 | 9.12 | 12.49 | 0.00 | 2.17 | up |
| HORVU.MOREX.r2.7HG0556260 | 451.00 | 621.00 | 424.00 | 1405.00 | 1013.00 | 1142.00 | 9.12 | 12.49 | 8.94 | 28.10 | 19.76 | 24.06 | 0.00 | 1.17 | up |
| HORVU.MOREX.r2.5HG0396930 | 56.00 | 39.00 | 23.00 | 230.00 | 160.00 | 202.00 | 4.06 | 2.81 | 1.71 | 16.47 | 11.19 | 15.24 | 0.00 | 2.02 | up |
| HORVU.MOREX.r2.1HG0047000 | 3259.00 | 2796.00 | 2089.00 | 8939.00 | 7083.00 | 8417.00 | 56.06 | 47.76 | 37.41 | 151.88 | 117.33 | 150.68 | 0.00 | 1.49 | up |
| HORVU.MOREX.r2.1HG0078220 | 72.00 | 96.00 | 95.00 | 222.00 | 212.00 | 167.00 | 1.00 | 1.32 | 1.37 | 3.05 | 2.84 | 2.42 | 0.00 | 1.08 | up |
| HORVU.MOREX.r2.2HG0095300 | 10.00 | 21.00 | 7.00 | 688.00 | 681.00 | 741.00 | 0.28 | 0.59 | 0.20 | 19.16 | 18.50 | 21.74 | 0.00 | 5.28 | up |
| HORVU.MOREX.r2.4HG0337350 | 40.00 | 11.00 | 29.00 | 207.00 | 176.00 | 210.00 | 1.29 | 0.33 | 0.98 | 6.59 | 5.45 | 7.04 | 0.00 | 2.40 | up |
| HORVU.MOREX.r2.5HG0444050 | 20.00 | 22.00 | 17.00 | 76.00 | 97.00 | 102.00 | 0.28 | 0.31 | 0.25 | 1.06 | 1.32 | 1.50 | 0.00 | 1.92 | up |
| HORVU.MOREX.r2.7HG0527710 | 2575.00 | 2675.00 | 2778.00 | 4986.00 | 10680.00 | 13111.00 | 23.25 | 23.98 | 26.11 | 44.46 | 92.85 | 123.19 | 0.00 | 1.66 | up |
| HORVU.MOREX.r2.2HG0166950 | 425.00 | 527.00 | 393.00 | 2882.00 | 2061.00 | 2690.00 | 5.62 | 6.90 | 5.40 | 37.61 | 26.23 | 36.99 | 0.00 | 2.39 | up |
| HORVU.MOREX.r2.5HG0406790 | 490.00 | 610.00 | 436.00 | 1865.00 | 1244.00 | 1566.00 | 6.23 | 7.70 | 5.77 | 23.43 | 15.23 | 20.73 | 0.00 | 1.51 | up |
| HORVU.MOREX.r2.6HG0448330 | 174.00 | 288.00 | 172.00 | 691.00 | 469.00 | 589.00 | 2.05 | 3.39 | 2.11 | 8.07 | 5.34 | 7.26 | 0.00 | 1.35 | up |
| HORVU.MOREX.r2.3HG0269820 | 9411.00 | 4773.00 | 5217.00 | 22875.00 | 34938.00 | 58894.00 | 102.11 | 51.42 | 58.93 | 245.11 | 364.97 | 664.92 | 0.00 | 1.98 | up |
| HORVU.MOREX.r2.2HG0137040 | 203.00 | 220.00 | 229.00 | 1248.00 | 1192.00 | 1035.00 | 3.28 | 3.53 | 3.86 | 19.96 | 18.58 | 17.44 | 0.00 | 2.32 | up |
| HORVU.MOREX.r2.3HG0194540 | 110.00 | 185.00 | 157.00 | 326.00 | 327.00 | 601.00 | 1.21 | 2.03 | 1.79 | 3.55 | 3.47 | 6.90 | 0.00 | 1.31 | up |
| HORVU.MOREX.r2.3HG0234390 | 887.00 | 381.00 | 163.00 | 8924.00 | 4324.00 | 7355.00 | 4.11 | 1.75 | 0.78 | 40.84 | 19.29 | 35.46 | 0.00 | 2.47 | up |
| HORVU.MOREX.r2.1HG0038910 | 254.00 | 226.00 | 120.00 | 724.00 | 456.00 | 397.00 | 2.32 | 2.05 | 1.15 | 6.55 | 4.02 | 3.79 | 0.00 | 1.23 | up |
| HORVU.MOREX.r2.2HG0138080 | 2.00 | 2.00 | 4.00 | 146.00 | 75.00 | 275.00 | 0.03 | 0.03 | 0.07 | 2.70 | 1.35 | 5.37 | 0.00 | 4.01 | up |
| HORVU.MOREX.r2.2HG0083970 | 2586.00 | 2061.00 | 1675.00 | 23735.00 | 14430.00 | 18659.00 | 56.32 | 44.56 | 37.97 | 510.56 | 302.61 | 422.89 | 0.00 | 2.98 | up |
| HORVU.MOREX.r2.6HG0519270 | 42.00 | 79.00 | 31.00 | 145.00 | 114.00 | 118.00 | 1.32 | 2.47 | 1.00 | 4.52 | 3.46 | 3.86 | 0.00 | 1.12 | up |
| HORVU.MOREX.r2.2HG0155730 | 95.00 | 21.00 | 26.00 | 139.00 | 150.00 | 226.00 | 1.09 | 0.23 | 0.30 | 1.57 | 1.65 | 2.69 | 0.01 | 1.39 | up |
| HORVU.MOREX.r2.2HG0112800 | 229.00 | 338.00 | 220.00 | 1125.00 | 943.00 | 1256.00 | 1.58 | 2.32 | 1.58 | 7.69 | 6.28 | 9.05 | 0.00 | 1.96 | up |
| HORVU.MOREX.r2.3HG0252060 | 1398.00 | 1354.00 | 1061.00 | 8060.00 | 6158.00 | 8240.00 | 5.53 | 5.32 | 4.37 | 31.49 | 23.46 | 33.92 | 0.00 | 2.45 | up |
| HORVU.MOREX.r2.3HG0258600 | 284.00 | 229.00 | 152.00 | 919.00 | 707.00 | 1098.00 | 1.58 | 1.26 | 0.88 | 5.05 | 3.79 | 6.36 | 0.00 | 1.87 | up |
| HORVU.MOREX.r2.3HG0212880 | 36.00 | 35.00 | 22.00 | 67.00 | 94.00 | 75.00 | 1.11 | 1.08 | 0.69 | 2.05 | 2.81 | 2.40 | 0.00 | 1.14 | up |
| HORVU.MOREX.r2.7HG0568930 | 670.00 | 896.00 | 623.00 | 2800.00 | 1805.00 | 2271.00 | 7.79 | 10.35 | 7.54 | 32.16 | 20.21 | 27.47 | 0.00 | 1.55 | up |
| HORVU.MOREX.r2.4HG0282470 | 150.00 | 75.00 | 22.00 | 675.00 | 528.00 | 535.00 | 1.88 | 0.93 | 0.29 | 8.40 | 6.41 | 7.01 | 0.00 | 2.24 | up |
| HORVU.MOREX.r2.2HG0165740 | 0.00 | 0.00 | 1.00 | 532.00 | 301.00 | 1027.00 | 0.00 | 0.00 | 0.01 | 6.16 | 3.40 | 12.55 | 0.00 | 6.25 | up |
| HORVU.MOREX.r2.6HG0461220 | 5990.00 | 9823.00 | 6632.00 | 15863.00 | 15311.00 | 19706.00 | 96.76 | 157.56 | 111.53 | 253.07 | 238.14 | 331.24 | 0.00 | 1.11 | up |
| HORVU.MOREX.r2.7HG0619090 | 220.00 | 350.00 | 188.00 | 926.00 | 926.00 | 1262.00 | 5.87 | 9.25 | 5.21 | 24.37 | 23.78 | 35.01 | 0.00 | 1.89 | up |
| HORVU.MOREX.r2.1HG0066270 | 522.00 | 726.00 | 709.00 | 3003.00 | 3894.00 | 4820.00 | 13.98 | 19.32 | 19.78 | 79.48 | 100.47 | 134.40 | 0.00 | 2.43 | up |
| HORVU.MOREX.r2.7HG0610110 | 344.00 | 486.00 | 298.00 | 897.00 | 757.00 | 901.00 | 4.75 | 6.66 | 4.28 | 12.23 | 10.06 | 12.94 | 0.00 | 1.10 | up |
| HORVU.MOREX.r2.5HG0445910 | 18.00 | 50.00 | 18.00 | 100.00 | 82.00 | 158.00 | 0.15 | 0.42 | 0.15 | 0.83 | 0.66 | 1.39 | 0.00 | 1.58 | up |
| HORVU.MOREX.r2.6HG0522520 | 52.00 | 125.00 | 78.00 | 415.00 | 339.00 | 413.00 | 0.36 | 0.87 | 0.56 | 2.86 | 2.27 | 2.99 | 0.00 | 1.98 | up |
| HORVU.MOREX.r2.1HG0013370 | 421.00 | 299.00 | 211.00 | 2853.00 | 2524.00 | 3003.00 | 7.15 | 5.04 | 3.72 | 47.91 | 41.32 | 53.13 | 0.00 | 2.97 | up |
| HORVU.MOREX.r2.2HG0109440 | 140.00 | 105.00 | 65.00 | 1357.00 | 914.00 | 1515.00 | 1.15 | 0.85 | 0.55 | 11.01 | 7.22 | 12.94 | 0.00 | 3.29 | up |
| HORVU.MOREX.r2.3HG0239650 | 2.00 | 0.00 | 0.00 | 227.00 | 142.00 | 68.00 | 0.05 | 0.00 | 0.00 | 5.77 | 3.52 | 1.82 | 0.00 | 4.44 | up |
| HORVU.MOREX.r2.5HG0433970 | 3243.00 | 5826.00 | 3886.00 | 11023.00 | 7674.00 | 9164.00 | 80.93 | 144.38 | 100.97 | 271.69 | 184.40 | 237.99 | 0.00 | 1.02 | up |
| HORVU.MOREX.r2.5HG0435530 | 36.00 | 40.00 | 30.00 | 143.00 | 201.00 | 160.00 | 0.43 | 0.48 | 0.37 | 1.71 | 2.34 | 2.01 | 0.00 | 2.03 | up |
| HORVU.MOREX.r2.7HG0531330 | 226.00 | 193.00 | 213.00 | 871.00 | 874.00 | 1435.00 | 1.28 | 1.08 | 1.26 | 4.88 | 4.78 | 8.48 | 0.00 | 2.15 | up |
| HORVU.MOREX.r2.1HG0059150 | 55.00 | 30.00 | 37.00 | 93.00 | 93.00 | 105.00 | 0.68 | 0.36 | 0.47 | 1.14 | 1.11 | 1.36 | 0.00 | 1.07 | up |
| HORVU.MOREX.r2.1HG0057280 | 2492.00 | 1321.00 | 1213.00 | 7413.00 | 5330.00 | 7078.00 | 114.99 | 60.54 | 58.25 | 337.89 | 236.82 | 339.91 | 0.00 | 1.80 | up |
| HORVU.MOREX.r2.5HG0416230 | 46.00 | 6.00 | 22.00 | 114.00 | 175.00 | 168.00 | 0.65 | 0.08 | 0.32 | 1.59 | 2.38 | 2.48 | 0.00 | 1.92 | up |
| HORVU.MOREX.r2.3HG0232740 | 1377.00 | 1517.00 | 1310.00 | 8813.00 | 6430.00 | 7302.00 | 16.54 | 18.09 | 16.39 | 104.58 | 74.39 | 91.30 | 0.00 | 2.33 | up |
| HORVU.MOREX.r2.3HG0262320 | 503.00 | 356.00 | 273.00 | 999.00 | 874.00 | 1176.00 | 9.64 | 6.78 | 5.45 | 18.92 | 16.14 | 23.48 | 0.00 | 1.31 | up |
| HORVU.MOREX.r2.7HG0586520 | 138.00 | 271.00 | 114.00 | 754.00 | 381.00 | 601.00 | 1.95 | 3.82 | 1.69 | 10.58 | 5.21 | 8.89 | 0.00 | 1.52 | up |
| HORVU.MOREX.r2.2HG0177480 | 43.00 | 32.00 | 19.00 | 198.00 | 184.00 | 238.00 | 1.75 | 1.28 | 0.79 | 7.98 | 7.22 | 10.12 | 0.00 | 2.41 | up |
| HORVU.MOREX.r2.3HG0227190 | 31.00 | 54.00 | 43.00 | 155.00 | 106.00 | 148.00 | 0.44 | 0.76 | 0.64 | 2.21 | 1.47 | 2.21 | 0.00 | 1.47 | up |
| HORVU.MOREX.r2.3HG0186860 | 272.00 | 461.00 | 376.00 | 833.00 | 824.00 | 884.00 | 1.30 | 2.18 | 1.87 | 3.93 | 3.79 | 4.39 | 0.00 | 1.11 | up |
| HORVU.MOREX.r2.5HG0359910 | 251.00 | 280.00 | 262.00 | 314.00 | 715.00 | 756.00 | 3.35 | 3.72 | 3.65 | 4.15 | 9.21 | 10.53 | 0.00 | 1.03 | up |
| HORVU.MOREX.r2.5HG0410240 | 117.00 | 85.00 | 25.00 | 895.00 | 998.00 | 894.00 | 1.00 | 0.72 | 0.22 | 7.59 | 8.25 | 7.98 | 0.00 | 3.10 | up |
| HORVU.MOREX.r2.5HG0352030 | 1158.00 | 1138.00 | 900.00 | 51659.00 | 43610.00 | 50409.00 | 29.52 | 28.80 | 23.90 | 1301.31 | 1070.98 | 1337.93 | 0.00 | 5.36 | up |
| HORVU.MOREX.r2.7HG0549240 | 19.00 | 9.00 | 7.00 | 442.00 | 197.00 | 191.00 | 0.51 | 0.25 | 0.20 | 12.19 | 5.29 | 5.55 | 0.00 | 3.65 | up |
| HORVU.MOREX.r2.1HG0051320 | 1752.00 | 1280.00 | 1083.00 | 4536.00 | 4673.00 | 5122.00 | 30.23 | 21.93 | 19.44 | 77.29 | 77.64 | 91.96 | 0.00 | 1.69 | up |
| HORVU.MOREX.r2.2HG0163970 | 2.00 | 6.00 | 10.00 | 67.00 | 100.00 | 99.00 | 0.01 | 0.03 | 0.05 | 0.31 | 0.46 | 0.49 | 0.00 | 2.95 | up |
| HORVU.MOREX.r2.4HG0318640 | 256.00 | 220.00 | 254.00 | 584.00 | 531.00 | 535.00 | 12.71 | 10.81 | 13.11 | 28.64 | 25.39 | 27.62 | 0.00 | 1.10 | up |
| HORVU.MOREX.r2.2HG0150150 | 380.00 | 927.00 | 599.00 | 3006.00 | 2087.00 | 2577.00 | 2.40 | 5.81 | 3.93 | 18.74 | 12.69 | 16.93 | 0.00 | 1.83 | up |
| HORVU.MOREX.r2.7HG0531840 | 61.00 | 66.00 | 35.00 | 899.00 | 551.00 | 758.00 | 1.37 | 1.47 | 0.82 | 20.16 | 12.03 | 17.91 | 0.00 | 3.45 | up |
| HORVU.MOREX.r2.2HG0114520 | 865.00 | 967.00 | 684.00 | 2843.00 | 2683.00 | 3244.00 | 8.27 | 9.18 | 6.80 | 26.85 | 24.70 | 32.29 | 0.00 | 1.72 | up |
| HORVU.MOREX.r2.5HG0418270 | 2.00 | 4.00 | 2.00 | 191.00 | 109.00 | 131.00 | 0.01 | 0.03 | 0.01 | 1.29 | 0.72 | 0.93 | 0.00 | 4.30 | up |
| HORVU.MOREX.r2.7HG0583590 | 1406.00 | 1614.00 | 1062.00 | 3634.00 | 2939.00 | 3824.00 | 18.25 | 20.80 | 14.35 | 46.58 | 36.73 | 51.65 | 0.00 | 1.27 | up |
| HORVU.MOREX.r2.5HG0436750 | 1163.00 | 1280.00 | 984.00 | 3732.00 | 2907.00 | 3381.00 | 15.88 | 17.34 | 13.98 | 50.31 | 38.20 | 48.03 | 0.00 | 1.47 | up |
| HORVU.MOREX.r2.4HG0299180 | 9.00 | 10.00 | 23.00 | 375.00 | 312.00 | 111.00 | 0.32 | 0.36 | 0.86 | 13.78 | 11.17 | 4.30 | 0.00 | 3.28 | up |
| HORVU.MOREX.r2.7HG0617100 | 1868.00 | 2567.00 | 1337.00 | 16207.00 | 10994.00 | 16855.00 | 28.89 | 39.41 | 21.52 | 247.56 | 163.71 | 271.27 | 0.00 | 2.74 | up |
| HORVU.MOREX.r2.5HG0415160 | 0.00 | 0.00 | 0.00 | 83.00 | 80.00 | 173.00 | 0.00 | 0.00 | 0.00 | 5.73 | 5.34 | 12.61 | 0.00 | 4.53 | up |
| HORVU.MOREX.r2.2HG0151170 | 109.00 | 274.00 | 236.00 | 1524.00 | 1178.00 | 1508.00 | 1.29 | 3.25 | 2.93 | 17.97 | 13.53 | 18.73 | 0.00 | 2.51 | up |
| HORVU.MOREX.r2.3HG0189040 | 0.00 | 0.00 | 0.00 | 135.00 | 71.00 | 136.00 | 0.00 | 0.00 | 0.00 | 1.64 | 0.84 | 1.74 | 0.00 | 4.69 | up |
| HORVU.MOREX.r2.7HG0542800 | 71.00 | 81.00 | 53.00 | 664.00 | 211.00 | 477.00 | 0.54 | 0.61 | 0.42 | 5.04 | 1.56 | 3.81 | 0.00 | 2.32 | up |
| HORVU.MOREX.r2.2HG0152280 | 206.00 | 414.00 | 273.00 | 860.00 | 707.00 | 736.00 | 2.32 | 4.63 | 3.20 | 9.57 | 7.66 | 8.63 | 0.00 | 1.26 | up |
| HORVU.MOREX.r2.6HG0456270 | 282.00 | 325.00 | 16.00 | 21637.00 | 13558.00 | 12742.00 | 2.96 | 3.39 | 0.17 | 225.12 | 137.52 | 139.68 | 0.00 | 2.43 | up |
| HORVU.MOREX.r2.3HG0268090 | 450.00 | 879.00 | 674.00 | 1906.00 | 2023.00 | 2007.00 | 5.41 | 10.50 | 8.44 | 22.66 | 23.45 | 25.14 | 0.00 | 1.46 | up |
| HORVU.MOREX.r2.3HG0233570 | 61.00 | 146.00 | 47.00 | 9893.00 | 9475.00 | 13197.00 | 1.09 | 2.61 | 0.87 | 176.99 | 165.26 | 248.77 | 0.00 | 5.43 | up |
| HORVU.MOREX.r2.2HG0154390 | 488.00 | 323.00 | 148.00 | 1930.00 | 1678.00 | 1672.00 | 5.82 | 3.82 | 1.83 | 22.71 | 19.25 | 20.73 | 0.00 | 2.18 | up |
| Hordeum_vulgare_newGene_5990 | 6520.00 | 4732.00 | 4592.00 | 12035.00 | 12975.00 | 19294.00 | 121.93 | 87.85 | 89.40 | 222.26 | 233.59 | 375.42 | 0.00 | 1.37 | up |
| HORVU.MOREX.r2.2HG0178520 | 167.00 | 129.00 | 63.00 | 2534.00 | 1776.00 | 2372.00 | 1.85 | 1.42 | 0.73 | 27.93 | 19.08 | 27.55 | 0.00 | 3.84 | up |
| HORVU.MOREX.r2.7HG0607230 | 473.00 | 391.00 | 306.00 | 1672.00 | 1163.00 | 1582.00 | 5.08 | 4.17 | 3.42 | 17.78 | 12.05 | 17.73 | 0.00 | 1.79 | up |
| HORVU.MOREX.r2.1HG0040550 | 119.00 | 42.00 | 10.00 | 7215.00 | 3952.00 | 6943.00 | 2.92 | 1.01 | 0.26 | 174.84 | 93.35 | 177.27 | 0.00 | 3.27 | up |
| HORVU.MOREX.r2.3HG0260840 | 170.00 | 317.00 | 142.00 | 1249.00 | 1003.00 | 1286.00 | 2.30 | 4.25 | 1.99 | 16.68 | 13.05 | 18.09 | 0.00 | 2.29 | up |
| Hordeum_vulgare_newGene_43 | 16.00 | 20.00 | 19.00 | 322.00 | 195.00 | 511.00 | 0.14 | 0.18 | 0.18 | 2.90 | 1.71 | 4.86 | 0.00 | 3.57 | up |
| HORVU.MOREX.r2.6HG0453610 | 1306.00 | 836.00 | 768.00 | 2085.00 | 1994.00 | 2264.00 | 47.47 | 30.14 | 29.05 | 74.84 | 69.77 | 85.61 | 0.00 | 1.03 | up |
| HORVU.MOREX.r2.2HG0160590 | 132.00 | 158.00 | 92.00 | 6225.00 | 3287.00 | 4384.00 | 2.30 | 2.74 | 1.67 | 108.00 | 55.60 | 80.15 | 0.00 | 4.82 | up |
| HORVU.MOREX.r2.1HG0040610 | 28.00 | 34.00 | 16.00 | 666.00 | 244.00 | 474.00 | 0.68 | 0.82 | 0.41 | 16.12 | 5.74 | 12.09 | 0.00 | 3.52 | up |
| HORVU.MOREX.r2.1HG0024150 | 199.00 | 270.00 | 190.00 | 856.00 | 514.00 | 656.00 | 2.25 | 3.02 | 2.23 | 9.55 | 5.59 | 7.71 | 0.00 | 1.50 | up |
| HORVU.MOREX.r2.5HG0367920 | 119.00 | 222.00 | 122.00 | 1041.00 | 909.00 | 1039.00 | 4.14 | 7.66 | 4.39 | 35.78 | 30.47 | 37.65 | 0.00 | 2.51 | up |
| HORVU.MOREX.r2.7HG0549080 | 364.00 | 1253.00 | 586.00 | 62664.00 | 45457.00 | 48655.00 | 12.81 | 43.84 | 21.47 | 2181.25 | 1542.56 | 1784.43 | 0.00 | 4.81 | up |
| HORVU.MOREX.r2.3HG0259290 | 892.00 | 1054.00 | 641.00 | 7134.00 | 4897.00 | 7327.00 | 14.24 | 16.71 | 10.65 | 112.55 | 75.33 | 121.81 | 0.00 | 2.74 | up |
| HORVU.MOREX.r2.2HG0165980 | 0.00 | 2.00 | 0.00 | 83.00 | 65.00 | 74.00 | 0.00 | 0.01 | 0.00 | 0.58 | 0.45 | 0.55 | 0.00 | 4.04 | up |
| HORVU.MOREX.r2.7HG0549620 | 443.00 | 557.00 | 506.00 | 1622.00 | 1156.00 | 1444.00 | 5.91 | 7.37 | 7.03 | 21.36 | 14.84 | 20.03 | 0.00 | 1.40 | up |
| HORVU.MOREX.r2.1HG0066180 | 116.00 | 84.00 | 95.00 | 255.00 | 195.00 | 323.00 | 1.99 | 1.42 | 1.69 | 4.31 | 3.22 | 5.75 | 0.00 | 1.24 | up |
| HORVU.MOREX.r2.4HG0278450 | 177.00 | 134.00 | 117.00 | 1259.00 | 832.00 | 998.00 | 14.84 | 11.16 | 10.20 | 104.76 | 67.52 | 87.47 | 0.00 | 2.66 | up |
| HORVU.MOREX.r2.7HG0555100 | 3.00 | 35.00 | 31.00 | 237.00 | 132.00 | 190.00 | 0.22 | 2.61 | 2.42 | 17.62 | 9.60 | 14.92 | 0.00 | 2.18 | up |
| HORVU.MOREX.r2.7HG0528940 | 81.00 | 111.00 | 90.00 | 384.00 | 208.00 | 272.00 | 0.48 | 0.64 | 0.55 | 2.22 | 1.17 | 1.66 | 0.00 | 1.45 | up |
| HORVU.MOREX.r2.1HG0051740 | 2016.00 | 2485.00 | 1704.00 | 5620.00 | 4477.00 | 5146.00 | 20.28 | 24.82 | 17.85 | 55.84 | 43.36 | 53.87 | 0.00 | 1.23 | up |
| HORVU.MOREX.r2.2HG0159320 | 63.00 | 44.00 | 74.00 | 697.00 | 389.00 | 365.00 | 1.99 | 1.38 | 2.45 | 21.97 | 11.96 | 12.13 | 0.00 | 2.65 | up |
| HORVU.MOREX.r2.4HG0347310 | 37.00 | 34.00 | 24.00 | 188.00 | 216.00 | 378.00 | 0.47 | 0.43 | 0.31 | 2.35 | 2.63 | 4.98 | 0.00 | 2.64 | up |
| Hordeum_vulgare_newGene_3942 | 39.00 | 90.00 | 49.00 | 195.00 | 186.00 | 352.00 | 1.35 | 3.15 | 1.78 | 6.82 | 6.34 | 12.99 | 0.00 | 1.75 | up |
| HORVU.MOREX.r2.2HG0149820 | 45.00 | 27.00 | 13.00 | 76.00 | 246.00 | 212.00 | 1.06 | 0.62 | 0.32 | 1.77 | 5.64 | 5.24 | 0.00 | 2.02 | up |
| Hordeum_vulgare_newGene_6599 | 1398.00 | 758.00 | 633.00 | 10662.00 | 10404.00 | 17058.00 | 23.49 | 12.62 | 11.08 | 177.47 | 168.83 | 300.28 | 0.00 | 3.41 | up |
| HORVU.MOREX.r2.5HG0387900 | 741.00 | 531.00 | 472.00 | 1869.00 | 3171.00 | 2772.00 | 11.99 | 8.53 | 7.96 | 29.90 | 49.45 | 46.72 | 0.00 | 2.00 | up |
| HORVU.MOREX.r2.1HG0007090 | 13.00 | 24.00 | 24.00 | 123.00 | 135.00 | 155.00 | 0.14 | 0.26 | 0.28 | 1.39 | 1.48 | 1.85 | 0.00 | 2.41 | up |
| HORVU.MOREX.r2.UnG0624610 | 48.00 | 35.00 | 32.00 | 165.00 | 144.00 | 234.00 | 0.40 | 0.29 | 0.28 | 1.39 | 1.18 | 2.08 | 0.00 | 1.97 | up |
| HORVU.MOREX.r2.6HG0451760 | 301.00 | 395.00 | 304.00 | 2042.00 | 1276.00 | 1817.00 | 4.80 | 6.26 | 5.05 | 32.22 | 19.62 | 30.20 | 0.00 | 2.22 | up |
| HORVU.MOREX.r2.2HG0161460 | 30.00 | 10.00 | 8.00 | 315.00 | 203.00 | 361.00 | 0.29 | 0.10 | 0.07 | 3.11 | 1.95 | 3.75 | 0.00 | 3.40 | up |
| HORVU.MOREX.r2.1HG0067240 | 683.00 | 856.00 | 794.00 | 3262.00 | 2075.00 | 2974.00 | 9.19 | 11.44 | 11.12 | 43.37 | 26.89 | 41.65 | 0.00 | 1.72 | up |
| HORVU.MOREX.r2.5HG0356190 | 157.00 | 188.00 | 136.00 | 268.00 | 406.00 | 521.00 | 6.32 | 7.50 | 5.68 | 10.65 | 15.78 | 21.86 | 0.00 | 1.18 | up |
| HORVU.MOREX.r2.5HG0402410 | 614.00 | 486.00 | 358.00 | 1600.00 | 1191.00 | 1605.00 | 8.98 | 7.06 | 5.45 | 23.13 | 16.79 | 24.45 | 0.00 | 1.48 | up |
| HORVU.MOREX.r2.4HG0338500 | 282.00 | 440.00 | 604.00 | 1436.00 | 865.00 | 1100.00 | 13.86 | 21.52 | 30.99 | 69.87 | 41.00 | 56.40 | 0.00 | 1.20 | up |
| HORVU.MOREX.r2.1HG0058590 | 1983.00 | 2526.00 | 1486.00 | 4942.00 | 4995.00 | 4598.00 | 20.89 | 26.41 | 16.29 | 51.42 | 50.66 | 50.40 | 0.00 | 1.20 | up |
| HORVU.MOREX.r2.3HG0263380 | 75.00 | 39.00 | 17.00 | 362.00 | 173.00 | 349.00 | 1.01 | 0.52 | 0.23 | 4.83 | 2.24 | 4.91 | 0.00 | 2.19 | up |
| HORVU.MOREX.r2.7HG0611160 | 62.00 | 60.00 | 14.00 | 350.00 | 265.00 | 270.00 | 1.44 | 1.38 | 0.32 | 8.07 | 5.95 | 6.55 | 0.00 | 2.24 | up |
| HORVU.MOREX.r2.2HG0135750 | 645.00 | 552.00 | 333.00 | 8022.00 | 5075.00 | 5810.00 | 4.83 | 4.10 | 2.60 | 59.37 | 36.61 | 45.30 | 0.00 | 3.38 | up |
| HORVU.MOREX.r2.4HG0327010 | 267.00 | 656.00 | 337.00 | 1423.00 | 829.00 | 1133.00 | 3.51 | 8.58 | 4.62 | 18.53 | 10.52 | 15.54 | 0.00 | 1.27 | up |
| HORVU.MOREX.r2.2HG0083080 | 31.00 | 47.00 | 57.00 | 327.00 | 172.00 | 274.00 | 0.27 | 0.41 | 0.52 | 2.87 | 1.47 | 2.54 | 0.00 | 2.19 | up |
| Hordeum_vulgare_newGene_2801 | 3892.00 | 4476.00 | 3705.00 | 10337.00 | 11848.00 | 14784.00 | 153.60 | 175.39 | 152.25 | 402.94 | 450.25 | 607.20 | 0.00 | 1.53 | up |
| HORVU.MOREX.r2.1HG0007110 | 1844.00 | 2831.00 | 1799.00 | 11324.00 | 7311.00 | 8716.00 | 32.59 | 49.68 | 33.10 | 197.67 | 124.41 | 160.32 | 0.00 | 1.96 | up |
| HORVU.MOREX.r2.2HG0151240 | 433.00 | 546.00 | 389.00 | 1113.00 | 881.00 | 1095.00 | 3.49 | 4.37 | 3.27 | 8.87 | 6.85 | 9.20 | 0.00 | 1.10 | up |
| HORVU.MOREX.r2.2HG0147240 | 516.00 | 451.00 | 399.00 | 2599.00 | 2228.00 | 2958.00 | 11.03 | 9.56 | 8.88 | 54.88 | 45.85 | 65.81 | 0.00 | 2.39 | up |
| HORVU.MOREX.r2.7HG0592480 | 4891.00 | 2387.00 | 853.00 | 17446.00 | 14327.00 | 17067.00 | 98.77 | 47.85 | 17.92 | 347.90 | 278.54 | 358.59 | 0.00 | 1.80 | up |
| HORVU.MOREX.r2.7HG0533680 | 2269.00 | 1628.00 | 882.00 | 6205.00 | 5584.00 | 6505.00 | 68.71 | 48.95 | 27.81 | 185.60 | 162.82 | 205.02 | 0.00 | 1.76 | up |
| HORVU.MOREX.r2.UnG0626650 | 15287.00 | 8326.00 | 7838.00 | 127821.00 | 141406.00 | 183384.00 | 510.95 | 276.32 | 272.73 | 4219.16 | 4550.37 | 6377.84 | 0.00 | 3.55 | up |
| HORVU.MOREX.r2.2HG0171400 | 362.00 | 409.00 | 318.00 | 709.00 | 625.00 | 1242.00 | 18.62 | 20.86 | 16.99 | 35.99 | 30.94 | 66.46 | 0.00 | 1.12 | up |
| HORVU.MOREX.r2.7HG0623110 | 108.00 | 69.00 | 56.00 | 1377.00 | 1331.00 | 1482.00 | 1.20 | 0.75 | 0.65 | 15.14 | 14.28 | 17.17 | 0.00 | 3.91 | up |
| HORVU.MOREX.r2.5HG0446810 | 202.00 | 329.00 | 256.00 | 1053.00 | 862.00 | 1268.00 | 1.80 | 2.91 | 2.37 | 9.27 | 7.40 | 11.76 | 0.00 | 1.88 | up |
| HORVU.MOREX.r2.3HG0239750 | 5058.00 | 8204.00 | 6564.00 | 45255.00 | 32218.00 | 40341.00 | 20.41 | 32.87 | 27.58 | 180.37 | 125.19 | 169.41 | 0.00 | 2.44 | up |
| HORVU.MOREX.r2.2HG0165630 | 412.00 | 344.00 | 168.00 | 523.00 | 745.00 | 1475.00 | 6.97 | 5.78 | 2.95 | 8.76 | 12.15 | 26.01 | 0.00 | 1.33 | up |
| HORVU.MOREX.r2.7HG0587150 | 433.00 | 367.00 | 335.00 | 1093.00 | 831.00 | 956.00 | 5.05 | 4.25 | 4.06 | 12.60 | 9.34 | 11.61 | 0.00 | 1.26 | up |
| HORVU.MOREX.r2.5HG0430550 | 5.00 | 10.00 | 27.00 | 272.00 | 161.00 | 153.00 | 0.06 | 0.12 | 0.33 | 3.24 | 1.87 | 1.91 | 0.00 | 2.91 | up |
| HORVU.MOREX.r2.6HG0496940 | 392.00 | 249.00 | 225.00 | 1282.00 | 962.00 | 954.00 | 5.00 | 3.15 | 2.99 | 16.18 | 11.84 | 12.68 | 0.00 | 1.73 | up |
| HORVU.MOREX.r2.1HG0074860 | 27.00 | 20.00 | 11.00 | 143.00 | 91.00 | 123.00 | 0.29 | 0.22 | 0.12 | 1.57 | 0.97 | 1.43 | 0.00 | 2.20 | up |
| HORVU.MOREX.r2.5HG0384820 | 7.00 | 0.00 | 9.00 | 74.00 | 89.00 | 146.00 | 0.06 | 0.00 | 0.09 | 0.78 | 0.91 | 1.61 | 0.00 | 2.88 | up |
| HORVU.MOREX.r2.2HG0154440 | 263.00 | 156.00 | 105.00 | 713.00 | 648.00 | 894.00 | 14.42 | 8.49 | 5.97 | 38.62 | 34.22 | 51.00 | 0.00 | 1.88 | up |
| HORVU.MOREX.r2.4HG0315810 | 569.00 | 756.00 | 503.00 | 2484.00 | 2599.00 | 3063.00 | 9.29 | 12.26 | 8.56 | 40.07 | 40.87 | 52.06 | 0.00 | 2.06 | up |
| HORVU.MOREX.r2.3HG0200650 | 392.00 | 406.00 | 344.00 | 1082.00 | 1205.00 | 1491.00 | 6.77 | 6.97 | 6.18 | 18.48 | 20.07 | 26.85 | 0.00 | 1.63 | up |
| HORVU.MOREX.r2.5HG0391680 | 78.00 | 85.00 | 73.00 | 222.00 | 176.00 | 240.00 | 0.42 | 0.45 | 0.40 | 1.18 | 0.91 | 1.34 | 0.00 | 1.31 | up |
| HORVU.MOREX.r2.2HG0139090 | 516.00 | 395.00 | 257.00 | 936.00 | 682.00 | 965.00 | 8.97 | 6.82 | 4.65 | 16.08 | 11.42 | 17.47 | 0.00 | 1.03 | up |
| HORVU.MOREX.r2.2HG0084180 | 451.00 | 724.00 | 333.00 | 3630.00 | 1912.00 | 2544.00 | 2.42 | 3.86 | 1.86 | 19.25 | 9.88 | 14.21 | 0.00 | 2.21 | up |
| HORVU.MOREX.r2.4HG0337250 | 344.00 | 350.00 | 448.00 | 1368.00 | 1123.00 | 1359.00 | 6.75 | 6.82 | 9.15 | 26.54 | 21.24 | 27.78 | 0.00 | 1.65 | up |
| HORVU.MOREX.r2.3HG0233270 | 352.00 | 479.00 | 267.00 | 957.00 | 730.00 | 913.00 | 3.17 | 4.29 | 2.50 | 8.52 | 6.33 | 8.56 | 0.00 | 1.15 | up |
| HORVU.MOREX.r2.5HG0352100 | 252.00 | 232.00 | 202.00 | 612.00 | 443.00 | 472.00 | 6.14 | 5.62 | 5.13 | 14.77 | 10.40 | 11.98 | 0.00 | 1.07 | up |
| HORVU.MOREX.r2.3HG0259560 | 27.00 | 40.00 | 38.00 | 115.00 | 65.00 | 82.00 | 1.74 | 2.61 | 2.60 | 7.53 | 4.13 | 5.67 | 0.01 | 1.11 | up |
| HORVU.MOREX.r2.2HG0148400 | 1082.00 | 1185.00 | 1006.00 | 1989.00 | 2956.00 | 3403.00 | 30.98 | 33.69 | 29.98 | 56.25 | 81.52 | 101.42 | 0.00 | 1.26 | up |
| HORVU.MOREX.r2.1HG0031840 | 53.00 | 64.00 | 22.00 | 268.00 | 179.00 | 240.00 | 0.67 | 0.81 | 0.29 | 3.43 | 2.22 | 3.23 | 0.00 | 1.99 | up |
| HORVU.MOREX.r2.1HG0043770 | 62.00 | 155.00 | 120.00 | 345.00 | 374.00 | 311.00 | 1.07 | 2.69 | 2.18 | 5.96 | 6.31 | 5.65 | 0.00 | 1.43 | up |
| HORVU.MOREX.r2.6HG0479800 | 110.00 | 126.00 | 72.00 | 804.00 | 544.00 | 696.00 | 3.66 | 4.16 | 2.49 | 26.53 | 17.50 | 24.19 | 0.00 | 2.53 | up |
| HORVU.MOREX.r2.2HG0145690 | 535.00 | 487.00 | 407.00 | 646.00 | 1451.00 | 1752.00 | 9.04 | 8.17 | 7.16 | 10.78 | 23.61 | 30.80 | 0.00 | 1.26 | up |
| HORVU.MOREX.r2.6HG0479960 | 0.00 | 0.00 | 0.00 | 1289.00 | 990.00 | 1583.00 | 0.00 | 0.00 | 0.00 | 13.41 | 10.04 | 17.35 | 0.00 | 7.93 | up |
| HORVU.MOREX.r2.1HG0077030 | 3450.00 | 4685.00 | 4223.00 | 51117.00 | 32041.00 | 49218.00 | 60.06 | 80.99 | 76.55 | 879.00 | 537.13 | 891.74 | 0.00 | 3.24 | up |
| HORVU.MOREX.r2.2HG0091370 | 147.00 | 112.00 | 77.00 | 376.00 | 290.00 | 332.00 | 2.33 | 1.77 | 1.27 | 5.91 | 4.44 | 5.50 | 0.00 | 1.42 | up |
| HORVU.MOREX.r2.4HG0278610 | 1072.00 | 622.00 | 734.00 | 3334.00 | 1389.00 | 2021.00 | 24.94 | 14.36 | 17.75 | 76.57 | 31.10 | 48.91 | 0.00 | 1.29 | up |
| HORVU.MOREX.r2.3HG0195630 | 196.00 | 78.00 | 31.00 | 2365.00 | 1233.00 | 1277.00 | 1.68 | 0.66 | 0.28 | 20.06 | 10.19 | 11.41 | 0.00 | 2.41 | up |
| HORVU.MOREX.r2.7HG0616250 | 106.00 | 176.00 | 261.00 | 483.00 | 293.00 | 512.00 | 1.91 | 3.17 | 4.93 | 8.66 | 5.11 | 9.67 | 0.01 | 1.06 | up |
| HORVU.MOREX.r2.5HG0359870 | 239.00 | 269.00 | 96.00 | 1874.00 | 1291.00 | 1704.00 | 4.42 | 4.94 | 1.84 | 34.28 | 23.02 | 32.83 | 0.00 | 2.71 | up |
| HORVU.MOREX.r2.1HG0050390 | 473.00 | 389.00 | 481.00 | 1929.00 | 1603.00 | 2131.00 | 6.37 | 5.21 | 6.75 | 25.70 | 20.81 | 29.91 | 0.00 | 1.96 | up |
| HORVU.MOREX.r2.5HG0437180 | 521.00 | 1231.00 | 853.00 | 3952.00 | 3992.00 | 5103.00 | 10.73 | 25.19 | 18.31 | 80.48 | 79.25 | 109.49 | 0.00 | 2.14 | up |
| HORVU.MOREX.r2.1HG0057620 | 569.00 | 184.00 | 96.00 | 1434.00 | 1585.00 | 1503.00 | 6.91 | 2.21 | 1.21 | 17.23 | 18.56 | 19.03 | 0.01 | 1.56 | up |
| HORVU.MOREX.r2.1HG0042300 | 11.00 | 45.00 | 26.00 | 124.00 | 105.00 | 135.00 | 0.09 | 0.35 | 0.21 | 0.98 | 0.81 | 1.13 | 0.00 | 1.75 | up |
| HORVU.MOREX.r2.5HG0416810 | 1470.00 | 1444.00 | 1235.00 | 3990.00 | 2923.00 | 3989.00 | 26.06 | 25.42 | 22.79 | 69.87 | 49.89 | 73.59 | 0.00 | 1.32 | up |
| HORVU.MOREX.r2.7HG0555350 | 75.00 | 75.00 | 90.00 | 138.00 | 261.00 | 173.00 | 3.11 | 3.09 | 3.91 | 5.66 | 10.51 | 7.49 | 0.00 | 1.09 | up |
| HORVU.MOREX.r2.3HG0237240 | 211.00 | 158.00 | 112.00 | 2898.00 | 1839.00 | 2287.00 | 3.06 | 2.28 | 1.69 | 41.61 | 25.74 | 34.59 | 0.00 | 3.60 | up |
| Hordeum_vulgare_newGene_4654 | 44.00 | 36.00 | 18.00 | 140.00 | 106.00 | 167.00 | 0.40 | 0.32 | 0.16 | 1.25 | 0.93 | 1.58 | 0.00 | 1.76 | up |
| HORVU.MOREX.r2.3HG0233040 | 225.00 | 146.00 | 119.00 | 999.00 | 724.00 | 858.00 | 3.47 | 2.22 | 1.90 | 15.21 | 10.75 | 13.76 | 0.00 | 2.20 | up |
| HORVU.MOREX.r2.1HG0025160 | 122.00 | 89.00 | 122.00 | 287.00 | 493.00 | 421.00 | 2.83 | 2.06 | 2.95 | 6.61 | 11.08 | 10.23 | 0.00 | 1.67 | up |
| HORVU.MOREX.r2.3HG0261000 | 14.00 | 14.00 | 5.00 | 192.00 | 105.00 | 149.00 | 0.25 | 0.24 | 0.08 | 3.38 | 1.81 | 2.77 | 0.00 | 3.08 | up |
| HORVU.MOREX.r2.1HG0069390 | 17.00 | 75.00 | 62.00 | 234.00 | 124.00 | 137.00 | 0.53 | 2.34 | 2.04 | 7.32 | 3.79 | 4.53 | 0.01 | 1.32 | up |
| HORVU.MOREX.r2.1HG0049980 | 3.00 | 7.00 | 0.00 | 274.00 | 170.00 | 332.00 | 0.01 | 0.05 | 0.00 | 2.03 | 1.23 | 2.59 | 0.00 | 4.76 | up |
| HORVU.MOREX.r2.5HG0397720 | 112.00 | 79.00 | 54.00 | 494.00 | 402.00 | 612.00 | 2.10 | 1.47 | 1.04 | 9.15 | 7.26 | 11.94 | 0.00 | 2.37 | up |
| HORVU.MOREX.r2.4HG0337360 | 1096.00 | 566.00 | 455.00 | 3630.00 | 2604.00 | 4029.00 | 30.63 | 15.70 | 13.22 | 100.23 | 70.07 | 117.21 | 0.00 | 2.04 | up |
| HORVU.MOREX.r2.3HG0245010 | 108.00 | 82.00 | 68.00 | 323.00 | 284.00 | 386.00 | 0.69 | 0.52 | 0.45 | 2.04 | 1.75 | 2.57 | 0.00 | 1.78 | up |
| HORVU.MOREX.r2.7HG0551820 | 4.00 | 2.00 | 6.00 | 1000.00 | 407.00 | 692.00 | 0.08 | 0.04 | 0.16 | 25.41 | 10.06 | 18.52 | 0.00 | 5.91 | up |
| HORVU.MOREX.r2.3HG0195670 | 1144.00 | 1136.00 | 1146.00 | 2969.00 | 2489.00 | 2801.00 | 10.95 | 10.80 | 11.43 | 28.09 | 22.95 | 27.92 | 0.00 | 1.20 | up |
| HORVU.MOREX.r2.5HG0411280 | 1130.00 | 1016.00 | 592.00 | 3082.00 | 1686.00 | 2063.00 | 7.09 | 6.33 | 3.87 | 19.11 | 10.19 | 13.48 | 0.00 | 1.19 | up |
| HORVU.MOREX.r2.6HG0449490 | 207.00 | 155.00 | 132.00 | 436.00 | 385.00 | 509.00 | 3.41 | 2.53 | 2.27 | 7.11 | 6.11 | 8.75 | 0.00 | 1.31 | up |
| HORVU.MOREX.r2.5HG0355230 | 9.00 | 1.00 | 9.00 | 188.00 | 72.00 | 50.00 | 0.13 | 0.01 | 0.13 | 2.58 | 0.96 | 0.71 | 0.00 | 2.57 | up |
| HORVU.MOREX.r2.3HG0260250 | 20.00 | 10.00 | 8.00 | 3570.00 | 2217.00 | 5237.00 | 0.49 | 0.24 | 0.20 | 88.00 | 53.26 | 136.02 | 0.00 | 7.05 | up |
| HORVU.MOREX.r2.1HG0047770 | 23.00 | 18.00 | 16.00 | 134.00 | 159.00 | 227.00 | 0.56 | 0.43 | 0.41 | 3.25 | 3.77 | 5.83 | 0.00 | 2.76 | up |
| HORVU.MOREX.r2.5HG0397880 | 3.00 | 8.00 | 12.00 | 585.00 | 291.00 | 343.00 | 0.03 | 0.11 | 0.17 | 7.74 | 3.75 | 4.77 | 0.00 | 4.71 | up |
| HORVU.MOREX.r2.4HG0333660 | 0.00 | 0.00 | 0.00 | 139.00 | 138.00 | 208.00 | 0.00 | 0.00 | 0.00 | 1.89 | 1.82 | 2.98 | 0.00 | 5.29 | up |
| HORVU.MOREX.r2.4HG0280910 | 6154.00 | 6965.00 | 5985.00 | 27954.00 | 23040.00 | 29108.00 | 240.20 | 269.90 | 243.21 | 1077.53 | 865.80 | 1182.18 | 0.00 | 1.99 | up |
| HORVU.MOREX.r2.6HG0511920 | 144.00 | 79.00 | 104.00 | 317.00 | 320.00 | 372.00 | 2.63 | 1.42 | 1.98 | 5.74 | 5.65 | 7.09 | 0.00 | 1.47 | up |
| HORVU.MOREX.r2.5HG0362960 | 8.00 | 27.00 | 12.00 | 567.00 | 445.00 | 475.00 | 0.07 | 0.24 | 0.11 | 5.03 | 3.85 | 4.44 | 0.00 | 4.44 | up |
| HORVU.MOREX.r2.6HG0512380 | 39.00 | 12.00 | 20.00 | 1885.00 | 821.00 | 1106.00 | 0.68 | 0.19 | 0.36 | 32.60 | 13.83 | 20.15 | 0.00 | 4.88 | up |
| Hordeum_vulgare_newGene_3507 | 4201.00 | 3465.00 | 2401.00 | 10645.00 | 11849.00 | 13342.00 | 77.81 | 63.71 | 46.29 | 194.70 | 211.28 | 257.12 | 0.00 | 1.72 | up |
| HORVU.MOREX.r2.6HG0503270 | 451.00 | 272.00 | 205.00 | 1809.00 | 1454.00 | 1957.00 | 5.42 | 3.24 | 2.56 | 21.47 | 16.82 | 24.46 | 0.00 | 2.27 | up |
| HORVU.MOREX.r2.5HG0429900 | 10604.00 | 17281.00 | 13819.00 | 36486.00 | 34829.00 | 35669.00 | 45.35 | 73.37 | 61.52 | 154.08 | 143.39 | 158.71 | 0.00 | 1.28 | up |
| HORVU.MOREX.r2.3HG0248090 | 464.00 | 431.00 | 312.00 | 710.00 | 918.00 | 1374.00 | 3.91 | 3.60 | 2.74 | 5.91 | 7.46 | 12.06 | 0.00 | 1.19 | up |
| HORVU.MOREX.r2.UnG0631950 | 322.00 | 645.00 | 394.00 | 4061.00 | 2419.00 | 4924.00 | 3.14 | 6.24 | 3.99 | 39.13 | 22.72 | 49.99 | 0.00 | 2.79 | up |
| HORVU.MOREX.r2.6HG0491120 | 2.00 | 7.00 | 0.00 | 477.00 | 191.00 | 299.00 | 0.03 | 0.09 | 0.00 | 6.89 | 2.69 | 4.55 | 0.00 | 4.95 | up |
| HORVU.MOREX.r2.1HG0065060 | 466.00 | 616.00 | 351.00 | 1034.00 | 1195.00 | 1174.00 | 7.66 | 10.07 | 6.01 | 16.81 | 18.95 | 20.12 | 0.00 | 1.17 | up |
| HORVU.MOREX.r2.7HG0527240 | 41.00 | 27.00 | 34.00 | 69.00 | 121.00 | 211.00 | 0.78 | 0.51 | 0.68 | 1.30 | 2.23 | 4.22 | 0.00 | 1.58 | up |
| HORVU.MOREX.r2.1HG0031680 | 640.00 | 895.00 | 759.00 | 1636.00 | 1528.00 | 1686.00 | 10.28 | 14.26 | 12.69 | 25.95 | 23.62 | 28.18 | 0.00 | 1.02 | up |
| HORVU.MOREX.r2.3HG0261990 | 7.00 | 10.00 | 7.00 | 8478.00 | 2966.00 | 6568.00 | 0.06 | 0.09 | 0.07 | 82.25 | 28.05 | 67.13 | 0.00 | 7.02 | up |
| HORVU.MOREX.r2.7HG0589860 | 29.00 | 26.00 | 14.00 | 1371.00 | 1148.00 | 1341.00 | 0.52 | 0.46 | 0.26 | 24.67 | 20.13 | 25.43 | 0.00 | 5.43 | up |
| HORVU.MOREX.r2.6HG0526450 | 38.00 | 15.00 | 33.00 | 90.00 | 129.00 | 92.00 | 0.25 | 0.09 | 0.23 | 0.60 | 0.83 | 0.64 | 0.00 | 1.52 | up |
| HORVU.MOREX.r2.1HG0010850 | 349.00 | 388.00 | 256.00 | 1726.00 | 1215.00 | 1348.00 | 4.30 | 4.74 | 3.28 | 21.00 | 14.40 | 17.27 | 0.00 | 1.99 | up |
| HORVU.MOREX.r2.7HG0612690 | 2512.00 | 2533.00 | 2359.00 | 5448.00 | 5710.00 | 6713.00 | 23.30 | 23.32 | 22.78 | 49.90 | 50.99 | 64.78 | 0.00 | 1.21 | up |
| HORVU.MOREX.r2.2HG0158400 | 12.00 | 12.00 | 8.00 | 1761.00 | 1109.00 | 1354.00 | 0.13 | 0.14 | 0.10 | 20.89 | 12.82 | 16.92 | 0.00 | 6.34 | up |
| HORVU.MOREX.r2.3HG0235080 | 182.00 | 285.00 | 218.00 | 809.00 | 1016.00 | 1194.00 | 1.48 | 2.30 | 1.84 | 6.50 | 7.95 | 10.10 | 0.00 | 2.00 | up |
| HORVU.MOREX.r2.2HG0114510 | 684.00 | 691.00 | 447.00 | 3598.00 | 1976.00 | 3116.00 | 6.03 | 6.05 | 4.10 | 31.35 | 16.79 | 28.61 | 0.00 | 2.09 | up |
| HORVU.MOREX.r2.3HG0230900 | 341.00 | 281.00 | 223.00 | 1015.00 | 985.00 | 1274.00 | 14.14 | 11.59 | 9.61 | 41.64 | 39.36 | 55.06 | 0.00 | 1.83 | up |
| HORVU.MOREX.r2.3HG0265650 | 3308.00 | 3919.00 | 2317.00 | 17776.00 | 14399.00 | 19980.00 | 85.50 | 100.57 | 62.34 | 453.75 | 358.32 | 537.37 | 0.00 | 2.32 | up |
| HORVU.MOREX.r2.6HG0489990 | 336.00 | 440.00 | 163.00 | 7706.00 | 3671.00 | 4869.00 | 9.80 | 12.75 | 4.93 | 222.40 | 103.29 | 148.06 | 0.00 | 3.67 | up |
| HORVU.MOREX.r2.7HG0531680 | 290.00 | 188.00 | 141.00 | 581.00 | 380.00 | 417.00 | 10.78 | 6.93 | 5.46 | 21.36 | 13.64 | 16.14 | 0.00 | 1.02 | up |
| HORVU.MOREX.r2.7HG0600630 | 790.00 | 652.00 | 571.00 | 2298.00 | 2118.00 | 2636.00 | 5.58 | 4.58 | 4.20 | 16.05 | 14.43 | 19.40 | 0.00 | 1.71 | up |
| HORVU.MOREX.r2.7HG0551400 | 1140.00 | 2147.00 | 1396.00 | 3707.00 | 2849.00 | 3369.00 | 20.39 | 38.14 | 26.01 | 65.51 | 49.07 | 62.71 | 0.00 | 1.00 | up |
| HORVU.MOREX.r2.6HG0516400 | 478.00 | 710.00 | 638.00 | 1163.00 | 1243.00 | 1545.00 | 2.78 | 4.11 | 3.87 | 6.69 | 6.97 | 9.37 | 0.00 | 1.03 | up |
| HORVU.MOREX.r2.4HG0343710 | 1398.00 | 2630.00 | 1397.00 | 8762.00 | 5683.00 | 7035.00 | 24.12 | 45.05 | 25.09 | 149.32 | 94.42 | 126.32 | 0.00 | 1.84 | up |
| HORVU.MOREX.r2.4HG0332780 | 339.00 | 169.00 | 37.00 | 12877.00 | 11309.00 | 13093.00 | 4.35 | 2.15 | 0.49 | 163.25 | 139.78 | 174.89 | 0.00 | 3.40 | up |
| HORVU.MOREX.r2.3HG0221260 | 1674.00 | 3425.00 | 2817.00 | 7102.00 | 5010.00 | 6086.00 | 17.64 | 35.82 | 30.90 | 73.89 | 50.81 | 66.71 | 0.00 | 1.10 | up |
| HORVU.MOREX.r2.2HG0144850 | 48.00 | 64.00 | 35.00 | 118.00 | 97.00 | 154.00 | 0.93 | 1.22 | 0.70 | 2.26 | 1.81 | 3.11 | 0.00 | 1.15 | up |
| HORVU.MOREX.r2.2HG0179320 | 0.00 | 0.00 | 0.00 | 603.00 | 262.00 | 409.00 | 0.00 | 0.00 | 0.00 | 7.85 | 3.32 | 5.60 | 0.00 | 6.30 | up |
| HORVU.MOREX.r2.2HG0113980 | 45.00 | 51.00 | 62.00 | 159.00 | 209.00 | 303.00 | 0.38 | 0.43 | 0.54 | 1.33 | 1.71 | 2.69 | 0.00 | 1.84 | up |
| HORVU.MOREX.r2.4HG0320870 | 1538.00 | 1365.00 | 1203.00 | 3284.00 | 2689.00 | 3243.00 | 27.68 | 24.39 | 22.55 | 58.38 | 46.61 | 60.75 | 0.00 | 1.10 | up |
| HORVU.MOREX.r2.2HG0143300 | 254.00 | 322.00 | 256.00 | 799.00 | 906.00 | 1120.00 | 3.34 | 4.20 | 3.51 | 10.40 | 11.50 | 15.36 | 0.00 | 1.66 | up |
| HORVU.MOREX.r2.2HG0090920 | 392.00 | 683.00 | 792.00 | 1640.00 | 1176.00 | 1794.00 | 5.48 | 9.47 | 11.52 | 22.63 | 15.82 | 26.09 | 0.00 | 1.17 | up |
| HORVU.MOREX.r2.7HG0545310 | 4319.00 | 5650.00 | 4683.00 | 10649.00 | 14341.00 | 16606.00 | 48.68 | 63.22 | 54.95 | 118.52 | 155.62 | 194.74 | 0.00 | 1.42 | up |
| HORVU.MOREX.r2.3HG0236250 | 29.00 | 18.00 | 25.00 | 512.00 | 256.00 | 471.00 | 0.78 | 0.49 | 0.69 | 13.93 | 6.77 | 13.51 | 0.00 | 3.58 | up |
| HORVU.MOREX.r2.7HG0552530 | 509.00 | 292.00 | 326.00 | 3104.00 | 2295.00 | 2874.00 | 10.03 | 5.71 | 6.68 | 60.43 | 43.54 | 58.95 | 0.00 | 2.67 | up |
| HORVU.MOREX.r2.2HG0169030 | 73.00 | 34.00 | 16.00 | 180.00 | 163.00 | 164.00 | 6.01 | 2.76 | 1.38 | 14.74 | 12.97 | 14.16 | 0.00 | 1.63 | up |
| HORVU.MOREX.r2.5HG0350340 | 80.00 | 122.00 | 85.00 | 316.00 | 223.00 | 245.00 | 2.85 | 4.31 | 3.12 | 11.13 | 7.64 | 9.06 | 0.00 | 1.32 | up |
| HORVU.MOREX.r2.3HG0268970 | 901.00 | 592.00 | 494.00 | 5719.00 | 5449.00 | 8106.00 | 7.60 | 4.96 | 4.34 | 47.67 | 44.28 | 71.19 | 0.00 | 3.04 | up |
| HORVU.MOREX.r2.1HG0022190 | 35.00 | 30.00 | 33.00 | 70.00 | 88.00 | 82.00 | 0.56 | 0.48 | 0.56 | 1.13 | 1.39 | 1.39 | 0.00 | 1.12 | up |
| HORVU.MOREX.r2.1HG0063880 | 2.00 | 1.00 | 2.00 | 125.00 | 91.00 | 145.00 | 0.04 | 0.02 | 0.02 | 2.62 | 1.85 | 3.22 | 0.00 | 4.35 | up |
| HORVU.MOREX.r2.2HG0098620 | 199.00 | 236.00 | 134.00 | 726.00 | 467.00 | 788.00 | 2.12 | 2.49 | 1.48 | 7.65 | 4.80 | 8.74 | 0.00 | 1.64 | up |
| HORVU.MOREX.r2.4HG0343900 | 32778.00 | 27464.00 | 21610.00 | 42789.00 | 56278.00 | 91602.00 | 308.47 | 256.62 | 211.72 | 397.66 | 509.89 | 896.97 | 0.00 | 1.10 | up |
| HORVU.MOREX.r2.1HG0073300 | 97.00 | 148.00 | 120.00 | 1063.00 | 737.00 | 923.00 | 0.68 | 1.03 | 0.88 | 7.39 | 4.99 | 6.76 | 0.00 | 2.72 | up |
| HORVU.MOREX.r2.4HG0283680 | 110.00 | 134.00 | 69.00 | 776.00 | 397.00 | 700.00 | 7.43 | 8.98 | 4.80 | 51.80 | 25.84 | 49.24 | 0.00 | 2.32 | up |
| HORVU.MOREX.r2.5HG0356200 | 7358.00 | 10202.00 | 8584.00 | 24114.00 | 22036.00 | 33872.00 | 403.70 | 555.78 | 490.34 | 1306.58 | 1164.00 | 1933.69 | 0.00 | 1.52 | up |
| HORVU.MOREX.r2.5HG0443530 | 18.00 | 2.00 | 3.00 | 168.00 | 187.00 | 187.00 | 0.35 | 0.04 | 0.06 | 3.26 | 3.53 | 3.83 | 0.00 | 3.36 | up |
| HORVU.MOREX.r2.4HG0319040 | 256.00 | 382.00 | 342.00 | 724.00 | 705.00 | 735.00 | 10.71 | 15.84 | 14.88 | 29.89 | 28.39 | 32.00 | 0.00 | 1.07 | up |
| HORVU.MOREX.r2.3HG0255740 | 1453.00 | 1493.00 | 1179.00 | 3166.00 | 4609.00 | 4626.00 | 28.64 | 29.22 | 24.20 | 61.64 | 87.47 | 94.89 | 0.00 | 1.50 | up |
| HORVU.MOREX.r2.2HG0165880 | 208.00 | 300.00 | 324.00 | 817.00 | 738.00 | 931.00 | 1.42 | 2.04 | 2.31 | 5.52 | 4.87 | 6.64 | 0.00 | 1.47 | up |
| HORVU.MOREX.r2.4HG0288970 | 189.00 | 89.00 | 49.00 | 917.00 | 660.00 | 1068.00 | 9.73 | 4.51 | 2.62 | 46.58 | 32.68 | 57.17 | 0.00 | 2.55 | up |
| Hordeum_vulgare_newGene_7624 | 187.00 | 600.00 | 597.00 | 1836.00 | 1531.00 | 2784.00 | 4.25 | 13.56 | 14.15 | 41.28 | 33.56 | 65.98 | 0.00 | 1.61 | up |
| HORVU.MOREX.r2.4HG0342500 | 613.00 | 689.00 | 499.00 | 1384.00 | 1137.00 | 1449.00 | 15.10 | 16.84 | 12.79 | 33.68 | 26.96 | 37.15 | 0.00 | 1.07 | up |
| HORVU.MOREX.r2.3HG0266100 | 204.00 | 180.00 | 178.00 | 6657.00 | 6875.00 | 9519.00 | 3.45 | 3.02 | 3.14 | 111.47 | 112.23 | 167.94 | 0.00 | 5.12 | up |
| HORVU.MOREX.r2.3HG0237080 | 164.00 | 226.00 | 115.00 | 580.00 | 544.00 | 556.00 | 1.87 | 2.58 | 1.37 | 6.57 | 6.01 | 6.64 | 0.00 | 1.61 | up |
| Hordeum_vulgare_newGene_5574 | 35.00 | 21.00 | 99.00 | 215.00 | 137.00 | 170.00 | 1.08 | 0.58 | 3.40 | 7.14 | 4.30 | 5.87 | 0.01 | 1.31 | up |
| HORVU.MOREX.r2.2HG0139280 | 112.00 | 78.00 | 65.00 | 269.00 | 136.00 | 186.00 | 1.35 | 0.94 | 0.81 | 3.21 | 1.58 | 2.34 | 0.01 | 1.04 | up |
| HORVU.MOREX.r2.3HG0263420 | 1308.00 | 1453.00 | 1101.00 | 2888.00 | 2636.00 | 2558.00 | 22.10 | 24.38 | 19.37 | 48.22 | 42.90 | 44.98 | 0.00 | 1.01 | up |
| HORVU.MOREX.r2.1HG0044100 | 405.00 | 787.00 | 572.00 | 3536.00 | 2353.00 | 2841.00 | 4.88 | 9.42 | 7.19 | 42.13 | 27.33 | 35.66 | 0.00 | 2.15 | up |
| HORVU.MOREX.r2.1HG0040480 | 559.00 | 106.00 | 87.00 | 2003.00 | 2326.00 | 2556.00 | 13.60 | 2.54 | 2.19 | 48.12 | 54.49 | 64.70 | 0.00 | 1.81 | up |
| HORVU.MOREX.r2.6HG0505130 | 161.00 | 87.00 | 54.00 | 328.00 | 205.00 | 218.00 | 6.28 | 3.35 | 2.16 | 12.63 | 7.70 | 8.83 | 0.01 | 1.10 | up |
| HORVU.MOREX.r2.UnG0625330 | 44.00 | 112.00 | 37.00 | 199.00 | 131.00 | 192.00 | 0.31 | 0.78 | 0.27 | 1.40 | 0.89 | 1.41 | 0.01 | 1.20 | up |
| HORVU.MOREX.r2.5HG0410210 | 52.00 | 28.00 | 20.00 | 236.00 | 158.00 | 156.00 | 0.40 | 0.22 | 0.16 | 1.85 | 1.20 | 1.28 | 0.00 | 2.08 | up |
| HORVU.MOREX.r2.7HG0585930 | 17.00 | 18.00 | 41.00 | 130.00 | 101.00 | 299.00 | 0.44 | 0.48 | 1.12 | 3.41 | 2.58 | 8.30 | 0.00 | 2.14 | up |
| HORVU.MOREX.r2.5HG0354550 | 214.00 | 221.00 | 80.00 | 793.00 | 583.00 | 817.00 | 1.52 | 1.57 | 0.59 | 5.58 | 4.00 | 6.06 | 0.00 | 1.85 | up |
| HORVU.MOREX.r2.5HG0392060 | 69.00 | 96.00 | 71.00 | 284.00 | 332.00 | 490.00 | 0.62 | 0.85 | 0.66 | 2.53 | 2.88 | 4.60 | 0.00 | 2.03 | up |
| HORVU.MOREX.r2.7HG0611940 | 428.00 | 748.00 | 443.00 | 2328.00 | 1756.00 | 1618.00 | 8.69 | 15.09 | 9.37 | 46.74 | 34.36 | 34.22 | 0.00 | 1.69 | up |
| HORVU.MOREX.r2.2HG0089560 | 82.00 | 150.00 | 93.00 | 270.00 | 273.00 | 266.00 | 0.69 | 1.25 | 0.82 | 2.25 | 2.21 | 2.33 | 0.00 | 1.20 | up |
| HORVU.MOREX.r2.1HG0060010 | 497.00 | 309.00 | 210.00 | 1987.00 | 1507.00 | 1765.00 | 5.45 | 3.36 | 2.39 | 21.53 | 15.91 | 20.14 | 0.00 | 2.16 | up |
| HORVU.MOREX.r2.6HG0511800 | 4.00 | 25.00 | 14.00 | 575.00 | 306.00 | 1115.00 | 0.04 | 0.30 | 0.17 | 6.79 | 3.52 | 13.88 | 0.00 | 3.07 | up |
| HORVU.MOREX.r2.5HG0437960 | 40.00 | 49.00 | 34.00 | 216.00 | 118.00 | 188.00 | 0.24 | 0.30 | 0.21 | 1.30 | 0.69 | 1.20 | 0.00 | 1.83 | up |
| HORVU.MOREX.r2.7HG0610260 | 1058.00 | 629.00 | 694.00 | 2530.00 | 2315.00 | 2496.00 | 15.08 | 8.89 | 10.29 | 35.61 | 31.76 | 37.02 | 0.00 | 1.51 | up |
| HORVU.MOREX.r2.3HG0268980 | 239.00 | 136.00 | 107.00 | 1245.00 | 1128.00 | 1609.00 | 11.08 | 6.26 | 5.15 | 57.20 | 50.50 | 77.85 | 0.00 | 2.76 | up |
| HORVU.MOREX.r2.6HG0505440 | 9.00 | 6.00 | 12.00 | 143.00 | 96.00 | 59.00 | 0.13 | 0.08 | 0.19 | 2.14 | 1.39 | 0.92 | 0.00 | 2.68 | up |
| HORVU.MOREX.r2.4HG0337410 | 439.00 | 562.00 | 841.00 | 2469.00 | 1949.00 | 1743.00 | 23.59 | 30.01 | 47.11 | 131.29 | 101.00 | 97.64 | 0.00 | 1.58 | up |
| HORVU.MOREX.r2.4HG0341130 | 135.00 | 54.00 | 21.00 | 1232.00 | 936.00 | 1111.00 | 1.15 | 0.45 | 0.18 | 10.39 | 7.70 | 9.87 | 0.00 | 2.56 | up |
| HORVU.MOREX.r2.4HG0332820 | 1143.00 | 2672.00 | 1828.00 | 13889.00 | 8751.00 | 12539.00 | 17.27 | 40.06 | 28.75 | 207.19 | 127.27 | 197.08 | 0.00 | 2.42 | up |
| HORVU.MOREX.r2.1HG0061590 | 1778.00 | 1507.00 | 910.00 | 3539.00 | 3454.00 | 4243.00 | 46.57 | 39.18 | 24.82 | 91.56 | 87.10 | 115.65 | 0.00 | 1.31 | up |
| HORVU.MOREX.r2.2HG0108330 | 44.00 | 99.00 | 53.00 | 187.00 | 115.00 | 195.00 | 0.25 | 0.55 | 0.31 | 1.05 | 0.63 | 1.16 | 0.00 | 1.15 | up |
| HORVU.MOREX.r2.7HG0621450 | 89.00 | 50.00 | 68.00 | 312.00 | 134.00 | 264.00 | 1.62 | 0.90 | 1.28 | 5.62 | 2.35 | 5.00 | 0.00 | 1.50 | up |
| HORVU.MOREX.r2.3HG0193470 | 89.00 | 125.00 | 156.00 | 1098.00 | 702.00 | 952.00 | 2.33 | 3.26 | 4.28 | 28.64 | 17.86 | 26.16 | 0.00 | 2.66 | up |
| HORVU.MOREX.r2.5HG0447820 | 493.00 | 823.00 | 435.00 | 21491.00 | 11184.00 | 14840.00 | 1.96 | 3.25 | 1.80 | 84.43 | 42.83 | 61.42 | 0.00 | 4.40 | up |
| HORVU.MOREX.r2.1HG0016070 | 6.00 | 73.00 | 22.00 | 56680.00 | 31543.00 | 55679.00 | 0.05 | 0.71 | 0.22 | 551.76 | 299.34 | 571.08 | 0.00 | 3.92 | up |
| HORVU.MOREX.r2.3HG0263070 | 195.00 | 171.00 | 161.00 | 574.00 | 387.00 | 481.00 | 3.90 | 3.40 | 3.36 | 11.36 | 7.46 | 10.03 | 0.00 | 1.34 | up |
| HORVU.MOREX.r2.2HG0100990 | 65.00 | 113.00 | 57.00 | 576.00 | 349.00 | 633.00 | 3.55 | 6.19 | 3.27 | 31.47 | 18.58 | 36.47 | 0.00 | 2.44 | up |
| HORVU.MOREX.r2.7HG0595660 | 818.00 | 719.00 | 461.00 | 2181.00 | 2416.00 | 2479.00 | 3.14 | 2.74 | 1.84 | 8.26 | 8.92 | 9.89 | 0.00 | 1.71 | up |
| HORVU.MOREX.r2.2HG0176250 | 1267.00 | 1177.00 | 706.00 | 4615.00 | 3228.00 | 4261.00 | 14.53 | 13.41 | 8.43 | 52.27 | 35.65 | 50.85 | 0.00 | 1.81 | up |
| HORVU.MOREX.r2.1HG0063430 | 91.00 | 131.00 | 74.00 | 657.00 | 382.00 | 363.00 | 1.15 | 1.65 | 0.97 | 8.23 | 4.66 | 4.79 | 0.00 | 2.02 | up |
| HORVU.MOREX.r2.4HG0342150 | 627.00 | 460.00 | 401.00 | 1191.00 | 959.00 | 1032.00 | 6.63 | 4.83 | 4.41 | 12.45 | 9.77 | 11.37 | 0.00 | 1.01 | up |
| HORVU.MOREX.r2.6HG0526370 | 150.00 | 95.00 | 110.00 | 1528.00 | 813.00 | 1409.00 | 2.09 | 1.30 | 1.58 | 20.99 | 10.88 | 20.39 | 0.00 | 3.09 | up |
| HORVU.MOREX.r2.1HG0040640 | 688.00 | 1193.00 | 874.00 | 1826.00 | 1993.00 | 2154.00 | 16.87 | 29.04 | 22.31 | 44.24 | 47.08 | 54.99 | 0.00 | 1.04 | up |
| HORVU.MOREX.r2.3HG0244060 | 490.00 | 665.00 | 571.00 | 1678.00 | 1540.00 | 1361.00 | 2.95 | 3.97 | 3.57 | 9.97 | 8.93 | 8.52 | 0.00 | 1.33 | up |
| HORVU.MOREX.r2.7HG0610210 | 117.00 | 83.00 | 83.00 | 158.00 | 245.00 | 237.00 | 2.19 | 1.55 | 1.62 | 2.94 | 4.44 | 4.66 | 0.00 | 1.04 | up |
| HORVU.MOREX.r2.1HG0066900 | 153.00 | 250.00 | 124.00 | 495.00 | 318.00 | 481.00 | 1.74 | 2.83 | 1.46 | 5.57 | 3.49 | 5.70 | 0.00 | 1.17 | up |
| HORVU.MOREX.r2.1HG0051890 | 390.00 | 288.00 | 371.00 | 866.00 | 689.00 | 730.00 | 3.52 | 2.58 | 3.49 | 7.72 | 5.99 | 6.86 | 0.00 | 1.03 | up |
| HORVU.MOREX.r2.6HG0512370 | 2171.00 | 3062.00 | 1982.00 | 5414.00 | 4996.00 | 5858.00 | 21.95 | 30.74 | 20.86 | 54.07 | 48.65 | 61.65 | 0.00 | 1.11 | up |
| HORVU.MOREX.r2.1HG0038570 | 463.00 | 430.00 | 419.00 | 923.00 | 879.00 | 1166.00 | 3.25 | 2.99 | 3.06 | 6.39 | 5.93 | 8.51 | 0.00 | 1.10 | up |
| HORVU.MOREX.r2.7HG0601740 | 132.00 | 37.00 | 36.00 | 235.00 | 211.00 | 281.00 | 1.70 | 0.47 | 0.47 | 3.00 | 2.62 | 3.78 | 0.00 | 1.44 | up |
| Hordeum_vulgare_newGene_1040 | 45.00 | 36.00 | 44.00 | 97.00 | 95.00 | 157.00 | 0.83 | 0.66 | 0.84 | 1.79 | 1.69 | 3.05 | 0.00 | 1.27 | up |
| HORVU.MOREX.r2.2HG0080880 | 4.00 | 11.00 | 17.00 | 183.00 | 147.00 | 194.00 | 0.05 | 0.12 | 0.21 | 2.12 | 1.66 | 2.37 | 0.00 | 3.34 | up |
| HORVU.MOREX.r2.7HG0529600 | 161.00 | 78.00 | 68.00 | 331.00 | 377.00 | 587.00 | 1.51 | 0.72 | 0.66 | 3.07 | 3.41 | 5.74 | 0.00 | 1.78 | up |
| HORVU.MOREX.r2.5HG0393330 | 533.00 | 647.00 | 501.00 | 1234.00 | 962.00 | 1506.00 | 3.05 | 3.67 | 2.98 | 6.97 | 5.30 | 8.97 | 0.00 | 1.06 | up |
| HORVU.MOREX.r2.7HG0538260 | 325.00 | 739.00 | 449.00 | 1898.00 | 1587.00 | 2017.00 | 2.83 | 6.39 | 4.07 | 16.34 | 13.32 | 18.30 | 0.00 | 1.71 | up |
| HORVU.MOREX.r2.5HG0389740 | 60.00 | 70.00 | 91.00 | 117.00 | 170.00 | 218.00 | 0.45 | 0.51 | 0.71 | 0.87 | 1.22 | 1.69 | 0.01 | 1.03 | up |
| HORVU.MOREX.r2.1HG0022060 | 859.00 | 778.00 | 565.00 | 1791.00 | 1653.00 | 2087.00 | 11.51 | 10.35 | 7.88 | 23.69 | 21.32 | 29.09 | 0.00 | 1.24 | up |
| HORVU.MOREX.r2.4HG0328730 | 173.00 | 437.00 | 331.00 | 731.00 | 746.00 | 1104.00 | 3.03 | 7.59 | 6.02 | 12.63 | 12.57 | 20.11 | 0.00 | 1.29 | up |
| HORVU.MOREX.r2.3HG0194250 | 180.00 | 157.00 | 71.00 | 366.00 | 284.00 | 676.00 | 14.49 | 12.55 | 5.90 | 29.17 | 22.06 | 56.81 | 0.00 | 1.44 | up |
| HORVU.MOREX.r2.3HG0263620 | 50.00 | 121.00 | 66.00 | 570.00 | 169.00 | 333.00 | 0.49 | 1.18 | 0.67 | 5.55 | 1.60 | 3.41 | 0.00 | 1.78 | up |
| HORVU.MOREX.r2.1HG0047780 | 7.00 | 0.00 | 0.00 | 770.00 | 547.00 | 724.00 | 0.24 | 0.00 | 0.00 | 27.12 | 18.78 | 26.85 | 0.01 | 1.54 | up |
| HORVU.MOREX.r2.3HG0268900 | 77.00 | 74.00 | 120.00 | 323.00 | 138.00 | 374.00 | 2.92 | 2.79 | 4.72 | 12.12 | 5.05 | 14.75 | 0.00 | 1.35 | up |
| HORVU.MOREX.r2.7HG0607610 | 612.00 | 922.00 | 960.00 | 2314.00 | 1857.00 | 2450.00 | 28.68 | 42.91 | 46.83 | 107.16 | 83.82 | 119.52 | 0.00 | 1.31 | up |
| HORVU.MOREX.r2.3HG0224430 | 1053.00 | 524.00 | 366.00 | 3483.00 | 1865.00 | 3105.00 | 4.22 | 2.08 | 1.53 | 13.78 | 7.19 | 12.94 | 0.00 | 1.62 | up |
| HORVU.MOREX.r2.2HG0100030 | 629.00 | 409.00 | 356.00 | 3340.00 | 2637.00 | 3094.00 | 7.10 | 4.58 | 4.18 | 37.25 | 28.67 | 36.35 | 0.00 | 2.53 | up |
| HORVU.MOREX.r2.2HG0109510 | 123.00 | 206.00 | 121.00 | 520.00 | 476.00 | 678.00 | 1.00 | 1.68 | 1.03 | 4.21 | 3.76 | 5.79 | 0.00 | 1.75 | up |
| HORVU.MOREX.r2.1HG0042350 | 2008.00 | 1584.00 | 1416.00 | 5117.00 | 3941.00 | 4836.00 | 32.62 | 25.54 | 23.95 | 82.09 | 61.63 | 81.74 | 0.00 | 1.38 | up |
| HORVU.MOREX.r2.2HG0145130 | 43.00 | 27.00 | 23.00 | 7215.00 | 3946.00 | 5639.00 | 0.72 | 0.44 | 0.39 | 120.11 | 64.03 | 98.90 | 0.00 | 6.87 | up |
| HORVU.MOREX.r2.3HG0235690 | 31.00 | 31.00 | 17.00 | 93.00 | 55.00 | 70.00 | 0.52 | 0.51 | 0.30 | 1.57 | 0.91 | 1.25 | 0.01 | 1.21 | up |
| HORVU.MOREX.r2.2HG0132470 | 122.00 | 165.00 | 109.00 | 299.00 | 321.00 | 274.00 | 1.20 | 1.61 | 1.11 | 2.90 | 3.04 | 2.80 | 0.00 | 1.09 | up |
| HORVU.MOREX.r2.1HG0039310 | 152.00 | 77.00 | 63.00 | 516.00 | 329.00 | 1679.00 | 1.91 | 0.96 | 0.82 | 6.41 | 3.99 | 21.98 | 0.00 | 1.85 | up |
| HORVU.MOREX.r2.2HG0154140 | 1550.00 | 1622.00 | 1431.00 | 50452.00 | 28012.00 | 30794.00 | 26.43 | 27.46 | 25.40 | 849.76 | 459.96 | 546.48 | 0.00 | 4.32 | up |
| HORVU.MOREX.r2.5HG0439330 | 144.00 | 172.00 | 64.00 | 374.00 | 362.00 | 473.00 | 1.73 | 2.06 | 0.80 | 4.46 | 4.21 | 5.94 | 0.00 | 1.48 | up |
| HORVU.MOREX.r2.1HG0016800 | 2.00 | 0.00 | 0.00 | 1927.00 | 671.00 | 1396.00 | 0.05 | 0.00 | 0.00 | 49.18 | 16.68 | 37.54 | 0.00 | 5.48 | up |
| HORVU.MOREX.r2.2HG0161570 | 85.00 | 53.00 | 69.00 | 668.00 | 443.00 | 572.00 | 1.34 | 0.82 | 1.12 | 10.41 | 6.73 | 9.40 | 0.00 | 2.77 | up |
| HORVU.MOREX.r2.5HG0357880 | 933.00 | 1744.00 | 1247.00 | 3467.00 | 2807.00 | 3995.00 | 7.29 | 13.53 | 10.15 | 26.76 | 21.12 | 32.49 | 0.00 | 1.29 | up |
| HORVU.MOREX.r2.7HG0620410 | 39.00 | 51.00 | 29.00 | 109.00 | 131.00 | 135.00 | 0.64 | 0.83 | 0.50 | 1.78 | 2.10 | 2.33 | 0.00 | 1.47 | up |
| HORVU.MOREX.r2.2HG0160760 | 57.00 | 88.00 | 67.00 | 391.00 | 528.00 | 912.00 | 3.07 | 4.75 | 3.81 | 21.17 | 27.87 | 52.01 | 0.00 | 2.76 | up |
| HORVU.MOREX.r2.2HG0096010 | 522.00 | 733.00 | 509.00 | 1292.00 | 1468.00 | 1618.00 | 3.51 | 4.89 | 3.56 | 8.58 | 9.50 | 11.32 | 0.00 | 1.24 | up |
| HORVU.MOREX.r2.3HG0186920 | 1421.00 | 1097.00 | 910.00 | 3587.00 | 3880.00 | 5687.00 | 11.65 | 8.93 | 7.77 | 29.05 | 30.64 | 48.53 | 0.00 | 1.80 | up |
| HORVU.MOREX.r2.1HG0066420 | 42.00 | 26.00 | 31.00 | 98.00 | 115.00 | 128.00 | 0.32 | 0.19 | 0.25 | 0.75 | 0.86 | 1.03 | 0.00 | 1.55 | up |
| HORVU.MOREX.r2.2HG0080910 | 8.00 | 4.00 | 7.00 | 252.00 | 212.00 | 186.00 | 0.08 | 0.04 | 0.08 | 2.81 | 2.30 | 2.18 | 0.00 | 4.33 | up |
| HORVU.MOREX.r2.2HG0135180 | 81.00 | 44.00 | 23.00 | 1066.00 | 498.00 | 638.00 | 2.69 | 1.43 | 0.77 | 35.17 | 15.99 | 22.18 | 0.00 | 3.28 | up |
| HORVU.MOREX.r2.5HG0407280 | 1937.00 | 1791.00 | 1343.00 | 3731.00 | 3751.00 | 4108.00 | 7.87 | 7.23 | 5.68 | 14.97 | 14.67 | 17.37 | 0.00 | 1.12 | up |
| HORVU.MOREX.r2.6HG0515810 | 905.00 | 1302.00 | 842.00 | 2149.00 | 2925.00 | 3220.00 | 5.01 | 7.15 | 4.85 | 11.74 | 15.58 | 18.54 | 0.00 | 1.35 | up |
| HORVU.MOREX.r2.5HG0422960 | 4290.00 | 3649.00 | 3469.00 | 10877.00 | 10665.00 | 13993.00 | 81.79 | 69.08 | 68.86 | 204.82 | 195.78 | 277.63 | 0.00 | 1.55 | up |
| HORVU.MOREX.r2.3HG0269840 | 2.00 | 0.00 | 4.00 | 85.00 | 66.00 | 69.00 | 0.02 | 0.00 | 0.05 | 1.02 | 0.78 | 0.87 | 0.00 | 3.55 | up |
| Hordeum_vulgare_newGene_5697 | 12.00 | 23.00 | 37.00 | 77.00 | 75.00 | 89.00 | 0.24 | 0.46 | 0.84 | 1.59 | 1.57 | 1.89 | 0.00 | 1.39 | up |
| HORVU.MOREX.r2.4HG0326890 | 809.00 | 537.00 | 582.00 | 2422.00 | 2198.00 | 2867.00 | 7.08 | 4.66 | 5.30 | 20.95 | 18.53 | 26.13 | 0.00 | 1.83 | up |
| HORVU.MOREX.r2.6HG0462570 | 20.00 | 35.00 | 26.00 | 62.00 | 69.00 | 103.00 | 0.21 | 0.36 | 0.28 | 0.64 | 0.69 | 1.12 | 0.00 | 1.28 | up |
| HORVU.MOREX.r2.3HG0228480 | 628.00 | 547.00 | 482.00 | 1380.00 | 875.00 | 1388.00 | 4.06 | 3.51 | 3.24 | 8.81 | 5.45 | 9.34 | 0.00 | 1.04 | up |
| HORVU.MOREX.r2.4HG0328360 | 17.00 | 11.00 | 22.00 | 367.00 | 217.00 | 306.00 | 0.24 | 0.15 | 0.33 | 5.29 | 3.05 | 4.64 | 0.00 | 3.61 | up |
| HORVU.MOREX.r2.2HG0175700 | 120.00 | 217.00 | 96.00 | 1391.00 | 876.00 | 1052.00 | 1.00 | 1.80 | 0.84 | 11.51 | 7.07 | 9.17 | 0.00 | 2.68 | up |
| HORVU.MOREX.r2.5HG0429310 | 72.00 | 18.00 | 17.00 | 2429.00 | 2110.00 | 4203.00 | 1.86 | 0.45 | 0.43 | 62.54 | 52.96 | 114.06 | 0.00 | 3.80 | up |
| HORVU.MOREX.r2.1HG0038230 | 24.00 | 58.00 | 67.00 | 1122.00 | 370.00 | 736.00 | 0.23 | 0.55 | 0.67 | 10.70 | 3.43 | 7.39 | 0.00 | 3.24 | up |
| HORVU.MOREX.r2.1HG0073890 | 0.00 | 4.00 | 19.00 | 6805.00 | 4000.00 | 4247.00 | 0.00 | 0.06 | 0.28 | 97.71 | 55.99 | 64.24 | 0.00 | 1.84 | up |
| HORVU.MOREX.r2.7HG0537070 | 30.00 | 75.00 | 30.00 | 3154.00 | 1246.00 | 1530.00 | 0.38 | 0.96 | 0.40 | 40.34 | 15.53 | 20.62 | 0.00 | 4.01 | up |
| HORVU.MOREX.r2.1HG0038650 | 4145.00 | 4733.00 | 4332.00 | 11670.00 | 8554.00 | 10424.00 | 47.36 | 53.69 | 51.53 | 131.68 | 94.09 | 123.93 | 0.00 | 1.15 | up |
| HORVU.MOREX.r2.6HG0458320 | 215.00 | 211.00 | 154.00 | 514.00 | 534.00 | 631.00 | 3.21 | 3.13 | 2.39 | 7.58 | 7.68 | 9.81 | 0.00 | 1.44 | up |
| HORVU.MOREX.r2.5HG0403460 | 276.00 | 259.00 | 161.00 | 481.00 | 564.00 | 610.00 | 4.94 | 4.61 | 3.00 | 8.52 | 9.73 | 11.39 | 0.00 | 1.15 | up |
| HORVU.MOREX.r2.1HG0036030 | 141.00 | 174.00 | 124.00 | 417.00 | 424.00 | 424.00 | 3.50 | 4.29 | 3.20 | 10.26 | 10.18 | 11.01 | 0.00 | 1.44 | up |
| HORVU.MOREX.r2.4HG0333650 | 1011.00 | 805.00 | 568.00 | 2804.00 | 3142.00 | 3747.00 | 14.47 | 11.44 | 8.46 | 39.66 | 43.33 | 55.84 | 0.00 | 1.89 | up |
| HORVU.MOREX.r2.2HG0092470 | 212.00 | 102.00 | 54.00 | 778.00 | 838.00 | 945.00 | 3.91 | 1.86 | 1.04 | 14.18 | 14.89 | 18.14 | 0.00 | 2.39 | up |
| HORVU.MOREX.r2.4HG0280940 | 0.00 | 3.00 | 1.00 | 170.00 | 98.00 | 121.00 | 0.00 | 0.12 | 0.04 | 7.49 | 4.21 | 5.62 | 0.00 | 4.48 | up |
| HORVU.MOREX.r2.5HG0438980 | 478.00 | 657.00 | 492.00 | 1313.00 | 1296.00 | 1989.00 | 5.97 | 8.16 | 6.40 | 16.21 | 15.60 | 25.88 | 0.00 | 1.40 | up |
| HORVU.MOREX.r2.6HG0499150 | 33.00 | 53.00 | 59.00 | 356.00 | 168.00 | 272.00 | 0.29 | 0.47 | 0.54 | 3.13 | 1.44 | 2.51 | 0.00 | 2.13 | up |
| HORVU.MOREX.r2.4HG0280990 | 89.00 | 69.00 | 87.00 | 456.00 | 476.00 | 1066.00 | 4.21 | 3.25 | 4.28 | 21.44 | 21.84 | 52.83 | 0.00 | 2.64 | up |
| HORVU.MOREX.r2.7HG0587860 | 3031.00 | 1527.00 | 1355.00 | 3332.00 | 5358.00 | 6489.00 | 45.66 | 22.84 | 21.24 | 49.58 | 77.72 | 101.73 | 0.00 | 1.19 | up |
| HORVU.MOREX.r2.5HG0438640 | 473.00 | 817.00 | 837.00 | 1988.00 | 1218.00 | 1991.00 | 11.26 | 19.31 | 20.76 | 46.78 | 27.94 | 49.36 | 0.00 | 1.16 | up |
| HORVU.MOREX.r2.3HG0218650 | 2504.00 | 2866.00 | 2422.00 | 7196.00 | 5972.00 | 5005.00 | 11.47 | 13.04 | 11.56 | 32.57 | 26.35 | 23.87 | 0.00 | 1.15 | up |
| HORVU.MOREX.r2.1HG0044730 | 585.00 | 593.00 | 503.00 | 1520.00 | 1642.00 | 1699.00 | 4.02 | 4.05 | 3.60 | 10.33 | 10.87 | 12.17 | 0.00 | 1.46 | up |
| Hordeum_vulgare_newGene_1804 | 12.00 | 11.00 | 12.00 | 145.00 | 74.00 | 111.00 | 0.19 | 0.17 | 0.21 | 2.42 | 1.21 | 1.95 | 0.00 | 2.66 | up |
| HORVU.MOREX.r2.5HG0410760 | 7934.00 | 8050.00 | 6996.00 | 22864.00 | 17826.00 | 19195.00 | 89.77 | 90.44 | 82.41 | 255.48 | 194.19 | 225.98 | 0.00 | 1.31 | up |
| HORVU.MOREX.r2.3HG0227130 | 82.00 | 135.00 | 87.00 | 282.00 | 189.00 | 300.00 | 3.62 | 5.91 | 4.01 | 12.34 | 8.07 | 13.85 | 0.00 | 1.20 | up |
| HORVU.MOREX.r2.1HG0059960 | 276.00 | 265.00 | 136.00 | 756.00 | 520.00 | 635.00 | 2.67 | 2.54 | 1.37 | 7.23 | 4.85 | 6.40 | 0.00 | 1.36 | up |
| HORVU.MOREX.r2.1HG0037480 | 354.00 | 392.00 | 271.00 | 1044.00 | 655.00 | 904.00 | 5.44 | 5.98 | 4.33 | 15.86 | 9.70 | 14.46 | 0.00 | 1.26 | up |
| HORVU.MOREX.r2.2HG0091860 | 33.00 | 0.00 | 7.00 | 112.00 | 77.00 | 130.00 | 0.55 | 0.00 | 0.11 | 1.83 | 1.23 | 2.24 | 0.01 | 1.60 | up |
| HORVU.MOREX.r2.5HG0353010 | 31.00 | 44.00 | 35.00 | 91.00 | 82.00 | 116.00 | 2.48 | 3.45 | 2.93 | 7.20 | 6.34 | 9.67 | 0.00 | 1.21 | up |
| HORVU.MOREX.r2.6HG0455510 | 3407.00 | 4497.00 | 3496.00 | 10827.00 | 10265.00 | 15169.00 | 53.98 | 70.75 | 57.67 | 169.43 | 156.60 | 250.11 | 0.00 | 1.58 | up |
| HORVU.MOREX.r2.2HG0143850 | 183.00 | 232.00 | 205.00 | 485.00 | 504.00 | 557.00 | 3.96 | 4.98 | 4.63 | 10.38 | 10.52 | 12.56 | 0.00 | 1.24 | up |
| HORVU.MOREX.r2.6HG0505640 | 559.00 | 271.00 | 519.00 | 4138.00 | 1117.00 | 2188.00 | 28.48 | 13.69 | 27.56 | 208.48 | 54.84 | 116.12 | 0.00 | 1.74 | up |
| HORVU.MOREX.r2.7HG0587270 | 3210.00 | 4886.00 | 3875.00 | 9409.00 | 8712.00 | 11197.00 | 32.98 | 49.85 | 41.45 | 95.47 | 86.18 | 119.71 | 0.00 | 1.22 | up |
| HORVU.MOREX.r2.3HG0265660 | 106.00 | 200.00 | 156.00 | 458.00 | 440.00 | 838.00 | 2.72 | 5.13 | 4.19 | 11.67 | 10.94 | 22.52 | 0.00 | 1.70 | up |
| HORVU.MOREX.r2.4HG0344500 | 236.00 | 162.00 | 129.00 | 1213.00 | 983.00 | 1191.00 | 2.42 | 1.64 | 1.37 | 12.28 | 9.70 | 12.71 | 0.00 | 2.50 | up |
| HORVU.MOREX.r2.2HG0147290 | 24.00 | 12.00 | 14.00 | 123.00 | 92.00 | 50.00 | 0.31 | 0.14 | 0.19 | 1.58 | 1.15 | 0.67 | 0.00 | 1.88 | up |
| HORVU.MOREX.r2.5HG0350050 | 32374.00 | 12385.00 | 13274.00 | 69994.00 | 137794.00 | 240870.00 | 202.02 | 76.73 | 86.24 | 431.34 | 827.83 | 1563.97 | 0.00 | 1.93 | up |
| HORVU.MOREX.r2.2HG0170780 | 13.00 | 35.00 | 10.00 | 11018.00 | 5854.00 | 8370.00 | 0.11 | 0.31 | 0.09 | 96.61 | 50.04 | 77.33 | 0.00 | 6.20 | up |
| HORVU.MOREX.r2.2HG0088540 | 238.00 | 248.00 | 177.00 | 3782.00 | 2373.00 | 3461.00 | 2.81 | 2.91 | 2.18 | 44.14 | 27.00 | 42.56 | 0.00 | 3.65 | up |
| HORVU.MOREX.r2.2HG0153500 | 780.00 | 805.00 | 666.00 | 8594.00 | 5373.00 | 5674.00 | 11.00 | 11.28 | 9.78 | 119.80 | 73.01 | 83.34 | 0.00 | 2.97 | up |
| HORVU.MOREX.r2.4HG0290030 | 14.00 | 5.00 | 0.00 | 256.00 | 285.00 | 351.00 | 0.39 | 0.13 | 0.00 | 7.14 | 7.78 | 10.34 | 0.00 | 2.29 | up |
| HORVU.MOREX.r2.3HG0237940 | 117.00 | 72.00 | 12.00 | 300.00 | 176.00 | 292.00 | 2.27 | 1.38 | 0.23 | 5.77 | 3.29 | 5.91 | 0.01 | 1.42 | up |
| HORVU.MOREX.r2.2HG0142460 | 11.00 | 14.00 | 2.00 | 81.00 | 59.00 | 85.00 | 0.13 | 0.16 | 0.02 | 0.94 | 0.66 | 1.04 | 0.00 | 2.28 | up |
| HORVU.MOREX.r2.4HG0340050 | 126.00 | 316.00 | 100.00 | 914.00 | 795.00 | 1014.00 | 5.89 | 14.68 | 4.88 | 42.33 | 35.88 | 49.44 | 0.00 | 2.05 | up |
| HORVU.MOREX.r2.1HG0021720 | 26.00 | 43.00 | 33.00 | 95.00 | 109.00 | 126.00 | 0.11 | 0.19 | 0.15 | 0.41 | 0.46 | 0.57 | 0.00 | 1.49 | up |
| HORVU.MOREX.r2.2HG0084600 | 1108.00 | 1445.00 | 1171.00 | 3159.00 | 2619.00 | 3116.00 | 7.85 | 10.17 | 8.65 | 22.12 | 17.88 | 22.99 | 0.00 | 1.19 | up |
| Hordeum_vulgare_newGene_360 | 14.00 | 28.00 | 14.00 | 123.00 | 89.00 | 72.00 | 0.23 | 0.46 | 0.24 | 2.04 | 1.44 | 1.25 | 0.00 | 1.94 | up |
| HORVU.MOREX.r2.4HG0318300 | 98.00 | 90.00 | 75.00 | 736.00 | 338.00 | 584.00 | 1.24 | 1.13 | 0.99 | 9.25 | 4.14 | 7.73 | 0.00 | 2.39 | up |
| HORVU.MOREX.r2.5HG0442260 | 135.00 | 140.00 | 111.00 | 1061.00 | 612.00 | 887.00 | 0.83 | 0.85 | 0.71 | 6.47 | 3.64 | 5.70 | 0.00 | 2.54 | up |
| HORVU.MOREX.r2.2HG0109140 | 275.00 | 261.00 | 138.00 | 549.00 | 568.00 | 645.00 | 4.92 | 4.65 | 2.56 | 9.72 | 9.81 | 12.04 | 0.00 | 1.27 | up |
| HORVU.MOREX.r2.7HG0620680 | 594.00 | 1161.00 | 536.00 | 1921.00 | 1767.00 | 2234.00 | 19.52 | 37.87 | 18.32 | 62.32 | 55.89 | 76.35 | 0.00 | 1.26 | up |
| HORVU.MOREX.r2.7HG0610690 | 17.00 | 29.00 | 27.00 | 64.00 | 59.00 | 107.00 | 0.22 | 0.37 | 0.36 | 0.82 | 0.73 | 1.45 | 0.00 | 1.35 | up |
| HORVU.MOREX.r2.6HG0526610 | 300.00 | 510.00 | 467.00 | 1109.00 | 883.00 | 758.00 | 1.83 | 3.10 | 2.97 | 6.70 | 5.20 | 4.83 | 0.00 | 1.01 | up |
| HORVU.MOREX.r2.4HG0315500 | 476.00 | 627.00 | 299.00 | 2256.00 | 1604.00 | 2064.00 | 14.26 | 18.64 | 9.31 | 66.77 | 46.27 | 64.37 | 0.00 | 1.93 | up |
| HORVU.MOREX.r2.3HG0253050 | 1615.00 | 1829.00 | 1933.00 | 5351.00 | 4012.00 | 4175.00 | 16.95 | 19.06 | 21.12 | 55.47 | 40.55 | 45.60 | 0.00 | 1.25 | up |
| HORVU.MOREX.r2.7HG0613640 | 23.00 | 75.00 | 43.00 | 195.00 | 113.00 | 95.00 | 0.54 | 1.75 | 1.04 | 4.53 | 2.55 | 2.33 | 0.01 | 1.22 | up |
| HORVU.MOREX.r2.5HG0409550 | 103.00 | 131.00 | 136.00 | 289.00 | 249.00 | 311.00 | 1.00 | 1.26 | 1.38 | 2.79 | 2.34 | 3.16 | 0.00 | 1.10 | up |
| HORVU.MOREX.r2.2HG0162590 | 63.00 | 56.00 | 24.00 | 332.00 | 275.00 | 422.00 | 0.89 | 0.78 | 0.35 | 4.67 | 3.78 | 6.27 | 0.00 | 2.50 | up |
| HORVU.MOREX.r2.7HG0605030 | 81.00 | 224.00 | 153.00 | 675.00 | 725.00 | 582.00 | 0.83 | 2.28 | 1.63 | 6.86 | 7.18 | 6.23 | 0.00 | 1.90 | up |
| HORVU.MOREX.r2.6HG0479630 | 72.00 | 59.00 | 46.00 | 205.00 | 132.00 | 230.00 | 2.91 | 2.37 | 1.92 | 8.21 | 5.15 | 9.71 | 0.00 | 1.48 | up |
| HORVU.MOREX.r2.7HG0558900 | 198.00 | 463.00 | 341.00 | 764.00 | 786.00 | 692.00 | 1.19 | 2.76 | 2.13 | 4.53 | 4.55 | 4.33 | 0.00 | 1.05 | up |
| HORVU.MOREX.r2.1HG0014880 | 302.00 | 268.00 | 176.00 | 652.00 | 578.00 | 822.00 | 5.36 | 4.73 | 3.25 | 11.45 | 9.89 | 15.20 | 0.00 | 1.34 | up |
| HORVU.MOREX.r2.3HG0245140 | 19.00 | 4.00 | 12.00 | 4175.00 | 2339.00 | 2876.00 | 0.19 | 0.04 | 0.13 | 41.27 | 22.53 | 29.95 | 0.00 | 7.08 | up |
| HORVU.MOREX.r2.3HG0199190 | 168.00 | 115.00 | 61.00 | 635.00 | 574.00 | 766.00 | 1.49 | 1.01 | 0.56 | 5.58 | 4.92 | 7.10 | 0.00 | 2.25 | up |
| HORVU.MOREX.r2.3HG0246490 | 16.00 | 51.00 | 28.00 | 155.00 | 171.00 | 145.00 | 0.10 | 0.31 | 0.18 | 0.95 | 1.02 | 0.93 | 0.00 | 1.97 | up |
| HORVU.MOREX.r2.3HG0235350 | 910.00 | 491.00 | 547.00 | 1385.00 | 1564.00 | 2086.00 | 12.05 | 6.45 | 7.54 | 18.11 | 19.94 | 28.75 | 0.00 | 1.23 | up |
| HORVU.MOREX.r2.2HG0107330 | 14.00 | 28.00 | 38.00 | 72.00 | 261.00 | 331.00 | 0.28 | 0.57 | 0.84 | 1.51 | 5.35 | 7.32 | 0.00 | 2.28 | up |
| HORVU.MOREX.r2.6HG0465610 | 95.00 | 51.00 | 30.00 | 293.00 | 194.00 | 189.00 | 1.35 | 0.72 | 0.44 | 4.15 | 2.68 | 2.81 | 0.00 | 1.62 | up |
| HORVU.MOREX.r2.3HG0262300 | 57.00 | 58.00 | 14.00 | 681.00 | 271.00 | 460.00 | 1.20 | 1.22 | 0.29 | 14.31 | 5.56 | 10.19 | 0.00 | 2.79 | up |
| HORVU.MOREX.r2.6HG0453740 | 624.00 | 391.00 | 457.00 | 1714.00 | 1419.00 | 2179.00 | 3.36 | 2.09 | 2.56 | 9.12 | 7.36 | 12.22 | 0.00 | 1.71 | up |
| HORVU.MOREX.r2.2HG0085760 | 74.00 | 22.00 | 12.00 | 176.00 | 99.00 | 142.00 | 1.20 | 0.35 | 0.20 | 2.84 | 1.55 | 2.40 | 0.01 | 1.41 | up |
| HORVU.MOREX.r2.2HG0138100 | 0.00 | 4.00 | 0.00 | 153.00 | 98.00 | 331.00 | 0.00 | 0.04 | 0.00 | 1.60 | 1.00 | 3.67 | 0.00 | 4.37 | up |
| HORVU.MOREX.r2.1HG0076510 | 22.00 | 25.00 | 16.00 | 229.00 | 120.00 | 149.00 | 0.24 | 0.27 | 0.18 | 2.44 | 1.25 | 1.67 | 0.00 | 2.56 | up |
| HORVU.MOREX.r2.7HG0533150 | 0.00 | 0.00 | 0.00 | 90.00 | 187.00 | 200.00 | 0.00 | 0.00 | 0.00 | 1.48 | 3.00 | 3.48 | 0.00 | 5.06 | up |
| HORVU.MOREX.r2.3HG0186590 | 1055.00 | 855.00 | 341.00 | 3422.00 | 2651.00 | 4137.00 | 17.17 | 13.82 | 5.78 | 55.04 | 41.58 | 70.12 | 0.00 | 1.93 | up |
| HORVU.MOREX.r2.3HG0244380 | 0.00 | 0.00 | 0.00 | 495.00 | 110.00 | 414.00 | 0.00 | 0.00 | 0.00 | 4.23 | 0.91 | 3.74 | 0.00 | 5.39 | up |
| HORVU.MOREX.r2.7HG0527840 | 13294.00 | 16565.00 | 16129.00 | 24098.00 | 42085.00 | 45290.00 | 124.30 | 153.78 | 157.01 | 222.51 | 378.84 | 440.63 | 0.00 | 1.17 | up |
| HORVU.MOREX.r2.2HG0144100 | 44.00 | 54.00 | 31.00 | 173.00 | 87.00 | 122.00 | 1.38 | 1.70 | 0.99 | 5.43 | 2.66 | 4.01 | 0.00 | 1.34 | up |
| HORVU.MOREX.r2.7HG0545660 | 48.00 | 133.00 | 108.00 | 236.00 | 263.00 | 235.00 | 0.66 | 1.84 | 1.57 | 3.25 | 3.53 | 3.41 | 0.00 | 1.17 | up |
| HORVU.MOREX.r2.6HG0474180 | 61.00 | 38.00 | 47.00 | 500.00 | 252.00 | 321.00 | 1.03 | 0.63 | 0.82 | 8.36 | 4.11 | 5.65 | 0.00 | 2.54 | up |
| HORVU.MOREX.r2.7HG0532730 | 453.00 | 371.00 | 232.00 | 2155.00 | 1506.00 | 2533.00 | 11.55 | 9.39 | 6.15 | 54.28 | 36.97 | 67.23 | 0.00 | 2.34 | up |
| HORVU.MOREX.r2.5HG0393010 | 86.00 | 104.00 | 42.00 | 235.00 | 164.00 | 244.00 | 0.58 | 0.69 | 0.29 | 1.57 | 1.06 | 1.71 | 0.00 | 1.28 | up |
| HORVU.MOREX.r2.2HG0150280 | 2780.00 | 1481.00 | 1382.00 | 3766.00 | 4919.00 | 4746.00 | 43.34 | 22.92 | 22.42 | 57.97 | 73.84 | 76.99 | 0.00 | 1.12 | up |
| Hordeum_vulgare_newGene_6949 | 259.00 | 154.00 | 168.00 | 709.00 | 741.00 | 869.00 | 8.03 | 5.07 | 5.62 | 22.79 | 22.80 | 25.95 | 0.00 | 1.84 | up |
| HORVU.MOREX.r2.3HG0260630 | 6694.00 | 5225.00 | 4207.00 | 13098.00 | 11671.00 | 13329.00 | 86.90 | 67.34 | 56.85 | 167.91 | 145.86 | 180.04 | 0.00 | 1.16 | up |
| HORVU.MOREX.r2.7HG0599500 | 4289.00 | 5434.00 | 4054.00 | 24090.00 | 17386.00 | 23187.00 | 50.69 | 63.78 | 49.89 | 281.21 | 197.86 | 285.18 | 0.00 | 2.13 | up |
| HORVU.MOREX.r2.3HG0272180 | 7941.00 | 5968.00 | 2857.00 | 116669.00 | 101211.00 | 127552.00 | 314.17 | 234.43 | 117.68 | 4558.38 | 3855.13 | 5250.84 | 0.00 | 3.98 | up |
| HORVU.MOREX.r2.4HG0334150 | 1645.00 | 1966.00 | 1507.00 | 13368.00 | 9230.00 | 14999.00 | 22.55 | 26.77 | 21.51 | 181.07 | 121.89 | 214.06 | 0.00 | 2.73 | up |
| HORVU.MOREX.r2.5HG0368520 | 550.00 | 653.00 | 476.00 | 2826.00 | 2292.00 | 2392.00 | 4.74 | 5.58 | 4.26 | 24.04 | 19.01 | 21.44 | 0.00 | 2.07 | up |
| HORVU.MOREX.r2.5HG0353110 | 104.00 | 55.00 | 47.00 | 265.00 | 131.00 | 241.00 | 1.08 | 0.56 | 0.50 | 2.72 | 1.31 | 2.60 | 0.00 | 1.36 | up |
| HORVU.MOREX.r2.7HG0569730 | 22.00 | 32.00 | 53.00 | 776.00 | 361.00 | 514.00 | 1.18 | 1.73 | 3.04 | 42.82 | 19.41 | 29.90 | 0.00 | 3.40 | up |
| HORVU.MOREX.r2.4HG0339800 | 85.00 | 60.00 | 26.00 | 172.00 | 192.00 | 203.00 | 1.81 | 1.26 | 0.57 | 3.63 | 3.94 | 4.52 | 0.00 | 1.46 | up |
| HORVU.MOREX.r2.4HG0328580 | 202.00 | 81.00 | 10.00 | 2246.00 | 2041.00 | 4108.00 | 7.19 | 2.86 | 0.35 | 79.11 | 70.10 | 152.49 | 0.00 | 2.03 | up |
| HORVU.MOREX.r2.3HG0233950 | 2219.00 | 1953.00 | 1533.00 | 4753.00 | 5729.00 | 5858.00 | 25.34 | 22.15 | 18.23 | 53.62 | 63.02 | 69.64 | 0.00 | 1.44 | up |
| HORVU.MOREX.r2.1HG0040630 | 1284.00 | 617.00 | 348.00 | 7339.00 | 6067.00 | 8928.00 | 31.37 | 14.96 | 8.84 | 177.09 | 142.73 | 226.99 | 0.00 | 2.46 | up |
| HORVU.MOREX.r2.2HG0099630 | 346.00 | 287.00 | 213.00 | 832.00 | 566.00 | 683.00 | 3.21 | 2.64 | 2.05 | 7.62 | 5.05 | 6.59 | 0.00 | 1.19 | up |
| HORVU.MOREX.r2.6HG0515190 | 133.00 | 103.00 | 48.00 | 187.00 | 265.00 | 246.00 | 1.56 | 1.20 | 0.59 | 2.17 | 3.00 | 3.01 | 0.00 | 1.11 | up |
| HORVU.MOREX.r2.2HG0086780 | 1119.00 | 1000.00 | 842.00 | 2501.00 | 1774.00 | 2048.00 | 9.80 | 8.69 | 7.67 | 21.63 | 14.95 | 18.66 | 0.00 | 1.02 | up |
| HORVU.MOREX.r2.5HG0396840 | 175.00 | 202.00 | 113.00 | 2505.00 | 1660.00 | 2186.00 | 7.20 | 8.24 | 4.84 | 102.01 | 65.88 | 93.80 | 0.00 | 3.47 | up |
| HORVU.MOREX.r2.2HG0143070 | 1753.00 | 1325.00 | 840.00 | 3874.00 | 3462.00 | 5175.00 | 28.32 | 21.24 | 14.12 | 61.79 | 53.84 | 86.98 | 0.00 | 1.53 | up |
| HORVU.MOREX.r2.7HG0618470 | 51.00 | 12.00 | 27.00 | 155.00 | 142.00 | 254.00 | 0.58 | 0.13 | 0.32 | 1.77 | 1.58 | 3.05 | 0.00 | 2.05 | up |
| HORVU.MOREX.r2.2HG0172630 | 1890.00 | 1404.00 | 1358.00 | 4915.00 | 4063.00 | 4784.00 | 21.85 | 16.12 | 16.34 | 56.12 | 45.22 | 57.54 | 0.00 | 1.47 | up |
| HORVU.MOREX.r2.1HG0053260 | 3087.00 | 2638.00 | 1173.00 | 20295.00 | 13582.00 | 20945.00 | 199.49 | 169.25 | 78.88 | 1295.09 | 844.93 | 1408.30 | 0.00 | 2.70 | up |
| HORVU.MOREX.r2.3HG0199800 | 506.00 | 645.00 | 461.00 | 1053.00 | 1074.00 | 1343.00 | 5.57 | 7.05 | 5.29 | 11.45 | 11.38 | 15.38 | 0.00 | 1.04 | up |
| HORVU.MOREX.r2.4HG0327950 | 622.00 | 465.00 | 458.00 | 1210.00 | 1685.00 | 2453.00 | 7.14 | 5.30 | 5.47 | 13.73 | 18.65 | 29.33 | 0.00 | 1.63 | up |
| HORVU.MOREX.r2.4HG0320630 | 39.00 | 10.00 | 3.00 | 126.00 | 105.00 | 88.00 | 1.00 | 0.24 | 0.07 | 3.19 | 2.59 | 2.35 | 0.00 | 1.72 | up |
| HORVU.MOREX.r2.2HG0141340 | 491.00 | 281.00 | 257.00 | 988.00 | 978.00 | 1029.00 | 8.91 | 5.07 | 4.85 | 17.72 | 17.11 | 19.44 | 0.00 | 1.40 | up |
| HORVU.MOREX.r2.1HG0040430 | 542.00 | 708.00 | 544.00 | 1579.00 | 1264.00 | 1425.00 | 2.84 | 3.68 | 2.97 | 8.17 | 6.37 | 7.77 | 0.00 | 1.18 | up |
| HORVU.MOREX.r2.4HG0317590 | 522.00 | 440.00 | 183.00 | 2333.00 | 1919.00 | 2302.00 | 6.87 | 5.76 | 2.51 | 30.38 | 24.36 | 31.58 | 0.00 | 2.27 | up |
| HORVU.MOREX.r2.7HG0592010 | 27.00 | 46.00 | 32.00 | 133.00 | 73.00 | 113.00 | 0.19 | 0.32 | 0.23 | 0.94 | 0.50 | 0.84 | 0.00 | 1.36 | up |
| HORVU.MOREX.r2.3HG0266210 | 95.00 | 74.00 | 74.00 | 1347.00 | 1365.00 | 2088.00 | 1.63 | 1.25 | 1.32 | 22.94 | 22.66 | 37.48 | 0.00 | 4.02 | up |
| HORVU.MOREX.r2.7HG0611910 | 1699.00 | 3500.00 | 1848.00 | 11865.00 | 8376.00 | 9188.00 | 9.27 | 18.96 | 10.50 | 63.92 | 43.99 | 52.16 | 0.00 | 1.91 | up |
| HORVU.MOREX.r2.1HG0046920 | 45.00 | 152.00 | 74.00 | 199.00 | 426.00 | 499.00 | 0.58 | 1.94 | 0.99 | 2.53 | 5.30 | 6.70 | 0.00 | 1.69 | up |
| HORVU.MOREX.r2.2HG0157530 | 315.00 | 344.00 | 259.00 | 917.00 | 712.00 | 982.00 | 6.22 | 6.74 | 5.32 | 17.90 | 13.54 | 20.21 | 0.00 | 1.42 | up |
| HORVU.MOREX.r2.4HG0321610 | 810.00 | 1047.00 | 929.00 | 1791.00 | 2109.00 | 2479.00 | 8.87 | 11.38 | 10.58 | 19.36 | 22.23 | 28.24 | 0.00 | 1.12 | up |
| HORVU.MOREX.r2.6HG0475820 | 117.00 | 189.00 | 94.00 | 668.00 | 431.00 | 538.00 | 0.40 | 0.65 | 0.34 | 2.27 | 1.43 | 1.93 | 0.00 | 1.85 | up |
| Hordeum_vulgare_newGene_5856 | 53.00 | 81.00 | 107.00 | 202.00 | 174.00 | 285.00 | 0.42 | 0.68 | 0.77 | 1.50 | 1.27 | 2.00 | 0.00 | 1.26 | up |
| HORVU.MOREX.r2.5HG0409600 | 49.00 | 47.00 | 15.00 | 189.00 | 208.00 | 210.00 | 0.49 | 0.46 | 0.15 | 1.87 | 2.00 | 2.19 | 0.00 | 2.10 | up |
| HORVU.MOREX.r2.2HG0101040 | 31.00 | 52.00 | 24.00 | 113.00 | 70.00 | 329.00 | 2.58 | 4.27 | 2.03 | 9.37 | 5.65 | 28.77 | 0.00 | 1.68 | up |
| HORVU.MOREX.r2.6HG0454550 | 63.00 | 72.00 | 49.00 | 134.00 | 151.00 | 171.00 | 2.32 | 2.66 | 1.88 | 4.93 | 5.40 | 6.62 | 0.00 | 1.18 | up |
| HORVU.MOREX.r2.1HG0001400 | 11.00 | 11.00 | 23.00 | 266.00 | 201.00 | 299.00 | 0.10 | 0.10 | 0.21 | 2.38 | 1.75 | 2.83 | 0.00 | 3.52 | up |
| HORVU.MOREX.r2.5HG0367910 | 179.00 | 375.00 | 177.00 | 2255.00 | 1710.00 | 1953.00 | 6.20 | 12.95 | 6.41 | 77.55 | 57.31 | 70.75 | 0.00 | 2.79 | up |
| HORVU.MOREX.r2.7HG0584050 | 80.00 | 78.00 | 41.00 | 326.00 | 205.00 | 220.00 | 1.52 | 1.47 | 0.80 | 6.17 | 3.78 | 4.38 | 0.00 | 1.70 | up |
| HORVU.MOREX.r2.5HG0413760 | 62.00 | 72.00 | 71.00 | 144.00 | 153.00 | 160.00 | 0.39 | 0.45 | 0.46 | 0.89 | 0.92 | 1.04 | 0.00 | 1.05 | up |
| HORVU.MOREX.r2.1HG0030370 | 777.00 | 647.00 | 626.00 | 1211.00 | 1784.00 | 2488.00 | 8.02 | 6.63 | 6.72 | 12.35 | 17.74 | 26.74 | 0.00 | 1.29 | up |
| HORVU.MOREX.r2.7HG0590510 | 18.00 | 55.00 | 57.00 | 87.00 | 147.00 | 152.00 | 0.20 | 0.62 | 0.67 | 0.97 | 1.61 | 1.80 | 0.01 | 1.26 | up |
| HORVU.MOREX.r2.3HG0256140 | 6.00 | 0.00 | 9.00 | 88.00 | 56.00 | 59.00 | 0.30 | 0.00 | 0.42 | 4.32 | 2.66 | 3.07 | 0.00 | 2.44 | up |
| HORVU.MOREX.r2.3HG0199670 | 14.00 | 11.00 | 9.00 | 294.00 | 186.00 | 291.00 | 0.16 | 0.12 | 0.10 | 3.40 | 2.09 | 3.54 | 0.00 | 3.89 | up |
| HORVU.MOREX.r2.6HG0526180 | 147.00 | 362.00 | 294.00 | 751.00 | 508.00 | 797.00 | 2.07 | 5.07 | 4.31 | 10.47 | 6.90 | 11.70 | 0.00 | 1.20 | up |
| HORVU.MOREX.r2.4HG0339550 | 164.00 | 162.00 | 52.00 | 4281.00 | 2415.00 | 3922.00 | 1.80 | 1.77 | 0.59 | 46.65 | 25.66 | 45.03 | 0.00 | 3.71 | up |
| HORVU.MOREX.r2.5HG0367560 | 221.00 | 143.00 | 104.00 | 541.00 | 262.00 | 359.00 | 1.98 | 1.27 | 0.97 | 4.78 | 2.26 | 3.35 | 0.00 | 1.13 | up |
| HORVU.MOREX.r2.3HG0262920 | 43.00 | 25.00 | 58.00 | 156.00 | 115.00 | 171.00 | 0.59 | 0.35 | 0.84 | 2.17 | 1.55 | 2.50 | 0.00 | 1.52 | up |
| HORVU.MOREX.r2.5HG0349880 | 693.00 | 501.00 | 194.00 | 10182.00 | 6509.00 | 8844.00 | 5.63 | 4.04 | 1.64 | 81.68 | 50.89 | 74.74 | 0.00 | 3.35 | up |
| HORVU.MOREX.r2.4HG0294660 | 548.00 | 650.00 | 500.00 | 1145.00 | 1027.00 | 1471.00 | 5.86 | 6.91 | 5.57 | 12.11 | 10.58 | 16.39 | 0.00 | 1.03 | up |
| HORVU.MOREX.r2.UnG0627300 | 35.00 | 72.00 | 71.00 | 118.00 | 152.00 | 133.00 | 0.22 | 0.44 | 0.46 | 0.72 | 0.91 | 0.86 | 0.01 | 1.01 | up |
| HORVU.MOREX.r2.2HG0164860 | 32.00 | 34.00 | 49.00 | 157.00 | 90.00 | 68.00 | 0.33 | 0.35 | 0.54 | 1.65 | 0.92 | 0.75 | 0.01 | 1.18 | up |
| HORVU.MOREX.r2.1HG0062100 | 375.00 | 396.00 | 279.00 | 960.00 | 730.00 | 676.00 | 2.28 | 2.40 | 1.77 | 5.78 | 4.28 | 4.29 | 0.00 | 1.09 | up |
| HORVU.MOREX.r2.5HG0414080 | 222.00 | 452.00 | 421.00 | 827.00 | 820.00 | 914.00 | 3.29 | 6.67 | 6.51 | 12.14 | 11.73 | 14.13 | 0.00 | 1.11 | up |
| HORVU.MOREX.r2.3HG0272550 | 18708.00 | 18607.00 | 10503.00 | 108611.00 | 101405.00 | 134652.00 | 740.16 | 730.94 | 432.62 | 4243.51 | 3862.51 | 5543.13 | 0.00 | 2.68 | up |
| HORVU.MOREX.r2.6HG0469440 | 779.00 | 549.00 | 450.00 | 2010.00 | 1536.00 | 1877.00 | 4.88 | 3.41 | 2.93 | 12.43 | 9.26 | 12.24 | 0.00 | 1.49 | up |
| HORVU.MOREX.r2.7HG0527920 | 218.00 | 198.00 | 130.00 | 557.00 | 342.00 | 525.00 | 1.92 | 1.73 | 1.18 | 4.83 | 2.89 | 4.80 | 0.00 | 1.25 | up |
| HORVU.MOREX.r2.7HG0549680 | 152.00 | 100.00 | 90.00 | 301.00 | 287.00 | 336.00 | 2.11 | 1.37 | 1.30 | 4.14 | 3.83 | 4.86 | 0.00 | 1.30 | up |
| HORVU.MOREX.r2.3HG0186320 | 3772.00 | 3030.00 | 2095.00 | 9998.00 | 8463.00 | 10786.00 | 56.24 | 44.85 | 32.52 | 147.23 | 121.49 | 167.36 | 0.00 | 1.61 | up |
| HORVU.MOREX.r2.2HG0101500 | 0.00 | 3.00 | 0.00 | 203.00 | 174.00 | 261.00 | 0.00 | 0.10 | 0.00 | 10.47 | 8.73 | 14.21 | 0.00 | 5.31 | up |
| HORVU.MOREX.r2.6HG0465340 | 121.00 | 112.00 | 253.00 | 455.00 | 385.00 | 595.00 | 1.32 | 1.21 | 2.87 | 4.91 | 4.05 | 6.77 | 0.00 | 1.34 | up |
| HORVU.MOREX.r2.6HG0509890 | 1545.00 | 1367.00 | 1047.00 | 2781.00 | 2526.00 | 3035.00 | 21.09 | 18.53 | 14.88 | 37.49 | 33.20 | 43.10 | 0.00 | 1.00 | up |
| HORVU.MOREX.r2.4HG0338490 | 243.00 | 221.00 | 95.00 | 1853.00 | 865.00 | 1434.00 | 11.37 | 10.27 | 4.62 | 85.79 | 39.03 | 69.95 | 0.00 | 2.54 | up |
| HORVU.MOREX.r2.7HG0588540 | 30.00 | 7.00 | 9.00 | 364.00 | 173.00 | 281.00 | 0.88 | 0.19 | 0.26 | 10.76 | 4.97 | 8.76 | 0.00 | 3.23 | up |
| HORVU.MOREX.r2.6HG0519770 | 136.00 | 400.00 | 387.00 | 782.00 | 777.00 | 668.00 | 1.13 | 3.30 | 3.35 | 6.42 | 6.22 | 5.78 | 0.00 | 1.10 | up |
| HORVU.MOREX.r2.3HG0245240 | 132.00 | 86.00 | 82.00 | 379.00 | 705.00 | 900.00 | 1.94 | 1.25 | 1.25 | 5.51 | 9.99 | 13.77 | 0.00 | 2.40 | up |
| HORVU.MOREX.r2.2HG0165160 | 426.00 | 365.00 | 319.00 | 1009.00 | 1093.00 | 993.00 | 4.23 | 3.60 | 3.30 | 9.91 | 10.46 | 10.26 | 0.00 | 1.40 | up |
| HORVU.MOREX.r2.1HG0043210 | 7587.00 | 9718.00 | 6520.00 | 230615.00 | 168077.00 | 217913.00 | 171.70 | 218.35 | 153.60 | 5153.79 | 3661.89 | 5131.10 | 0.00 | 4.51 | up |
| HORVU.MOREX.r2.6HG0458460 | 2230.00 | 2960.00 | 1608.00 | 6883.00 | 4608.00 | 6618.00 | 144.09 | 189.91 | 108.15 | 439.24 | 286.68 | 444.94 | 0.00 | 1.31 | up |
| HORVU.MOREX.r2.6HG0521770 | 633.00 | 488.00 | 447.00 | 3530.00 | 2391.00 | 4138.00 | 13.10 | 10.02 | 9.62 | 72.13 | 47.63 | 89.09 | 0.00 | 2.50 | up |
| Hordeum_vulgare_newGene_2766 | 51.00 | 40.00 | 60.00 | 281.00 | 171.00 | 342.00 | 0.39 | 0.31 | 0.48 | 2.17 | 1.29 | 2.78 | 0.00 | 2.09 | up |
| HORVU.MOREX.r2.7HG0561660 | 3195.00 | 2635.00 | 2898.00 | 5912.00 | 7152.00 | 6114.00 | 49.15 | 40.24 | 46.42 | 89.83 | 105.93 | 97.88 | 0.00 | 1.06 | up |
| HORVU.MOREX.r2.6HG0464980 | 15.00 | 58.00 | 51.00 | 1402.00 | 671.00 | 1043.00 | 0.36 | 1.46 | 1.35 | 35.30 | 16.46 | 27.68 | 0.00 | 3.95 | up |
| HORVU.MOREX.r2.5HG0367850 | 87.00 | 247.00 | 108.00 | 575.00 | 422.00 | 529.00 | 3.01 | 8.51 | 3.89 | 19.77 | 14.13 | 19.16 | 0.00 | 1.57 | up |
| HORVU.MOREX.r2.3HG0235640 | 96.00 | 47.00 | 62.00 | 746.00 | 454.00 | 681.00 | 0.48 | 0.23 | 0.32 | 3.69 | 2.19 | 3.55 | 0.00 | 2.86 | up |
| HORVU.MOREX.r2.5HG0416690 | 4503.00 | 4862.00 | 3995.00 | 13479.00 | 14063.00 | 16170.00 | 53.22 | 57.06 | 49.16 | 157.35 | 160.04 | 198.87 | 0.00 | 1.64 | up |
| HORVU.MOREX.r2.5HG0385400 | 33.00 | 54.00 | 37.00 | 170.00 | 113.00 | 123.00 | 0.17 | 0.28 | 0.20 | 0.87 | 0.57 | 0.66 | 0.00 | 1.50 | up |
| HORVU.MOREX.r2.5HG0393830 | 67.00 | 71.00 | 69.00 | 135.00 | 163.00 | 167.00 | 0.81 | 0.86 | 0.88 | 1.64 | 1.92 | 2.13 | 0.00 | 1.06 | up |
| HORVU.MOREX.r2.2HG0082080 | 430.00 | 321.00 | 146.00 | 2021.00 | 2141.00 | 2188.00 | 5.10 | 3.78 | 1.81 | 23.74 | 24.51 | 27.06 | 0.00 | 2.55 | up |
| HORVU.MOREX.r2.1HG0076450 | 350.00 | 421.00 | 427.00 | 1933.00 | 1557.00 | 1499.00 | 4.14 | 4.96 | 5.27 | 22.65 | 17.79 | 18.51 | 0.00 | 1.96 | up |
| HORVU.MOREX.r2.7HG0592280 | 50.00 | 59.00 | 44.00 | 1400.00 | 1044.00 | 1595.00 | 0.55 | 0.65 | 0.50 | 15.37 | 11.18 | 18.45 | 0.00 | 4.43 | up |
| HORVU.MOREX.r2.7HG0596500 | 135.00 | 241.00 | 254.00 | 1328.00 | 1603.00 | 2444.00 | 2.64 | 4.69 | 5.19 | 25.77 | 30.32 | 49.96 | 0.00 | 2.81 | up |
| HORVU.MOREX.r2.3HG0255940 | 1764.00 | 907.00 | 582.00 | 2759.00 | 2932.00 | 3224.00 | 39.00 | 19.90 | 13.39 | 60.25 | 62.41 | 74.16 | 0.00 | 1.28 | up |
| HORVU.MOREX.r2.4HG0335550 | 499.00 | 355.00 | 148.00 | 2089.00 | 1288.00 | 1588.00 | 10.28 | 7.27 | 3.16 | 42.53 | 25.57 | 34.06 | 0.00 | 2.01 | up |
| HORVU.MOREX.r2.1HG0050160 | 20.00 | 21.00 | 18.00 | 114.00 | 100.00 | 115.00 | 0.13 | 0.13 | 0.12 | 0.73 | 0.62 | 0.78 | 0.00 | 2.18 | up |
| HORVU.MOREX.r2.1HG0052830 | 4.00 | 24.00 | 15.00 | 118.00 | 138.00 | 58.00 | 0.03 | 0.16 | 0.11 | 0.80 | 0.91 | 0.41 | 0.00 | 2.11 | up |
| HORVU.MOREX.r2.6HG0455190 | 24.00 | 12.00 | 45.00 | 88.00 | 117.00 | 244.00 | 0.47 | 0.23 | 0.95 | 1.75 | 2.28 | 5.12 | 0.00 | 1.83 | up |
| HORVU.MOREX.r2.UnG0630000 | 57.00 | 97.00 | 83.00 | 1229.00 | 1065.00 | 844.00 | 0.79 | 1.33 | 1.19 | 16.88 | 14.26 | 12.20 | 0.00 | 3.50 | up |
| HORVU.MOREX.r2.7HG0534370 | 27.00 | 43.00 | 23.00 | 9642.00 | 4781.00 | 7315.00 | 0.30 | 0.48 | 0.27 | 108.37 | 52.38 | 86.62 | 0.00 | 7.18 | up |
| HORVU.MOREX.r2.2HG0154770 | 3337.00 | 3017.00 | 2566.00 | 7982.00 | 8695.00 | 12150.00 | 27.56 | 24.74 | 22.06 | 65.11 | 69.15 | 104.44 | 0.00 | 1.59 | up |
| HORVU.MOREX.r2.6HG0457770 | 173.00 | 239.00 | 219.00 | 1308.00 | 1286.00 | 1495.00 | 1.29 | 1.77 | 1.70 | 9.64 | 9.24 | 11.61 | 0.00 | 2.58 | up |
| HORVU.MOREX.r2.7HG0551800 | 1890.00 | 1778.00 | 1618.00 | 3493.00 | 3297.00 | 4848.00 | 34.13 | 31.88 | 30.42 | 62.30 | 57.33 | 91.09 | 0.00 | 1.06 | up |
| Hordeum_vulgare_newGene_6773 | 457.00 | 877.00 | 550.00 | 5539.00 | 3335.00 | 4667.00 | 3.47 | 6.62 | 4.35 | 41.60 | 24.42 | 36.93 | 0.00 | 2.64 | up |
| Hordeum_vulgare_newGene_6775 | 229.00 | 211.00 | 158.00 | 394.00 | 438.00 | 514.00 | 7.99 | 7.29 | 5.74 | 13.58 | 14.74 | 18.67 | 0.00 | 1.08 | up |
| HORVU.MOREX.r2.7HG0536060 | 79.00 | 57.00 | 54.00 | 365.00 | 216.00 | 290.00 | 5.06 | 3.60 | 3.60 | 23.25 | 13.44 | 19.45 | 0.00 | 1.97 | up |
| HORVU.MOREX.r2.6HG0463750 | 267.00 | 348.00 | 232.00 | 788.00 | 644.00 | 827.00 | 1.21 | 1.56 | 1.09 | 3.52 | 2.80 | 3.89 | 0.00 | 1.33 | up |
| HORVU.MOREX.r2.1HG0007880 | 2.00 | 10.00 | 0.00 | 134.00 | 108.00 | 106.00 | 0.04 | 0.19 | 0.00 | 2.61 | 2.05 | 2.17 | 0.00 | 3.45 | up |
| HORVU.MOREX.r2.5HG0407350 | 8.00 | 0.00 | 3.00 | 324.00 | 349.00 | 307.00 | 0.22 | 0.00 | 0.09 | 10.04 | 10.56 | 10.02 | 0.00 | 5.35 | up |
| HORVU.MOREX.r2.3HG0230650 | 48.00 | 85.00 | 78.00 | 183.00 | 153.00 | 134.00 | 0.37 | 0.66 | 0.64 | 1.42 | 1.16 | 1.10 | 0.00 | 1.02 | up |
| HORVU.MOREX.r2.2HG0129090 | 38.00 | 71.00 | 32.00 | 202.00 | 204.00 | 318.00 | 0.45 | 0.83 | 0.39 | 2.35 | 2.32 | 3.90 | 0.00 | 2.06 | up |
| HORVU.MOREX.r2.5HG0389190 | 151.00 | 145.00 | 64.00 | 33080.00 | 20023.00 | 27134.00 | 2.32 | 2.20 | 1.02 | 502.63 | 296.59 | 434.38 | 0.00 | 7.18 | up |
| HORVU.MOREX.r2.2HG0165750 | 437.00 | 585.00 | 474.00 | 4038.00 | 1706.00 | 3552.00 | 4.48 | 5.95 | 5.05 | 40.90 | 16.84 | 37.90 | 0.00 | 2.40 | up |
| HORVU.MOREX.r2.6HG0508560 | 64.00 | 67.00 | 92.00 | 159.00 | 177.00 | 167.00 | 0.56 | 0.58 | 0.84 | 1.38 | 1.50 | 1.53 | 0.00 | 1.05 | up |
| HORVU.MOREX.r2.2HG0114300 | 1501.00 | 1957.00 | 1491.00 | 4702.00 | 4141.00 | 5020.00 | 12.69 | 16.42 | 13.11 | 39.25 | 33.69 | 44.15 | 0.00 | 1.42 | up |
| HORVU.MOREX.r2.7HG0560340 | 1019.00 | 720.00 | 459.00 | 6231.00 | 2861.00 | 3662.00 | 84.65 | 59.39 | 39.62 | 511.19 | 228.80 | 316.51 | 0.00 | 2.26 | up |
| HORVU.MOREX.r2.7HG0605040 | 8.00 | 24.00 | 17.00 | 70.00 | 70.00 | 59.00 | 0.51 | 1.57 | 1.16 | 4.58 | 4.45 | 4.05 | 0.00 | 1.62 | up |
| HORVU.MOREX.r2.6HG0505650 | 847.00 | 346.00 | 314.00 | 6306.00 | 4676.00 | 6985.00 | 73.47 | 29.81 | 28.35 | 540.49 | 390.71 | 630.81 | 0.00 | 2.74 | up |
| HORVU.MOREX.r2.6HG0449830 | 57.00 | 57.00 | 44.00 | 324.00 | 134.00 | 199.00 | 0.99 | 0.98 | 0.79 | 5.58 | 2.24 | 3.61 | 0.00 | 1.77 | up |
| HORVU.MOREX.r2.4HG0314970 | 0.00 | 0.00 | 0.00 | 133.00 | 161.00 | 55.00 | 0.00 | 0.00 | 0.00 | 2.55 | 3.00 | 1.10 | 0.00 | 4.53 | up |
| HORVU.MOREX.r2.7HG0567670 | 341.00 | 423.00 | 345.00 | 976.00 | 840.00 | 873.00 | 3.38 | 4.17 | 3.56 | 9.58 | 8.04 | 9.03 | 0.00 | 1.21 | up |
| HORVU.MOREX.r2.2HG0081840 | 4.00 | 29.00 | 21.00 | 215.00 | 89.00 | 120.00 | 0.04 | 0.33 | 0.25 | 2.46 | 0.99 | 1.45 | 0.00 | 2.17 | up |
| HORVU.MOREX.r2.7HG0591640 | 5153.00 | 4546.00 | 3724.00 | 12199.00 | 8542.00 | 12104.00 | 89.73 | 78.59 | 67.50 | 209.76 | 143.20 | 219.29 | 0.00 | 1.21 | up |
| HORVU.MOREX.r2.5HG0357930 | 495.00 | 823.00 | 564.00 | 1965.00 | 1687.00 | 2446.00 | 4.26 | 7.03 | 5.06 | 16.71 | 13.99 | 21.92 | 0.00 | 1.59 | up |
| HORVU.MOREX.r2.4HG0321620 | 28.00 | 27.00 | 11.00 | 112.00 | 82.00 | 116.00 | 0.23 | 0.21 | 0.09 | 0.89 | 0.63 | 0.97 | 0.00 | 1.86 | up |
| HORVU.MOREX.r2.4HG0286630 | 32.00 | 29.00 | 48.00 | 110.00 | 105.00 | 164.00 | 0.35 | 0.31 | 0.55 | 1.20 | 1.11 | 1.88 | 0.00 | 1.53 | up |
| HORVU.MOREX.r2.6HG0521180 | 227.00 | 455.00 | 295.00 | 884.00 | 559.00 | 667.00 | 2.01 | 4.00 | 2.72 | 7.74 | 4.77 | 6.14 | 0.00 | 1.00 | up |
| HORVU.MOREX.r2.1HG0050900 | 205.00 | 363.00 | 237.00 | 940.00 | 695.00 | 738.00 | 1.51 | 2.66 | 1.82 | 6.86 | 4.95 | 5.68 | 0.00 | 1.44 | up |
| HORVU.MOREX.r2.6HG0511040 | 85.00 | 143.00 | 140.00 | 328.00 | 254.00 | 274.00 | 0.50 | 0.84 | 0.85 | 1.91 | 1.44 | 1.68 | 0.00 | 1.10 | up |
| HORVU.MOREX.r2.6HG0482280 | 325.00 | 205.00 | 188.00 | 625.00 | 508.00 | 542.00 | 5.40 | 3.39 | 3.25 | 10.28 | 8.14 | 9.39 | 0.00 | 1.11 | up |
| HORVU.MOREX.r2.3HG0231980 | 4161.00 | 5386.00 | 4516.00 | 9693.00 | 8834.00 | 11583.00 | 26.74 | 34.37 | 30.21 | 61.51 | 54.65 | 77.45 | 0.00 | 1.03 | up |
| HORVU.MOREX.r2.7HG0549590 | 5.00 | 11.00 | 14.00 | 622.00 | 566.00 | 463.00 | 0.16 | 0.39 | 0.49 | 22.18 | 19.68 | 17.37 | 0.00 | 5.18 | up |
| HORVU.MOREX.r2.1HG0070350 | 154.00 | 129.00 | 116.00 | 480.00 | 233.00 | 346.00 | 1.56 | 1.29 | 1.23 | 4.81 | 2.27 | 3.65 | 0.00 | 1.25 | up |
| HORVU.MOREX.r2.3HG0229630 | 1526.00 | 2581.00 | 2004.00 | 21616.00 | 13027.00 | 15963.00 | 29.58 | 49.68 | 40.43 | 413.82 | 243.13 | 321.99 | 0.00 | 2.86 | up |
| HORVU.MOREX.r2.7HG0620940 | 393.00 | 534.00 | 199.00 | 3449.00 | 2243.00 | 2871.00 | 10.05 | 13.58 | 5.29 | 87.25 | 55.31 | 76.51 | 0.00 | 2.66 | up |
| HORVU.MOREX.r2.7HG0602420 | 1345.00 | 959.00 | 722.00 | 1739.00 | 2206.00 | 2574.00 | 20.00 | 14.15 | 11.18 | 25.53 | 31.59 | 39.83 | 0.00 | 1.01 | up |
| HORVU.MOREX.r2.5HG0430680 | 482.00 | 849.00 | 524.00 | 1265.00 | 1307.00 | 1430.00 | 4.70 | 8.22 | 5.32 | 12.19 | 12.27 | 14.51 | 0.00 | 1.03 | up |
| HORVU.MOREX.r2.4HG0278540 | 223.00 | 95.00 | 26.00 | 1496.00 | 1025.00 | 1370.00 | 8.70 | 3.65 | 1.04 | 57.64 | 38.48 | 55.61 | 0.00 | 2.08 | up |
| HORVU.MOREX.r2.4HG0279630 | 93.00 | 241.00 | 68.00 | 10421.00 | 7620.00 | 14403.00 | 2.35 | 6.05 | 1.79 | 261.35 | 186.31 | 380.59 | 0.00 | 4.46 | up |
| HORVU.MOREX.r2.3HG0234320 | 615.00 | 536.00 | 355.00 | 24076.00 | 13473.00 | 16506.00 | 2.83 | 2.45 | 1.70 | 109.57 | 59.77 | 79.14 | 0.00 | 4.82 | up |
| HORVU.MOREX.r2.3HG0242500 | 0.00 | 7.00 | 7.00 | 117.00 | 79.00 | 91.00 | 0.00 | 0.14 | 0.15 | 2.43 | 1.61 | 1.99 | 0.00 | 3.17 | up |
| HORVU.MOREX.r2.1HG0004080 | 393.00 | 706.00 | 521.00 | 3457.00 | 2224.00 | 2992.00 | 4.27 | 7.62 | 5.89 | 37.11 | 23.27 | 33.83 | 0.00 | 2.26 | up |
| HORVU.MOREX.r2.2HG0158630 | 3551.00 | 4015.00 | 2846.00 | 10198.00 | 7461.00 | 10665.00 | 32.06 | 35.99 | 26.76 | 90.95 | 64.87 | 100.21 | 0.00 | 1.36 | up |
| HORVU.MOREX.r2.1HG0040490 | 1914.00 | 1772.00 | 1138.00 | 6625.00 | 5808.00 | 6623.00 | 47.16 | 43.35 | 29.19 | 161.22 | 137.78 | 169.81 | 0.00 | 1.87 | up |
| HORVU.MOREX.r2.4HG0277890 | 4990.00 | 3259.00 | 1462.00 | 59320.00 | 52661.00 | 64285.00 | 113.35 | 73.50 | 34.57 | 1330.85 | 1151.81 | 1519.59 | 0.00 | 3.40 | up |
| HORVU.MOREX.r2.3HG0260850 | 123.00 | 74.00 | 69.00 | 239.00 | 205.00 | 237.00 | 4.93 | 2.92 | 2.85 | 9.47 | 7.88 | 9.89 | 0.00 | 1.21 | up |
| HORVU.MOREX.r2.2HG0155370 | 24.00 | 13.00 | 0.00 | 6460.00 | 5040.00 | 10839.00 | 0.64 | 0.32 | 0.00 | 169.41 | 128.86 | 299.49 | 0.00 | 1.84 | up |
| HORVU.MOREX.r2.2HG0125570 | 93.00 | 129.00 | 55.00 | 260.00 | 214.00 | 304.00 | 1.98 | 2.75 | 1.21 | 5.50 | 4.41 | 6.78 | 0.00 | 1.32 | up |
| HORVU.MOREX.r2.7HG0555000 | 555.00 | 265.00 | 394.00 | 1974.00 | 1599.00 | 1987.00 | 18.44 | 8.72 | 13.60 | 64.77 | 51.16 | 68.70 | 0.00 | 2.00 | up |
| HORVU.MOREX.r2.2HG0083460 | 461.00 | 507.00 | 378.00 | 1171.00 | 909.00 | 814.00 | 2.70 | 2.95 | 2.31 | 6.79 | 5.14 | 4.98 | 0.00 | 1.03 | up |
| HORVU.MOREX.r2.1HG0047900 | 0.00 | 3.00 | 2.00 | 986.00 | 610.00 | 761.00 | 0.00 | 0.02 | 0.02 | 10.33 | 6.23 | 8.40 | 0.00 | 6.86 | up |
| HORVU.MOREX.r2.5HG0438970 | 34.00 | 85.00 | 70.00 | 110.00 | 205.00 | 167.00 | 0.93 | 2.30 | 1.99 | 2.97 | 5.41 | 4.76 | 0.01 | 1.13 | up |
| HORVU.MOREX.r2.2HG0160580 | 160.00 | 204.00 | 94.00 | 7307.00 | 3469.00 | 4545.00 | 2.86 | 3.62 | 1.74 | 129.12 | 59.75 | 84.62 | 0.00 | 4.60 | up |
| HORVU.MOREX.r2.7HG0616070 | 175.00 | 159.00 | 173.00 | 402.00 | 332.00 | 398.00 | 1.13 | 1.02 | 1.16 | 2.56 | 2.06 | 2.68 | 0.00 | 1.07 | up |
| HORVU.MOREX.r2.2HG0099600 | 650.00 | 340.00 | 352.00 | 1475.00 | 858.00 | 1003.00 | 5.77 | 3.00 | 3.25 | 12.93 | 7.33 | 9.26 | 0.00 | 1.16 | up |
| HORVU.MOREX.r2.2HG0161180 | 58.00 | 58.00 | 90.00 | 140.00 | 161.00 | 156.00 | 0.20 | 0.20 | 0.32 | 0.48 | 0.54 | 0.56 | 0.00 | 1.01 | up |
| HORVU.MOREX.r2.2HG0112510 | 795.00 | 1149.00 | 810.00 | 2458.00 | 1918.00 | 2138.00 | 5.62 | 8.06 | 5.95 | 17.15 | 13.05 | 15.72 | 0.00 | 1.17 | up |
| HORVU.MOREX.r2.7HG0554930 | 566.00 | 664.00 | 640.00 | 1692.00 | 1312.00 | 1766.00 | 6.16 | 7.17 | 7.25 | 18.20 | 13.75 | 20.01 | 0.00 | 1.27 | up |
| HORVU.MOREX.r2.3HG0191370 | 475.00 | 713.00 | 522.00 | 1250.00 | 1277.00 | 1629.00 | 3.33 | 4.96 | 3.81 | 8.66 | 8.62 | 11.89 | 0.00 | 1.20 | up |
| HORVU.MOREX.r2.1HG0051730 | 690.00 | 308.00 | 241.00 | 1701.00 | 881.00 | 1042.00 | 7.77 | 3.44 | 2.82 | 18.92 | 9.55 | 12.22 | 0.00 | 1.32 | up |
| HORVU.MOREX.r2.3HG0193840 | 49.00 | 31.00 | 81.00 | 387.00 | 204.00 | 299.00 | 0.60 | 0.37 | 1.05 | 4.75 | 2.44 | 3.87 | 0.00 | 2.07 | up |
| HORVU.MOREX.r2.3HG0192130 | 30.00 | 18.00 | 32.00 | 124.00 | 84.00 | 69.00 | 1.17 | 0.70 | 1.34 | 4.94 | 3.25 | 2.89 | 0.00 | 1.47 | up |
| HORVU.MOREX.r2.UnG0632630 | 42.00 | 15.00 | 4.00 | 194.00 | 117.00 | 216.00 | 0.68 | 0.23 | 0.07 | 3.11 | 1.82 | 3.65 | 0.00 | 2.20 | up |
| HORVU.MOREX.r2.2HG0080580 | 235.00 | 254.00 | 362.00 | 1171.00 | 487.00 | 982.00 | 1.86 | 2.00 | 2.99 | 9.18 | 3.72 | 8.11 | 0.00 | 1.43 | up |
| Hordeum_vulgare_newGene_4476 | 50.00 | 59.00 | 35.00 | 136.00 | 107.00 | 112.00 | 1.75 | 2.07 | 1.29 | 4.77 | 3.65 | 4.15 | 0.00 | 1.15 | up |
| HORVU.MOREX.r2.7HG0598720 | 39.00 | 50.00 | 40.00 | 11651.00 | 5981.00 | 9047.00 | 0.44 | 0.56 | 0.46 | 130.95 | 65.53 | 107.13 | 0.00 | 7.13 | up |
| HORVU.MOREX.r2.2HG0178050 | 12.00 | 110.00 | 32.00 | 378.00 | 196.00 | 281.00 | 0.31 | 2.99 | 0.91 | 10.24 | 5.17 | 8.02 | 0.00 | 1.81 | up |
| HORVU.MOREX.r2.2HG0145840 | 520.00 | 541.00 | 574.00 | 2096.00 | 1560.00 | 1700.00 | 5.88 | 6.08 | 6.76 | 23.42 | 16.98 | 20.01 | 0.00 | 1.62 | up |
| HORVU.MOREX.r2.6HG0476120 | 36.00 | 43.00 | 23.00 | 819.00 | 726.00 | 1074.00 | 1.06 | 1.25 | 0.70 | 23.88 | 20.62 | 32.97 | 0.00 | 4.32 | up |
| HORVU.MOREX.r2.1HG0046180 | 522.00 | 677.00 | 471.00 | 1269.00 | 1059.00 | 1204.00 | 8.54 | 11.00 | 8.02 | 20.53 | 16.70 | 20.52 | 0.00 | 1.01 | up |
| HORVU.MOREX.r2.1HG0074990 | 276.00 | 162.00 | 126.00 | 1454.00 | 744.00 | 995.00 | 9.72 | 5.66 | 4.60 | 50.59 | 25.22 | 36.48 | 0.00 | 2.22 | up |
| HORVU.MOREX.r2.7HG0563920 | 44.00 | 21.00 | 27.00 | 73.00 | 105.00 | 196.00 | 0.59 | 0.27 | 0.37 | 0.97 | 1.36 | 2.75 | 0.00 | 1.60 | up |
| HORVU.MOREX.r2.2HG0155850 | 14.00 | 30.00 | 25.00 | 66.00 | 70.00 | 109.00 | 0.57 | 1.26 | 1.07 | 2.77 | 2.83 | 4.80 | 0.00 | 1.50 | up |
| HORVU.MOREX.r2.3HG0232400 | 3730.00 | 2609.00 | 1789.00 | 8011.00 | 6980.00 | 8280.00 | 41.87 | 29.08 | 20.91 | 88.82 | 75.44 | 96.73 | 0.00 | 1.40 | up |
| HORVU.MOREX.r2.1HG0035630 | 14.00 | 31.00 | 45.00 | 82.00 | 118.00 | 108.00 | 0.24 | 0.53 | 0.80 | 1.40 | 1.97 | 1.95 | 0.00 | 1.43 | up |
| HORVU.MOREX.r2.1HG0077330 | 3673.00 | 3381.00 | 2996.00 | 17306.00 | 15336.00 | 21608.00 | 90.49 | 82.73 | 76.87 | 421.15 | 363.84 | 554.06 | 0.00 | 2.32 | up |
| HORVU.MOREX.r2.3HG0183910 | 31.00 | 47.00 | 40.00 | 93.00 | 93.00 | 111.00 | 0.65 | 0.98 | 0.88 | 1.94 | 1.90 | 2.45 | 0.00 | 1.17 | up |
| HORVU.MOREX.r2.7HG0542410 | 10.00 | 3.00 | 0.00 | 85.00 | 63.00 | 110.00 | 0.44 | 0.10 | 0.00 | 3.77 | 2.69 | 5.13 | 0.00 | 2.81 | up |
| Hordeum_vulgare_newGene_5118 | 10.00 | 16.00 | 11.00 | 60.00 | 61.00 | 99.00 | 0.31 | 0.51 | 0.37 | 2.02 | 1.98 | 3.51 | 0.00 | 2.07 | up |
| HORVU.MOREX.r2.2HG0139100 | 328.00 | 431.00 | 426.00 | 1156.00 | 1126.00 | 1162.00 | 2.47 | 3.22 | 3.34 | 8.59 | 8.17 | 9.10 | 0.00 | 1.46 | up |
| HORVU.MOREX.r2.7HG0597810 | 168.00 | 100.00 | 107.00 | 317.00 | 297.00 | 241.00 | 4.02 | 2.37 | 2.66 | 7.50 | 6.86 | 6.01 | 0.00 | 1.06 | up |
| HORVU.MOREX.r2.1HG0007120 | 137.00 | 148.00 | 76.00 | 649.00 | 332.00 | 337.00 | 1.19 | 1.27 | 0.68 | 5.57 | 2.78 | 3.05 | 0.00 | 1.64 | up |
| HORVU.MOREX.r2.5HG0394350 | 124.00 | 129.00 | 167.00 | 1468.00 | 659.00 | 1077.00 | 1.22 | 1.27 | 1.72 | 14.38 | 6.29 | 11.12 | 0.00 | 2.64 | up |
| HORVU.MOREX.r2.2HG0140860 | 4677.00 | 3707.00 | 2882.00 | 15615.00 | 12410.00 | 14579.00 | 74.11 | 58.32 | 47.54 | 244.36 | 189.33 | 240.39 | 0.00 | 1.81 | up |
| HORVU.MOREX.r2.6HG0469530 | 0.00 | 0.00 | 0.00 | 272.00 | 104.00 | 171.00 | 0.00 | 0.00 | 0.00 | 6.76 | 2.50 | 4.47 | 0.00 | 5.13 | up |
| HORVU.MOREX.r2.5HG0430600 | 155.00 | 84.00 | 78.00 | 522.00 | 330.00 | 609.00 | 4.75 | 2.55 | 2.49 | 15.83 | 9.75 | 19.47 | 0.00 | 1.93 | up |
| HORVU.MOREX.r2.2HG0079390 | 0.00 | 0.00 | 0.00 | 172.00 | 250.00 | 353.00 | 0.00 | 0.00 | 0.00 | 9.45 | 13.45 | 20.49 | 0.00 | 5.75 | up |
| HORVU.MOREX.r2.1HG0017090 | 5.00 | 3.00 | 12.00 | 238.00 | 269.00 | 253.00 | 0.09 | 0.05 | 0.21 | 4.10 | 4.52 | 4.59 | 0.00 | 4.46 | up |
| HORVU.MOREX.r2.4HG0336860 | 14.00 | 5.00 | 0.00 | 1630.00 | 1871.00 | 3834.00 | 0.39 | 0.12 | 0.00 | 45.21 | 50.61 | 112.07 | 0.00 | 2.26 | up |
| HORVU.MOREX.r2.3HG0195620 | 42.00 | 20.00 | 35.00 | 819.00 | 611.00 | 601.00 | 0.36 | 0.16 | 0.31 | 6.98 | 5.07 | 5.39 | 0.00 | 4.00 | up |
| HORVU.MOREX.r2.6HG0463640 | 0.00 | 14.00 | 7.00 | 104.00 | 133.00 | 166.00 | 0.00 | 0.16 | 0.08 | 1.17 | 1.46 | 1.96 | 0.00 | 3.02 | up |
| HORVU.MOREX.r2.6HG0518840 | 159.00 | 183.00 | 247.00 | 1001.00 | 889.00 | 964.00 | 1.00 | 1.15 | 1.62 | 6.25 | 5.41 | 6.34 | 0.00 | 2.13 | up |
| HORVU.MOREX.r2.2HG0105020 | 72.00 | 57.00 | 35.00 | 130.00 | 117.00 | 160.00 | 0.92 | 0.72 | 0.46 | 1.64 | 1.44 | 2.13 | 0.00 | 1.14 | up |
| HORVU.MOREX.r2.3HG0233240 | 303.00 | 453.00 | 276.00 | 712.00 | 664.00 | 904.00 | 5.28 | 7.84 | 5.00 | 12.27 | 11.16 | 16.42 | 0.00 | 1.06 | up |
| HORVU.MOREX.r2.3HG0228420 | 2759.00 | 3030.00 | 2687.00 | 8468.00 | 6836.00 | 8081.00 | 25.34 | 27.64 | 25.70 | 76.83 | 60.46 | 77.25 | 0.00 | 1.40 | up |
| HORVU.MOREX.r2.4HG0275920 | 421.00 | 391.00 | 278.00 | 832.00 | 718.00 | 868.00 | 3.40 | 3.14 | 2.34 | 6.66 | 5.60 | 7.31 | 0.00 | 1.07 | up |
| HORVU.MOREX.r2.7HG0620100 | 0.00 | 4.00 | 9.00 | 283.00 | 150.00 | 275.00 | 0.00 | 0.07 | 0.18 | 5.66 | 2.92 | 5.80 | 0.00 | 4.31 | up |
| HORVU.MOREX.r2.1HG0040660 | 4202.00 | 3356.00 | 2131.00 | 42751.00 | 28263.00 | 43573.00 | 80.39 | 63.75 | 42.43 | 807.69 | 520.56 | 867.37 | 0.00 | 3.31 | up |
| HORVU.MOREX.r2.3HG0256100 | 105.00 | 110.00 | 46.00 | 306.00 | 376.00 | 450.00 | 2.68 | 2.81 | 1.22 | 7.76 | 9.31 | 12.04 | 0.00 | 1.88 | up |
| HORVU.MOREX.r2.2HG0113880 | 919.00 | 720.00 | 593.00 | 1894.00 | 1766.00 | 3064.00 | 17.52 | 13.62 | 11.76 | 35.66 | 32.42 | 60.79 | 0.00 | 1.46 | up |
| HORVU.MOREX.r2.1HG0023190 | 83.00 | 151.00 | 128.00 | 688.00 | 700.00 | 921.00 | 2.40 | 4.35 | 3.87 | 19.75 | 19.58 | 27.86 | 0.00 | 2.48 | up |
| HORVU.MOREX.r2.2HG0164830 | 154.00 | 170.00 | 137.00 | 441.00 | 331.00 | 311.00 | 0.89 | 0.97 | 0.82 | 2.52 | 1.84 | 1.87 | 0.00 | 1.14 | up |
| HORVU.MOREX.r2.4HG0282010 | 1681.00 | 2818.00 | 1895.00 | 8186.00 | 5618.00 | 7676.00 | 19.70 | 32.80 | 23.12 | 94.78 | 63.41 | 93.65 | 0.00 | 1.64 | up |
| HORVU.MOREX.r2.2HG0145460 | 8.00 | 10.00 | 0.00 | 706.00 | 298.00 | 653.00 | 0.09 | 0.11 | 0.00 | 7.87 | 3.24 | 7.67 | 0.00 | 2.73 | up |
| HORVU.MOREX.r2.3HG0260790 | 312.00 | 317.00 | 222.00 | 3346.00 | 2257.00 | 2836.00 | 5.85 | 5.90 | 4.33 | 61.98 | 40.75 | 55.34 | 0.00 | 3.14 | up |
| HORVU.MOREX.r2.1HG0007650 | 301.00 | 268.00 | 177.00 | 1103.00 | 763.00 | 910.00 | 9.89 | 8.72 | 6.03 | 35.78 | 24.12 | 31.10 | 0.00 | 1.76 | up |
| HORVU.MOREX.r2.5HG0412120 | 1365.00 | 2079.00 | 1463.00 | 4079.00 | 3786.00 | 4254.00 | 15.81 | 23.91 | 17.64 | 46.66 | 42.23 | 51.28 | 0.00 | 1.23 | up |
| HORVU.MOREX.r2.5HG0429660 | 33.00 | 34.00 | 18.00 | 59.00 | 123.00 | 126.00 | 0.43 | 0.45 | 0.24 | 0.78 | 1.58 | 1.75 | 0.00 | 1.52 | up |
| HORVU.MOREX.r2.5HG0397900 | 17.00 | 18.00 | 28.00 | 400.00 | 206.00 | 249.00 | 0.20 | 0.22 | 0.37 | 5.03 | 2.52 | 3.29 | 0.00 | 3.25 | up |
| HORVU.MOREX.r2.2HG0091380 | 29.00 | 31.00 | 2.00 | 142.00 | 113.00 | 139.00 | 0.44 | 0.49 | 0.03 | 2.21 | 1.71 | 2.28 | 0.00 | 1.88 | up |
| HORVU.MOREX.r2.7HG0538240 | 77.00 | 231.00 | 124.00 | 399.00 | 345.00 | 501.00 | 0.92 | 2.75 | 1.55 | 4.73 | 3.99 | 6.25 | 0.00 | 1.33 | up |
| HORVU.MOREX.r2.7HG0549070 | 18.00 | 15.00 | 15.00 | 2077.00 | 1481.00 | 1629.00 | 0.61 | 0.50 | 0.53 | 72.27 | 50.24 | 59.72 | 0.00 | 6.25 | up |
| HORVU.MOREX.r2.1HG0057890 | 560.00 | 675.00 | 387.00 | 1445.00 | 1809.00 | 1985.00 | 8.90 | 10.65 | 6.39 | 22.66 | 27.67 | 32.81 | 0.00 | 1.58 | up |
| HORVU.MOREX.r2.7HG0596410 | 352.00 | 290.00 | 298.00 | 607.00 | 646.00 | 847.00 | 3.31 | 2.71 | 2.92 | 5.65 | 5.86 | 8.30 | 0.00 | 1.07 | up |
| HORVU.MOREX.r2.3HG0200840 | 111.00 | 44.00 | 52.00 | 1568.00 | 894.00 | 1068.00 | 1.65 | 0.65 | 0.80 | 23.20 | 12.90 | 16.66 | 0.00 | 3.60 | up |
| HORVU.MOREX.r2.4HG0286830 | 28.00 | 45.00 | 33.00 | 67.00 | 79.00 | 100.00 | 0.27 | 0.43 | 0.34 | 0.64 | 0.74 | 1.01 | 0.01 | 1.04 | up |
| HORVU.MOREX.r2.2HG0100050 | 360.00 | 282.00 | 130.00 | 5012.00 | 3661.00 | 4576.00 | 4.06 | 3.15 | 1.53 | 55.89 | 39.79 | 53.76 | 0.00 | 3.73 | up |
| HORVU.MOREX.r2.4HG0342400 | 57.00 | 40.00 | 28.00 | 71.00 | 134.00 | 248.00 | 2.25 | 1.55 | 1.13 | 2.78 | 5.12 | 10.27 | 0.00 | 1.46 | up |
| HORVU.MOREX.r2.2HG0144240 | 116.00 | 76.00 | 62.00 | 212.00 | 177.00 | 197.00 | 1.42 | 0.93 | 0.79 | 2.59 | 2.11 | 2.53 | 0.00 | 1.07 | up |
| HORVU.MOREX.r2.7HG0604310 | 299.00 | 141.00 | 121.00 | 674.00 | 468.00 | 505.00 | 6.00 | 2.81 | 2.52 | 13.39 | 9.05 | 10.56 | 0.00 | 1.35 | up |
| HORVU.MOREX.r2.3HG0193380 | 677.00 | 1022.00 | 755.00 | 1857.00 | 2258.00 | 3652.00 | 7.04 | 10.55 | 8.18 | 19.07 | 22.61 | 39.52 | 0.00 | 1.52 | up |
| HORVU.MOREX.r2.3HG0233940 | 81.00 | 90.00 | 53.00 | 428.00 | 350.00 | 467.00 | 0.59 | 0.65 | 0.40 | 3.09 | 2.46 | 3.55 | 0.00 | 2.29 | up |
| HORVU.MOREX.r2.6HG0518420 | 350.00 | 384.00 | 203.00 | 637.00 | 642.00 | 960.00 | 3.03 | 3.30 | 1.82 | 5.45 | 5.35 | 8.66 | 0.00 | 1.14 | up |
| HORVU.MOREX.r2.2HG0177960 | 247.00 | 228.00 | 166.00 | 1216.00 | 759.00 | 1357.00 | 1.57 | 1.44 | 1.10 | 7.64 | 4.65 | 8.98 | 0.00 | 2.19 | up |
| HORVU.MOREX.r2.4HG0278010 | 148.00 | 167.00 | 120.00 | 398.00 | 277.00 | 414.00 | 4.29 | 4.82 | 3.62 | 11.42 | 7.75 | 12.52 | 0.00 | 1.21 | up |
| HORVU.MOREX.r2.4HG0316810 | 183.00 | 392.00 | 214.00 | 2232.00 | 1630.00 | 2183.00 | 7.09 | 15.08 | 8.63 | 85.44 | 60.83 | 88.03 | 0.00 | 2.72 | up |
| HORVU.MOREX.r2.7HG0544060 | 25.00 | 28.00 | 59.00 | 188.00 | 165.00 | 292.00 | 1.37 | 1.53 | 3.42 | 10.37 | 8.85 | 16.98 | 0.00 | 2.10 | up |
| HORVU.MOREX.r2.3HG0236740 | 21.00 | 52.00 | 39.00 | 127.00 | 175.00 | 111.00 | 0.18 | 0.46 | 0.35 | 1.11 | 1.50 | 1.03 | 0.00 | 1.60 | up |
| HORVU.MOREX.r2.7HG0547940 | 172.00 | 300.00 | 205.00 | 465.00 | 455.00 | 723.00 | 1.95 | 3.39 | 2.42 | 5.23 | 4.99 | 8.57 | 0.00 | 1.16 | up |
| HORVU.MOREX.r2.5HG0429330 | 476.00 | 461.00 | 346.00 | 1561.00 | 1158.00 | 1614.00 | 10.20 | 9.80 | 7.71 | 33.07 | 23.91 | 36.04 | 0.00 | 1.65 | up |
| HORVU.MOREX.r2.2HG0144950 | 28.00 | 12.00 | 13.00 | 168.00 | 99.00 | 125.00 | 0.16 | 0.06 | 0.07 | 0.92 | 0.53 | 0.72 | 0.00 | 2.37 | up |
| HORVU.MOREX.r2.1HG0075800 | 120.00 | 55.00 | 65.00 | 265.00 | 359.00 | 439.00 | 2.00 | 0.90 | 1.12 | 4.36 | 5.78 | 7.62 | 0.00 | 1.87 | up |
| HORVU.MOREX.r2.1HG0007840 | 12.00 | 48.00 | 15.00 | 10980.00 | 5862.00 | 8026.00 | 0.14 | 0.53 | 0.18 | 122.93 | 63.97 | 94.68 | 0.00 | 5.73 | up |
| HORVU.MOREX.r2.4HG0326540 | 53.00 | 31.00 | 10.00 | 156.00 | 194.00 | 315.00 | 0.94 | 0.55 | 0.18 | 2.75 | 3.34 | 5.85 | 0.00 | 2.20 | up |
| HORVU.MOREX.r2.4HG0333670 | 83.00 | 68.00 | 67.00 | 1174.00 | 844.00 | 1545.00 | 1.16 | 0.94 | 0.98 | 16.37 | 11.46 | 22.69 | 0.00 | 3.72 | up |
| HORVU.MOREX.r2.4HG0339730 | 1.00 | 1.00 | 4.00 | 282.00 | 240.00 | 493.00 | 0.00 | 0.01 | 0.04 | 3.03 | 2.51 | 5.58 | 0.00 | 5.42 | up |
| HORVU.MOREX.r2.7HG0530190 | 98.00 | 205.00 | 227.00 | 963.00 | 857.00 | 1045.00 | 0.82 | 1.71 | 1.98 | 7.97 | 6.92 | 9.12 | 0.00 | 2.21 | up |
| HORVU.MOREX.r2.2HG0108680 | 120.00 | 245.00 | 208.00 | 433.00 | 410.00 | 433.00 | 1.02 | 2.08 | 1.85 | 3.65 | 3.38 | 3.85 | 0.00 | 1.04 | up |
| HORVU.MOREX.r2.5HG0406300 | 346.00 | 337.00 | 146.00 | 4097.00 | 2946.00 | 3248.00 | 3.82 | 3.69 | 1.68 | 44.73 | 31.35 | 37.37 | 0.00 | 3.34 | up |
| HORVU.MOREX.r2.1HG0066230 | 1937.00 | 1797.00 | 1549.00 | 4069.00 | 4463.00 | 5170.00 | 51.43 | 47.37 | 42.82 | 106.70 | 114.08 | 142.83 | 0.00 | 1.30 | up |
| HORVU.MOREX.r2.1HG0060840 | 1138.00 | 548.00 | 536.00 | 2233.00 | 1647.00 | 1812.00 | 17.55 | 8.39 | 8.60 | 34.01 | 24.45 | 29.07 | 0.00 | 1.20 | up |
| HORVU.MOREX.r2.3HG0265830 | 9238.00 | 9184.00 | 8942.00 | 15222.00 | 15805.00 | 27261.00 | 159.90 | 157.83 | 161.13 | 260.19 | 263.38 | 490.97 | 0.00 | 1.00 | up |
| HORVU.MOREX.r2.3HG0261230 | 227.00 | 177.00 | 128.00 | 5936.00 | 4167.00 | 4244.00 | 1.68 | 1.30 | 0.98 | 43.43 | 29.72 | 32.71 | 0.00 | 4.48 | up |
| HORVU.MOREX.r2.6HG0510660 | 8.00 | 23.00 | 17.00 | 250.00 | 101.00 | 201.00 | 0.50 | 1.50 | 1.21 | 16.85 | 6.64 | 14.30 | 0.00 | 2.83 | up |
| HORVU.MOREX.r2.2HG0114490 | 391.00 | 401.00 | 206.00 | 1152.00 | 708.00 | 1060.00 | 3.26 | 3.31 | 1.78 | 9.47 | 5.67 | 9.19 | 0.00 | 1.41 | up |
| HORVU.MOREX.r2.5HG0418820 | 935.00 | 777.00 | 617.00 | 6790.00 | 5913.00 | 9027.00 | 13.03 | 10.76 | 8.95 | 93.52 | 79.38 | 131.00 | 0.00 | 3.04 | up |
| HORVU.MOREX.r2.2HG0175680 | 37.00 | 46.00 | 46.00 | 579.00 | 450.00 | 407.00 | 0.33 | 0.40 | 0.42 | 5.11 | 3.87 | 3.77 | 0.00 | 3.24 | up |
| HORVU.MOREX.r2.5HG0414090 | 520.00 | 488.00 | 369.00 | 1803.00 | 1431.00 | 1306.00 | 7.97 | 7.43 | 5.89 | 27.32 | 21.13 | 20.85 | 0.00 | 1.62 | up |
| HORVU.MOREX.r2.5HG0354290 | 611.00 | 492.00 | 367.00 | 2609.00 | 1406.00 | 2011.00 | 5.84 | 4.67 | 3.65 | 24.64 | 12.94 | 20.01 | 0.00 | 1.87 | up |
| HORVU.MOREX.r2.2HG0100950 | 567.00 | 331.00 | 138.00 | 2807.00 | 2297.00 | 2680.00 | 6.88 | 3.99 | 1.74 | 33.65 | 26.85 | 33.85 | 0.00 | 2.52 | up |
| HORVU.MOREX.r2.5HG0397090 | 7.00 | 4.00 | 1.00 | 95.00 | 69.00 | 66.00 | 0.30 | 0.20 | 0.01 | 4.74 | 3.35 | 3.44 | 0.00 | 3.12 | up |
| HORVU.MOREX.r2.2HG0132800 | 835.00 | 778.00 | 669.00 | 3427.00 | 3252.00 | 3523.00 | 9.64 | 8.92 | 8.04 | 39.07 | 36.14 | 42.32 | 0.00 | 2.08 | up |
| HORVU.MOREX.r2.5HG0408020 | 83.00 | 46.00 | 48.00 | 148.00 | 157.00 | 143.00 | 1.32 | 0.73 | 0.79 | 2.32 | 2.41 | 2.38 | 0.00 | 1.17 | up |
| HORVU.MOREX.r2.5HG0388110 | 47.00 | 134.00 | 88.00 | 186.00 | 279.00 | 177.00 | 0.49 | 1.41 | 0.97 | 1.94 | 2.84 | 1.95 | 0.01 | 1.06 | up |
| HORVU.MOREX.r2.5HG0406540 | 80.00 | 208.00 | 143.00 | 834.00 | 927.00 | 1601.00 | 0.85 | 2.21 | 1.59 | 8.82 | 9.55 | 17.83 | 0.00 | 2.62 | up |
| HORVU.MOREX.r2.5HG0350720 | 2318.00 | 2563.00 | 2320.00 | 4373.00 | 5280.00 | 6641.00 | 13.31 | 14.61 | 13.87 | 24.79 | 29.18 | 39.67 | 0.00 | 1.10 | up |
| Hordeum_vulgare_newGene_5269 | 5.00 | 4.00 | 4.00 | 83.00 | 79.00 | 61.00 | 0.05 | 0.04 | 0.04 | 0.83 | 0.77 | 0.64 | 0.00 | 3.16 | up |
| HORVU.MOREX.r2.1HG0066800 | 136.00 | 127.00 | 94.00 | 331.00 | 289.00 | 295.00 | 1.26 | 1.17 | 0.91 | 3.05 | 2.59 | 2.87 | 0.00 | 1.26 | up |
| HORVU.MOREX.r2.5HG0367880 | 231.00 | 352.00 | 191.00 | 935.00 | 800.00 | 1046.00 | 8.02 | 12.17 | 6.92 | 32.15 | 26.80 | 37.87 | 0.00 | 1.72 | up |
| HORVU.MOREX.r2.3HG0263870 | 705.00 | 1100.00 | 768.00 | 6805.00 | 5059.00 | 6418.00 | 11.38 | 17.63 | 12.91 | 108.56 | 78.68 | 107.88 | 0.00 | 2.69 | up |
| HORVU.MOREX.r2.2HG0131100 | 224.00 | 229.00 | 219.00 | 845.00 | 847.00 | 1028.00 | 3.18 | 3.23 | 3.24 | 11.89 | 11.62 | 15.24 | 0.00 | 1.92 | up |
| HORVU.MOREX.r2.7HG0551040 | 2000.00 | 2051.00 | 1598.00 | 6464.00 | 4821.00 | 6516.00 | 31.52 | 32.10 | 26.21 | 100.60 | 73.15 | 106.85 | 0.00 | 1.57 | up |
| HORVU.MOREX.r2.7HG0594640 | 70.00 | 168.00 | 89.00 | 568.00 | 508.00 | 737.00 | 0.77 | 1.84 | 1.02 | 6.20 | 5.40 | 8.47 | 0.00 | 2.22 | up |
| HORVU.MOREX.r2.3HG0260690 | 2710.00 | 2890.00 | 2674.00 | 6323.00 | 5202.00 | 7385.00 | 17.95 | 19.00 | 18.44 | 41.36 | 33.17 | 50.90 | 0.00 | 1.12 | up |
| HORVU.MOREX.r2.7HG0577550 | 26.00 | 38.00 | 16.00 | 101.00 | 76.00 | 77.00 | 2.85 | 4.15 | 1.80 | 11.00 | 8.06 | 8.77 | 0.00 | 1.40 | up |
| HORVU.MOREX.r2.3HG0191040 | 3059.00 | 3032.00 | 2351.00 | 7194.00 | 6757.00 | 6941.00 | 13.53 | 13.31 | 10.82 | 31.42 | 28.77 | 31.94 | 0.00 | 1.24 | up |
| HORVU.MOREX.r2.6HG0473310 | 1039.00 | 1360.00 | 842.00 | 2523.00 | 2076.00 | 2487.00 | 17.26 | 22.42 | 14.56 | 41.40 | 33.21 | 42.99 | 0.00 | 1.06 | up |
| HORVU.MOREX.r2.6HG0474930 | 1901.00 | 1846.00 | 1093.00 | 12505.00 | 9153.00 | 13147.00 | 29.87 | 28.80 | 17.87 | 194.10 | 138.51 | 215.01 | 0.00 | 2.67 | up |
| HORVU.MOREX.r2.2HG0140160 | 98.00 | 123.00 | 103.00 | 384.00 | 326.00 | 345.00 | 1.66 | 2.07 | 1.82 | 6.44 | 5.34 | 6.10 | 0.00 | 1.60 | up |
| HORVU.MOREX.r2.6HG0494040 | 212.00 | 126.00 | 86.00 | 413.00 | 415.00 | 491.00 | 1.63 | 0.96 | 0.68 | 3.13 | 3.08 | 3.93 | 0.00 | 1.45 | up |
| HORVU.MOREX.r2.5HG0413210 | 3069.00 | 2622.00 | 1641.00 | 5673.00 | 5291.00 | 5273.00 | 48.11 | 40.80 | 26.78 | 87.81 | 79.84 | 86.00 | 0.00 | 1.06 | up |
| HORVU.MOREX.r2.1HG0053820 | 138.00 | 96.00 | 97.00 | 803.00 | 522.00 | 800.00 | 1.55 | 1.07 | 1.13 | 8.95 | 5.67 | 9.39 | 0.00 | 2.47 | up |
| HORVU.MOREX.r2.2HG0150420 | 1056.00 | 1112.00 | 1206.00 | 3425.00 | 2807.00 | 2479.00 | 21.17 | 22.12 | 25.16 | 67.82 | 54.18 | 51.72 | 0.00 | 1.29 | up |
| HORVU.MOREX.r2.5HG0407260 | 484.00 | 569.00 | 533.00 | 2215.00 | 1573.00 | 1781.00 | 5.07 | 5.92 | 5.81 | 22.92 | 15.86 | 19.42 | 0.00 | 1.72 | up |
| HORVU.MOREX.r2.3HG0203750 | 271.00 | 450.00 | 324.00 | 1462.00 | 1064.00 | 1296.00 | 18.31 | 30.20 | 22.77 | 97.59 | 69.27 | 91.16 | 0.00 | 1.75 | up |
| HORVU.MOREX.r2.5HG0382660 | 524.00 | 581.00 | 414.00 | 1091.00 | 1077.00 | 1235.00 | 4.50 | 4.95 | 3.70 | 9.25 | 8.90 | 11.04 | 0.00 | 1.10 | up |
| HORVU.MOREX.r2.2HG0166840 | 65.00 | 136.00 | 80.00 | 776.00 | 576.00 | 140.00 | 0.85 | 1.77 | 1.10 | 10.12 | 7.33 | 1.91 | 0.00 | 1.89 | up |
| HORVU.MOREX.r2.7HG0538640 | 185.00 | 183.00 | 289.00 | 755.00 | 875.00 | 847.00 | 1.62 | 1.59 | 2.65 | 6.56 | 7.40 | 7.75 | 0.00 | 1.77 | up |
| HORVU.MOREX.r2.1HG0012070 | 4.00 | 2.00 | 12.00 | 163.00 | 125.00 | 186.00 | 0.04 | 0.02 | 0.12 | 1.64 | 1.22 | 1.97 | 0.00 | 3.65 | up |
| HORVU.MOREX.r2.5HG0384540 | 33.00 | 27.00 | 67.00 | 101.00 | 153.00 | 121.00 | 0.60 | 0.48 | 1.25 | 1.81 | 2.67 | 2.28 | 0.00 | 1.27 | up |
| HORVU.MOREX.r2.3HG0246570 | 214.00 | 150.00 | 123.00 | 451.00 | 364.00 | 371.00 | 2.24 | 1.56 | 1.34 | 4.67 | 3.68 | 4.05 | 0.00 | 1.17 | up |
| HORVU.MOREX.r2.4HG0320060 | 38.00 | 24.00 | 8.00 | 231.00 | 152.00 | 103.00 | 0.37 | 0.23 | 0.08 | 2.23 | 1.43 | 1.05 | 0.00 | 2.17 | up |
| HORVU.MOREX.r2.4HG0327870 | 15.00 | 3.00 | 9.00 | 86.00 | 137.00 | 141.00 | 0.13 | 0.03 | 0.08 | 0.79 | 1.23 | 1.36 | 0.00 | 2.90 | up |
| HORVU.MOREX.r2.1HG0015680 | 161.00 | 195.00 | 131.00 | 715.00 | 358.00 | 550.00 | 2.13 | 2.56 | 1.80 | 9.35 | 4.56 | 7.57 | 0.00 | 1.57 | up |
| HORVU.MOREX.r2.7HG0600290 | 0.00 | 17.00 | 8.00 | 229.00 | 118.00 | 158.00 | 0.00 | 0.35 | 0.16 | 4.94 | 2.47 | 3.59 | 0.00 | 2.98 | up |
| HORVU.MOREX.r2.1HG0011520 | 50.00 | 51.00 | 35.00 | 407.00 | 188.00 | 228.00 | 2.65 | 2.66 | 1.92 | 21.41 | 9.62 | 12.62 | 0.00 | 2.26 | up |
| HORVU.MOREX.r2.2HG0092490 | 390.00 | 160.00 | 104.00 | 4715.00 | 5319.00 | 9878.00 | 7.24 | 2.95 | 2.00 | 86.51 | 95.14 | 190.97 | 0.00 | 3.37 | up |
| HORVU.MOREX.r2.7HG0562920 | 372.00 | 459.00 | 331.00 | 988.00 | 919.00 | 1312.00 | 3.93 | 4.81 | 3.64 | 10.31 | 9.35 | 14.43 | 0.00 | 1.38 | up |
| HORVU.MOREX.r2.3HG0200100 | 910.00 | 920.00 | 421.00 | 7989.00 | 5397.00 | 7915.00 | 11.26 | 11.30 | 5.41 | 97.62 | 64.29 | 101.90 | 0.00 | 2.98 | up |
| HORVU.MOREX.r2.2HG0165780 | 39.00 | 23.00 | 24.00 | 151.00 | 88.00 | 153.00 | 0.43 | 0.25 | 0.28 | 1.68 | 0.95 | 1.80 | 0.00 | 1.85 | up |
| HORVU.MOREX.r2.5HG0394280 | 170.00 | 130.00 | 30.00 | 738.00 | 391.00 | 644.00 | 4.75 | 3.61 | 0.86 | 20.36 | 10.50 | 18.71 | 0.00 | 1.95 | up |
| HORVU.MOREX.r2.6HG0524290 | 20132.00 | 24050.00 | 14984.00 | 54314.00 | 53479.00 | 71469.00 | 801.96 | 951.19 | 621.42 | 2136.64 | 2050.95 | 2962.26 | 0.00 | 1.51 | up |
| HORVU.MOREX.r2.2HG0082310 | 32.00 | 25.00 | 33.00 | 184.00 | 223.00 | 298.00 | 0.26 | 0.20 | 0.28 | 1.47 | 1.74 | 2.51 | 0.00 | 2.64 | up |
| HORVU.MOREX.r2.3HG0201020 | 775.00 | 1117.00 | 533.00 | 2435.00 | 2021.00 | 2928.00 | 13.41 | 19.19 | 9.59 | 41.61 | 33.67 | 52.72 | 0.00 | 1.49 | up |
| HORVU.MOREX.r2.7HG0607560 | 25.00 | 8.00 | 0.00 | 696.00 | 461.00 | 511.00 | 0.29 | 0.09 | 0.00 | 8.20 | 5.29 | 6.35 | 0.01 | 1.64 | up |
| HORVU.MOREX.r2.4HG0322390 | 103.00 | 102.00 | 108.00 | 673.00 | 510.00 | 789.00 | 2.91 | 2.87 | 3.18 | 18.84 | 13.90 | 23.27 | 0.00 | 2.48 | up |
| HORVU.MOREX.r2.3HG0266030 | 1178.00 | 1039.00 | 1607.00 | 24372.00 | 19743.00 | 22376.00 | 19.35 | 16.94 | 27.47 | 395.42 | 312.28 | 382.49 | 0.00 | 3.91 | up |
| HORVU.MOREX.r2.2HG0144330 | 866.00 | 1136.00 | 961.00 | 1374.00 | 2291.00 | 2823.00 | 10.80 | 14.08 | 12.48 | 16.93 | 27.52 | 36.65 | 0.00 | 1.03 | up |
| HORVU.MOREX.r2.2HG0159360 | 513.00 | 634.00 | 536.00 | 1554.00 | 891.00 | 1176.00 | 5.12 | 6.27 | 5.56 | 15.30 | 8.55 | 12.20 | 0.00 | 1.02 | up |
| HORVU.MOREX.r2.UnG0636550 | 3.00 | 8.00 | 11.00 | 60.00 | 62.00 | 62.00 | 0.09 | 0.26 | 0.37 | 2.04 | 2.08 | 2.24 | 0.00 | 2.34 | up |
| Hordeum_vulgare_newGene_4487 | 115.00 | 219.00 | 101.00 | 619.00 | 591.00 | 420.00 | 0.70 | 1.29 | 0.87 | 3.95 | 4.08 | 2.96 | 0.00 | 1.72 | up |
| HORVU.MOREX.r2.7HG0600800 | 37.00 | 29.00 | 12.00 | 133.00 | 78.00 | 75.00 | 0.71 | 0.56 | 0.24 | 2.55 | 1.45 | 1.52 | 0.00 | 1.49 | up |
| HORVU.MOREX.r2.2HG0084430 | 238.00 | 343.00 | 226.00 | 750.00 | 680.00 | 785.00 | 1.76 | 2.51 | 1.74 | 5.47 | 4.83 | 6.03 | 0.00 | 1.37 | up |
| HORVU.MOREX.r2.7HG0535730 | 5105.00 | 4172.00 | 3422.00 | 10929.00 | 9005.00 | 11555.00 | 58.67 | 47.60 | 40.94 | 124.05 | 99.65 | 138.19 | 0.00 | 1.23 | up |
| HORVU.MOREX.r2.6HG0511810 | 232.00 | 290.00 | 112.00 | 17047.00 | 9769.00 | 17904.00 | 2.77 | 3.44 | 1.38 | 201.45 | 112.55 | 222.93 | 0.00 | 5.56 | up |
| HORVU.MOREX.r2.6HG0494420 | 375.00 | 400.00 | 338.00 | 957.00 | 797.00 | 701.00 | 3.05 | 3.23 | 2.87 | 7.71 | 6.26 | 5.95 | 0.00 | 1.07 | up |
| HORVU.MOREX.r2.2HG0161220 | 28.00 | 30.00 | 5.00 | 5582.00 | 2675.00 | 4572.00 | 0.28 | 0.30 | 0.05 | 56.04 | 26.18 | 48.36 | 0.00 | 4.76 | up |
| HORVU.MOREX.r2.1HG0053730 | 2005.00 | 1345.00 | 658.00 | 7730.00 | 6116.00 | 7900.00 | 128.13 | 85.31 | 43.72 | 487.82 | 376.26 | 525.28 | 0.00 | 2.18 | up |
| HORVU.MOREX.r2.6HG0512670 | 64.00 | 91.00 | 59.00 | 396.00 | 264.00 | 315.00 | 4.07 | 5.72 | 3.90 | 24.95 | 16.22 | 20.91 | 0.00 | 2.01 | up |
| HORVU.MOREX.r2.3HG0263370 | 895.00 | 919.00 | 573.00 | 4082.00 | 2607.00 | 3745.00 | 11.61 | 11.84 | 7.74 | 52.33 | 32.57 | 50.57 | 0.00 | 1.99 | up |
| HORVU.MOREX.r2.5HG0363420 | 124.00 | 92.00 | 25.00 | 325.00 | 166.00 | 359.00 | 3.10 | 2.27 | 0.63 | 8.03 | 4.00 | 9.35 | 0.00 | 1.43 | up |
| HORVU.MOREX.r2.4HG0348400 | 6.00 | 6.00 | 2.00 | 277.00 | 101.00 | 158.00 | 0.03 | 0.03 | 0.01 | 1.54 | 0.55 | 0.92 | 0.00 | 3.93 | up |
| HORVU.MOREX.r2.4HG0332850 | 756.00 | 1142.00 | 597.00 | 23712.00 | 13739.00 | 16268.00 | 22.90 | 34.33 | 18.80 | 709.30 | 400.64 | 512.71 | 0.00 | 4.13 | up |
| HORVU.MOREX.r2.5HG0400750 | 18.00 | 16.00 | 8.00 | 739.00 | 743.00 | 622.00 | 0.30 | 0.28 | 0.14 | 12.78 | 12.53 | 11.32 | 0.00 | 5.20 | up |
| HORVU.MOREX.r2.6HG0451220 | 31.00 | 52.00 | 42.00 | 84.00 | 108.00 | 116.00 | 0.19 | 0.32 | 0.27 | 0.51 | 0.65 | 0.75 | 0.00 | 1.13 | up |
| HORVU.MOREX.r2.4HG0328610 | 2116.00 | 907.00 | 1071.00 | 7101.00 | 4275.00 | 8137.00 | 25.22 | 10.72 | 13.28 | 83.57 | 49.04 | 100.89 | 0.00 | 1.78 | up |
| HORVU.MOREX.r2.1HG0016060 | 273.00 | 299.00 | 324.00 | 1458.00 | 719.00 | 1289.00 | 3.34 | 3.64 | 4.14 | 17.70 | 8.50 | 16.48 | 0.00 | 1.78 | up |
| HORVU.MOREX.r2.3HG0190830 | 24.00 | 40.00 | 40.00 | 131.00 | 141.00 | 115.00 | 0.14 | 0.22 | 0.23 | 0.74 | 0.77 | 0.68 | 0.00 | 1.68 | up |
| HORVU.MOREX.r2.5HG0426300 | 799.00 | 677.00 | 414.00 | 2573.00 | 1659.00 | 2095.00 | 9.12 | 7.67 | 4.92 | 29.03 | 18.25 | 24.90 | 0.00 | 1.60 | up |
| HORVU.MOREX.r2.7HG0550810 | 117.00 | 296.00 | 177.00 | 1839.00 | 951.00 | 1319.00 | 0.92 | 2.32 | 1.45 | 14.33 | 7.22 | 10.82 | 0.00 | 2.50 | up |
| HORVU.MOREX.r2.2HG0173540 | 25.00 | 35.00 | 49.00 | 74.00 | 112.00 | 122.00 | 1.76 | 2.50 | 3.66 | 5.29 | 7.77 | 9.17 | 0.00 | 1.25 | up |
| HORVU.MOREX.r2.7HG0556360 | 4818.00 | 5843.00 | 5000.00 | 13616.00 | 14641.00 | 17944.00 | 45.34 | 54.59 | 48.99 | 126.54 | 132.65 | 175.71 | 0.00 | 1.49 | up |
| HORVU.MOREX.r2.2HG0160620 | 2848.00 | 2452.00 | 1905.00 | 6646.00 | 4955.00 | 5460.00 | 50.96 | 43.56 | 35.48 | 117.45 | 85.35 | 101.66 | 0.00 | 1.16 | up |
| HORVU.MOREX.r2.4HG0288590 | 1182.00 | 1795.00 | 1739.00 | 3129.00 | 5843.00 | 6044.00 | 18.08 | 27.28 | 27.70 | 47.29 | 86.09 | 96.25 | 0.00 | 1.52 | up |
| HORVU.MOREX.r2.6HG0526360 | 13.00 | 24.00 | 19.00 | 98.00 | 71.00 | 69.00 | 0.30 | 0.55 | 0.46 | 2.28 | 1.59 | 1.68 | 0.00 | 1.75 | up |
| HORVU.MOREX.r2.2HG0169700 | 333.00 | 845.00 | 653.00 | 1325.00 | 1549.00 | 1621.00 | 2.34 | 5.89 | 4.78 | 9.20 | 10.48 | 11.86 | 0.00 | 1.17 | up |
| HORVU.MOREX.r2.3HG0204200 | 153.00 | 69.00 | 23.00 | 5566.00 | 3527.00 | 6496.00 | 1.73 | 0.77 | 0.27 | 62.55 | 38.65 | 76.93 | 0.00 | 3.68 | up |
| HORVU.MOREX.r2.6HG0453980 | 112.00 | 108.00 | 83.00 | 181.00 | 209.00 | 267.00 | 1.31 | 1.26 | 1.01 | 2.10 | 2.36 | 3.27 | 0.00 | 1.01 | up |
| HORVU.MOREX.r2.5HG0401270 | 1741.00 | 2540.00 | 1912.00 | 4407.00 | 17080.00 | 22720.00 | 21.31 | 30.87 | 24.37 | 53.28 | 201.33 | 289.45 | 0.00 | 1.96 | up |
| HORVU.MOREX.r2.7HG0530410 | 208.00 | 120.00 | 104.00 | 361.00 | 394.00 | 400.00 | 1.44 | 0.83 | 0.75 | 2.48 | 2.64 | 2.89 | 0.00 | 1.27 | up |
| HORVU.MOREX.r2.3HG0183440 | 492.00 | 539.00 | 486.00 | 1496.00 | 977.00 | 1281.00 | 4.27 | 4.64 | 4.40 | 12.84 | 8.17 | 11.59 | 0.00 | 1.22 | up |
| HORVU.MOREX.r2.1HG0042460 | 120.00 | 68.00 | 74.00 | 618.00 | 402.00 | 540.00 | 3.20 | 1.81 | 2.06 | 16.35 | 10.37 | 15.03 | 0.00 | 2.33 | up |
| HORVU.MOREX.r2.6HG0453490 | 1757.00 | 3450.00 | 2573.00 | 20296.00 | 24272.00 | 41126.00 | 20.64 | 40.24 | 31.46 | 235.48 | 274.55 | 502.77 | 0.00 | 3.16 | up |
| HORVU.MOREX.r2.5HG0427360 | 9.00 | 18.00 | 14.00 | 2502.00 | 1486.00 | 1841.00 | 0.13 | 0.29 | 0.23 | 41.41 | 23.97 | 32.10 | 0.00 | 6.47 | up |
| HORVU.MOREX.r2.UnG0626920 | 0.00 | 0.00 | 0.00 | 941.00 | 795.00 | 1293.00 | 0.00 | 0.00 | 0.00 | 23.90 | 19.68 | 34.61 | 0.00 | 7.61 | up |
| HORVU.MOREX.r2.4HG0341380 | 1535.00 | 1394.00 | 916.00 | 4369.00 | 2711.00 | 4145.00 | 16.37 | 14.77 | 10.17 | 46.03 | 27.85 | 46.02 | 0.00 | 1.43 | up |
| HORVU.MOREX.r2.7HG0606060 | 29.00 | 52.00 | 51.00 | 200.00 | 94.00 | 226.00 | 0.44 | 0.77 | 0.79 | 3.00 | 1.37 | 3.57 | 0.00 | 1.65 | up |
| HORVU.MOREX.r2.3HG0257390 | 557.00 | 799.00 | 703.00 | 2684.00 | 1895.00 | 2279.00 | 6.94 | 9.89 | 9.13 | 33.08 | 22.77 | 29.58 | 0.00 | 1.64 | up |
| HORVU.MOREX.r2.2HG0147030 | 626.00 | 988.00 | 694.00 | 2088.00 | 1489.00 | 1557.00 | 10.54 | 16.52 | 12.18 | 34.75 | 24.16 | 27.30 | 0.00 | 1.07 | up |
| HORVU.MOREX.r2.2HG0173360 | 615.00 | 390.00 | 227.00 | 944.00 | 876.00 | 1042.00 | 27.72 | 17.43 | 10.62 | 42.00 | 37.98 | 48.86 | 0.00 | 1.07 | up |
| HORVU.MOREX.r2.6HG0488210 | 3411.00 | 2631.00 | 2369.00 | 7744.00 | 6746.00 | 8510.00 | 37.00 | 28.34 | 26.76 | 82.98 | 70.46 | 96.07 | 0.00 | 1.36 | up |
| HORVU.MOREX.r2.2HG0169850 | 34.00 | 36.00 | 37.00 | 409.00 | 354.00 | 611.00 | 0.58 | 0.61 | 0.65 | 7.02 | 5.92 | 11.06 | 0.00 | 3.34 | up |
| HORVU.MOREX.r2.5HG0398150 | 3659.00 | 3485.00 | 2424.00 | 7703.00 | 8776.00 | 7372.00 | 41.72 | 39.46 | 28.78 | 86.74 | 96.34 | 87.47 | 0.00 | 1.24 | up |
| HORVU.MOREX.r2.2HG0089450 | 360.00 | 560.00 | 456.00 | 1462.00 | 1299.00 | 1529.00 | 3.05 | 4.70 | 4.02 | 12.22 | 10.59 | 13.46 | 0.00 | 1.55 | up |
| HORVU.MOREX.r2.1HG0077380 | 2655.00 | 4051.00 | 3009.00 | 7980.00 | 9143.00 | 11148.00 | 20.58 | 31.19 | 24.29 | 61.11 | 68.26 | 89.95 | 0.00 | 1.45 | up |
| HORVU.MOREX.r2.4HG0341550 | 199.00 | 391.00 | 210.00 | 628.00 | 519.00 | 651.00 | 3.57 | 6.97 | 3.93 | 11.16 | 8.98 | 12.19 | 0.00 | 1.06 | up |
| HORVU.MOREX.r2.2HG0168660 | 34.00 | 32.00 | 5.00 | 501.00 | 314.00 | 564.00 | 0.44 | 0.40 | 0.06 | 6.35 | 3.88 | 7.52 | 0.00 | 3.45 | up |
| HORVU.MOREX.r2.1HG0066640 | 89.00 | 78.00 | 45.00 | 2013.00 | 1298.00 | 1413.00 | 0.97 | 0.84 | 0.51 | 21.77 | 13.69 | 16.10 | 0.00 | 4.15 | up |
| HORVU.MOREX.r2.5HG0404290 | 175.00 | 201.00 | 219.00 | 854.00 | 492.00 | 941.00 | 2.63 | 3.01 | 3.43 | 12.73 | 7.15 | 14.78 | 0.00 | 1.77 | up |
| HORVU.MOREX.r2.6HG0501280 | 313.00 | 70.00 | 11.00 | 7872.00 | 4622.00 | 7518.00 | 4.13 | 0.91 | 0.14 | 102.52 | 58.68 | 103.16 | 0.00 | 1.94 | up |
| HORVU.MOREX.r2.7HG0534180 | 5801.00 | 1858.00 | 2386.00 | 10211.00 | 12738.00 | 21039.00 | 124.03 | 39.44 | 53.11 | 215.61 | 262.21 | 468.08 | 0.01 | 1.48 | up |
| HORVU.MOREX.r2.2HG0178460 | 1.00 | 0.00 | 4.00 | 198.00 | 74.00 | 120.00 | 0.02 | 0.00 | 0.10 | 4.85 | 1.77 | 3.10 | 0.00 | 4.05 | up |
| HORVU.MOREX.r2.1HG0032950 | 87.00 | 41.00 | 54.00 | 169.00 | 293.00 | 353.00 | 0.67 | 0.31 | 0.43 | 1.28 | 2.18 | 2.84 | 0.00 | 1.84 | up |
| HORVU.MOREX.r2.4HG0288990 | 186.00 | 100.00 | 88.00 | 449.00 | 330.00 | 426.00 | 9.72 | 5.20 | 4.76 | 23.20 | 16.62 | 23.18 | 0.00 | 1.49 | up |
| HORVU.MOREX.r2.2HG0159130 | 439.00 | 599.00 | 432.00 | 1592.00 | 1243.00 | 1398.00 | 5.14 | 6.97 | 5.26 | 18.43 | 14.02 | 17.05 | 0.00 | 1.44 | up |
| HORVU.MOREX.r2.1HG0077220 | 81.00 | 60.00 | 37.00 | 487.00 | 243.00 | 319.00 | 0.93 | 0.68 | 0.44 | 5.51 | 2.68 | 3.80 | 0.00 | 2.23 | up |
| HORVU.MOREX.r2.7HG0528860 | 1689.00 | 2211.00 | 2655.00 | 4071.00 | 4328.00 | 5670.00 | 21.12 | 27.45 | 34.56 | 50.28 | 52.11 | 73.78 | 0.00 | 1.01 | up |
| HORVU.MOREX.r2.6HG0485060 | 50.00 | 74.00 | 31.00 | 119.00 | 146.00 | 114.00 | 0.41 | 0.61 | 0.27 | 0.97 | 1.16 | 0.98 | 0.00 | 1.12 | up |
| HORVU.MOREX.r2.1HG0016790 | 422.00 | 385.00 | 223.00 | 9782.00 | 5684.00 | 6742.00 | 10.95 | 9.90 | 6.00 | 250.80 | 142.08 | 182.13 | 0.00 | 4.11 | up |
| HORVU.MOREX.r2.2HG0136370 | 5009.00 | 3933.00 | 4675.00 | 10703.00 | 9773.00 | 11839.00 | 70.19 | 54.72 | 68.21 | 148.12 | 131.85 | 172.62 | 0.00 | 1.17 | up |
| HORVU.MOREX.r2.6HG0520700 | 2129.00 | 1648.00 | 1161.00 | 6504.00 | 6321.00 | 11127.00 | 34.68 | 26.65 | 19.69 | 104.63 | 99.14 | 188.61 | 0.00 | 2.09 | up |
| HORVU.MOREX.r2.3HG0272150 | 143.00 | 189.00 | 150.00 | 433.00 | 541.00 | 534.00 | 1.43 | 1.88 | 1.57 | 4.29 | 5.23 | 5.57 | 0.00 | 1.55 | up |
| HORVU.MOREX.r2.2HG0137960 | 46.00 | 34.00 | 24.00 | 145.00 | 85.00 | 154.00 | 0.61 | 0.44 | 0.32 | 1.92 | 1.10 | 2.15 | 0.00 | 1.59 | up |
| HORVU.MOREX.r2.1HG0050170 | 9.00 | 9.00 | 12.00 | 77.00 | 57.00 | 98.00 | 0.05 | 0.05 | 0.07 | 0.42 | 0.30 | 0.56 | 0.00 | 2.37 | up |
| HORVU.MOREX.r2.7HG0558200 | 183.00 | 135.00 | 141.00 | 2190.00 | 2029.00 | 2548.00 | 2.01 | 1.48 | 1.62 | 23.91 | 21.59 | 29.31 | 0.00 | 3.70 | up |
| HORVU.MOREX.r2.4HG0345750 | 93.00 | 68.00 | 65.00 | 245.00 | 142.00 | 147.00 | 0.80 | 0.58 | 0.59 | 2.09 | 1.18 | 1.32 | 0.00 | 1.08 | up |
| HORVU.MOREX.r2.7HG0554980 | 64.00 | 17.00 | 30.00 | 176.00 | 120.00 | 255.00 | 2.13 | 0.55 | 1.02 | 5.82 | 3.85 | 8.90 | 0.00 | 1.81 | up |
| HORVU.MOREX.r2.1HG0011530 | 398.00 | 307.00 | 258.00 | 919.00 | 575.00 | 889.00 | 22.00 | 16.86 | 14.83 | 50.24 | 30.62 | 51.21 | 0.00 | 1.19 | up |
| HORVU.MOREX.r2.3HG0247070 | 374.00 | 435.00 | 350.00 | 9752.00 | 7111.00 | 10725.00 | 6.85 | 7.91 | 6.68 | 176.68 | 125.60 | 204.73 | 0.00 | 4.38 | up |
| HORVU.MOREX.r2.5HG0404090 | 287.00 | 175.00 | 156.00 | 1272.00 | 575.00 | 1111.00 | 7.66 | 4.61 | 4.33 | 33.49 | 14.75 | 30.81 | 0.00 | 1.99 | up |
| HORVU.MOREX.r2.1HG0000980 | 62.00 | 48.00 | 34.00 | 150.00 | 122.00 | 124.00 | 2.05 | 1.57 | 1.14 | 4.89 | 3.85 | 4.25 | 0.00 | 1.28 | up |
| HORVU.MOREX.r2.7HG0591450 | 1908.00 | 1634.00 | 1347.00 | 5408.00 | 6757.00 | 7177.00 | 22.69 | 19.30 | 16.67 | 63.51 | 77.36 | 88.81 | 0.00 | 1.88 | up |
| HORVU.MOREX.r2.3HG0244560 | 54.00 | 137.00 | 86.00 | 359.00 | 274.00 | 324.00 | 0.38 | 0.95 | 0.62 | 2.48 | 1.84 | 2.36 | 0.00 | 1.59 | up |
| HORVU.MOREX.r2.7HG0544940 | 296.00 | 186.00 | 95.00 | 1818.00 | 1502.00 | 2067.00 | 2.01 | 1.25 | 0.67 | 12.21 | 9.84 | 14.63 | 0.00 | 2.87 | up |
| HORVU.MOREX.r2.7HG0565210 | 344.00 | 328.00 | 254.00 | 1284.00 | 904.00 | 1299.00 | 2.72 | 2.57 | 2.09 | 10.05 | 6.89 | 10.71 | 0.00 | 1.79 | up |
| HORVU.MOREX.r2.5HG0403450 | 97.00 | 114.00 | 94.00 | 262.00 | 284.00 | 276.00 | 1.76 | 2.04 | 1.78 | 4.70 | 4.96 | 5.21 | 0.00 | 1.34 | up |
| HORVU.MOREX.r2.3HG0186300 | 1928.00 | 1339.00 | 870.00 | 5585.00 | 4551.00 | 5503.00 | 29.58 | 20.40 | 13.89 | 84.63 | 67.22 | 87.86 | 0.00 | 1.76 | up |
| HORVU.MOREX.r2.2HG0092440 | 2784.00 | 1666.00 | 1086.00 | 9285.00 | 10008.00 | 18032.00 | 51.23 | 30.44 | 20.80 | 168.74 | 177.32 | 345.31 | 0.00 | 2.13 | up |
| HORVU.MOREX.r2.5HG0392490 | 46.00 | 82.00 | 49.00 | 871.00 | 588.00 | 748.00 | 0.50 | 0.91 | 0.56 | 9.60 | 6.32 | 8.68 | 0.00 | 3.37 | up |
| HORVU.MOREX.r2.3HG0275390 | 108.00 | 83.00 | 66.00 | 326.00 | 342.00 | 388.00 | 1.82 | 1.38 | 1.15 | 5.44 | 5.58 | 6.85 | 0.00 | 1.88 | up |
| HORVU.MOREX.r2.2HG0162330 | 0.00 | 0.00 | 3.00 | 246.00 | 501.00 | 286.00 | 0.00 | 0.00 | 0.05 | 5.34 | 10.61 | 6.54 | 0.00 | 5.79 | up |
| HORVU.MOREX.r2.5HG0393720 | 30.00 | 19.00 | 25.00 | 74.00 | 68.00 | 54.00 | 1.67 | 1.03 | 1.42 | 4.09 | 3.69 | 3.14 | 0.00 | 1.17 | up |
| HORVU.MOREX.r2.7HG0532400 | 416.00 | 448.00 | 313.00 | 1199.00 | 844.00 | 1151.00 | 3.47 | 3.71 | 2.71 | 9.88 | 6.77 | 9.99 | 0.00 | 1.35 | up |
| HORVU.MOREX.r2.2HG0163060 | 326.00 | 287.00 | 166.00 | 569.00 | 780.00 | 787.00 | 20.12 | 17.63 | 10.64 | 34.76 | 46.45 | 50.66 | 0.00 | 1.32 | up |
| HORVU.MOREX.r2.3HG0262580 | 34.00 | 9.00 | 3.00 | 452.00 | 333.00 | 308.00 | 0.66 | 0.16 | 0.06 | 8.74 | 6.26 | 6.26 | 0.00 | 3.42 | up |
| HORVU.MOREX.r2.5HG0396590 | 497.00 | 584.00 | 371.00 | 956.00 | 1131.00 | 1192.00 | 5.34 | 6.23 | 4.15 | 10.14 | 11.70 | 13.33 | 0.00 | 1.10 | up |
| HORVU.MOREX.r2.3HG0245260 | 1695.00 | 1518.00 | 815.00 | 5500.00 | 3949.00 | 4840.00 | 13.28 | 11.81 | 6.65 | 42.57 | 29.80 | 39.47 | 0.00 | 1.68 | up |
| HORVU.MOREX.r2.3HG0240180 | 122.00 | 94.00 | 85.00 | 386.00 | 274.00 | 348.00 | 2.64 | 2.02 | 1.91 | 8.26 | 5.72 | 7.85 | 0.00 | 1.60 | up |
| HORVU.MOREX.r2.2HG0098350 | 644.00 | 736.00 | 572.00 | 1460.00 | 1306.00 | 1477.00 | 5.40 | 6.12 | 4.99 | 12.08 | 10.53 | 12.87 | 0.00 | 1.06 | up |
| HORVU.MOREX.r2.UnG0627890 | 296.00 | 260.00 | 369.00 | 3746.00 | 2224.00 | 2815.00 | 2.88 | 2.51 | 3.74 | 36.09 | 20.89 | 28.57 | 0.00 | 3.03 | up |
| HORVU.MOREX.r2.5HG0412430 | 138.00 | 109.00 | 9.00 | 5739.00 | 4097.00 | 4562.00 | 1.68 | 1.31 | 0.11 | 68.95 | 47.99 | 57.75 | 0.00 | 2.70 | up |
| HORVU.MOREX.r2.5HG0395660 | 39.00 | 31.00 | 31.00 | 260.00 | 170.00 | 280.00 | 0.32 | 0.25 | 0.26 | 2.12 | 1.35 | 2.41 | 0.00 | 2.51 | up |
| HORVU.MOREX.r2.2HG0087900 | 1262.00 | 1114.00 | 915.00 | 3235.00 | 2755.00 | 3349.00 | 12.83 | 11.24 | 9.68 | 32.47 | 26.96 | 35.43 | 0.00 | 1.42 | up |
| HORVU.MOREX.r2.7HG0558340 | 532.00 | 465.00 | 381.00 | 2489.00 | 2023.00 | 2519.00 | 6.50 | 5.64 | 4.84 | 30.03 | 23.79 | 32.02 | 0.00 | 2.24 | up |
| HORVU.MOREX.r2.3HG0201290 | 53.00 | 62.00 | 71.00 | 120.00 | 122.00 | 169.00 | 0.57 | 0.67 | 0.80 | 1.28 | 1.27 | 1.91 | 0.00 | 1.01 | up |
| HORVU.MOREX.r2.6HG0524500 | 47.00 | 22.00 | 5.00 | 409.00 | 181.00 | 236.00 | 0.68 | 0.32 | 0.08 | 5.91 | 2.55 | 3.58 | 0.00 | 2.55 | up |
| HORVU.MOREX.r2.2HG0114750 | 2080.00 | 2186.00 | 1598.00 | 6142.00 | 5937.00 | 7196.00 | 49.97 | 52.16 | 39.98 | 145.76 | 137.36 | 179.92 | 0.00 | 1.64 | up |
| HORVU.MOREX.r2.7HG0612100 | 943.00 | 888.00 | 995.00 | 3598.00 | 2058.00 | 3061.00 | 26.75 | 25.00 | 29.37 | 100.80 | 56.19 | 90.34 | 0.00 | 1.51 | up |
| HORVU.MOREX.r2.3HG0270060 | 65.00 | 86.00 | 122.00 | 805.00 | 561.00 | 918.00 | 4.72 | 6.21 | 9.20 | 57.73 | 39.26 | 69.41 | 0.00 | 2.78 | up |
| HORVU.MOREX.r2.3HG0192020 | 53.00 | 137.00 | 54.00 | 187.00 | 340.00 | 338.00 | 0.87 | 2.26 | 0.93 | 3.07 | 5.44 | 5.84 | 0.00 | 1.55 | up |
| HORVU.MOREX.r2.4HG0338350 | 78.00 | 88.00 | 71.00 | 316.00 | 142.00 | 193.00 | 1.15 | 1.30 | 1.09 | 4.63 | 2.02 | 2.97 | 0.00 | 1.27 | up |
| HORVU.MOREX.r2.7HG0581900 | 181.00 | 234.00 | 206.00 | 1278.00 | 798.00 | 911.00 | 2.11 | 2.71 | 2.50 | 14.73 | 8.96 | 11.06 | 0.00 | 2.12 | up |
| HORVU.MOREX.r2.5HG0413670 | 185.00 | 291.00 | 303.00 | 674.00 | 636.00 | 407.00 | 2.00 | 3.12 | 3.41 | 7.21 | 6.63 | 4.58 | 0.00 | 1.02 | up |
| HORVU.MOREX.r2.1HG0056620 | 1919.00 | 2224.00 | 1520.00 | 4975.00 | 4000.00 | 5842.00 | 11.57 | 13.32 | 9.55 | 29.64 | 23.23 | 36.67 | 0.00 | 1.31 | up |
| HORVU.MOREX.r2.7HG0534320 | 0.00 | 0.00 | 3.00 | 344.00 | 75.00 | 281.00 | 0.00 | 0.00 | 0.16 | 19.37 | 4.06 | 16.65 | 0.00 | 2.24 | up |
| HORVU.MOREX.r2.1HG0050960 | 20.00 | 9.00 | 0.00 | 70.00 | 81.00 | 165.00 | 0.55 | 0.23 | 0.00 | 1.99 | 2.24 | 4.94 | 0.00 | 2.05 | up |
| HORVU.MOREX.r2.5HG0363900 | 522.00 | 615.00 | 429.00 | 1423.00 | 1239.00 | 1906.00 | 6.07 | 7.10 | 5.19 | 16.34 | 13.87 | 23.06 | 0.00 | 1.45 | up |
| HORVU.MOREX.r2.6HG0448600 | 38.00 | 13.00 | 16.00 | 86.00 | 59.00 | 91.00 | 0.26 | 0.09 | 0.11 | 0.59 | 0.39 | 0.65 | 0.00 | 1.40 | up |
| HORVU.MOREX.r2.5HG0417580 | 5.00 | 0.00 | 0.00 | 114.00 | 109.00 | 146.00 | 0.10 | 0.00 | 0.00 | 2.63 | 2.46 | 3.54 | 0.00 | 4.26 | up |
| HORVU.MOREX.r2.2HG0170790 | 3.00 | 4.00 | 12.00 | 7614.00 | 4121.00 | 6120.00 | 0.02 | 0.04 | 0.11 | 68.97 | 36.39 | 58.41 | 0.00 | 7.15 | up |
| HORVU.MOREX.r2.7HG0529150 | 9.00 | 11.00 | 4.00 | 66.00 | 62.00 | 133.00 | 0.11 | 0.14 | 0.05 | 0.82 | 0.75 | 1.74 | 0.00 | 2.59 | up |
| HORVU.MOREX.r2.2HG0142270 | 327.00 | 125.00 | 136.00 | 1750.00 | 2355.00 | 4005.00 | 8.74 | 3.32 | 3.79 | 46.32 | 60.76 | 111.68 | 0.00 | 2.79 | up |
| HORVU.MOREX.r2.7HG0550180 | 100.00 | 92.00 | 78.00 | 420.00 | 322.00 | 387.00 | 2.74 | 2.49 | 2.22 | 11.42 | 8.53 | 11.09 | 0.00 | 1.93 | up |
| HORVU.MOREX.r2.2HG0088550 | 42.00 | 33.00 | 54.00 | 1179.00 | 752.00 | 822.00 | 0.49 | 0.38 | 0.67 | 13.87 | 8.62 | 10.18 | 0.00 | 4.06 | up |
| HORVU.MOREX.r2.7HG0562850 | 1188.00 | 1024.00 | 816.00 | 4849.00 | 4350.00 | 5666.00 | 10.81 | 9.25 | 7.73 | 43.58 | 38.11 | 53.65 | 0.00 | 2.18 | up |
| HORVU.MOREX.r2.1HG0067320 | 113.00 | 90.00 | 25.00 | 1038.00 | 891.00 | 1261.00 | 1.88 | 1.49 | 0.42 | 17.12 | 14.33 | 21.91 | 0.00 | 3.25 | up |
| HORVU.MOREX.r2.1HG0067490 | 8239.00 | 6998.00 | 5844.00 | 21923.00 | 16642.00 | 18039.00 | 119.79 | 101.02 | 88.46 | 314.77 | 232.95 | 272.90 | 0.00 | 1.34 | up |
| HORVU.MOREX.r2.5HG0379640 | 248.00 | 126.00 | 112.00 | 595.00 | 684.00 | 717.00 | 2.38 | 1.20 | 1.12 | 5.65 | 6.34 | 7.18 | 0.00 | 1.82 | up |
| HORVU.MOREX.r2.7HG0624010 | 29.00 | 28.00 | 29.00 | 214.00 | 94.00 | 181.00 | 0.22 | 0.22 | 0.24 | 1.66 | 0.71 | 1.48 | 0.00 | 2.13 | up |
| HORVU.MOREX.r2.2HG0157880 | 84.00 | 27.00 | 10.00 | 1271.00 | 694.00 | 1032.00 | 6.91 | 2.20 | 0.82 | 104.22 | 55.46 | 89.21 | 0.00 | 2.55 | up |
| HORVU.MOREX.r2.7HG0589200 | 13.00 | 8.00 | 12.00 | 79.00 | 57.00 | 116.00 | 0.96 | 0.63 | 0.92 | 6.16 | 4.35 | 9.59 | 0.00 | 2.31 | up |
| HORVU.MOREX.r2.7HG0545630 | 244.00 | 432.00 | 183.00 | 1307.00 | 816.00 | 1271.00 | 3.78 | 6.64 | 2.95 | 20.01 | 12.17 | 20.51 | 0.00 | 1.79 | up |
| HORVU.MOREX.r2.3HG0210840 | 24.00 | 11.00 | 6.00 | 514.00 | 433.00 | 628.00 | 1.27 | 0.59 | 0.33 | 27.58 | 22.66 | 35.49 | 0.00 | 4.56 | up |
| HORVU.MOREX.r2.3HG0200880 | 1345.00 | 1789.00 | 1393.00 | 3032.00 | 2881.00 | 3567.00 | 22.21 | 29.34 | 23.95 | 49.46 | 45.82 | 61.32 | 0.00 | 1.00 | up |
| Hordeum_vulgare_newGene_373 | 154.00 | 247.00 | 198.00 | 1378.00 | 1261.00 | 1672.00 | 4.60 | 7.35 | 6.20 | 40.93 | 36.52 | 52.33 | 0.00 | 2.69 | up |
| Hordeum_vulgare_newGene_370 | 8.00 | 47.00 | 38.00 | 286.00 | 216.00 | 428.00 | 0.17 | 0.96 | 0.81 | 5.85 | 4.31 | 9.25 | 0.00 | 2.62 | up |
| HORVU.MOREX.r2.2HG0088020 | 1080.00 | 784.00 | 750.00 | 2709.00 | 1622.00 | 1619.00 | 6.16 | 4.44 | 4.45 | 15.27 | 8.91 | 9.61 | 0.00 | 1.07 | up |
| HORVU.MOREX.r2.1HG0005040 | 63.00 | 82.00 | 86.00 | 243.00 | 297.00 | 354.00 | 1.70 | 2.20 | 2.43 | 6.52 | 7.75 | 10.00 | 0.00 | 1.79 | up |
| HORVU.MOREX.r2.2HG0155970 | 157.00 | 259.00 | 201.00 | 1397.00 | 913.00 | 1211.00 | 2.22 | 3.65 | 2.97 | 19.62 | 12.49 | 17.91 | 0.00 | 2.35 | up |
| HORVU.MOREX.r2.7HG0610930 | 0.00 | 9.00 | 2.00 | 511.00 | 238.00 | 279.00 | 0.00 | 0.50 | 0.12 | 30.22 | 13.73 | 17.35 | 0.00 | 2.76 | up |
| HORVU.MOREX.r2.3HG0270900 | 2.00 | 0.00 | 0.00 | 251.00 | 239.00 | 260.00 | 0.04 | 0.00 | 0.00 | 4.58 | 4.26 | 5.00 | 0.00 | 5.80 | up |
| HORVU.MOREX.r2.7HG0612270 | 1720.00 | 1198.00 | 1213.00 | 3951.00 | 3023.00 | 3534.00 | 9.97 | 6.90 | 7.32 | 22.62 | 16.87 | 21.32 | 0.00 | 1.25 | up |
| HORVU.MOREX.r2.4HG0282440 | 2223.00 | 2875.00 | 2320.00 | 5620.00 | 5612.00 | 5817.00 | 44.11 | 56.66 | 47.93 | 110.15 | 107.23 | 120.12 | 0.00 | 1.14 | up |
| HORVU.MOREX.r2.3HG0201860 | 4.00 | 7.00 | 13.00 | 185.00 | 215.00 | 238.00 | 0.10 | 0.15 | 0.31 | 4.42 | 5.00 | 5.98 | 0.00 | 4.01 | up |
| HORVU.MOREX.r2.1HG0061200 | 723.00 | 650.00 | 528.00 | 1433.00 | 1379.00 | 1313.00 | 9.02 | 8.05 | 6.85 | 17.65 | 16.56 | 17.05 | 0.00 | 1.05 | up |
| HORVU.MOREX.r2.6HG0520660 | 53.00 | 80.00 | 39.00 | 1557.00 | 516.00 | 703.00 | 0.27 | 0.42 | 0.21 | 8.09 | 2.61 | 3.85 | 0.00 | 3.42 | up |
| HORVU.MOREX.r2.3HG0238870 | 3866.00 | 5891.00 | 4167.00 | 16705.00 | 16449.00 | 19388.00 | 30.71 | 46.47 | 34.46 | 131.07 | 125.82 | 160.27 | 0.00 | 1.83 | up |
| HORVU.MOREX.r2.2HG0154720 | 42.00 | 32.00 | 27.00 | 610.00 | 257.00 | 348.00 | 0.47 | 0.35 | 0.32 | 6.88 | 2.83 | 4.13 | 0.00 | 3.11 | up |
| HORVU.MOREX.r2.5HG0438580 | 140.00 | 227.00 | 112.00 | 302.00 | 499.00 | 823.00 | 1.41 | 2.26 | 1.17 | 2.99 | 4.83 | 8.61 | 0.00 | 1.52 | up |
| HORVU.MOREX.r2.5HG0408180 | 24.00 | 30.00 | 29.00 | 119.00 | 88.00 | 94.00 | 0.12 | 0.16 | 0.16 | 0.63 | 0.46 | 0.52 | 0.00 | 1.63 | up |
| HORVU.MOREX.r2.2HG0081690 | 138.00 | 230.00 | 154.00 | 949.00 | 1179.00 | 1252.00 | 2.17 | 3.58 | 2.50 | 14.72 | 17.84 | 20.47 | 0.00 | 2.54 | up |
| HORVU.MOREX.r2.1HG0074930 | 767.00 | 460.00 | 260.00 | 2633.00 | 1717.00 | 2509.00 | 29.93 | 17.80 | 10.54 | 101.46 | 64.50 | 101.90 | 0.00 | 1.95 | up |
| HORVU.MOREX.r2.7HG0599230 | 1049.00 | 1701.00 | 1380.00 | 29193.00 | 19080.00 | 24248.00 | 4.23 | 6.81 | 5.79 | 116.27 | 74.09 | 101.76 | 0.00 | 3.92 | up |
| HORVU.MOREX.r2.2HG0081860 | 487.00 | 937.00 | 575.00 | 5997.00 | 3564.00 | 5169.00 | 4.75 | 9.08 | 5.84 | 57.78 | 33.48 | 52.48 | 0.00 | 2.67 | up |
| HORVU.MOREX.r2.3HG0244440 | 437.00 | 430.00 | 393.00 | 1264.00 | 631.00 | 1025.00 | 5.19 | 5.07 | 4.86 | 14.85 | 7.22 | 12.67 | 0.00 | 1.10 | up |
| HORVU.MOREX.r2.1HG0036100 | 2458.00 | 2024.00 | 1394.00 | 5222.00 | 3812.00 | 5048.00 | 63.26 | 51.71 | 37.33 | 132.70 | 94.44 | 135.15 | 0.00 | 1.17 | up |
| HORVU.MOREX.r2.5HG0368210 | 235.00 | 284.00 | 256.00 | 857.00 | 812.00 | 1159.00 | 3.85 | 4.61 | 4.36 | 13.86 | 12.80 | 19.76 | 0.00 | 1.76 | up |
| HORVU.MOREX.r2.7HG0529390 | 0.00 | 0.00 | 0.00 | 120.00 | 59.00 | 104.00 | 0.00 | 0.00 | 0.00 | 0.66 | 0.31 | 0.60 | 0.00 | 4.43 | up |
| HORVU.MOREX.r2.7HG0528440 | 45.00 | 63.00 | 50.00 | 123.00 | 100.00 | 135.00 | 2.22 | 3.07 | 2.56 | 6.01 | 4.74 | 6.98 | 0.00 | 1.05 | up |
| HORVU.MOREX.r2.3HG0241870 | 101.00 | 60.00 | 55.00 | 215.00 | 159.00 | 271.00 | 2.66 | 1.56 | 1.52 | 5.63 | 4.04 | 7.48 | 0.00 | 1.37 | up |
| HORVU.MOREX.r2.2HG0106210 | 90.00 | 85.00 | 93.00 | 279.00 | 178.00 | 201.00 | 1.36 | 1.28 | 1.47 | 4.21 | 2.62 | 3.20 | 0.00 | 1.17 | up |
| HORVU.MOREX.r2.7HG0605490 | 610.00 | 651.00 | 409.00 | 1172.00 | 1150.00 | 1621.00 | 6.24 | 6.62 | 4.35 | 11.85 | 11.33 | 17.27 | 0.00 | 1.15 | up |
| HORVU.MOREX.r2.2HG0114080 | 288.00 | 329.00 | 231.00 | 1554.00 | 1547.00 | 2098.00 | 2.39 | 2.71 | 1.99 | 12.77 | 12.39 | 18.16 | 0.00 | 2.48 | up |
| HORVU.MOREX.r2.3HG0190050 | 0.00 | 0.00 | 0.00 | 392.00 | 678.00 | 478.00 | 0.00 | 0.00 | 0.00 | 4.34 | 7.32 | 5.58 | 0.00 | 6.81 | up |
| HORVU.MOREX.r2.7HG0589510 | 674.00 | 583.00 | 586.00 | 1733.00 | 1278.00 | 1681.00 | 14.78 | 12.69 | 13.37 | 37.56 | 27.00 | 38.38 | 0.00 | 1.26 | up |
| Hordeum_vulgare_newGene_5514 | 858.00 | 437.00 | 220.00 | 0.00 | 0.00 | 2.00 | 10.11 | 5.25 | 3.25 | 0.00 | 0.00 | 0.01 | 0.00 | (3.89) | down |
| HORVU.MOREX.r2.2HG0155090 | 381.00 | 191.00 | 557.00 | 160.00 | 210.00 | 68.00 | 4.57 | 2.28 | 6.96 | 1.90 | 2.43 | 0.84 | 0.01 | (1.21) | down |
| HORVU.MOREX.r2.5HG0412720 | 6788.00 | 6860.00 | 4623.00 | 2729.00 | 3403.00 | 2105.00 | 174.67 | 175.27 | 123.85 | 69.34 | 84.29 | 56.35 | 0.00 | (1.16) | down |
| HORVU.MOREX.r2.3HG0182450 | 86.00 | 96.00 | 73.00 | 33.00 | 31.00 | 32.00 | 4.82 | 5.37 | 4.27 | 1.82 | 1.65 | 1.84 | 0.00 | (1.31) | down |
| HORVU.MOREX.r2.2HG0139710 | 373.00 | 385.00 | 393.00 | 167.00 | 177.00 | 143.00 | 8.85 | 9.06 | 9.69 | 3.90 | 4.03 | 3.53 | 0.00 | (1.26) | down |
| HORVU.MOREX.r2.2HG0141350 | 402.00 | 723.00 | 884.00 | 326.00 | 305.00 | 296.00 | 5.04 | 9.02 | 11.55 | 4.04 | 3.68 | 3.86 | 0.00 | (1.08) | down |
| HORVU.MOREX.r2.4HG0288340 | 412.00 | 578.00 | 518.00 | 220.00 | 170.00 | 132.00 | 19.95 | 27.78 | 26.10 | 10.51 | 7.92 | 6.65 | 0.00 | (1.50) | down |
| HORVU.MOREX.r2.2HG0095910 | 1841.00 | 1582.00 | 1242.00 | 741.00 | 743.00 | 637.00 | 157.44 | 134.32 | 110.52 | 62.58 | 61.15 | 56.66 | 0.00 | (1.15) | down |
| HORVU.MOREX.r2.5HG0442420 | 524.00 | 264.00 | 399.00 | 205.00 | 114.00 | 126.00 | 7.41 | 3.70 | 5.87 | 2.86 | 1.55 | 1.85 | 0.00 | (1.32) | down |
| HORVU.MOREX.r2.2HG0147750 | 378.00 | 393.00 | 390.00 | 166.00 | 222.00 | 135.00 | 5.20 | 5.36 | 5.59 | 2.26 | 2.94 | 1.94 | 0.00 | (1.16) | down |
| HORVU.MOREX.r2.6HG0463660 | 1742.00 | 2021.00 | 1412.00 | 790.00 | 792.00 | 510.00 | 110.08 | 126.85 | 92.92 | 49.28 | 48.15 | 33.49 | 0.00 | (1.31) | down |
| HORVU.MOREX.r2.3HG0204550 | 1991.00 | 2369.00 | 2058.00 | 955.00 | 1070.00 | 1001.00 | 112.41 | 132.81 | 120.94 | 53.23 | 58.12 | 58.81 | 0.00 | (1.12) | down |
| HORVU.MOREX.r2.2HG0137070 | 66.00 | 96.00 | 113.00 | 52.00 | 37.00 | 23.00 | 2.52 | 3.61 | 4.49 | 1.95 | 1.34 | 0.90 | 0.01 | (1.15) | down |
| HORVU.MOREX.r2.7HG0609900 | 196.00 | 268.00 | 148.00 | 53.00 | 37.00 | 44.00 | 7.48 | 10.18 | 5.86 | 1.98 | 1.36 | 1.75 | 0.00 | (2.03) | down |
| HORVU.MOREX.r2.2HG0170320 | 6473.00 | 4457.00 | 3276.00 | 2337.00 | 1701.00 | 1752.00 | 145.91 | 99.74 | 76.87 | 52.01 | 36.90 | 41.08 | 0.00 | (1.26) | down |
| HORVU.MOREX.r2.7HG0619330 | 447.00 | 506.00 | 382.00 | 158.00 | 100.00 | 69.00 | 8.14 | 9.15 | 7.24 | 2.83 | 1.75 | 1.30 | 0.00 | (1.91) | down |
| Hordeum_vulgare_newGene_5995 | 269.00 | 237.00 | 90.00 | 12.00 | 36.00 | 67.00 | 7.16 | 6.48 | 2.45 | 0.31 | 0.90 | 1.89 | 0.00 | (1.79) | down |
| HORVU.MOREX.r2.6HG0514060 | 92305.00 | 81768.00 | 82902.00 | 40846.00 | 47140.00 | 37461.00 | 894.75 | 786.95 | 836.62 | 390.99 | 439.91 | 377.82 | 0.00 | (1.07) | down |
| HORVU.MOREX.r2.5HG0356780 | 240.00 | 127.00 | 152.00 | 95.00 | 65.00 | 55.00 | 13.39 | 7.04 | 8.81 | 5.22 | 3.48 | 3.19 | 0.00 | (1.18) | down |
| HORVU.MOREX.r2.6HG0496040 | 140.00 | 113.00 | 102.00 | 53.00 | 54.00 | 44.00 | 3.41 | 2.71 | 2.58 | 1.27 | 1.26 | 1.11 | 0.00 | (1.18) | down |
| HORVU.MOREX.r2.2HG0142620 | 151.00 | 108.00 | 134.00 | 48.00 | 88.00 | 46.00 | 6.90 | 4.89 | 6.38 | 2.14 | 3.86 | 2.14 | 0.00 | (1.05) | down |
| HORVU.MOREX.r2.6HG0490630 | 326.00 | 266.00 | 311.00 | 119.00 | 170.00 | 122.00 | 5.51 | 4.48 | 5.47 | 1.98 | 2.76 | 2.15 | 0.00 | (1.13) | down |
| HORVU.MOREX.r2.7HG0540020 | 96.00 | 113.00 | 221.00 | 42.00 | 42.00 | 43.00 | 1.58 | 1.84 | 3.79 | 0.68 | 0.66 | 0.73 | 0.00 | (1.56) | down |
| HORVU.MOREX.r2.5HG0369270 | 167.00 | 155.00 | 176.00 | 76.00 | 49.00 | 30.00 | 9.22 | 8.49 | 10.13 | 4.12 | 2.60 | 1.68 | 0.00 | (1.55) | down |
| HORVU.MOREX.r2.4HG0285990 | 4187.00 | 3358.00 | 3466.00 | 2003.00 | 1816.00 | 1253.00 | 106.33 | 84.66 | 91.63 | 50.21 | 44.40 | 33.10 | 0.00 | (1.13) | down |
| HORVU.MOREX.r2.4HG0344750 | 618.00 | 569.00 | 881.00 | 275.00 | 308.00 | 134.00 | 10.85 | 9.92 | 16.12 | 4.76 | 5.20 | 2.45 | 0.00 | (1.46) | down |
| HORVU.MOREX.r2.7HG0558860 | 346.00 | 278.00 | 57.00 | 37.00 | 34.00 | 26.00 | 2.09 | 1.67 | 0.35 | 0.22 | 0.20 | 0.16 | 0.00 | (2.18) | down |
| Hordeum_vulgare_newGene_5817 | 516.00 | 306.00 | 573.00 | 144.00 | 82.00 | 66.00 | 1.78 | 0.91 | 1.78 | 0.42 | 0.24 | 0.20 | 0.00 | (2.06) | down |
| Hordeum_vulgare_newGene_7502 | 553.00 | 524.00 | 516.00 | 245.00 | 261.00 | 195.00 | 6.75 | 6.02 | 6.08 | 2.82 | 2.94 | 2.39 | 0.00 | (1.20) | down |
| Hordeum_vulgare_newGene_1554 | 292.00 | 272.00 | 180.00 | 84.00 | 112.00 | 117.00 | 5.17 | 4.79 | 3.31 | 1.46 | 1.90 | 2.14 | 0.00 | (1.20) | down |
| HORVU.MOREX.r2.2HG0086070 | 1645.00 | 915.00 | 2748.00 | 836.00 | 724.00 | 485.00 | 54.65 | 30.16 | 95.05 | 27.41 | 23.14 | 16.76 | 0.00 | (1.29) | down |
| HORVU.MOREX.r2.1HG0047140 | 1813.00 | 1188.00 | 1907.00 | 1000.00 | 827.00 | 594.00 | 25.72 | 16.72 | 28.15 | 14.01 | 11.28 | 8.76 | 0.00 | (1.02) | down |
| HORVU.MOREX.r2.5HG0399490 | 425.00 | 323.00 | 368.00 | 204.00 | 182.00 | 145.00 | 7.91 | 5.97 | 7.13 | 3.75 | 3.26 | 2.80 | 0.00 | (1.08) | down |
| HORVU.MOREX.r2.4HG0346590 | 213.00 | 166.00 | 298.00 | 102.00 | 102.00 | 60.00 | 6.04 | 4.65 | 8.77 | 2.83 | 2.77 | 1.77 | 0.00 | (1.28) | down |
| HORVU.MOREX.r2.5HG0412750 | 423.00 | 480.00 | 348.00 | 134.00 | 199.00 | 195.00 | 11.44 | 12.87 | 9.78 | 3.56 | 5.17 | 5.49 | 0.00 | (1.23) | down |
| HORVU.MOREX.r2.3HG0259580 | 695.00 | 717.00 | 415.00 | 217.00 | 219.00 | 196.00 | 35.43 | 36.28 | 22.00 | 10.91 | 10.71 | 10.40 | 0.00 | (1.50) | down |
| HORVU.MOREX.r2.3HG0229370 | 2145.00 | 4404.00 | 2779.00 | 1153.00 | 1355.00 | 989.00 | 40.36 | 82.29 | 54.44 | 21.43 | 24.55 | 19.36 | 0.00 | (1.38) | down |
| HORVU.MOREX.r2.2HG0111800 | 311.00 | 57.00 | 61.00 | 16.00 | 10.00 | 22.00 | 5.10 | 0.92 | 1.03 | 0.26 | 0.15 | 0.36 | 0.00 | (2.15) | down |
| HORVU.MOREX.r2.4HG0281730 | 446.00 | 298.00 | 209.00 | 110.00 | 175.00 | 143.00 | 5.35 | 3.54 | 2.60 | 1.30 | 2.01 | 1.77 | 0.00 | (1.10) | down |
| Hordeum_vulgare_newGene_1401 | 865.00 | 934.00 | 628.00 | 328.00 | 388.00 | 339.00 | 24.56 | 26.33 | 18.55 | 9.18 | 10.61 | 10.00 | 0.00 | (1.21) | down |
| Hordeum_vulgare_newGene_256 | 78.00 | 74.00 | 76.00 | 40.00 | 20.00 | 13.00 | 0.62 | 0.48 | 0.52 | 0.29 | 0.18 | 0.12 | 0.00 | (1.39) | down |
| HORVU.MOREX.r2.7HG0593650 | 817.00 | 586.00 | 692.00 | 213.00 | 297.00 | 249.00 | 19.46 | 13.87 | 17.15 | 5.00 | 6.81 | 6.18 | 0.00 | (1.46) | down |
| HORVU.MOREX.r2.2HG0085320 | 665.00 | 291.00 | 934.00 | 366.00 | 195.00 | 191.00 | 8.72 | 3.79 | 12.76 | 4.74 | 2.46 | 2.60 | 0.01 | (1.19) | down |
| HORVU.MOREX.r2.3HG0188660 | 1322.00 | 336.00 | 373.00 | 245.00 | 210.00 | 238.00 | 66.27 | 16.68 | 19.45 | 12.09 | 10.09 | 12.37 | 0.00 | (1.33) | down |
| HORVU.MOREX.r2.5HG0356890 | 555.00 | 316.00 | 379.00 | 123.00 | 115.00 | 80.00 | 31.03 | 17.52 | 22.03 | 6.77 | 6.16 | 4.64 | 0.00 | (1.88) | down |
| HORVU.MOREX.r2.2HG0128900 | 349.00 | 330.00 | 403.00 | 187.00 | 187.00 | 167.00 | 16.46 | 15.49 | 19.81 | 8.71 | 8.49 | 8.18 | 0.00 | (1.02) | down |
| HORVU.MOREX.r2.1HG0072060 | 632.00 | 390.00 | 305.00 | 118.00 | 140.00 | 71.00 | 6.96 | 4.26 | 3.49 | 1.28 | 1.48 | 0.80 | 0.00 | (1.87) | down |
| HORVU.MOREX.r2.1HG0062060 | 778.00 | 795.00 | 674.00 | 343.00 | 351.00 | 216.00 | 28.28 | 28.67 | 25.50 | 12.31 | 12.28 | 8.15 | 0.00 | (1.31) | down |
| HORVU.MOREX.r2.5HG0406570 | 207.00 | 198.00 | 160.00 | 108.00 | 93.00 | 68.00 | 7.05 | 6.70 | 5.68 | 3.62 | 3.03 | 2.41 | 0.00 | (1.05) | down |
| HORVU.MOREX.r2.7HG0571590 | 43836.00 | 16054.00 | 37632.00 | 12284.00 | 10290.00 | 3104.00 | 980.58 | 356.54 | 876.39 | 271.34 | 221.60 | 72.24 | 0.01 | (1.48) | down |
| HORVU.MOREX.r2.1HG0017400 | 511.00 | 693.00 | 618.00 | 345.00 | 333.00 | 191.00 | 6.10 | 8.21 | 7.67 | 4.06 | 3.82 | 2.36 | 0.00 | (1.06) | down |
| HORVU.MOREX.r2.3HG0254050 | 127.00 | 58.00 | 96.00 | 37.00 | 26.00 | 35.00 | 6.89 | 3.10 | 5.42 | 1.94 | 1.32 | 1.93 | 0.00 | (1.33) | down |
| HORVU.MOREX.r2.7HG0624030 | 1063.00 | 1762.00 | 1512.00 | 593.00 | 675.00 | 721.00 | 17.17 | 28.25 | 25.43 | 9.46 | 10.49 | 12.10 | 0.00 | (1.12) | down |
| HORVU.MOREX.r2.1HG0066490 | 58.00 | 122.00 | 83.00 | 17.00 | 31.00 | 33.00 | 0.59 | 1.24 | 0.88 | 0.16 | 0.30 | 0.35 | 0.00 | (1.45) | down |
| HORVU.MOREX.r2.5HG0388290 | ######## | 96207.00 | ######## | 71072.00 | 70496.00 | 46352.00 | 5284.75 | 3728.56 | 6626.55 | 2739.57 | 2649.15 | 1882.51 | 0.00 | (1.08) | down |
| HORVU.MOREX.r2.4HG0279160 | 510.00 | 648.00 | 683.00 | 322.00 | 328.00 | 279.00 | 8.68 | 10.97 | 12.11 | 5.42 | 5.38 | 4.94 | 0.00 | (1.01) | down |
| HORVU.MOREX.r2.7HG0612980 | 728.00 | 649.00 | 472.00 | 257.00 | 362.00 | 302.00 | 10.13 | 8.96 | 6.83 | 3.53 | 4.85 | 4.37 | 0.00 | (1.01) | down |
| HORVU.MOREX.r2.2HG0111750 | 475.00 | 372.00 | 1042.00 | 275.00 | 150.00 | 216.00 | 16.43 | 12.75 | 37.54 | 9.39 | 4.98 | 7.75 | 0.00 | (1.40) | down |
| HORVU.MOREX.r2.1HG0071820 | 288.00 | 322.00 | 391.00 | 153.00 | 169.00 | 100.00 | 22.03 | 24.40 | 31.14 | 11.56 | 12.44 | 7.95 | 0.00 | (1.23) | down |
| HORVU.MOREX.r2.6HG0490720 | 188.00 | 185.00 | 219.00 | 108.00 | 102.00 | 85.00 | 1.56 | 1.52 | 1.89 | 0.88 | 0.81 | 0.73 | 0.00 | (1.00) | down |
| HORVU.MOREX.r2.2HG0176050 | 3864.00 | 2731.00 | 3738.00 | 2037.00 | 2093.00 | 961.00 | 45.58 | 31.99 | 45.91 | 23.72 | 23.77 | 11.79 | 0.00 | (1.01) | down |
| HORVU.MOREX.r2.2HG0083580 | 1379.00 | 2182.00 | 2746.00 | 748.00 | 1010.00 | 722.00 | 15.01 | 23.59 | 31.13 | 8.04 | 10.59 | 8.18 | 0.00 | (1.32) | down |
| HORVU.MOREX.r2.2HG0162190 | 153.00 | 537.00 | 440.00 | 98.00 | 180.00 | 131.00 | 0.75 | 2.62 | 2.25 | 0.48 | 0.85 | 0.67 | 0.00 | (1.29) | down |
| HORVU.MOREX.r2.1HG0002070 | 365.00 | 385.00 | 364.00 | 178.00 | 216.00 | 80.00 | 25.25 | 26.45 | 26.21 | 12.16 | 14.34 | 5.70 | 0.00 | (1.18) | down |
| HORVU.MOREX.r2.7HG0551540 | 230.00 | 63.00 | 103.00 | 0.00 | 0.00 | 0.00 | 6.89 | 1.86 | 3.19 | 0.00 | 0.00 | 0.00 | 0.00 | (4.49) | down |
| HORVU.MOREX.r2.1HG0029020 | 24281.00 | 13220.00 | 39413.00 | 12321.00 | 11904.00 | 9417.00 | 797.84 | 431.28 | 1348.26 | 399.80 | 376.57 | 321.95 | 0.00 | (1.14) | down |
| HORVU.MOREX.r2.4HG0281700 | 138.00 | 86.00 | 181.00 | 52.00 | 31.00 | 26.00 | 3.49 | 2.15 | 4.78 | 1.29 | 0.75 | 0.67 | 0.00 | (1.65) | down |
| HORVU.MOREX.r2.5HG0413010 | 224.00 | 244.00 | 392.00 | 170.00 | 95.00 | 109.00 | 4.32 | 4.68 | 7.87 | 3.23 | 1.76 | 2.19 | 0.00 | (1.13) | down |
| HORVU.MOREX.r2.7HG0622630 | 63.00 | 58.00 | 87.00 | 24.00 | 20.00 | 27.00 | 0.48 | 0.44 | 0.69 | 0.18 | 0.14 | 0.21 | 0.00 | (1.37) | down |
| HORVU.MOREX.r2.5HG0369380 | 1042.00 | 183.00 | 488.00 | 171.00 | 175.00 | 113.00 | 58.26 | 10.15 | 28.41 | 9.41 | 9.38 | 6.55 | 0.00 | (1.59) | down |
| HORVU.MOREX.r2.3HG0226640 | 407.00 | 510.00 | 435.00 | 192.00 | 175.00 | 215.00 | 7.41 | 9.23 | 8.25 | 3.44 | 3.06 | 4.07 | 0.00 | (1.21) | down |
| HORVU.MOREX.r2.3HG0254020 | 256.00 | 124.00 | 123.00 | 57.00 | 55.00 | 61.00 | 2.62 | 1.25 | 1.30 | 0.57 | 0.54 | 0.64 | 0.00 | (1.39) | down |
| Hordeum_vulgare_newGene_2624 | 101.00 | 112.00 | 82.00 | 24.00 | 34.00 | 41.00 | 2.92 | 3.22 | 2.46 | 0.68 | 0.95 | 1.22 | 0.00 | (1.44) | down |
| HORVU.MOREX.r2.5HG0424850 | 279.00 | 313.00 | 622.00 | 81.00 | 119.00 | 66.00 | 2.51 | 2.80 | 5.83 | 0.72 | 1.03 | 0.62 | 0.00 | (1.99) | down |
| HORVU.MOREX.r2.3HG0245410 | 135.00 | 98.00 | 106.00 | 32.00 | 38.00 | 12.00 | 4.46 | 3.21 | 3.64 | 1.03 | 1.21 | 0.41 | 0.00 | (1.78) | down |
| HORVU.MOREX.r2.3HG0270090 | 3725.00 | 2875.00 | 3765.00 | 1827.00 | 1662.00 | 1217.00 | 138.86 | 106.39 | 146.10 | 67.25 | 59.64 | 47.19 | 0.00 | (1.16) | down |
| HORVU.MOREX.r2.5HG0415670 | 5105.00 | 2719.00 | 4834.00 | 1082.00 | 2017.00 | 2288.00 | 101.67 | 53.75 | 100.23 | 21.26 | 38.67 | 47.41 | 0.00 | (1.17) | down |
| HORVU.MOREX.r2.6HG0448480 | 182.00 | 143.00 | 125.00 | 86.00 | 72.00 | 57.00 | 15.38 | 11.97 | 11.01 | 7.13 | 5.85 | 4.94 | 0.00 | (1.03) | down |
| HORVU.MOREX.r2.3HG0242690 | 2962.00 | 892.00 | 199.00 | 99.00 | 130.00 | 158.00 | 48.38 | 14.46 | 3.37 | 1.59 | 2.04 | 2.67 | 0.01 | (1.67) | down |
| HORVU.MOREX.r2.5HG0389370 | 125.00 | 110.00 | 89.00 | 40.00 | 56.00 | 25.00 | 4.03 | 3.53 | 3.00 | 1.26 | 1.73 | 0.81 | 0.00 | (1.31) | down |
| HORVU.MOREX.r2.5HG0407340 | 2286.00 | 1796.00 | 3235.00 | 1126.00 | 1107.00 | 867.00 | 20.73 | 16.18 | 30.56 | 10.09 | 9.66 | 8.18 | 0.00 | (1.23) | down |
| Hordeum_vulgare_newGene_5316 | 216.00 | 237.00 | 327.00 | 129.00 | 159.00 | 77.00 | 2.10 | 2.26 | 3.31 | 1.24 | 1.48 | 0.77 | 0.00 | (1.06) | down |
| HORVU.MOREX.r2.4HG0344630 | 585.00 | 237.00 | 291.00 | 63.00 | 86.00 | 46.00 | 18.68 | 7.51 | 9.67 | 1.98 | 2.62 | 1.51 | 0.00 | (2.24) | down |
| HORVU.MOREX.r2.7HG0527870 | 301.00 | 97.00 | 374.00 | 57.00 | 76.00 | 120.00 | 2.32 | 0.74 | 2.99 | 0.43 | 0.56 | 0.95 | 0.01 | (1.34) | down |
| HORVU.MOREX.r2.7HG0604600 | 1015.00 | 1121.00 | 1431.00 | 700.00 | 655.00 | 437.00 | 45.39 | 49.76 | 66.61 | 30.90 | 28.18 | 20.33 | 0.00 | (1.00) | down |
| HORVU.MOREX.r2.2HG0114030 | 76.00 | 96.00 | 70.00 | 26.00 | 35.00 | 42.00 | 3.46 | 4.32 | 3.32 | 1.14 | 1.51 | 1.99 | 0.00 | (1.13) | down |
| HORVU.MOREX.r2.6HG0456470 | 32340.00 | 19906.00 | 28494.00 | 16212.00 | 14741.00 | 8220.00 | 345.12 | 210.91 | 316.56 | 170.84 | 151.44 | 91.26 | 0.00 | (1.04) | down |
| HORVU.MOREX.r2.5HG0367090 | 1931.00 | 1946.00 | 2247.00 | 920.00 | 1100.00 | 891.00 | 39.96 | 39.99 | 48.41 | 18.79 | 21.92 | 19.18 | 0.00 | (1.11) | down |
| HORVU.MOREX.r2.6HG0468320 | 168.00 | 135.00 | 123.00 | 29.00 | 97.00 | 41.00 | 1.87 | 1.49 | 1.42 | 0.32 | 1.04 | 0.47 | 0.01 | (1.19) | down |
| Hordeum_vulgare_newGene_7134 | 140.00 | 112.00 | 139.00 | 46.00 | 41.00 | 70.00 | 1.38 | 1.09 | 1.43 | 0.45 | 0.38 | 0.72 | 0.00 | (1.22) | down |
| Hordeum_vulgare_newGene_5202 | 60.00 | 108.00 | 67.00 | 32.00 | 18.00 | 15.00 | 1.95 | 3.52 | 2.28 | 1.03 | 0.56 | 0.50 | 0.00 | (1.57) | down |
| HORVU.MOREX.r2.4HG0339040 | 1838.00 | 858.00 | 531.00 | 270.00 | 336.00 | 228.00 | 95.42 | 44.19 | 28.67 | 13.81 | 16.76 | 12.28 | 0.00 | (1.76) | down |
| HORVU.MOREX.r2.7HG0552740 | 703.00 | 299.00 | 392.00 | 0.00 | 0.00 | 0.00 | 37.49 | 15.80 | 21.74 | 0.00 | 0.00 | 0.00 | 0.00 | (6.43) | down |
| HORVU.MOREX.r2.6HG0498130 | 488.00 | 465.00 | 348.00 | 234.00 | 210.00 | 87.00 | 7.32 | 6.93 | 5.44 | 3.47 | 3.03 | 1.35 | 0.00 | (1.22) | down |
| HORVU.MOREX.r2.2HG0162990 | 534.00 | 634.00 | 427.00 | 351.00 | 221.00 | 189.00 | 31.98 | 37.70 | 26.61 | 20.76 | 12.70 | 11.73 | 0.00 | (1.04) | down |
| HORVU.MOREX.r2.6HG0519100 | 6918.00 | 4715.00 | 3685.00 | 2392.00 | 2216.00 | 1652.00 | 140.18 | 94.85 | 77.73 | 47.86 | 43.22 | 34.82 | 0.00 | (1.27) | down |
| Hordeum_vulgare_newGene_2928 | 1083.00 | 870.00 | 1323.00 | 625.00 | 489.00 | 419.00 | 15.17 | 12.09 | 19.28 | 8.64 | 6.58 | 6.10 | 0.00 | (1.10) | down |
| HORVU.MOREX.r2.6HG0463670 | 140.00 | 143.00 | 112.00 | 66.00 | 65.00 | 46.00 | 1.48 | 1.50 | 1.23 | 0.68 | 0.66 | 0.50 | 0.00 | (1.12) | down |
| HORVU.MOREX.r2.1HG0045880 | 75.00 | 101.00 | 69.00 | 43.00 | 20.00 | 16.00 | 1.33 | 1.79 | 1.28 | 0.76 | 0.34 | 0.30 | 0.00 | (1.39) | down |
| Hordeum_vulgare_newGene_7040 | 448.00 | 860.00 | 615.00 | 280.00 | 410.00 | 256.00 | 3.44 | 6.57 | 4.91 | 2.13 | 3.02 | 2.04 | 0.00 | (1.00) | down |
| HORVU.MOREX.r2.7HG0621050 | 140.00 | 116.00 | 69.00 | 34.00 | 18.00 | 42.00 | 0.96 | 0.79 | 0.49 | 0.23 | 0.12 | 0.30 | 0.00 | (1.52) | down |
| HORVU.MOREX.r2.6HG0523580 | 520.00 | 600.00 | 371.00 | 143.00 | 155.00 | 53.00 | 6.01 | 6.88 | 4.46 | 1.63 | 1.72 | 0.63 | 0.00 | (1.91) | down |
| HORVU.MOREX.r2.2HG0160340 | 2482.00 | 1361.00 | 3506.00 | 1479.00 | 1203.00 | 711.00 | 41.24 | 22.45 | 60.65 | 24.26 | 19.24 | 12.28 | 0.00 | (1.06) | down |
| HORVU.MOREX.r2.3HG0188690 | 726.00 | 135.00 | 201.00 | 127.00 | 106.00 | 70.00 | 36.39 | 6.71 | 10.44 | 6.26 | 5.07 | 3.64 | 0.00 | (1.45) | down |
| Hordeum_vulgare_newGene_5618 | 87.00 | 180.00 | 127.00 | 62.00 | 67.00 | 25.00 | 0.94 | 1.92 | 1.49 | 0.82 | 0.85 | 0.22 | 0.01 | (1.19) | down |
| Hordeum_vulgare_newGene_2919 | 276.00 | 308.00 | 138.00 | 102.00 | 72.00 | 63.00 | 6.17 | 6.92 | 3.43 | 2.36 | 1.66 | 1.60 | 0.00 | (1.47) | down |
| HORVU.MOREX.r2.3HG0201670 | 7926.00 | 4831.00 | 8499.00 | 4062.00 | 4029.00 | 2217.00 | 150.16 | 90.86 | 167.62 | 75.98 | 73.47 | 43.70 | 0.00 | (1.04) | down |
| HORVU.MOREX.r2.6HG0497430 | 1492.00 | 539.00 | 729.00 | 483.00 | 395.00 | 270.00 | 24.51 | 8.79 | 12.47 | 7.82 | 6.24 | 4.61 | 0.00 | (1.17) | down |
| HORVU.MOREX.r2.4HG0342420 | 215.00 | 170.00 | 229.00 | 78.00 | 91.00 | 69.00 | 3.94 | 3.10 | 4.37 | 1.41 | 1.60 | 1.32 | 0.00 | (1.34) | down |
| Hordeum_vulgare_newGene_4628 | 974.00 | 471.00 | 398.00 | 0.00 | 0.00 | 0.00 | 10.60 | 4.97 | 4.45 | 0.00 | 0.00 | 0.00 | 0.00 | (6.71) | down |
| HORVU.MOREX.r2.3HG0228770 | 670.00 | 234.00 | 528.00 | 249.00 | 107.00 | 161.00 | 10.55 | 3.65 | 8.66 | 3.87 | 1.61 | 2.62 | 0.00 | (1.30) | down |
| HORVU.MOREX.r2.3HG0182120 | 62.00 | 68.00 | 78.00 | 25.00 | 18.00 | 13.00 | 2.54 | 2.78 | 3.33 | 1.02 | 0.70 | 0.54 | 0.00 | (1.66) | down |
| HORVU.MOREX.r2.6HG0510160 | 318.00 | 177.00 | 221.00 | 93.00 | 104.00 | 36.00 | 6.23 | 3.44 | 4.51 | 1.80 | 1.97 | 0.73 | 0.00 | (1.46) | down |
| HORVU.MOREX.r2.7HG0552960 | 4067.00 | 2526.00 | 2564.00 | 1393.00 | 1424.00 | 631.00 | 59.13 | 36.46 | 38.80 | 19.99 | 19.93 | 9.53 | 0.00 | (1.35) | down |
| HORVU.MOREX.r2.4HG0321190 | 165.00 | 104.00 | 189.00 | 66.00 | 53.00 | 66.00 | 3.02 | 1.89 | 3.61 | 1.19 | 0.93 | 1.25 | 0.00 | (1.22) | down |
| Hordeum_vulgare_newGene_6008 | 147.00 | 202.00 | 149.00 | 58.00 | 58.00 | 58.00 | 2.52 | 3.45 | 2.66 | 0.98 | 0.95 | 1.03 | 0.00 | (1.46) | down |
| HORVU.MOREX.r2.5HG0442310 | 5306.00 | 13483.00 | 8771.00 | 4137.00 | 3598.00 | 2119.00 | 107.52 | 271.27 | 185.04 | 82.79 | 70.18 | 44.68 | 0.00 | (1.40) | down |
| Hordeum_vulgare_newGene_5532 | 682.00 | 223.00 | 305.00 | 0.00 | 0.00 | 0.00 | 5.89 | 1.88 | 2.61 | 0.00 | 0.00 | 0.00 | 0.00 | (5.99) | down |
| HORVU.MOREX.r2.2HG0108480 | 17218.00 | 9827.00 | 24512.00 | 9219.00 | 8162.00 | 7661.00 | 391.16 | 221.65 | 579.75 | 206.81 | 178.50 | 181.09 | 0.00 | (1.02) | down |
| Hordeum_vulgare_newGene_6322 | 116.00 | 105.00 | 84.00 | 0.00 | 0.00 | 0.00 | 5.35 | 4.78 | 4.01 | 0.00 | 0.00 | 0.00 | 0.00 | (4.85) | down |
| HORVU.MOREX.r2.5HG0369340 | 659.00 | 476.00 | 411.00 | 245.00 | 293.00 | 80.00 | 36.84 | 26.40 | 23.91 | 13.49 | 15.77 | 4.63 | 0.00 | (1.20) | down |
| HORVU.MOREX.r2.5HG0408750 | 247.00 | 110.00 | 302.00 | 58.00 | 79.00 | 68.00 | 3.00 | 1.32 | 3.82 | 0.69 | 0.92 | 0.86 | 0.00 | (1.51) | down |
| HORVU.MOREX.r2.6HG0487960 | 81.00 | 106.00 | 117.00 | 53.00 | 51.00 | 31.00 | 2.88 | 3.77 | 4.35 | 1.86 | 1.75 | 1.15 | 0.00 | (1.10) | down |
| HORVU.MOREX.r2.7HG0558570 | 243.00 | 91.00 | 80.00 | 6.00 | 18.00 | 14.00 | 8.01 | 2.96 | 2.75 | 0.17 | 0.57 | 0.46 | 0.00 | (2.63) | down |
| HORVU.MOREX.r2.6HG0470440 | 2860.00 | 1408.00 | 1589.00 | 913.00 | 863.00 | 650.00 | 77.01 | 37.62 | 44.52 | 24.26 | 22.37 | 18.20 | 0.00 | (1.24) | down |
| HORVU.MOREX.r2.UnG0627260 | 161.00 | 220.00 | 168.00 | 84.00 | 72.00 | 44.00 | 3.90 | 5.29 | 4.24 | 2.01 | 1.66 | 1.10 | 0.00 | (1.38) | down |
| HORVU.MOREX.r2.2HG0176870 | 79.00 | 85.00 | 85.00 | 25.00 | 17.00 | 23.00 | 0.53 | 0.56 | 0.59 | 0.16 | 0.10 | 0.16 | 0.00 | (1.75) | down |
| HORVU.MOREX.r2.7HG0551860 | 960.00 | 1282.00 | 315.00 | 104.00 | 98.00 | 77.00 | 70.61 | 93.65 | 24.08 | 7.52 | 6.88 | 5.88 | 0.00 | (2.78) | down |
| HORVU.MOREX.r2.5HG0429840 | 164.00 | 201.00 | 200.00 | 123.00 | 64.00 | 67.00 | 1.31 | 1.59 | 1.66 | 0.97 | 0.49 | 0.55 | 0.00 | (1.09) | down |
| HORVU.MOREX.r2.7HG0538500 | 184.00 | 188.00 | 328.00 | 97.00 | 119.00 | 50.00 | 1.71 | 1.73 | 3.17 | 0.89 | 1.06 | 0.48 | 0.00 | (1.28) | down |
| HORVU.MOREX.r2.7HG0612080 | 154.00 | 155.00 | 206.00 | 72.00 | 93.00 | 88.00 | 5.15 | 5.15 | 7.21 | 2.37 | 3.00 | 3.06 | 0.00 | (1.01) | down |
| HORVU.MOREX.r2.5HG0351680 | 347.00 | 197.00 | 356.00 | 143.00 | 187.00 | 57.00 | 3.61 | 2.03 | 3.85 | 1.47 | 1.87 | 0.61 | 0.01 | (1.11) | down |
| Hordeum_vulgare_newGene_5239 | 118.00 | 281.00 | 250.00 | 75.00 | 65.00 | 34.00 | 0.54 | 1.27 | 1.51 | 0.33 | 0.29 | 0.16 | 0.00 | (1.68) | down |
| HORVU.MOREX.r2.6HG0505200 | 105.00 | 57.00 | 219.00 | 41.00 | 48.00 | 17.00 | 1.22 | 0.65 | 2.66 | 0.47 | 0.53 | 0.20 | 0.00 | (1.46) | down |
| HORVU.MOREX.r2.7HG0587330 | 88.00 | 125.00 | 184.00 | 40.00 | 28.00 | 8.00 | 1.11 | 1.57 | 2.43 | 0.50 | 0.34 | 0.09 | 0.00 | (1.91) | down |
| HORVU.MOREX.r2.7HG0542500 | 423.00 | 513.00 | 633.00 | 207.00 | 278.00 | 103.00 | 7.67 | 9.24 | 11.98 | 3.70 | 4.86 | 1.95 | 0.00 | (1.34) | down |
| HORVU.MOREX.r2.7HG0620060 | 191.00 | 192.00 | 161.00 | 45.00 | 111.00 | 49.00 | 3.00 | 2.99 | 2.62 | 0.69 | 1.67 | 0.80 | 0.00 | (1.30) | down |
| HORVU.MOREX.r2.7HG0613110 | 4471.00 | 5319.00 | 4429.00 | 2180.00 | 2630.00 | 1328.00 | 56.89 | 67.21 | 58.67 | 27.39 | 32.21 | 17.58 | 0.00 | (1.21) | down |
| HORVU.MOREX.r2.4HG0282950 | 83.00 | 88.00 | 64.00 | 37.00 | 14.00 | 19.00 | 0.99 | 1.05 | 0.79 | 0.43 | 0.16 | 0.24 | 0.00 | (1.48) | down |
| HORVU.MOREX.r2.7HG0552950 | 270.00 | 97.00 | 149.00 | 26.00 | 10.00 | 20.00 | 10.96 | 3.90 | 6.28 | 1.03 | 0.35 | 0.84 | 0.00 | (2.59) | down |
| HORVU.MOREX.r2.1HG0073500 | 3870.00 | 2688.00 | 6008.00 | 1127.00 | 1268.00 | 907.00 | 73.31 | 50.54 | 118.49 | 21.07 | 23.11 | 17.87 | 0.00 | (1.85) | down |
| HORVU.MOREX.r2.4HG0343680 | 1185.00 | 893.00 | 1047.00 | 357.00 | 337.00 | 295.00 | 32.35 | 24.20 | 29.74 | 9.61 | 8.86 | 8.36 | 0.00 | (1.66) | down |
| HORVU.MOREX.r2.2HG0143470 | 88.00 | 142.00 | 140.00 | 58.00 | 34.00 | 56.00 | 0.76 | 1.22 | 1.26 | 0.49 | 0.28 | 0.50 | 0.00 | (1.20) | down |
| HORVU.MOREX.r2.5HG0438260 | 1677.00 | 1525.00 | 1611.00 | 833.00 | 869.00 | 612.00 | 39.79 | 35.94 | 39.80 | 19.51 | 19.84 | 15.11 | 0.00 | (1.09) | down |
| HORVU.MOREX.r2.1HG0021770 | 314.00 | 102.00 | 268.00 | 84.00 | 76.00 | 91.00 | 14.96 | 4.80 | 13.30 | 3.92 | 3.48 | 4.49 | 0.00 | (1.28) | down |
| HORVU.MOREX.r2.4HG0346340 | 2968.00 | 5629.00 | 2648.00 | 1577.00 | 618.00 | 448.00 | 123.29 | 232.15 | 114.50 | 64.66 | 24.71 | 19.34 | 0.00 | (1.55) | down |
| HORVU.MOREX.r2.5HG0412740 | 2582.00 | 2596.00 | 1584.00 | 912.00 | 965.00 | 674.00 | 64.16 | 64.06 | 40.98 | 22.38 | 23.08 | 17.41 | 0.00 | (1.39) | down |
| HORVU.MOREX.r2.7HG0557850 | 1060.00 | 579.00 | 546.00 | 340.00 | 376.00 | 321.00 | 59.27 | 32.14 | 31.78 | 18.74 | 20.23 | 18.65 | 0.00 | (1.05) | down |
| HORVU.MOREX.r2.2HG0154910 | 1831.00 | 2386.00 | 2239.00 | 972.00 | 1042.00 | 652.00 | 19.25 | 24.91 | 24.51 | 10.09 | 10.55 | 7.13 | 0.00 | (1.29) | down |
| HORVU.MOREX.r2.3HG0188670 | 450.00 | 99.00 | 184.00 | 80.00 | 36.00 | 34.00 | 22.56 | 4.89 | 9.57 | 3.92 | 1.73 | 1.73 | 0.00 | (1.80) | down |
| HORVU.MOREX.r2.7HG0542510 | 132.00 | 203.00 | 243.00 | 93.00 | 75.00 | 32.00 | 6.13 | 9.35 | 11.74 | 4.25 | 3.35 | 1.51 | 0.00 | (1.36) | down |
| HORVU.MOREX.r2.3HG0260260 | 16722.00 | 18045.00 | 17325.00 | 8935.00 | 8246.00 | 6340.00 | 926.22 | 992.36 | 999.05 | 488.73 | 439.73 | 365.35 | 0.00 | (1.18) | down |
| HORVU.MOREX.r2.7HG0552940 | 149.00 | 67.00 | 110.00 | 23.00 | 21.00 | 6.00 | 3.63 | 1.62 | 2.78 | 0.55 | 0.48 | 0.14 | 0.00 | (2.16) | down |
| HORVU.MOREX.r2.5HG0356880 | 1344.00 | 1114.00 | 1249.00 | 685.00 | 594.00 | 331.00 | 75.14 | 61.83 | 72.71 | 37.83 | 31.95 | 19.21 | 0.00 | (1.19) | down |
| HORVU.MOREX.r2.7HG0560430 | 34171.00 | 16723.00 | 44122.00 | 15030.00 | 14487.00 | 15350.00 | 801.36 | 389.37 | 1077.24 | 348.06 | 327.06 | 374.54 | 0.00 | (1.05) | down |
| HORVU.MOREX.r2.2HG0167700 | 223.00 | 318.00 | 313.00 | 121.00 | 127.00 | 76.00 | 1.91 | 2.71 | 2.80 | 1.02 | 1.05 | 0.68 | 0.00 | (1.36) | down |
| HORVU.MOREX.r2.5HG0369230 | 627.00 | 353.00 | 624.00 | 206.00 | 255.00 | 68.00 | 34.67 | 19.36 | 35.96 | 11.26 | 13.56 | 3.91 | 0.00 | (1.43) | down |
| HORVU.MOREX.r2.2HG0123440 | 135.00 | 105.00 | 130.00 | 47.00 | 79.00 | 33.00 | 2.44 | 1.87 | 2.45 | 0.82 | 1.37 | 0.61 | 0.00 | (1.13) | down |
| HORVU.MOREX.r2.7HG0563450 | 141.00 | 115.00 | 84.00 | 0.00 | 0.00 | 0.00 | 1.13 | 0.91 | 0.70 | 0.00 | 0.00 | 0.00 | 0.00 | (4.92) | down |
| HORVU.MOREX.r2.1HG0042230 | 265.00 | 307.00 | 395.00 | 116.00 | 172.00 | 161.00 | 2.62 | 3.02 | 4.08 | 1.13 | 1.64 | 1.65 | 0.00 | (1.09) | down |
| HORVU.MOREX.r2.4HG0276830 | 404.00 | 132.00 | 123.00 | 50.00 | 45.00 | 51.00 | 4.43 | 1.43 | 1.40 | 0.53 | 0.47 | 0.57 | 0.00 | (1.81) | down |
| HORVU.MOREX.r2.4HG0331980 | 210.00 | 80.00 | 226.00 | 64.00 | 70.00 | 41.00 | 5.28 | 1.99 | 5.92 | 1.59 | 1.69 | 1.06 | 0.00 | (1.37) | down |
| HORVU.MOREX.r2.4HG0343140 | 722.00 | 573.00 | 1071.00 | 378.00 | 414.00 | 236.00 | 7.44 | 5.87 | 11.49 | 3.84 | 4.10 | 2.53 | 0.00 | (1.17) | down |
| HORVU.MOREX.r2.6HG0454170 | 9370.00 | 7337.00 | 8670.00 | 5395.00 | 4437.00 | 2569.00 | 168.71 | 131.17 | 162.52 | 95.92 | 76.91 | 48.13 | 0.00 | (1.03) | down |
| HORVU.MOREX.r2.3HG0227720 | ######## | ######## | ######## | 76282.00 | 71804.00 | 42175.00 | 3435.65 | 2600.48 | 4225.87 | 1691.57 | 1552.31 | 985.40 | 0.00 | (1.24) | down |
| HORVU.MOREX.r2.7HG0533270 | 930.00 | 301.00 | 991.00 | 286.00 | 216.00 | 121.00 | 10.90 | 3.50 | 12.09 | 3.31 | 2.43 | 1.47 | 0.00 | (1.61) | down |
| Hordeum_vulgare_newGene_6078 | 145.00 | 91.00 | 147.00 | 60.00 | 62.00 | 45.00 | 4.27 | 2.61 | 4.53 | 1.70 | 1.72 | 1.33 | 0.00 | (1.13) | down |
| HORVU.MOREX.r2.1HG0071810 | 900.00 | 722.00 | 949.00 | 351.00 | 417.00 | 272.00 | 17.80 | 14.18 | 19.53 | 6.84 | 7.93 | 5.58 | 0.00 | (1.31) | down |
| HORVU.MOREX.r2.2HG0096100 | 162.00 | 111.00 | 88.00 | 39.00 | 69.00 | 48.00 | 3.88 | 2.62 | 2.17 | 0.92 | 1.59 | 1.18 | 0.00 | (1.11) | down |
| HORVU.MOREX.r2.2HG0082500 | 803.00 | 518.00 | 880.00 | 325.00 | 233.00 | 170.00 | 22.55 | 14.44 | 25.73 | 9.00 | 6.29 | 4.95 | 0.00 | (1.53) | down |
| HORVU.MOREX.r2.5HG0356760 | 336.00 | 307.00 | 241.00 | 145.00 | 133.00 | 70.00 | 18.75 | 17.01 | 13.99 | 7.99 | 7.14 | 4.04 | 0.00 | (1.29) | down |
| HORVU.MOREX.r2.5HG0402040 | 144.00 | 68.00 | 277.00 | 55.00 | 48.00 | 25.00 | 2.37 | 1.10 | 4.74 | 0.89 | 0.76 | 0.42 | 0.00 | (1.57) | down |
| HORVU.MOREX.r2.7HG0559210 | 105.00 | 92.00 | 151.00 | 46.00 | 65.00 | 53.00 | 1.91 | 1.66 | 2.86 | 0.82 | 1.13 | 0.99 | 0.01 | (1.03) | down |
| HORVU.MOREX.r2.4HG0346310 | 13183.00 | 16227.00 | 12221.00 | 7988.00 | 7428.00 | 5188.00 | 572.18 | 699.27 | 552.20 | 342.37 | 310.36 | 234.25 | 0.00 | (1.03) | down |
| HORVU.MOREX.r2.3HG0255670 | 428.00 | 572.00 | 786.00 | 256.00 | 290.00 | 185.00 | 9.79 | 12.99 | 18.71 | 5.77 | 6.39 | 4.39 | 0.00 | (1.26) | down |
| HORVU.MOREX.r2.7HG0532610 | 974.00 | 2092.00 | 1657.00 | 924.00 | 863.00 | 521.00 | 23.69 | 50.53 | 41.97 | 22.19 | 20.21 | 13.19 | 0.00 | (1.00) | down |
| HORVU.MOREX.r2.4HG0338660 | 107.00 | 130.00 | 166.00 | 58.00 | 81.00 | 54.00 | 5.10 | 6.19 | 8.27 | 2.73 | 3.72 | 2.69 | 0.00 | (1.02) | down |
| HORVU.MOREX.r2.5HG0430510 | 277.00 | 138.00 | 117.00 | 18.00 | 15.00 | 23.00 | 9.47 | 4.68 | 4.14 | 0.59 | 0.47 | 0.81 | 0.00 | (2.75) | down |
| HORVU.MOREX.r2.2HG0083490 | 60.00 | 67.00 | 71.00 | 24.00 | 33.00 | 17.00 | 2.25 | 2.48 | 2.76 | 0.88 | 1.17 | 0.65 | 0.00 | (1.28) | down |
| HORVU.MOREX.r2.1HG0009540 | 306.00 | 280.00 | 425.00 | 165.00 | 163.00 | 148.00 | 3.71 | 3.38 | 5.38 | 1.98 | 1.90 | 1.87 | 0.00 | (1.08) | down |
| HORVU.MOREX.r2.7HG0539380 | 485.00 | 205.00 | 199.00 | 0.00 | 0.00 | 0.00 | 15.65 | 6.57 | 6.69 | 0.00 | 0.00 | 0.00 | 0.00 | (5.74) | down |
| HORVU.MOREX.r2.1HG0053420 | 494.00 | 564.00 | 465.00 | 219.00 | 296.00 | 117.00 | 8.69 | 9.86 | 8.53 | 3.80 | 5.02 | 2.14 | 0.00 | (1.23) | down |
| HORVU.MOREX.r2.1HG0072930 | 841.00 | 1073.00 | 1074.00 | 526.00 | 565.00 | 415.00 | 16.34 | 20.71 | 21.75 | 10.10 | 10.57 | 8.38 | 0.00 | (1.01) | down |
| HORVU.MOREX.r2.2HG0149650 | 84.00 | 83.00 | 63.00 | 26.00 | 33.00 | 22.00 | 1.07 | 1.04 | 0.83 | 0.33 | 0.40 | 0.28 | 0.00 | (1.38) | down |
| HORVU.MOREX.r2.4HG0283150 | 672.00 | 543.00 | 689.00 | 294.00 | 219.00 | 221.00 | 6.40 | 5.14 | 6.83 | 2.76 | 2.00 | 2.18 | 0.00 | (1.37) | down |
| HORVU.MOREX.r2.5HG0386730 | 60.00 | 73.00 | 58.00 | 34.00 | 20.00 | 20.00 | 1.72 | 2.11 | 1.74 | 0.97 | 0.55 | 0.60 | 0.00 | (1.21) | down |
| HORVU.MOREX.r2.3HG0183870 | 71.00 | 95.00 | 80.00 | 36.00 | 46.00 | 34.00 | 0.93 | 1.24 | 1.09 | 0.47 | 0.58 | 0.46 | 0.00 | (1.03) | down |
| HORVU.MOREX.r2.1HG0078230 | 117.00 | 106.00 | 82.00 | 0.00 | 6.00 | 14.00 | 3.72 | 3.34 | 2.71 | 0.00 | 0.17 | 0.46 | 0.00 | (2.68) | down |
| HORVU.MOREX.r2.6HG0507730 | 567.00 | 290.00 | 872.00 | 316.00 | 278.00 | 186.00 | 20.45 | 10.38 | 32.77 | 11.26 | 9.64 | 6.98 | 0.01 | (1.07) | down |
| HORVU.MOREX.r2.7HG0557900 | 473.00 | 234.00 | 317.00 | 0.00 | 0.00 | 0.00 | 20.21 | 9.93 | 14.09 | 0.00 | 0.00 | 0.00 | 0.00 | (6.19) | down |
| HORVU.MOREX.r2.4HG0346630 | 495.00 | 248.00 | 787.00 | 295.00 | 190.00 | 180.00 | 6.42 | 3.19 | 10.63 | 3.78 | 2.37 | 2.42 | 0.01 | (1.10) | down |
| HORVU.MOREX.r2.5HG0438030 | 53.00 | 108.00 | 124.00 | 37.00 | 15.00 | 16.00 | 0.32 | 0.65 | 0.78 | 0.22 | 0.09 | 0.10 | 0.00 | (1.67) | down |
| HORVU.MOREX.r2.6HG0503340 | 548.00 | 259.00 | 565.00 | 219.00 | 200.00 | 156.00 | 4.99 | 2.35 | 5.37 | 1.97 | 1.75 | 1.47 | 0.00 | (1.19) | down |

**Supplementary Table 3. Primers used in the current study**

| Target Genes |  | Primer sequence (5’ - 3’) | Application |
| --- | --- | --- | --- |
| *HvActin* | Forward | TCGCAACTTAGAAGCACTTCCG | qRT-PCR |
|  | Reverse | AAGTACAGTGTCTGGATTGGAGGG | qRT-PCR |
| *Cu/Zn SOD* | Forward | CCCCTCACCAAGTCAGTCAT | qRT-PCR |
|  | Reverse | ATTGCAAGTCGGTGTCCTTC | qRT-PCR |
| *HvCAT1* | Forward | TGGACGGATGGTACTGAACA | qRT-PCR |
|  | Reverse | GTGCCTTTGGGTATCAGCAT | qRT-PCR |
| *HvAPX1* | Forward | CGCCCTCTTGTGGAGAAATA | qRT-PCR |
|  | Reverse | CGCGCATAGTAGCAGCAGTA | qRT-PCR |
| *HvGST6* | Forward | ATCTCGTCAGAAACCCGTTC | qRT-PCR |
|  | Reverse | CTTTCCACGACCACACATTG | qRT-PCR |
| *HvICS* | Forward | AGATTTACGATGGCGGTTTG | qRT-PCR |
|  | Reverse | TTCAGTGAGCTCGAGGAGGG | qRT-PCR |
| *HvERF1* | Forward | GAGGAAGAGCAGAGCGACAC | qRT-PCR |
|  | Reverse | GTCGCCACGAGTATGGTCTT | qRT-PCR |
| *HvWRKY38* | Forward | GTGAAGGACGGGTACCAATG | qRT-PCR |
|  | Reverse | GTCGCCACGAGTATGGTCTT | qRT-PCR |
| *HvPR1a* | Forward | CACACCAAACCCAGAATGGAGA | qRT-PCR |
|  | Reverse | CGTTGTGGGGTGAAAGGTAGT | qRT-PCR |
| *HvERFC3* | Forward | CGTGATGGAGCTTGAGGACCT | qRT-PCR |
|  | Reverse | AGACCGGAGAATCAGATGGAGT | qRT-PCR |
| *HvFme* | Forward | TGCTGGTGACATGATGGAGTTC | qRT-PCR |
|  | Reverse | TTACCAGCTTTCTCTCCGTGG | qRT-PCR |
